# Elucidation of the Pyridine Ring-Opening Mechanism of 2,2′-Bipyridine or 1,10-Phenanthroline Ligands at Re(I) Carbonyl Complexes

**DOI:** 10.1021/acs.inorgchem.3c04434

**Published:** 2024-04-19

**Authors:** Purificación Cañadas, Jesús Díaz, Ramón López, M. Isabel Menéndez, Julio Pérez, Lucía Riera

**Affiliations:** † Departamento de Química Orgánica e Inorgánica, Universidad de Oviedo, Julián Clavería, 8, Oviedo 33006, Spain; ‡ Departamento de Química Orgánica e Inorgánica, Universidad de Extremadura, Avda. de la Universidad s/n, Cáceres 33071, Spain; § Departamento de Química Física y Analítica, Universidad de Oviedo, Julián Clavería, 8, Oviedo 33006, Spain; ∥ Centro de Investigación en Nanomateriales y Nanotecnología (CINN), Consejo Superior de Investigaciones Científicas (CSIC). Avda. de la Vega, 4-6, El Entrego 33940, Spain

## Abstract

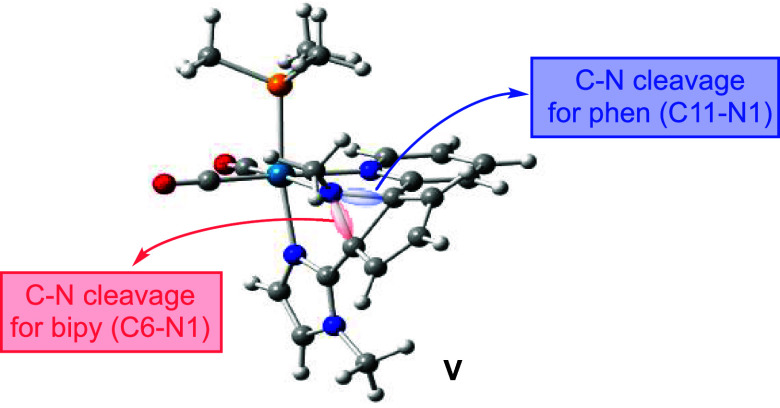

The cleavage of the C–N bonds of aromatic heterocycles, such as pyridines or quinolines, is a crucial step in the hydrodenitrogenation (HDN) industrial processes of fuels in order to minimize the emission of nitrogen oxides into the atmosphere. Due to the harsh conditions under which these reactions take place (high temperature and H_2_ pressure), the mechanism by which they occur is only partially understood, and any study at the molecular level that reveals new mechanistic possibilities in this area is of great interest. Herein, we unravel the pyridine ring-opening mechanism of 2,2′-bipyridine (bipy) and 1,10-phenanthroline (phen) ligands coordinated to the *cis*-{Re(CO)_2_(N-RIm)(PMe_3_)} (N-RIm= *N*-alkylimidazole) fragment under mild conditions. Computational calculations show that deprotonation of the pyridine ring, once dearomatized, is crucial to induce ring contraction, triggering extrusion of the nitrogen atom from the ring and cleavage of the C–N bond. It is noteworthy that different products (regioisomers) are obtained depending on whether the ligand used is bipy or phen due to the additional rigidity and stability conferred by the central ring of the phen ligand, an issue also addressed and clarified computationally. Strong support for the proposed mechanism is provided by the characterization and isolation, including three single-crystal X-ray diffraction structures, of several of the proposed reaction intermediates.

## Abstract 2

Different products (regioisomers) are obtained depending on whether the ligand used is bipy or phen due to the additional rigidity and stability conferred by the central ring of the phen ligand, an issue also addressed and clarified computationally. Strong support for the proposed mechanism is provided by the characterization and isolation, including three single-crystal X-ray diffraction structures of several of the proposed reaction intermediates.

## Introduction

Petroleum crude oils carry small amounts of nitrogen-containing organic compounds that, if not removed, would ultimately result in the emission of nitrogen oxide pollutants and in the poisoning of the catalysts employed for fuel processing. Thus, crude oils are treated with excess hydrogen at high temperatures and pressures in the presence of heterogeneous catalysts, a process called hydrodenitrogenation (HDN), by which those nitrogenated compounds are converted into ammonia and alkanes. The catalysts employed are oxides of transition metals, mostly molybdenum and tungsten, with promoters such as cobalt and nickel.^1−5^ Among the nitrogen-containing organic compounds present in crudes, aromatics such as pyridines and, in particular, quinolines are, because of their stability, the most difficult to remove. Details of the mechanism of HDN are not fully understood, and it is assumed that the metal serves as a Lewis acidic center that binds the organic molecules and also activates dihydrogen to form metal hydrides, which then add to, for instance, the pyridine rings.^6,7^

Only a few examples of pyridine ring-opening mediated by transition metal complexes have been reported. Most of them start with a very rare κ^2^(*C*, *N*) coordination mode of the pyridine derivative to group IV or V metal complexes in the presence of strongly reducing agents.^8−11^ In a different way, Mindiola et al. achieved pyridine ring-opening by a cycloaddition reaction between the pyridine C=N bond and a highly reactive titanium alkylidine intermediate.^12−14^ Using this strategy, these authors were also able to cleave the C–N bond of a fused aromatic N-heterocycle, such as quinoline and isoquinoline,^15^ which is particularly interesting since quinolines are the most abundant and difficult substrates to remove by HDN in the fuels. More recently, Luo and coworkers have reported the extrusion of a nitrogen atom from a pyridine or quinoline ring at a trinuclear titanium heptahydride complex under relatively mild conditions.^16^ In main group chemistry, a few scarce examples on silylenes, which mediate C=N bond cleavage of pyridines, have been reported.^17−19^

**Scheme 1:**
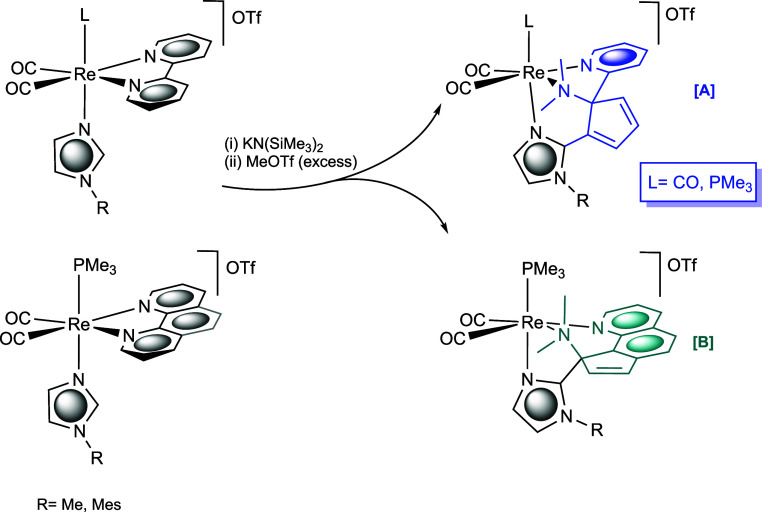
Ring-Opening Reactions of Bipy [**A**] or Phen [**B**] Ligands at Re(I) Carbonyl Fragments to Yield Different Regioisomers Depending on the Specific α-Diimine Ligand

We have shown that 2,2′-bipyridine (bipy) and 1,10-phenanthroline (phen) coordinated to cationic *fac*-rhenium(I) tricarbonyl fragments are not chemically inert ligands and can undergo, under mild conditions, the addition of internal nucleophiles generated, in most cases, by deprotonation of appropriately positioned coligands.^20−24^ In the particular case of the deprotonation of 1-methylimidazole (N-MeIm) coordinated to *fac*-{Re(bipy)(CO)_3_}, followed by the action of excess MeOTf, the pyridine ring contraction concomitant with C–N bond scission was obtained ([**A**] in Scheme 1).^25^ As we have demonstrated more recently, this type of pyridine ring-opening process is favored by more electron-rich *cis*-rhenium(I) dicarbonyl complexes, obtained from *fac*-{Re(CO)_3_} species by carbonyl substitution by a more σ-donor trimethylphosphane ligand. In fact, using *cis*-{Re(CO)_2_} imidazole complexes, we achieved pyridine ring-opening of a coordinated phen (a quinoline model, ([**B**] in Scheme 1), a reaction without precedents.^20^ It is particularly striking that the ring-opening products of bipy or phen ligands display different regiochemistry, so a study of the mechanism of this type of pyridine ring-opening process may lead to extending the knowledge of these very interesting reactions related to HDN processes.

To deepen the knowledge of these types of ring-opening reactions is of prime importance, and herein, we report an extensive and detailed study on the mechanism of pyridine ring-opening of *cis*,*trans*-[Re(CO)_2_(N-N)(N-RIm)(PMe_3_)]OTf (N-N = bipy or phen; N-RIm = *N*-alkylimidazole)^26^ compounds both computationally and experimentally, through the detection of possible reaction intermediates.

## Results and Discussion

As we have briefly mentioned in the Introduction, the addition of a strong base followed by MeOTf (excess) to *cis*,*trans*-[Re(CO)_2_(N-N)(N-RIm)(PMe_3_)]OTf (N-N = bipy or phen; N-RIm= N-MeIm or N-MesIm)^27^ compounds afforded products resulting from pyridine ring-opening of bipy or phen ligands.^20^ It is noteworthy that different regioisomers are obtained depending on the nature of the bidentate ligand, and this fact prompted us to delve deeper into this type of ring-opening process.

### Reaction Mechanism for the Bipy Ligand

**Scheme 2:**
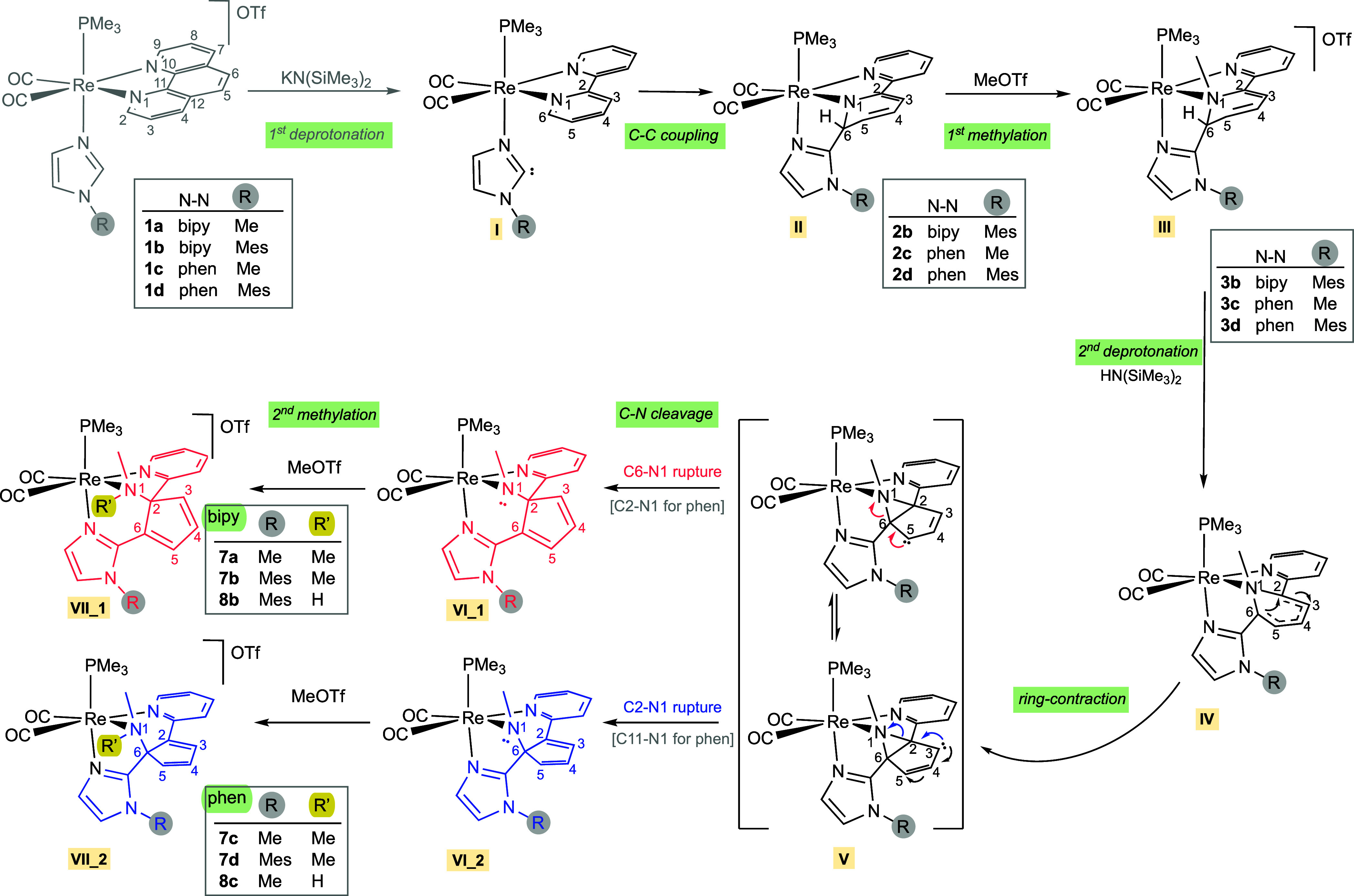
Schematic View (Showing the Numbering Scheme of Bipy and Phen Ligands) of the Steps Involved in the Bipy or Phen Ring-Opening Process in the Reaction of *cis*,*tran*s-[Re(CO)_2_(N-N)(N-RIm)(PMe_3_)]OTf (**1a**-**d**) Compounds with KN(SiMe_3_)_2_ and Excess MeOTf

We started our study by performing a density functional theory (DFT) study for the transformation of compound *cis*,*trans*-[Re(bipy)(CO)_2_(N-MeIm)(PMe_3_)]OTf (**1a**), bearing 2,2′-bipyridine and 1-methylimidazole, into the corresponding ring-opening product. Complex **I**, resulting from the deprotonation of the imidazole central CH group of compound **1a**, which is assumed to be the first step in the reaction (see Scheme 2), has been taken as the starting critical structure. The investigated process entails the evolution of deprotonated complex **I** in the presence of HN(SiMe_3_)_2_ (generated in the deprotonation of **1a** with KN(SiMe_3_)_2_ and MeOTf. The energies of the three species (**I**, HN(SiMe_3_)_2_, and MeOTf) were taken into account to set an energy reference common to all reaction steps. Henceforth, unless otherwise stated, only relative Gibbs free energies will be provided. However, the computational modeling for each step involves only the species that are active in it, and the energy of the isolated nonactive moieties has been eventually added.^28^

A schematic view of the most plausible reaction mechanism is shown in Scheme 2, which, according to our CPCM-B3LYP/6-31+G(d)-LANL2DZ calculations (see the Supporting Information), consists of six steps: (1) C–C coupling between the deprotonated central carbon of the imidazole ring (C_im_) and the C6 atom of the bipy ligand, (2) methylation on the nitrogen atom of the dearomatized pyridine ring (N1), (3) deprotonation of the bipy C6–H group by HN(SiMe_3_)_2_, (4) ring contraction of the dearomatized deprotonated pyridyl ring by C2–C6 bond formation, (5) cleavage of a N–C bond (N1–C2 or N1–C6), and (6) second methylation on the extruded nitrogen atom.

**Figure 1:**
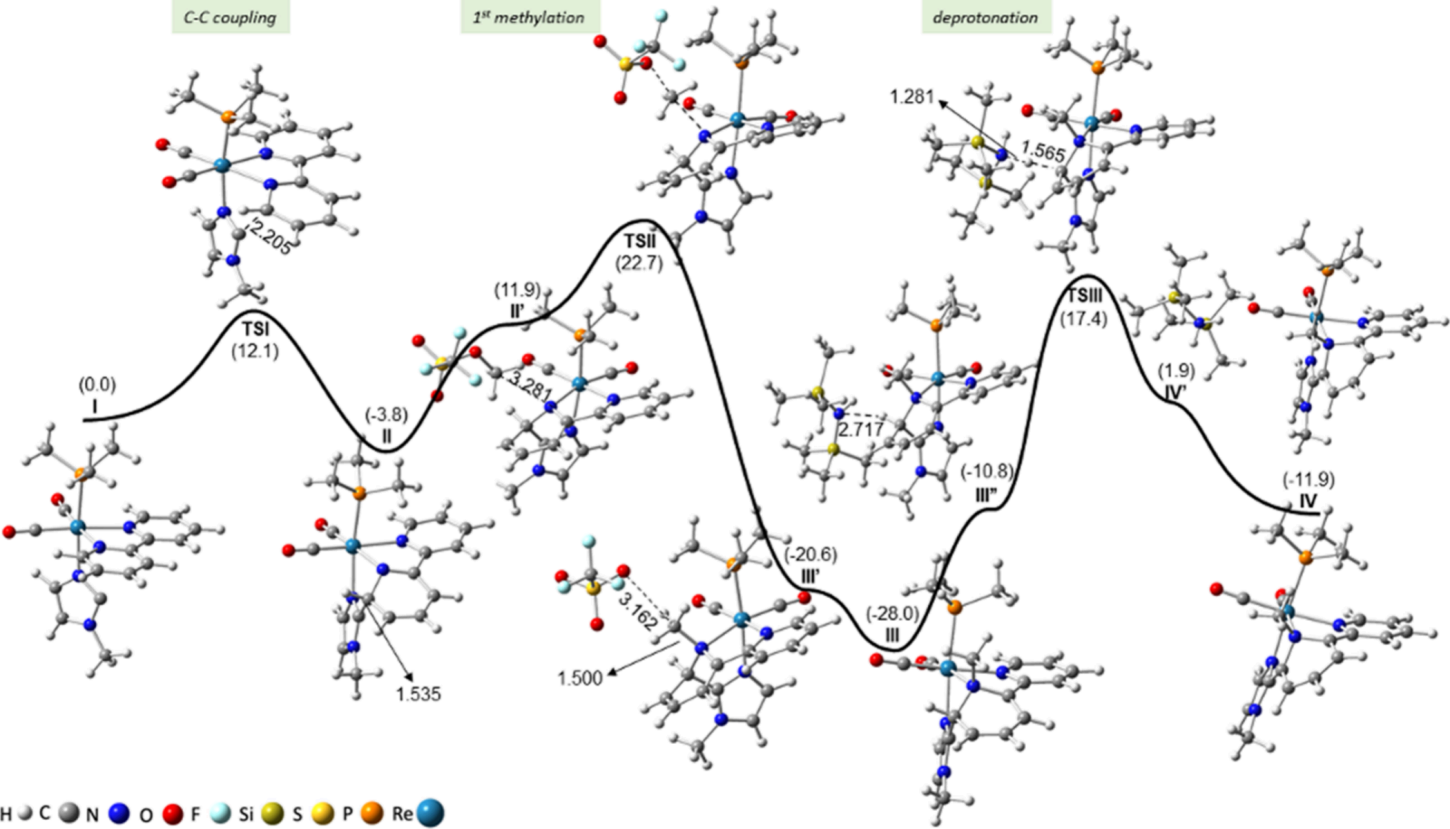
CPCM-B3LYP/6-31+G(d)-LANL2DZ Gibbs energy profile of steps 1–3 involved in the mechanism of the reaction of *cis,trans*-[Re(CO)_2_(bipy)(N-MeIm)(PMe_3_)]OTf (**1a**) with KN(SiMe_3_)_2_ and MeOTf (excess). All energies are given in kilocalories per mole (kcal/mol) and referenced to complex **I**. Some relevant distances (in Å) are also provided.

**Figure 2:**
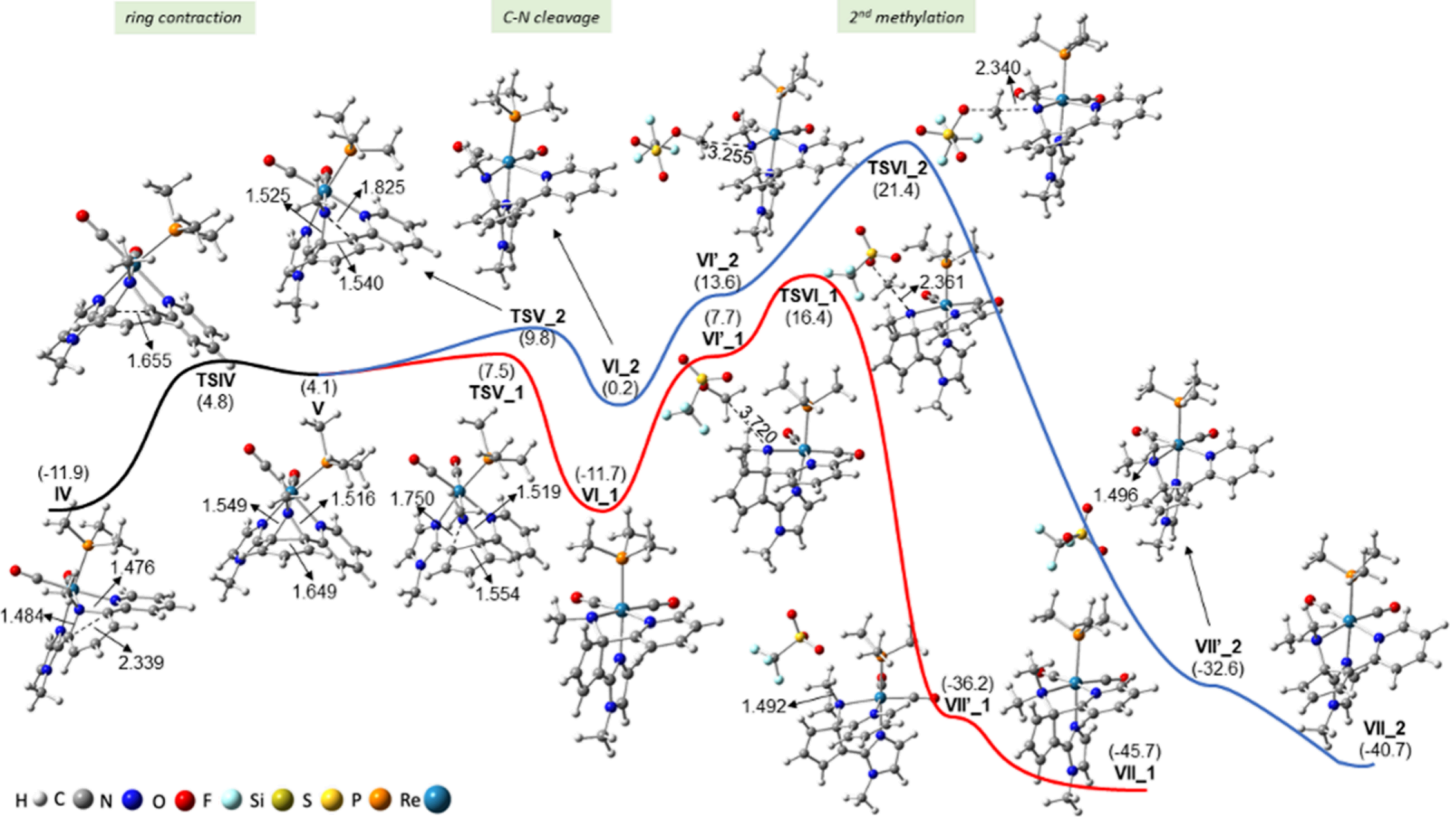
CPCM-B3LYP/6-31+G(d)-LANL2DZ Gibbs energy profile of the steps 4–6 involved in the mechanism of the reaction of *cis*,*trans-*[Re(CO)_2_(bipy)(N-MeIm)(PMe_3_)]OTf (**1a**) with KN(SiMe_3_)_2_ and MeOTf (excess). All energies are given in kcal/mol and referenced to complex **I**. Some relevant distances (in Å) are also provided.

Figure 1 shows the Gibbs energy profile involving steps 1–3, while that for steps 4–6 is collected in Figure 2. Further details on the energy and geometry of the species involved in these energy profiles are given in the Supporting Information (see Tables S1 and S2 and Figure S1). According to our computations, the first step in Figure 1 leads to the formation of intermediate **II** displaying a C_im_–C6 coupling via transition state **TSI**, which determines an energy barrier of 12.1 kcal/mol. Our results indicate that the attacked pyridyl ring becomes dearomatized, while the intact pyridine and imidazole rings hardly change their aromatic character (see Figure S2 in the Supporting Information).^29^ Thus, the stabilization arising from the formation of the C_im_–C6 bond overcomes the destabilization due to the loss of aromaticity of the attacked pyridine ring and explains the slightly higher stability of **II** in comparison with that of complex **I**.

The next step begins with the interaction of MeOTf with intermediate **II** to form the prereactive intermediate **II’**, 15.7 kcal/mol above **II**, which evolves through transition state **TSII** to afford intermediate **III’**, 32.5 kcal/mol more stable than **II’**. The H_3_C···N1 bond distance decreases from 3.281 Å at **II’** to 2.271 and 1.500 Å at **TSII** and **III’**, respectively. The preferential methylation on the nitrogen atom of the dearomatized pyridyl ring (N1) is expected as this amido-like nitrogen atom displays the most basic character at the tridentate ligand in complex **II**, with a natural atomic charge (NAC) of −0.570 e (values of +0.172, −0.365, −0.237, −0.283, and −0.130 e were found for the C2, C3, C4, C5, and C6 atoms, respectively). The methylation is controlled by **TSII** and determines an energy barrier of 10.8 kcal/mol measured from **II’**. Complete release of the triflate ion from **III’** leads to the very stable Re(I) monomethylated complex **III**, which is even 7.4 kcal/mol more stable than **III’** (see Figure 1). Afterward, HN(SiMe_3_)_2_, generated in the deprotonation of the coordinated N-MeIm [the reaction that generates the neutral complex **I** from cationic starting *cis*,*trans*-[Re(bipy)(CO)_2_(N-MeIm)(PMe_3_)]OTf (**1a**) and KN(SiMe_3_)_2_], deprotonates the bipy C6–H group. The approach of HN(SiMe_3_)_2_ to **III** affords the prereactive intermediate **III”** (17.2 kcal/mol less stable than **III**), which, in turn, evolves to complex **IV’** through transition state **TSIII** (28.2 and 45.4 kcal/mol above **III’** and **III**, respectively). The relatively large Gibbs energy barrier in solution of **TSIII** referred to **III** is due to an overestimation of the entropy contribution caused by the molecularity change (Δ*n* ≠ 0).^30^ The computationally affordable model used for intermediate **III** does not fully correspond to the actual scenery in the reaction medium since its interaction with OTf^–^ and HN(SiMe_3_)_2_ moieties (*n* = 3) has not been considered, which has the drawback of overestimating its stabilization in Gibbs energy. Thus, the actual Gibbs energy barrier of **TSIII** is presumably lower than that shown in Figure 1 (see the Supporting Information for more details). **IV’** leads to the formation of H_2_N(SiMe_3_)_2_^+^ ion and intermediate **IV**, which is 13.8 kcal/mol more stable than **IV’**, in part ascribed to the entropic gain.

As depicted in Figure 2, intermediate **IV** evolves by C–C coupling of C2 and C6 atoms, yielding a contraction of that ring via transition state **TSIV**. The C6···C2 distance varies from 2.339 Å at **IV** to 1.655 and 1.649 Å at **TSIV** and **V**, respectively. As a result, intermediate **V** displays a bicyclic moiety containing a five-membered ring fused to a *N*-methylated aziridine. This rearrangement involves a notable lengthening of the C6–N1 and C2–N1 bond distances by 0.065 and 0.040 Å, respectively, when going from **IV** to **V**. Reaching **TSIV** involves surmounting an energy barrier of 16.7 kcal/mol, which almost coincides with the Gibbs energy of **V**, and the geometries of both species, **TSIV** and **V**, are also very similar.^31^ So, the transformation of **TSIV** into **V** is easy and occurs very fast. Intermediate **V** may undergo cleavage of either the C6–N1 bond (route_1, in red in Figure 2) or the C2–N1 bond (route_2, in blue in Figure 2). The C–N bond distances (1.549 and 1.516 Å for C6–N1 and C2–N1, respectively), the electron density (ρ) at the bond critical points (BCPs) of C–N bonds obtained in a Bader topological analysis of the electron density (of 0.2170 and 0.2337 e/Å^3^ for C6–N1 and C2–N1, respectively), and the Wiberg indexes (WI) of C–N bonds (0.779 and 0.818 for C6–N1 and C2–N1, respectively) all point to the easier rupture of the C6–N1 bond. Consistent with this, the C6–N1 cleavage via transition state **TSV_1**, 3.4 kcal/mol higher in energy than **V**, is less energetically demanding than the cleavage of the C2–N1 bond through **TSV_2** by 2.3 kcal/mol. It is interesting to note that the C–N1 bond, which is broken (C6–N1 or C2–N1), determines the orientation of the resulting cyclopentadienyl ring and therefore the conformation of the resulting tridentate ligand. Specifically, at intermediate **VI_1**, the mentioned cyclopentadienyl is nearly coplanar with the imidazole ring, whereas that at intermediate **VI_2** is almost in the same plane as the pyridine ring, the former rearrangement being favored by 11.9 kcal/mol (see Figure 2). NICS(1) indices of −9.1, −3.3, and −9.5 ppm were found for the imidazole, cyclopentadienyl, and pyridine rings at **VI_1**, respectively, while values of −9.1, −3.7, and −8.2 ppm were obtained at **VI_2** (see Figure S3 in the Supporting Information).^32^ Therefore, the loss of aromaticity and π-electron delocalization of the pyridine ring by 1.3 ppm seems to be partly responsible for the lower stability of **VI_2** in comparison to **VI_1**. The cleavage of the C–N1 bond (C6–N1 or C2–N1) increases the negative NAC of N1 (by 0.188 or 0.167 e when comparing **VI_1** or **VI_2** with **V**, respectively), favoring the incorporation of a second methyl moiety on this atom by reaction with MeOTf (a reagent that is in excess), which is the last step of the reaction mechanism. Transition states **TSVI_1** and **TSVI_2** control this second methylation process, and they are 8.7 and 7.8 kcal/mol less stable than the prereactive intermediates **VI’_1** and **VI’_2** and yield intermediates **VII’_1** and **VII’_2**, respectively (see the evolution of methyl–N1 bond distances in Figure 2). Finally, intermediates **VII’_1** and **VII’_2** led to the corresponding dimethylated ring-opening products **VII_1** and **VII_2**, respectively, the former being more stable by 5 kcal/mol than the latter (see Figure 2). It is therefore interesting to note that, considering intermediate **IV** as a reference, the C6–N1 bond cleavage pathway (route _1, in red in Figure 2) presents an energy barrier of 28.3 kcal/mol, while a value of 33.3 kcal/mol is required in the C2–N1 bond cleavage pathway (route_2, in blue in Figure 2). Thus, complex **VII_1** is the kinetically and thermodynamically preferred product, and it is, indeed, the product obtained experimentally (**7a** in Scheme 2).

Other reaction mechanisms were explored but showed higher rate-determining energy barriers than those found in the reaction mechanism described above (see Figures S4 and S5 in the Supporting Information).

Experimental isolation of any of the intermediates in the bipy ring-opening reaction of *cis*,*trans*-[Re(bipy)(CO)_2_(N-MeIm)(PMe_3_)]OTf (**1a**), the one computationally studied in detail, was not possible due to their low stability. As we have observed previously, derivatives starting from the analogous N-MesIm compound were notably more stable;^21,25^ so, we studied the reactivity of *cis*,*trans*-[Re(bipy)(CO)_2_(N-MesIm)(PMe_3_)]OTf (**1b**), and some of the intermediates could be, at least, spectroscopically characterized in solution. Thus, the addition of a slight excess of the strong base KN(SiMe_3_)_2_ to a THF solution of **1b** at −78 °C afforded immediately deprotonation of the central imidazole CH group and formation of the C–C coupling species **2b** (Scheme 2; **2b** is the mesityl analogue of intermediate **II**, computationally obtained). The shift to lower wavenumbers of the IR ν_CO_ bands (from 1928 and 1848 cm^–1^ in **1b** to 1894 and 1816 cm^–1^ in **2b**) is in agreement with the formation of a neutral complex, and NMR spectra in CD_2_Cl_2_ solution clearly show the formation of a dearomatized product (Figures S12–S16 in the Supporting Information). The ^1^H NMR spectrum of **2b** at 233 K (Figure S12 in the Supporting Information) shows the loss of the mirror plane of the starting compound **1b**, showing one signal for each bipyridine hydrogen. In particular, the chemical shifts of the H5 and H6, at 5.20 and 4.18 ppm, respectively, are clearly indicative of a dearomatized product. The ^13^C NMR spectrum (Figure S13 in the Supporting Information) displays a signal at 62.4 ppm corresponding to the bipy C6 atom, which is in agreement with the dearomatization of that pyridyl ring as a consequence of the C–C coupling between the imidazole central carbon atom and bipy C6.

Addition of the equimolar amount of MeOTf to a solution of neutral dearomatized compound **2b** in CH_2_Cl_2_ afforded in 30 min at room temperature the cationic monomethylated compound **3b** (Scheme 2). The ^1^H NMR spectrum of **3b** (Figure S29 in the Supporting Information) provides the first evidence that the methylation of the amido-type nitrogen atom of the dearomatized product has been achieved. The spectrum displays the characteristic signals resulting from the protons of an asymmetric, nonaromatic bipyridine ligand (eight one-hydrogen signals, those at 5.16 and 4.97 ppm clearly indicating dearomatization) and a new three-hydrogen singlet at 3.19 ppm that corresponds to the new methyl group incorporated to the complex. The ^13^C NMR spectrum (Figure S30 in the Supporting Information) is in agreement with an asymmetric complex as clearly evidenced, for example, by one signal for each carbonyl ligand, at 209.4 and 207.1 ppm. The maintenance of the dearomatized nature of the bipy ligand is supported by the upfield shifted signal that corresponds to C6 (at 65.5 ppm), and a signal at 47.1 ppm is indicative of the incorporation of the new methyl group to the molecule. In addition, as a consequence of the methylation reaction, the metal complex is now cationic, as it is clearly shown by the IR ν_CO_ bands that are notably shifted to higher wavenumber values (to 1927 and 1849 cm^–1^ in **3b**).

The experimental reaction of *cis*,*trans*-[Re(bipy)(CO)_2_(N-MesIm)(PMe_3_)]OTf (**1b**) with the equimolar amount of KN(SiMe_3_)_2_ followed by the addition of MeOTf (excess) has been recently published, showing the formation of the ring-opening product **7a**, which is the experimental analog of intermediate **VII_2**.^20^

The feasibility of the computationally proposed mechanism is strongly supported by the isolation and characterization of compounds **2b** and **3b**.

### Effect of Using Phen instead of Bipy on the Reaction Mechanism

For the sake of completeness, we decided then to study the mechanism of the ring-opening reaction of the analogous phen compound *cis*,*trans*-[Re(CO)_2_(N-MeIm)(phen)(PMe_3_)]OTf (**1c**). According to our calculations, the steps of the reaction mechanism are mainly the same, but in this case, the thermodynamically and kinetically preferred product is a regioisomer of that obtained for the bipy ligand. In other words, whereas for bipy, it is more favorable the cleavage of the C6–N1 bond to afford the **VII_1** product, for phen, the other regioisomer, **VII_2**, resulting from the scission of C2–N1 (C11–N1 in the numbering scheme of phen, see Scheme 2) is the preferred one. All species found for the phen case will be denoted with the same acronym as their bipy analogues but adding the subscript **p** at the end (*i.e.,* intermediate **I**_**p**_ is the phen analogue of intermediate **I**).

The reaction mechanism from compound **1c** to intermediate **V**_**p**_ (steps 1–4) is very similar to that described above for bipy complex **1a**, so it is depicted in detail in the Supporting Information (Figure S6). It is interesting to note that all the species involved are slightly more stable with respect to their bipy analogues, and the C–C coupling step is more favorable (by 2.4 kcal/mol), reflecting the more electrophilic character of phen compared to bipy. In contrast, the ring contraction of the affected pyridyl ring once the second deprotonation has occurred (step from **IV**_**p**_ to **V**_**p**_) is more energetically demanding than for bipy (by 2.1 kcal/mol), probably due to the rigidity imposed by the central ring of the phen ligand.

**Figure 3:**
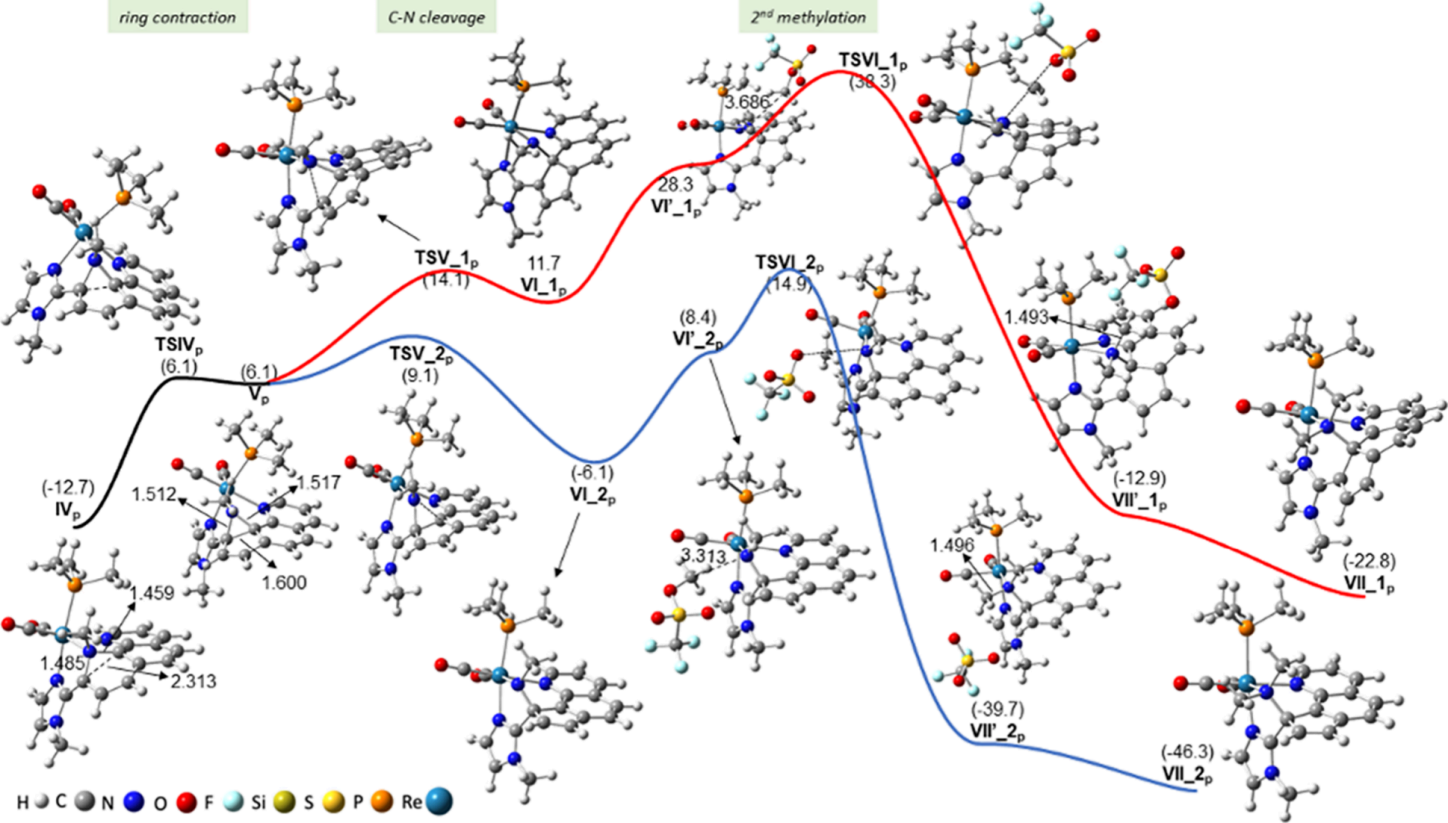
CPCM-B3LYP/6-31+G(d)-LANL2DZ Gibbs energy profile of the C–N cleavage and second methylation (fifth and sixth) steps involved in the mechanism of the reaction of *cis*,*trans*-[Re(CO)_2_(N-MeIm)(phen)(PMe_3_)]OTf (**1c**) with KN(SiMe_3_)_2_ and MeOTf (excess). To facilitate comparison with Figure 2, the fourth step (ring contraction) has also been included. All energies are given in kcal/mol and referenced to complex **I**_**p**_. Some relevant distances (Å) are also provided.

Regarding the C–N bond cleavage and the second methylation step (steps 5 and 6, see Figure 3), there is a substantial difference between the energy profiles for phen and bipy ligands. Cleavage of the C2–N1 bond (C6–N1 for bipy; route_1, in red in Figure 3) allows the resulting cyclopentadienyl moiety to lie almost coplanar with the imidazole ring at the **VI_1**_**p**_ intermediate, but as a consequence, C11 acquires an sp^3^ hybridization, and therefore, the central benzene ring of phen is forced to lose the aromaticity [NICS(1) value of 0.3 ppm compared to −10.2 ppm obtained for isolated benzene].^33^ As a result, a high energy barrier is required (26.8 kcal/mol, relative to **IV**_**p**_), and the resulting intermediate **VI_1**_**p**_ is highly unstable (24.4 kcal/mol referred to **IV**_**p**_).

The situation is even worse at the second methylation process, defined by a transition state (**TSVI_1**_**p**_) with a very high energy barrier of 51.0 kcal/mol, thus precluding the formation of the product **VII_1**_**p**_ (the one experimentally obtained and computationally preferred for the bipy analog). It has to be noted that phen species exhibit markedly higher relative energies, ranging from 20.6 to 23.3 kcal/mol, than their bipy analogues, which can be attributed to the fact that for the latter, the formation of this type of product does not entail a loss of aromaticity of any of its rings. The C11–N1 bond scission route (route_2, in blue in Figure 3; C2–N1 for bipy) is much more favorable than the route_1, just mentioned. The C–N bond rupture and the second methylation steps, controlled by transition states **TSV_2**_**p**_ and **TSVI_2**_**p**_, have energy barriers of 21.8 and 27.6 kcal/mol, respectively, which are 5.0 and 23.4 kcal/mol lower in energy than the corresponding energy barriers for route_1. The C2–N1 and C11–N1 bond distances (of 1.512 and 1.517 Å, respectively), the electron density at the bond critical points of C2–N1 and C11–N1 bonds obtained in a Bader topological analysis of the electron density (0.2355 and 0.2306 e/Å^3^, respectively), and the Wiberg indexes of C2–N1 and C11–N1 bonds (0.830 and 0.818, respectively) all point to the easier breakage of the C11–N1 bond. A huge preference for the dimethylated product **VII_2**_**p**_ over **VII_1**_**p**_ is observed (23.5 kcal/mol). In the former, no dearomatization of the central ring of the phen ligand is forced, but the resulting cyclopentaquinoline moiety displays a high degree of electron conjugation and planarity, two fundamental aspects that should contribute significantly to the stability of this species.

Therefore, for the phen ligand, computations predict, both kinetically and thermodynamically, a dimethylated product whose regiochemistry is different from that expected for the bipy analogue, which is indeed the one experimentally obtained (**7c** in Scheme 2). The rate-determining transition state of the C11–N1 cleavage route, **TSVI_2**_**p**_, is 23.4 kcal/mol more stable than that of the C2–N1 one, **TSVI_1**_**p**_, and the product **VII_2**_**p**_ is 23.5 kcal/mol more stable than **VII_1**_**p**_ (see Figure 3). We had previously observed that the replacement of bipyridine by phenanthroline at complexes derived from *fac*-{Re(CO)_3_(N-RIm)} fragments led to a notable enhancement of the stability of the resulting complexes, probably due to the additional stability provided by the central ring of the phenanthroline ligand.^25^ We decided then to try to detect any of the intermediates in the ring-opening reaction of the phenanthroline, especially with the N-MeIm ligand, as was not possible with the analogous bipy complex.

**Figure 4:**
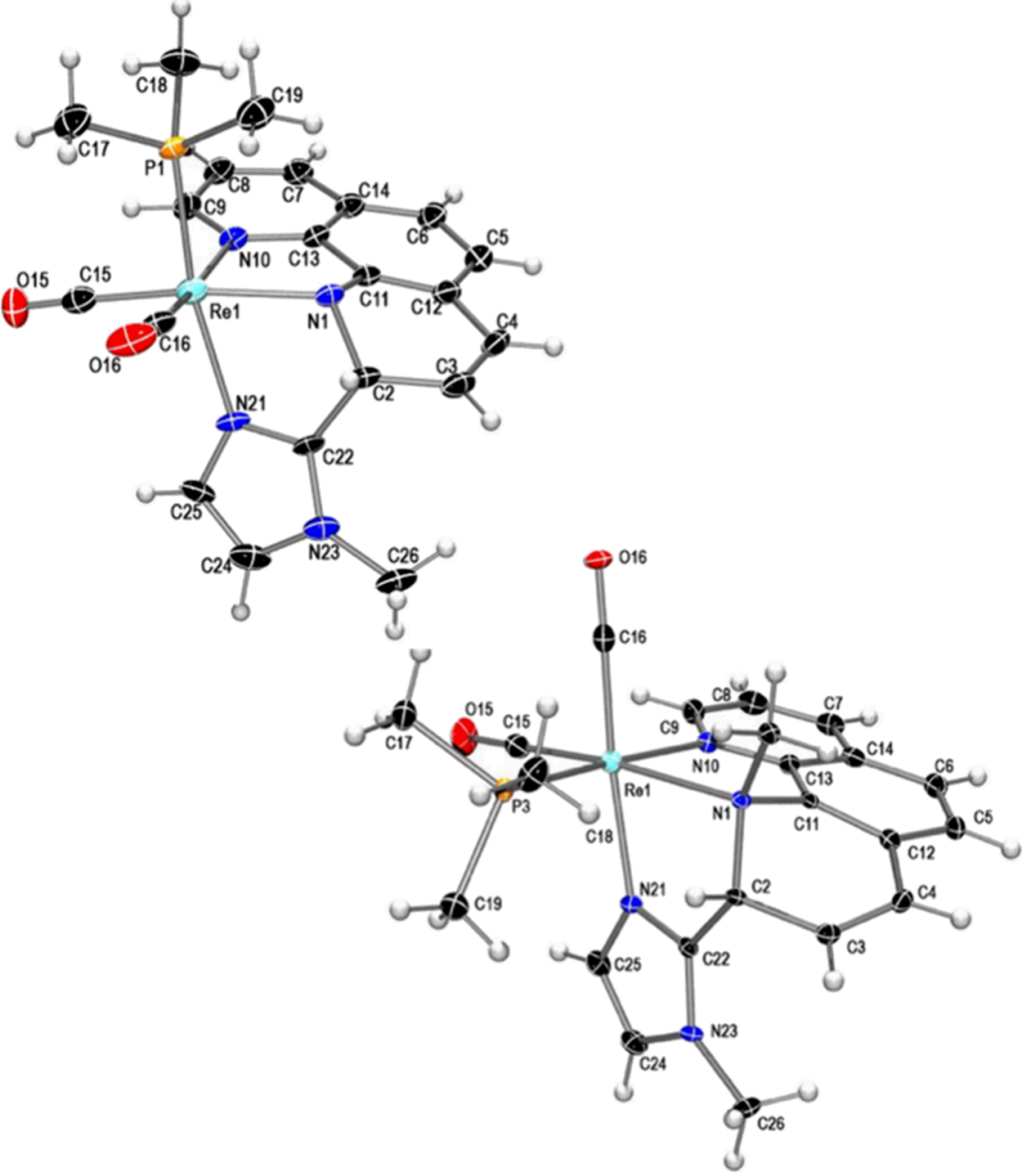
Molecular structure of complex **2c** (top) and of the cation of compound **3c’** (bottom) showing thermal ellipsoids at a 30% probability level.

The deprotonation of *cis,trans*-[Re(CO)_2_(N-RIm)(phen)(PMe_3_)]OTf (R = Me, **1c**; Mes, **1d**) resulted, in both cases, in the formation of species much more stable than those observed in the deprotonation of the complexes with bipy, and they could be isolated as brown solids and spectroscopically characterized in solution. The new compounds, **2c** and **2d**, are the products resulting from the C–C coupling between imidazole and phenanthroline ligands, the latter thus becoming dearomatized (Scheme 2). This fact is clearly evidenced in the CD_2_Cl_2_ solution NMR spectra (Figures S17–S28 in the Supporting Information), which feature for example, in the ^1^H NMR spectrum of **2c** (the experimentally isolated compound corresponding to intermediate **II**_**p**_ in Scheme 2), eight signals for the phen ligand, indicating the lack of symmetry of the molecule. In addition, the chemical shift of two of these signals, at 5.65 and 5.97 ppm, allows us to propose the dearomatization of one of the pyridine rings of phen (Figure S17 in the Supporting Information). The ^13^C NMR spectra of these neutral derivatives (**2c**,**d**), shown in Figures S18 and S24, respectively, in the Supporting Information, are consistent with the geometry proposed for these products. In both cases, the most informative signal is the one corresponding to the C2 carbon of the phen ligand at 61.5 ppm for **2c**, and at 63.1 ppm for **2d**. These chemical shifts are consistent with an sp^3^ hybridization of this carbon, as a result of the nucleophilic attack of the deprotonated imidazole, causing the dearomatization of the pyridyl ring of phen. The molecular structure of complex **2c**, determined by single-crystal X-ray diffraction, confirmed the tridentate N-donor ligand formed by the coupling between the central carbon of the imidazole (C22) and one of the *ortho* phen carbons (C2) as shown in Figure 4 (top). As a consequence, the phen is dearomatized in one of its rings, as evidenced by the bond distances and angles around the C2 carbon as well as the loss of planarity of the affected pyridine ring. The angles around carbon C2 indicate that its geometry is approximately tetrahedral, and the nitrogen atom of the activated pyridine ring has changed from belonging to a neutral imine-type ligand to an anionic amido-type ligand. This fact is reflected not only by the distance Re1–N1 (2.173 (6) Å) which is slightly shorter than the distance Re1–N10 (2.200 (5) Å) but also, more importantly, because the geometry around the nitrogen atom N1 is no longer planar, with the sum of the angles around N1 being 338.9°, indicative of a pyramidal geometry.

The reaction of the neutral complex **2c** with the equimolar amount of MeOTf in CH_2_Cl_2_ produced a notable shift of the IR bands to higher wavenumber ν_CO_ values (from 1891 and 1808 cm^–1^ to 1923 and 1844 cm^–1^), consistent with the formation of a cationic product. The ^31^P NMR spectrum in CD_2_Cl_2_ indicated, by the presence of two signals at −21.2 and −25.3 ppm, the formation of two Re(I) products (**3c** and **3c’**), and accordingly, the ^1^H NMR spectrum showed the presence of two dearomatized C–C coupling products, which could be separated by fractional crystallization. The molecular structure of **3c’**, determined by X-ray diffraction (Figure 4, bottom), was found to correspond to a C–C coupling product bearing a dearomatized pyridine ring of phen that has been methylated on the nitrogen atom (N1). As a result, it has changed from an amido type (in the neutral species, **2c**) to amino type (in **3c’**), which is clearly reflected in a lengthening of the Re1–N1 distance (from 2.173(6) Å in **2c** to 2.284(5) Å in **3c’**). Surprisingly, the solid-state structure of **3c’** shows that the complex has undergone isomerization, and the PMe_3_ ligand, initially in *trans* to the imidazole, is now in *cis* disposition. The spectroscopic characterization of the other product, **3c**, indicated a high similarity with compound **3c’**. In particular, NMR data in solution (Figures S34–S38 in the Supporting Information) display the same pattern of signals, indicating the presence of a dearomatized phen and the incorporation of a methyl group to the molecule. In fact, the signal of that methyl group in the ^1^H NMR spectrum (Figure S34 in the Supporting Information) is very informative since it is a small coupling constant doublet (at 3.44 ppm, ^4^*J*_HP_= 1.9 Hz), which allows to propose that **3c** is the isomer of **3c’** in which the phosphane has not changed its position (in **3c’**, that CH_3_ signal is a singlet at 3.41 ppm, see Figure S39 in the Supporting Information).^34^ Compound **3c** corresponds to intermediate **III**_**p**_ in Scheme 2 for the phenanthroline ring-opening reaction. The methylation reaction of the analogous mesityl complex **2d** with the equimolar amount of MeOTf afforded compound **3d** as the main product. Formation of the complex analogous to **3c’** (in which the phosphane and N-MesIm ligands would be in *cis* disposition) is not observed, probably because of the higher bulk of the mesityl substituent.

The reaction of *cis,trans*-[Re(CO)_2_(N-MeIm)(phen)(PMe_3_)]OTf (R = Me, **1c**) with KN(SiMe_3_)_2_ and MeOTf (excess) afforded the phenantroline ring-opening product **7c**, as we have already published.^20^ It has to be noted that another pyridine ring-opening product had been obtained in this reaction, and it was found to be a product in which the extruded nitrogen becomes both protonated and methylated (**8c** in Scheme 2), instead of doubly methylated as in **7c**.^35^ Taking into account the computational results on the mechanism of the ring-opening reaction, it is reasonable to think that in the last step of the reaction, the H_2_N(SiMe_3_)_2_^+^ ammonium cation, generated in step 3, can be competitive as an electrophile with MeOTf and could lead to a protonation instead of a second methylation.

Theoretical computations corroborated this hypothesis, and indeed, the H_2_N(SiMe_3_)_2_^+^ ammonium moiety could afford the protonation of the nitrogen atom N1 at **VI_1**_**p**_ and **VI_2**_**p**_ through transition states (**TSVII_1**_**p**_ and **TSVII_2**_**p**_), which are 0.2 and 6.7 kcal/mol lower in energy than those corresponding to the second methylation (**TSVI_1**_**p**_ and **TSVI_2**_**p**_, respectively).^36^ However, the protonated-methylated products, **VIII_1**_**p**_ and **VIII_2**_**p**_, are 12.8 and 12.5 kcal/mol higher in energy than those resulting from the second methylation, **VII_1**_**p**_ and **VII_2**_**p**_, respectively. Accordingly, looking at the route_2, which is the most favored for phen, the protonated-methylated product is kinetically preferred over the dimethylated one, while the latter is the thermodynamically favored one. So, it is reasonable to expect obtaining a mixture of both compounds **VII_2**_**p**_ and **VIII_2**_**p**_.^36^

**Scheme 3:**
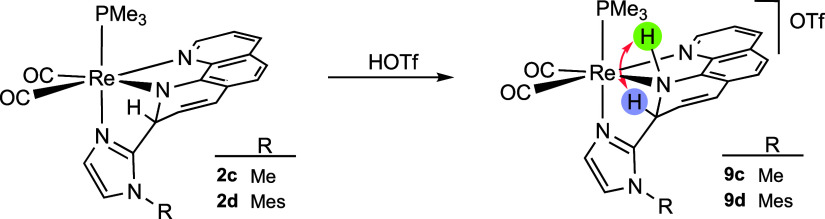
Protonation of the Amido-like Nitrogen Atom of the Dearomatized Phen Ligand in the Reaction of Complexes **2c,d** with HOTf

As a proof of concept for the reaction with an acid reagent, we tried the reaction of neutral dearomatized phenanthroline species **2c** or **2d**, bearing an amido-type nitrogen, with HOTf (Scheme 3). In both cases, the reaction took place almost immediately to yield the products of protonation onto the nitrogen atom **9c** or **9d**, respectively.

**Figure 5:**
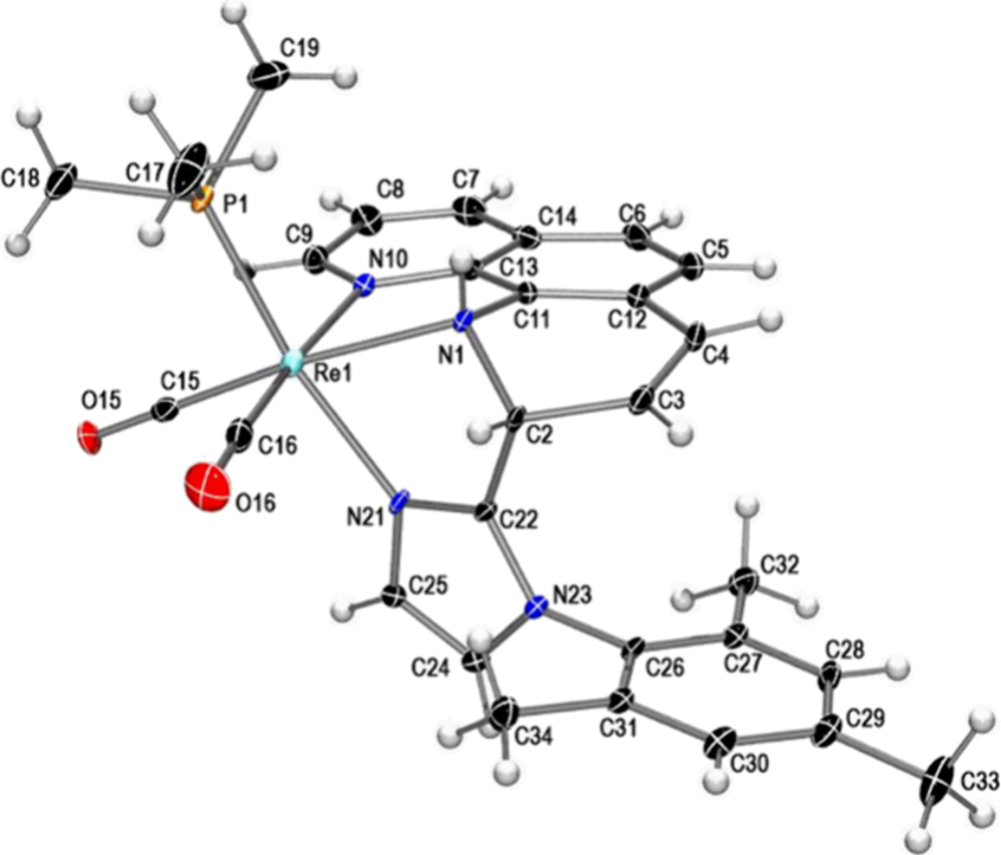
Molecular structure of the cation of compound **9d** with thermal ellipsoids at the 30% probability level.

In the ^1^H NMR spectra of both compounds **9c**,**d** in CD_2_Cl_2_ (Figures S51 and S56 for compounds **9c** and **9d**, respectively, in the Supporting Information), it is clearly shown that the deromatization present in the neutral precursor complexes is maintained. Eight signals of one hydrogen each, corresponding to an asymmetric phenanthroline ligand, are observed, and two of them are particularly upfield shifted (at 5.55 and 5.26 ppm for compound **9d**, for example), which clearly indicate the dearomatization of this ligand. This allows us to rule out the possibility that the protonation of the central carbon of the imidazole has taken place, with the consequent cleavage of the C–C bond, to regenerate the starting imidazole complex. The ^1^H NMR spectra also show a wide and low intensity singlet at 7.73 ppm in the case of **9c** (and at 7.65 ppm for **9d**), which is assigned to the NH group resulting from the protonation. Accordingly, the COSY spectra of these species (Figures S54 and S59 for compounds **9c** and **9d**, respectively, in the Supporting Information) show a strong correlation between the signal that we assigned to the NH group and that corresponding to the H2 hydrogen of the phenanthroline ligand (Scheme 3). Figure 5 shows the molecular structure of the cation present in compound **9d**. The tridentate ligand formed by the coupling between the imidazole and phenanthroline, which became dearomatized, is *fac*-coordinated to the *cis*-{Re(CO)_2_(PMe_3_)} fragment. The nitrogen atom N1 displays a pyramidal geometry, with the sum of the angles around this atom being 327.4°. This fact, together with the Re1–N1 distance of 2.251(4) Å, typical for an amino-type nitrogen in this type of compound, and the fact that it is a cationic metal allow us to propose that the protonation has taken place on this nitrogen atom, N1.

## Conclusions

In conclusion, the ring-opening mechanism of a pyridine moiety of bipy or phen ligands at electron-rich *cis*,*trans*-[Re(CO)_2_(N-N)(N-RIm)(PMe_3_)]OTf (**1a**–**d**) compounds has been understood. The addition of the strong base KN(SiMe_3_)_2_ causes the deprotonation of the central imidazole CH group, which adds to the *ortho* C6 bipy atom (C2 in the case of phen) to afford a dearomatized C–C coupling product (compounds **2b**–**d** fully experimentally characterized). Computations reveal that this dearomatization reaction is slightly more favorable for the phen ligand due to its more electrophilic character compared to bipy. The presence of the equimolar amount of MeOTf leads to the methylation of the amido-type nitrogen atom in neutral dearomatized complexes, yielding monomethylated cationic compounds (**3b**–**d**), which are remarkably stable according to DFT calculations. On the other hand, in the presence of an excess of MeOTf and HN(SiMe_3_)_2_ (resulting from the deprotonation reaction of the imidazole) in the reaction medium, a series of processes are initiated leading to the pyridine ring-opening products of the bipy or phen ligands (**7a**–**d**). Thus, after the first methylation, there is a deprotonation of the C6–H group of bipy by HN(SiMe_3_)_2_ (C2–H group in the case of phen) followed by a contraction of the pyridine ring upon the formation of the C6–C2 bond (C2–C11 in the case of phen), leading to the extrusion of the nitrogen atom, which is methylated a second time.

The theoretical calculations show that the ring contraction step involves the formation of intermediate **V**, which is a complex with a bicyclic moiety containing a five-membered ring fused to an *N*-methylated aziridine. Intermediate **V** is crucial in explaining the different products obtained depending on whether the ligand used is bipy or phen, as it is determined by the C–N bond that is broken in this intermediate. In the case of the bipy ligand, the most kinetically and thermodynamically favorable product is that resulting from the cleavage of the C6–N1 bond (**VII_1**, resulting from route_1). In contrast, for phen, the formation of the analogous product (**VII_1**_**p**_, resulting from the cleavage of the C2–N1 bond) is much less favorable (e.g., it is 23.4 kcal/mol less stable) than that obtained by cleavage of the C11–N1 bond (**VII_2**_**p**_, resulting from route_2). This is because the presence of the central ring of the phen ligand makes the formation of intermediate **VI_1**_**p**_ (17.8 kcal/mol less stable than **VI_2**_**p**_) especially unfavorable because it would also force the dearomatization of such a benzene ring with the concomitant destabilization.

## Supporting Information Available

Synthetic and computational details, including preparative procedures, spectroscopic data, computational Cartesian coordinates, and NMR spectra for the characterization of compounds (PDF)
