# Supplementary material for: Elucidation of the Pyridine Ring-Opening Mechanism of 2,2′-Bipyridine or 1,10-Phenanthroline Ligands at Re(I) Carbonyl Complexes
Source: Inorg Chem. 2024 Apr 19;63(19):8593–603. doi: 10.1021/acs.inorgchem.3c04434 (PMC11661672; doi:10.1021/acs.inorgchem.3c04434)

Supporting Information

Elucidation of pyridine ring-opening mechanism of 2,2'-bipyridine or 1,10-phenanthroline ligands at Re(I) carbonyl complexes

Purificación Cañadas,[a] Jesús Díaz,[b] Ramón López,*[c] M. Isabel Menéndez,[c] Julio Pérez[a,d] and Lucía Riera*[d]*

[a] Departamento de Química Orgánica e Inorgánica, Universidad de Oviedo, Julián Clavería, 8. 33006 Oviedo (Spain).

[b] Departamento de Química Orgánica e Inorgánica, Universidad de Extremadura, Avda. de la Universidad s/n. 33071 Cáceres (Spain).

[c] Departamento de Química Física y Analítica, Universidad de Oviedo, Julián Clavería, 8. 33006 Oviedo (Spain).

[d] Centro de Investigación en Nanomateriales y Nanotecnología (CINN), Consejo Superior de Investigaciones Científicas (CSIC). Avda. de la Vega, 4-6, 33940 El Entrego (Spain). E-mail: l.riera@cinn.es

Table of Contents

Computational Details. **S4**

1. Methods and technical details S4

2. Discussion about the overestimation of the Gibbs energy barrier in solution for the deprotonation step S4

Figure S1. Optimized structures of the species involved in the reaction of *cis,trans*-[Re(bipy)$(CO)_2$(*N*-MeIm)(PMe_3)]OTf with KN($SiMe_3$)$_2$ and MeOTf in dichloromethane solution. S6

Figure S2. NICS(1) values for the imidazole and pyridine rings of complexes **I** and **II**. S16

Figure S3. NICS(1) values for the imidazole, cyclopentadiene, and pyridine rings of complexes **VI_1** and **VI_2**. S16

Discussion about alternative reaction mechanisms. S17

Figure S4. Gibbs energy profile of alternative reaction mechanisms found for the reactivity of *cis*,*trans*-[Re(bipy)$(CO)_2$(N-MeIm)(PMe_3)]OTf towards KN($SiMe_3$)$_2$ and MeOTf in dichloromethane solution. S18

Figure S5. Optimized structures of the species involved in the alternative reaction mechanisms found for the reaction of *cis,trans*-[Re(bipy)$(CO)_2$(*N*-MeIm)(PMe_3)]OTf with KN($SiMe_3$)$_2$ and MeOTf in dichloromethane solution. S20

Figure S6. Gibbs energy profile of the steps 1-3 of the reaction mechanism for the C-C coupling/methylation/deprotonation sequence of *cis*,*trans*-[Re$(CO)_2$(N-MeIm)(phen)(PMe_3)]OTf. S23

Figure S7. NICS(1) values for the imidazole, cyclopentadiene, and pyridine rings of complexes **VI_I$_p$** and **VI_2$_p$**. S24

Figure S8. Gibbs energy profile of the reactivity of *cis*,*trans*-[Re$(CO)_2$(N-MeIm)(phen)(PMe_3)]OTf towards KN($SiMe_3$)$_2$ and MeOTf to afford the protonated-N Re(I) products **VIII_1$_p$** and **VIII_2$_p$**. S25

Table S1. Electronic energy, enthalpy, entropy, and Gibbs energy of the species involved in the reaction of *cis,trans*-[Re(bipy)$(CO)_2$(*N*-MeIm)(PMe_3)]OTf with KN($SiMe_3$)$_2$ and MeOTf in dichloromethane solution. S26

Table S2. Relative electronic energy, enthalpy, entropy contribution, and Gibbs energy of the species involved in the reaction of *cis,trans*-[Re(bipy)$(CO)_2$(*N*-MeIm)(PMe_3)]OTf with KN($SiMe_3$)$_2$ and MeOTf in dichloromethane solution. S28

Table S3. Electronic energy, enthalpy, entropy, and Gibbs energy of the species involved in the reaction of *cis,trans*-[Re$(CO)_2$(*N*-MeIm)(phen)(PMe_3)]OTf with KN($SiMe_3$)$_2$ and MeOTf in dichloromethane solution. S30

Table S4. Relative electronic energy, enthalpy, entropy contribution, and Gibbs energy of the species involved in the reaction of *cis,trans*-[Re$(CO)_2$(*N*-MeIm)(phen)(PMe_3)]OTf with KN($SiMe_3$)$_2$ and MeOTf in dichloromethane solution. S32

Table S5. Electronic energy, enthalpy, entropy, and Gibbs energy of the species involved in the protonation step of the reaction of *cis,trans*-[Re$(CO)_2$(*N*-MeIm)(phen)(PMe_3)]OTf with KN($SiMe_3$)$_2$ and MeOTf in dichloromethane solution. S33

Table S6. Relative electronic energy, enthalpy, entropy, and Gibbs energy of the species involved in the protonation step of the reaction of *cis,trans*-[Re$(CO)_2$(*N*-MeIm)(phen)(PMe_3)]OTf with KN($SiMe_3$)$_2$ and MeOTf in dichloromethane solution. S33

Discussion about the effect of solvent on the C-C coupling step S34

Figure S9. Optimized structures of the species involved in the C-C coupling step for the reaction of the *cis,trans*-[Re$(CO)_2$(*N*-MeIm)(N-N)(PMe_3)]OTf (N-N = bipy, phen) compounds with KN($SiMe_3$)$_2$ and MeOTf in tetrahydrofuran solution S35

Figure S10. Optimized structures of the species involved in the C-C coupling step for the reaction of the *cis,trans*-[Re$(CO)_2$(*N*-MeIm)(N-N)(PMe_3)]OTf (N-N = bipy, phen) compounds with KN($SiMe_3$)$_2$ and MeOTf in dichloromethane solution S36

Table S7. Electronic energy, enthalpy, entropy, and Gibbs energy of the species involved in the C-C coupling step for the reaction of the *cis,trans*-[Re$(CO)_2$(N-N)(*N*-MeIm)(PMe_3)]OTf (N-N = bipy, phen) compounds with KN($SiMe_3$)$_2$ and MeOTf in tetrahydrofuran solution. S37

Table S8. Relative electronic energy, enthalpy, entropy, and Gibbs energy of the species involved in the C-C coupling step for the reaction of the *cis,trans*-[Re(CO)$_2$(N-N)(*N*-MeIm)(PMe$_3$)]OTf (N-N = bipy, phen) compounds with KN(SiMe$_3$)$_2$ and MeOTf both in tetrahydrofuran solution and dichloromethane solution. S37
Figure S11. Bond lengths and net natural atomic charges of the non-hydrogen atoms of the dearomatized pyridine ring of the bipy ligand in complexes **III** and **IV** together with their Lewis structures. S38
Table S9. Cartesian coordinates of the species involved in the reaction of *cis,trans*-[Re(bipy)(CO)$_2$(*N*-MeIm)(PMe$_3$)]OTf with KN(SiMe$_3$)$_2$ and MeOTf in dichloromethane solution. S39
Table S10. Cartesian coordinates of the species involved in the reaction of *cis,trans*-[Re(CO)$_2$(*N*-MeIm)(phen)(PMe$_3$)]OTf with KN(SiMe$_3$)$_2$ and MeOTf in dichloromethane solution. S56

Experimental Section **S70**
General Details S70
Reaction of *cis,trans*-[Re(bipy)(CO)$_2$(N-MesIm)(PMe$_3$)]OTf (**1b**) with KN(SiMe$_3$)$_2$. Synthesis of compound **2b**. S70
Reaction of *cis,trans*-[Re(CO)$_2$(N-MeIm)(phen)(PMe$_3$)]OTf (**1c**) with KN(SiMe$_3$)$_2$. Synthesis of compound **2c**. S70
Reaction of *cis,trans*-[Re(CO)$_2$(N-MesIm)(phen)(PMe$_3$)]OTf (**1d**) with KN(SiMe$_3$)$_2$. Synthesis of compound **2d**. S71
Reaction of *cis,trans*-[Re(bipy)(CO)$_2$(N-MesIm)(PMe$_3$)]OTf (**1b**) with KN(SiMe$_3$)$_2$ and MeOTf. Synthesis of compound **3b**. S71
Reaction of *cis,trans*-[Re(CO)$_2$(N-MeIm)(phen)(PMe$_3$)]OTf (**1c**) with KN(SiMe$_3$)$_2$ and MeOTf. Synthesis of **3c** and **3c'**. S71
Reaction of *cis,trans*-[Re(CO)$_2$(N-MesIm)(phen)(PMe$_3$)]OTf (**1d**) with KN(SiMe$_3$)$_2$ and MeOTf. Synthesis of **3d**. S72
Reaction of *cis,trans*-[Re(CO)$_2$(N-MeIm)(phen)(PMe$_3$)]OTf (**1c**) with KN(SiMe$_3$)$_2$ and HOTf. Synthesis of compound **9c**. S72
Reaction of *cis,trans*-[Re(CO)$_2$(N-MesIm)(phen)(PMe$_3$)]OTf (**1d**) with KN(SiMe$_3$)$_2$ and HOTf. Synthesis of compound **9d**. S72
Crystal Structure Determination Details (X-ray structures of 2b, 3c'and 9d). General description. S74
References **S74**
NMR spectra
Figure S12. ^{1}H NMR spectrum of compound **2b** in CD$_2$Cl$_2$ at 233 K. S77
Figure S13. ^{13}C {^{1}H} NMR spectrum of compound **2b** in CD$_2$Cl$_2$ at 233 K. S78
Figure S14. ^{31}P {^{1}H} NMR spectrum of compound **2b** in CD$_2$Cl$_2$. S79
Figure S15. ^{1}H-^{1}H COSY NMR spectrum of compound **2b** in CD$_2$Cl$_2$ at 233 K. S80
Figure S16. ^{1}H-^{13}C HSQC NMR spectrum of compound **2b** in CD$_2$Cl$_2$ at 233 K. S81
Figure S17. ^{1}H NMR spectrum of compound **2c** in CD$_2$Cl$_2$. S82
Figure S18. ^{13}C {^{1}H} NMR spectrum of compound **2c** in CD$_2$Cl$_2$ at 213 K. S83
Figure S19. ^{31}P{^{1}H} NMR spectrum of compound **2c** in CD$_2$Cl$_2$. S84
Figure S20. ^{1}H-^{1}H COSY NMR spectrum of compound **2c** in CD$_2$Cl$_2$ at 213 K. S85
Figure S21. ^{1}H-^{13}C HSQC NMR spectrum of compound **2c** in CD$_2$Cl$_2$ at 213 K. S86
Figure S22. ^{1}H-^{13}C HMBC NMR spectrum of compound **2c** in CD$_2$Cl$_2$ at 213 K. S87
Figure S23. ^{1}H NMR spectrum of compound **2d** in CD$_2$Cl$_2$. S88
Figure S24. ^{13}C {^{1}H} NMR spectrum of compound **2d** in CD$_2$Cl$_2$. S89
Figure S25. ^{31}P {^{1}H} NMR spectrum of compound **2d** in CD$_2$Cl$_2$. S90
Figure S26. ^{1}H-^{1}H COSY NMR spectrum of compound **2d** in CD$_2$Cl$_2$. S91
Figure S27. ^{1}H-^{13}C HSQC NMR spectrum of compound **2d** in CD$_2$Cl$_2$. S92
Figure S28. ^{1}H-^{13}C HMBC NMR spectrum of compound **2d** in CD$_2$Cl$_2$. S93
Figure S29. ^{1}H NMR spectrum of compound **3b** in CD$_2$Cl$_2$. S94
Figure S30. ^{13}C {^{1}H} NMR spectrum of compound **3b** in CD$_2$Cl$_2$. S95
Figure S31. ^{31}P {^{1}H} NMR spectrum of compound **3b** in CD$_2$Cl$_2$. S96
Figure S32. ^{1}H-^{1}H COSY NMR spectrum of compound **3b** in CD$_2$Cl$_2$. S97

Figure S33. ^{1}H-^{13}C HSQC NMR spectrum of compound **3b** in CD_2Cl_2. S98
Figure S34. ^{1}H NMR spectrum of compound **3c** in CD_2Cl_2. S99
Figure S35. ^{13}C {^{1}H} NMR spectrum of compound **3c** in CD_2Cl_2. S100
Figure S36. ^{31}P {^{1}H} NMR spectrum of compound **3c** in CD_2Cl_2. S101
Figure S37. ^{1}H-^{1}H COSY NMR spectrum of compound **3c** in CD_2Cl_2. S102
Figure S38. ^{1}H-^{13}C HSQC NMR spectrum of compound **3c** in CD_2Cl_2. S103
Figure S39. ^{1}H NMR spectrum of compound **3c'** in CD_2Cl_2. S104
Figure S40. ^{13}C {^{1}H} NMR spectrum of compound **3c'** in CD_2Cl_2. S105
Figure S41. ^{31}P {^{1}H} NMR spectrum of compound **3c'** in CD_2Cl_2. S106
Figure S42. ^{1}H-^{1}H COSY NMR spectrum of compound **3c'** in CD_2Cl_2. S107
Figure S43. ^{1}H-^{13}C HSQC NMR spectrum of compound **3c'** in CD_2Cl_2. S108
Figure S44. ^{1}H-^{13}C HMBC NMR spectrum of compound **3c'** in CD_2Cl_2. S109
Figure S45. ^{1}H NMR spectrum of compound **3d** in CD_2Cl_2. S110
Figure S46. ^{13}C {^{1}H} NMR spectrum of compound **3d** in CD_2Cl_2. S111
Figure S47. ^{31}P {^{1}H} NMR spectrum of compound **3d** in CD_2Cl_2. S112
Figure S48. ^{1}H-^{1}H COSY NMR spectrum of compound **3d** in CD_2Cl_2. S113
Figure S49. ^{1}H-^{13}C HSQC NMR spectrum of compound **3d** in CD_2Cl_2. S114
Figure S50. ^{1}H-^{13}C HMBC NMR spectrum of compound **3d** in CD_2Cl_2. S115
Figure S51. ^{1}H NMR spectrum of compound **9c** in CD_2Cl_2. S116
Figure S52. ^{13}C {^{1}H} NMR spectrum of compound **9c** in CD_2Cl_2. S117
Figure S53. ^{31}P {^{1}H} NMR spectrum of compound **9c** in CD_2Cl_2. S118
Figure S54. ^{1}H-^{1}H COSY NMR spectrum of compound **9c** in CD_2Cl_2. S119
Figure S55. ^{1}H-^{13}C HSQC NMR spectrum of compound **9c** in CD_2Cl_2. S120
Figure S56. ^{1}H NMR spectrum of compound **9d** in CD_2Cl_2. S121
Figure S57. ^{13}C {^{1}H} NMR spectrum of compound **9d** in CD_2Cl_2. S122
Figure S58. ^{31}P {^{1}H} NMR spectrum of compound **9d** in CD_2Cl_2. S123
Figure S59. ^{1}H-^{1}H COSY NMR spectrum of compound **9d** in CD_2Cl_2. S124
Figure S60. ^{1}H-^{13}C HSQC NMR spectrum of compound **9d** in CD_2Cl_2. S125

Computational Details

1. Methods and technical details

All species involved in the reaction of *cis*,*trans*-[Re(CO)$_2$(N-N)(N-RIm)(PMe$_3$)](OTf) (N-N = 2,2'-bipyridine (bipy), 1,10-phenanthroline (phen); N-RIm = N-methylimidazole (N-MeIm)) compounds with KN(SiMe$_3$)$_2$, followed by the addition of an excess of methyl triflate (MeOTf), were optimized in CH_2Cl_2 solution from the outset with the Conductor-like Polarizable Continuum Model[1,2] (CPCM) and the Universal Force Field[3] (UFF) radii in conjunction with the hybrid density functional B3LYP[4-6] and the 6-31+G(d) basis set for nonmetal atoms[7] together with the valence double-ζ basis set LANL2DZ plus the effective core potential of Hay and Wadt for the Re atom,[8] and by using a modified Schlegel algorithm.[9-11] The C-C coupling step in the bipy case was also investigated within a tetrahydrofuran (THF) solution instead of CH_2Cl_2 to check the influence of these solvents on the energetics of this part of the reaction mechanism. Electrostatic, cavitation, dispersion, and repulsion terms[12] were considered in the CPCM computations, wherein relative permittivities of 8.93 and 7.4257 were assumed to simulate CH_2Cl_2 and THF as the solvents experimentally used, respectively. The nature of the stationary points found, minima (reactants, products or intermediates) and first-order saddle points (TS), was confirmed by analytical computations of harmonic vibrational frequencies. Starting from the optimized geometry obtained for each localized TS, intrinsic reaction coordinate (IRC) calculations were performed using the Gonzalez and Schlegel method to determine the two minimum-energy structures that are connected to each TS.[13,14] Gibbs free energies in CH_2Cl_2 solution (ΔG) were also calculated within the ideal gas, rigid rotor, and harmonic oscillator approximations at a pressure of 1 atm and a temperature of 298.15 K.[15] The calculation of thermodynamic magnitudes in solution starting with molecular partition functions developed for computing gas-phase thermodynamics properties is a standard procedure that has proven to be a correct and useful approach.[16] For interpretation purposes a topological analysis of the electron density based on the Atoms-in-Molecules (AIM) theory of Bader[17-19] in conjunction with a natural bond order (NBO)[20,21] analysis were performed. Besides, using the Gauge-independent atomic orbital (GIAO) method[22-26] at the B3LYP/6-311+G(d,p)-LANL2DZ level using the CPCM-B3LYP/6-31+G(d)-LANL2DZ optimized geometries, nucleus-independent chemical shifts (NICS)[27,28] were calculated at the ring critical point (RCP) of electron density located 1.0 Å above these RCP, NICS(1), as aromaticity indices. NICS(1) were recommended as an adequate measure of the π-electron delocalization.[29-32]

The computational protocol used in this work is equal or similar to those employed in related investigations on carbonyl Re(I) complexes.[33-37] All the quantum chemical calculations were performed with the Gaussian 09 (G09) suite of programs.[38]

2. Discussion about the overestimation of the Gibbs energy barrier in solution for the deprotonation step

The relatively large Gibbs energy barrier in solution of the deprotonation step in Figure 1 (transformation **III** → **TSIII** → **IV**) is due to an overstabilization of intermediate **III** as a consequence of not considering it interacting with other involved species such as OTf^- and HN(SiMe$_3$)$_2$. To get an idea about this, we compared **III** with intermediates **III'**, in which the metal fragment is interacting with OTf^-, and **III''**, in which the metal fragment is now interacting with HN(SiMe$_3$)$_2$. The transformation **III'** → **III** + OTf^- (molecularity change = 1) stabilizes the system at 7.4 kcal/mol, while a value of 17.1 kcal/mol would correspond to the transformation **III''** → **III** + HN(SiMe$_3$)$_2$ (molecularity change = 1). So, **III** could have been overstabilized between 7.4 and 17.1 kcal/mol, and consequently the actual Gibbs energy barrier of **TSIII** referred to **III** would be lower than that shown in Figure 1

(probably between 38.0 and 27.3 kcal/mol). Similar reasoning can be applied to the **TSIIIp** barrier referred to **IIIp** in Figure S6.

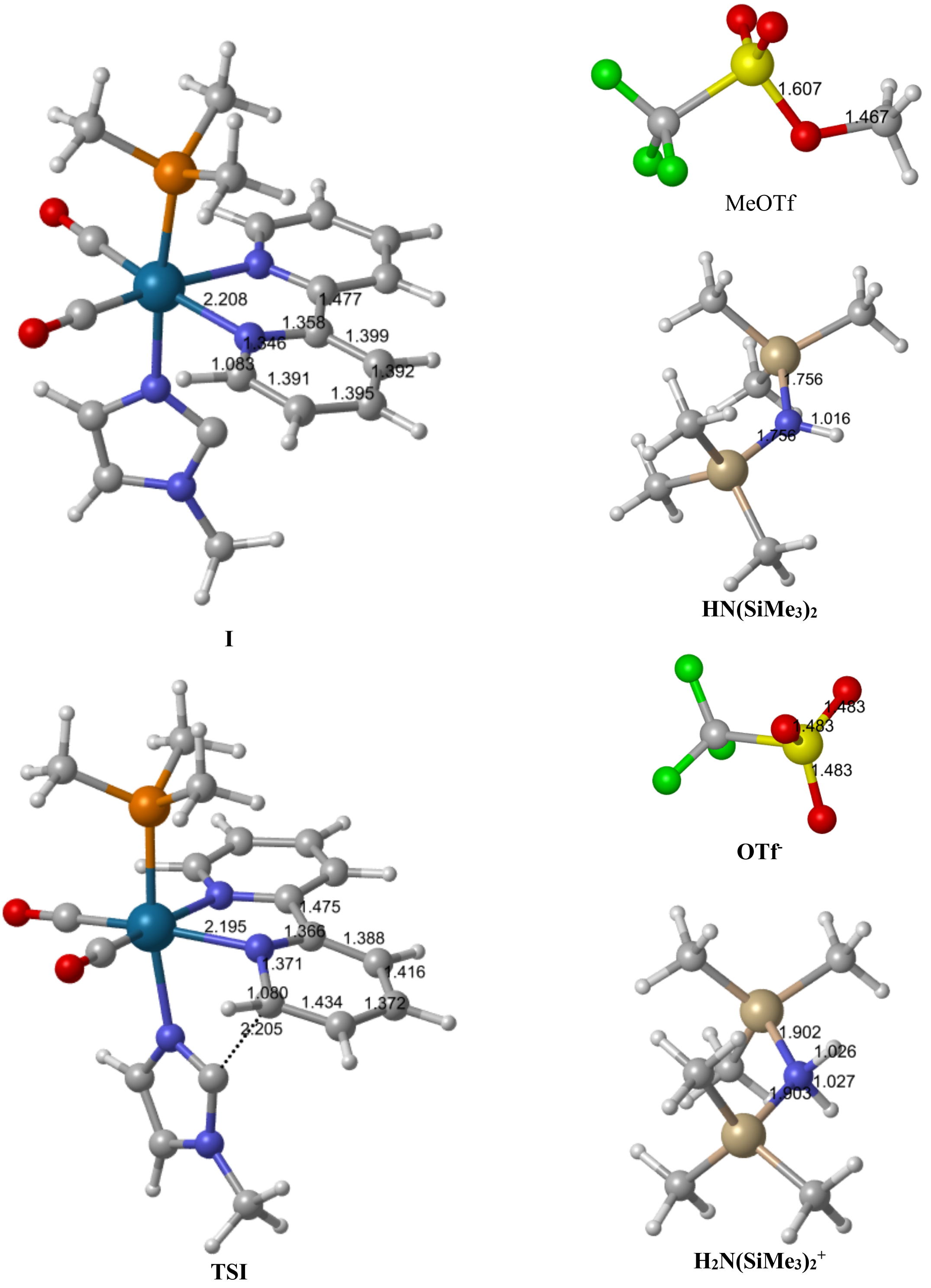

Figure S1. CPCM-B3LYP/6-31+G(d)-LANL2DZ optimized structures of the species involved in the reaction of the *cis,trans*-[Re(bipy)$(CO)_2$(*N*-MeIm)(PMe_3)]OTf compound with $KN(SiMe_3)_2$ and MeOTf in dichloromethane solution. Some relevant bond distances are included in angstroms.

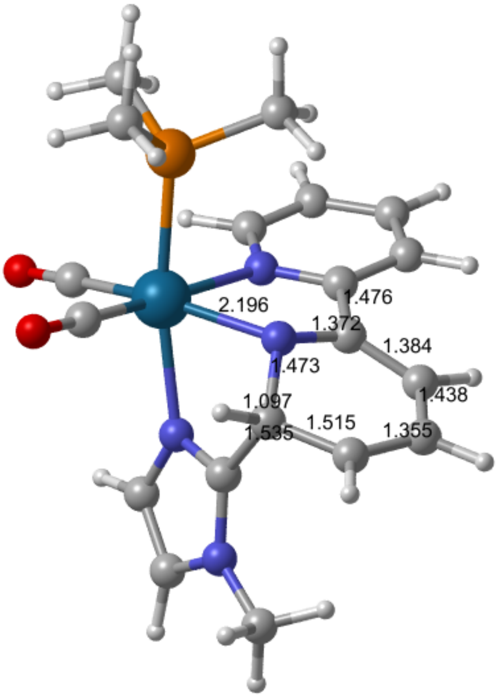

II

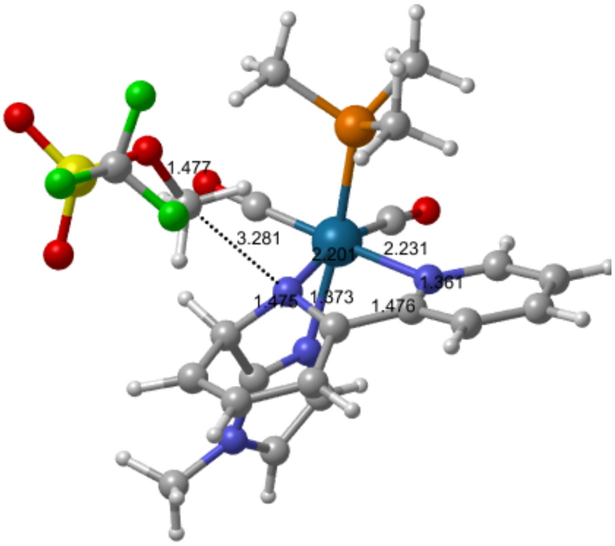

II’

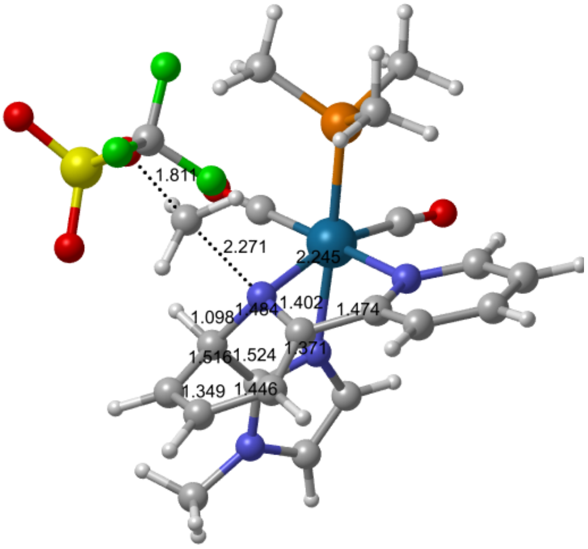

TSII

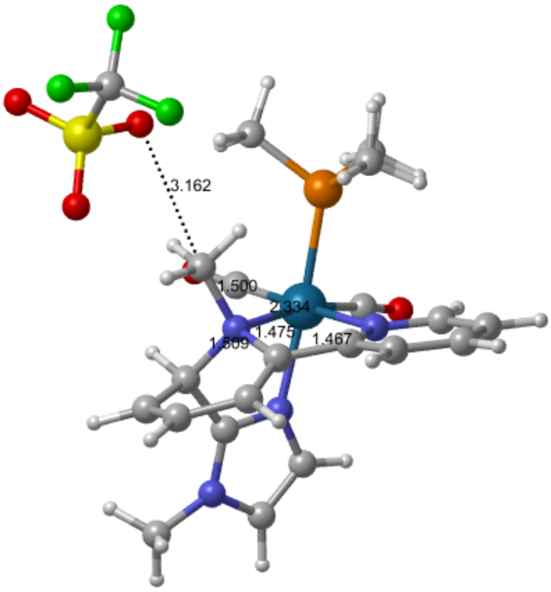

III’

Figure S1. *(Cont.)*

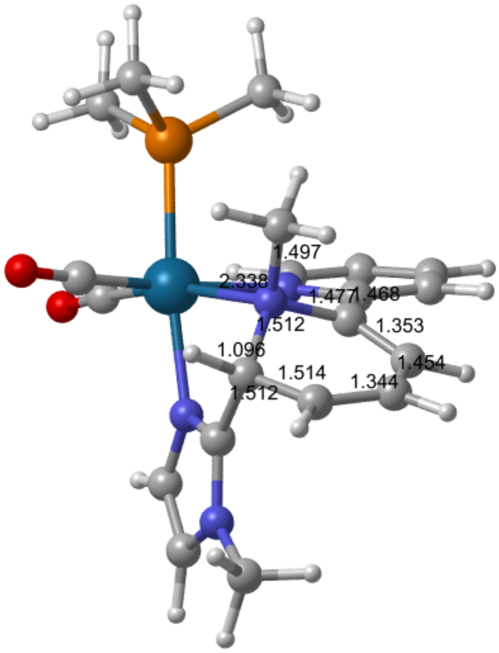

III

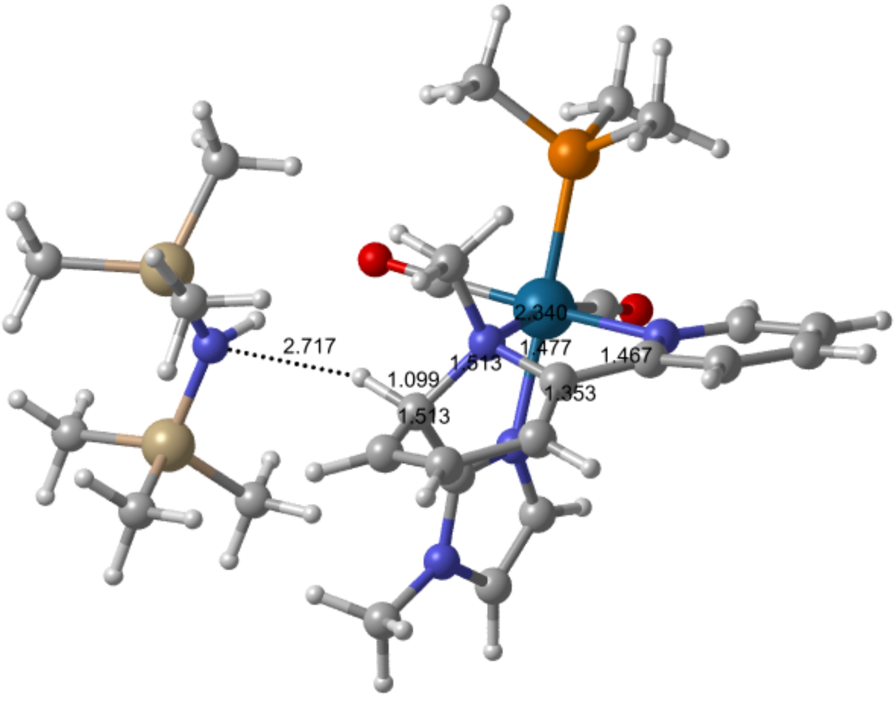

III''

Figure S1. *(Cont.)*

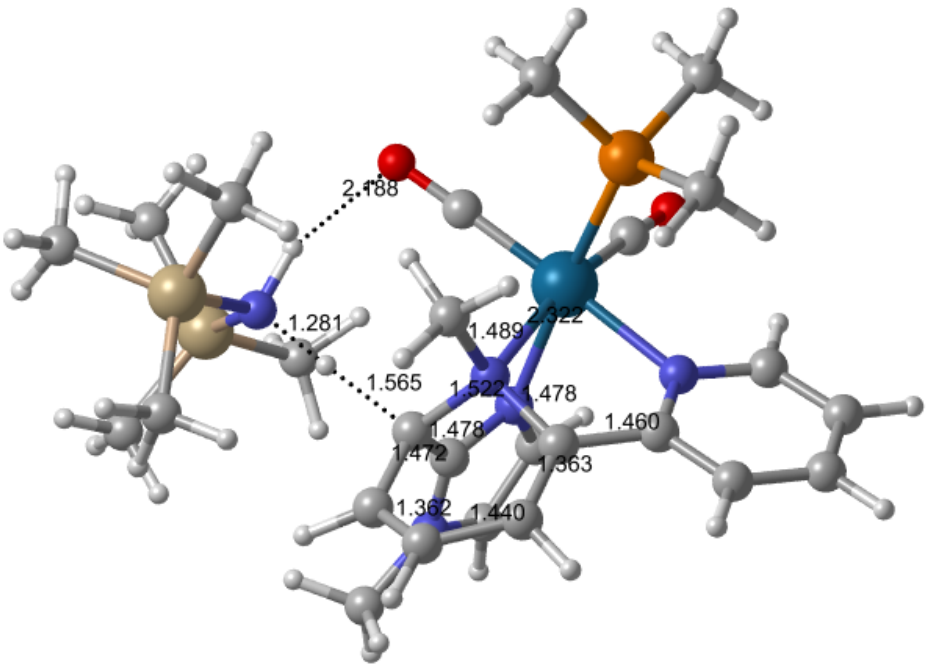

TSIII

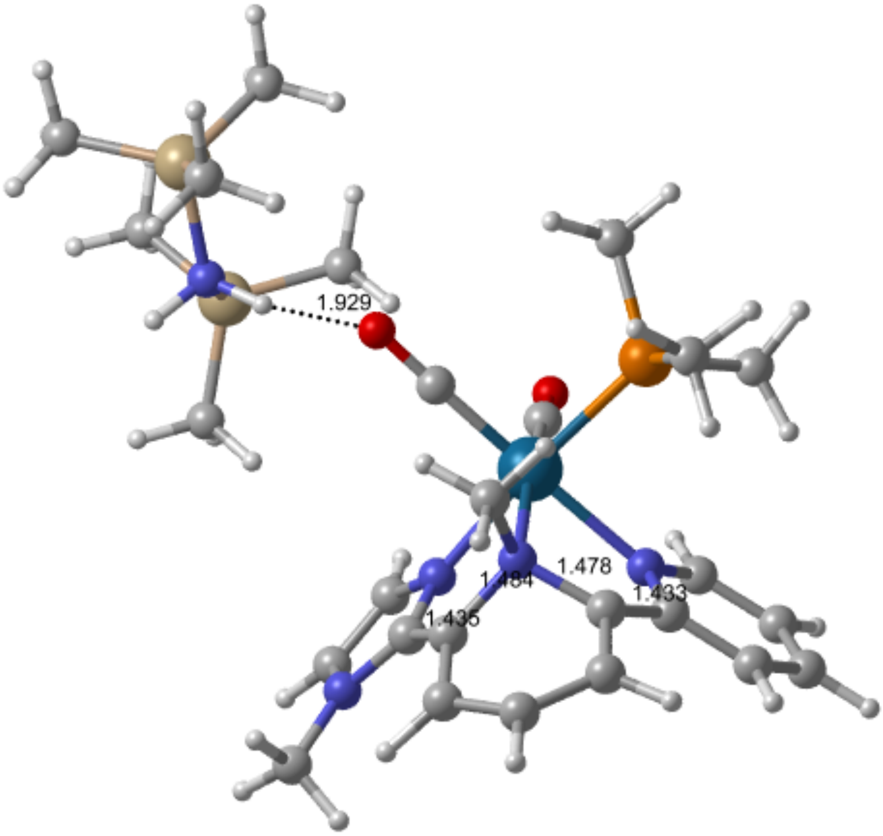

IV'

Figure S1. *(Cont.)*

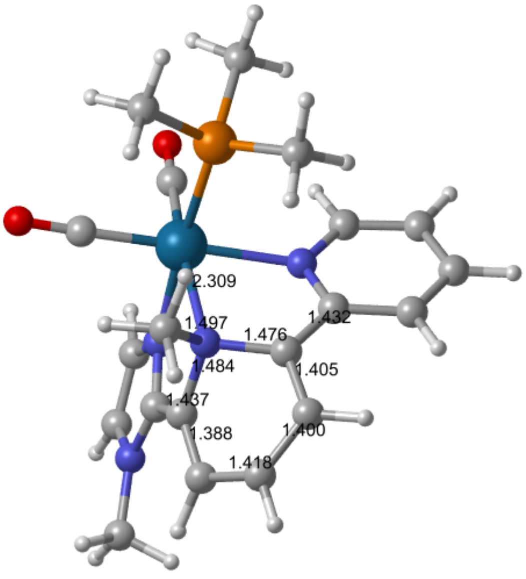

IV

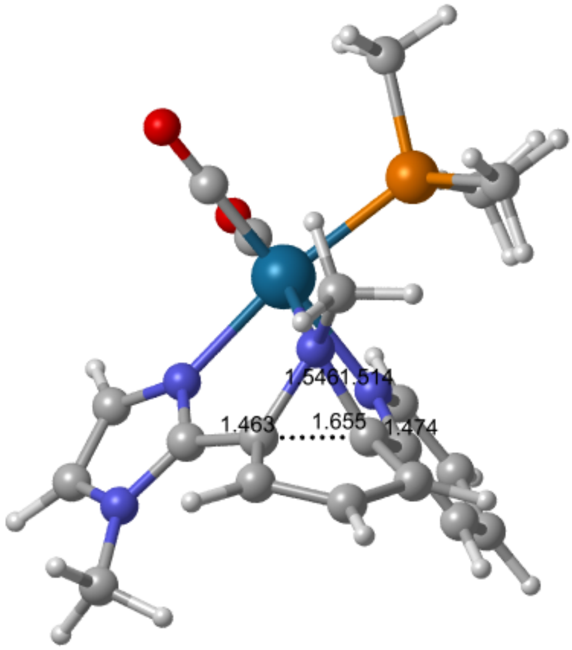

TSIV

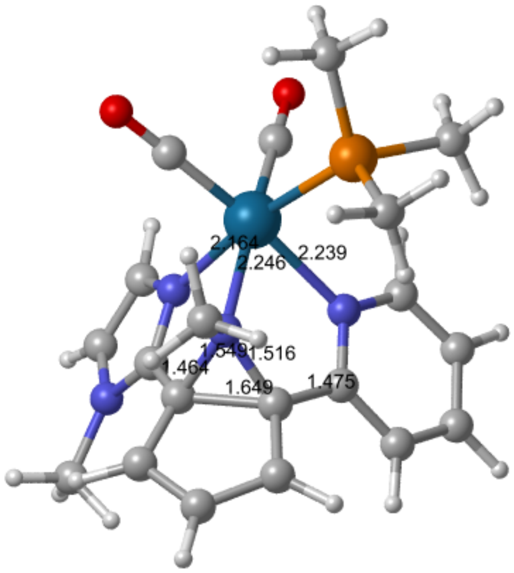

V

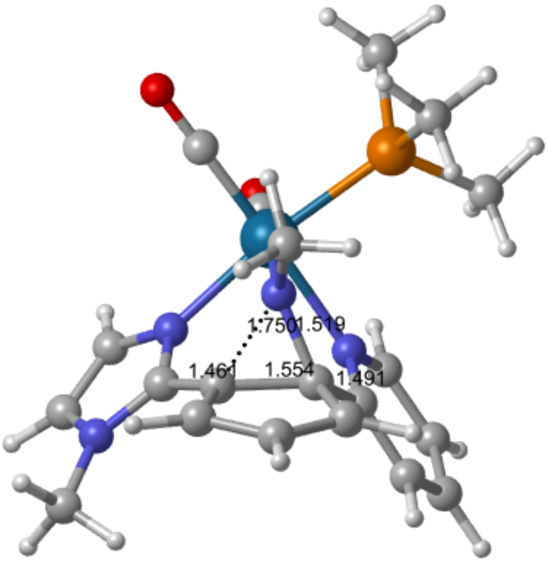

TSV_1

Figure S1. *(Cont.)*

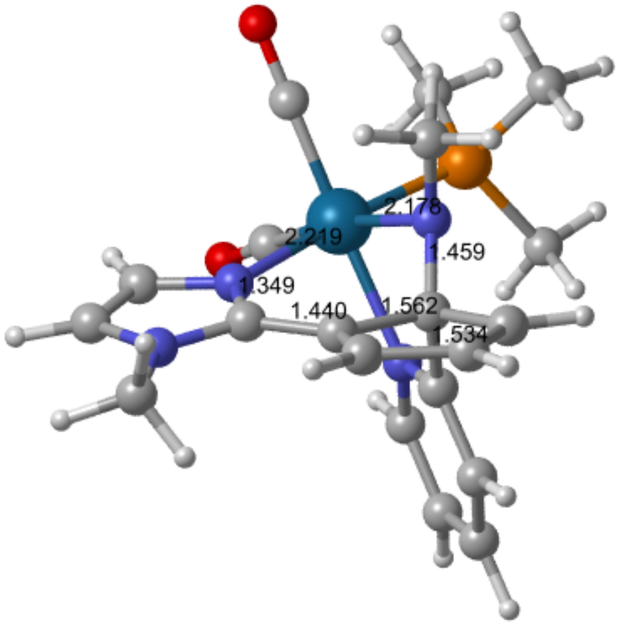

VI_1

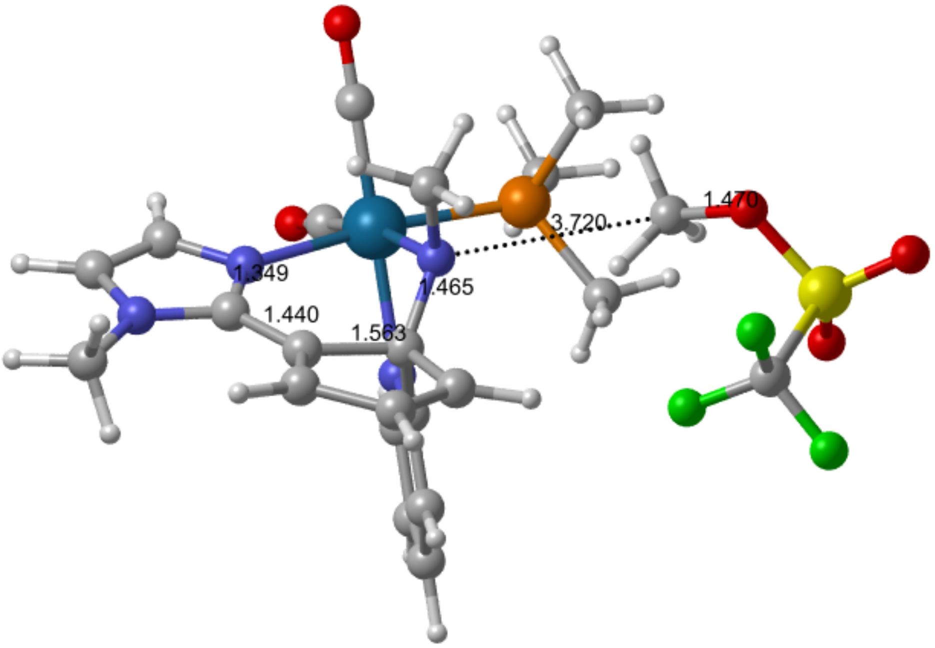

VI'_1

Figure S1. *(Cont.)*

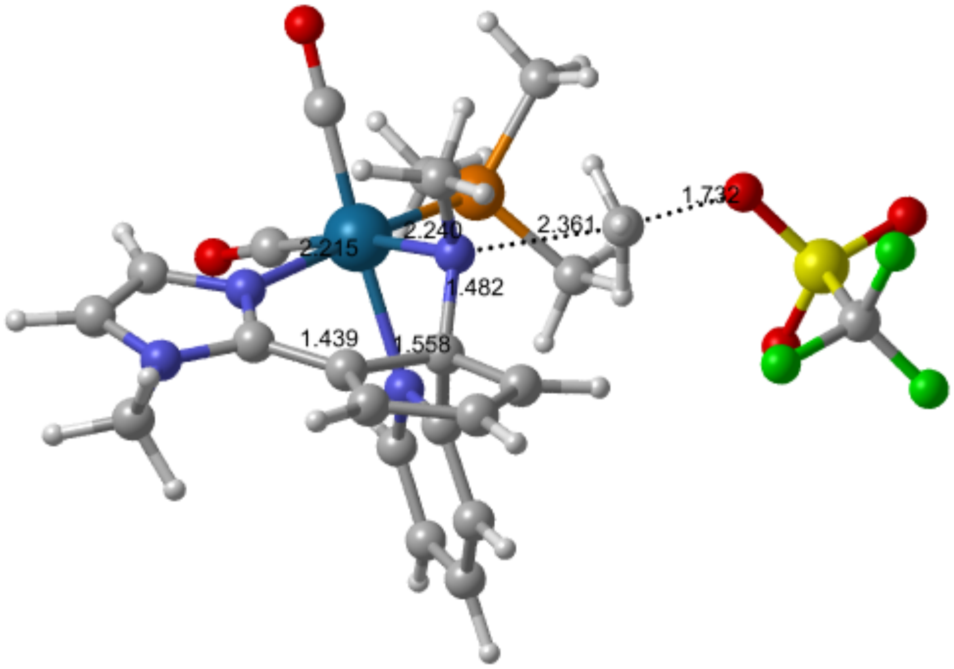

TSVI_1

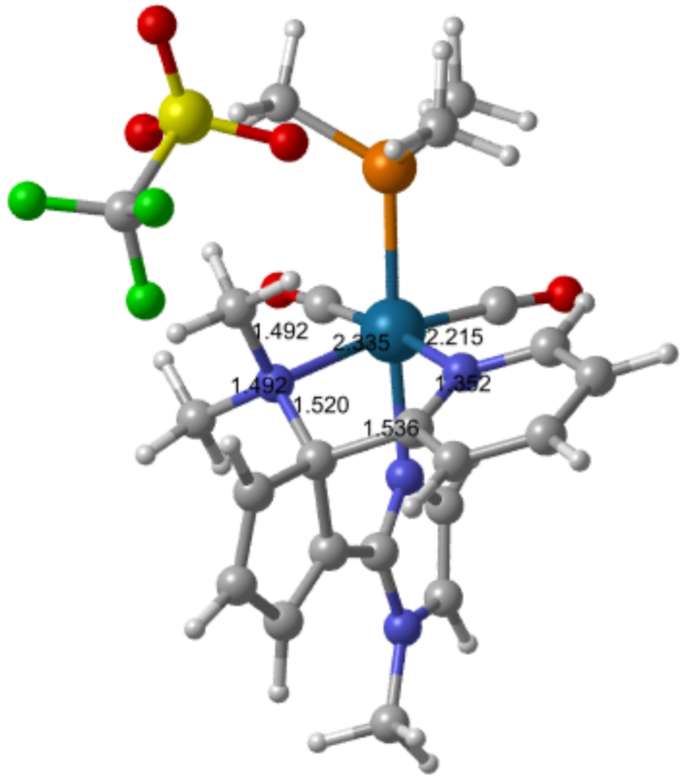

VII'_1

Figure S1. *(Cont.)*

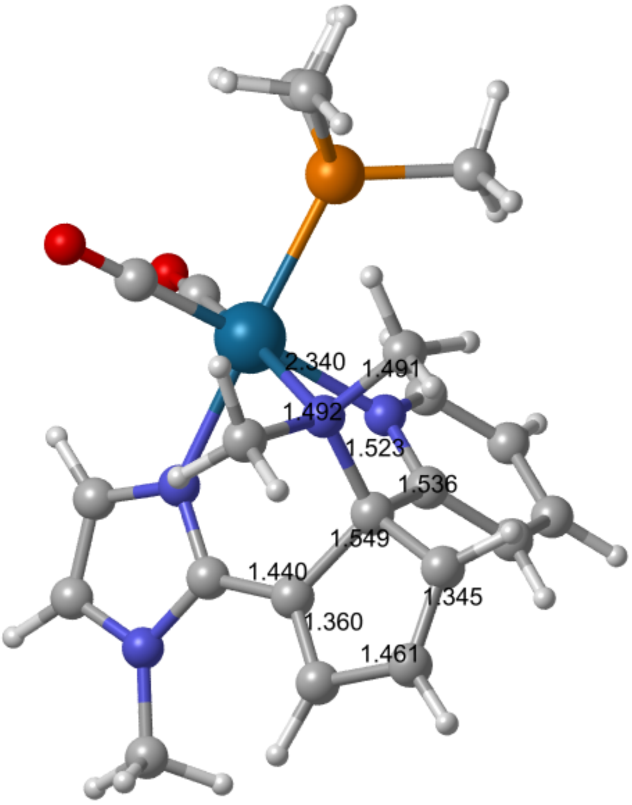

VII_1

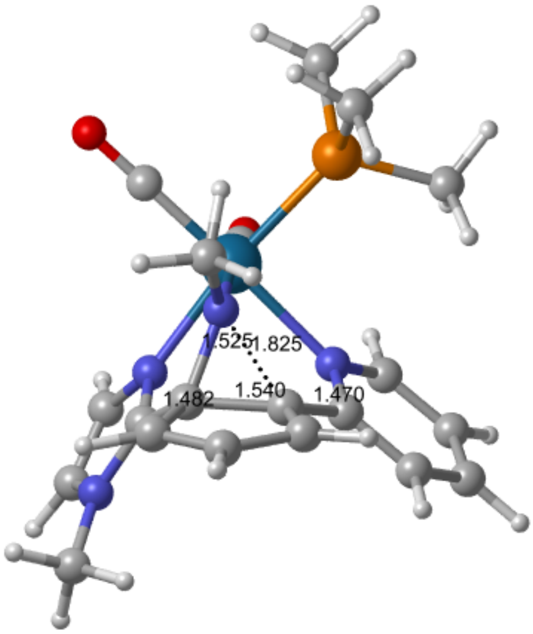

TSV_2

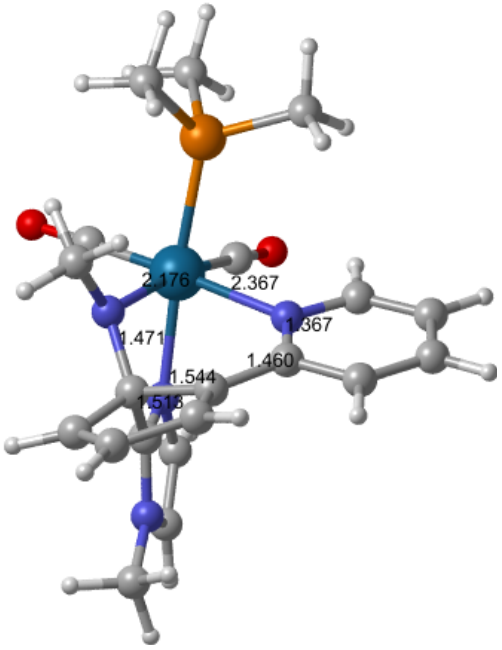

VI_2

Figure S1. *(Cont.)*

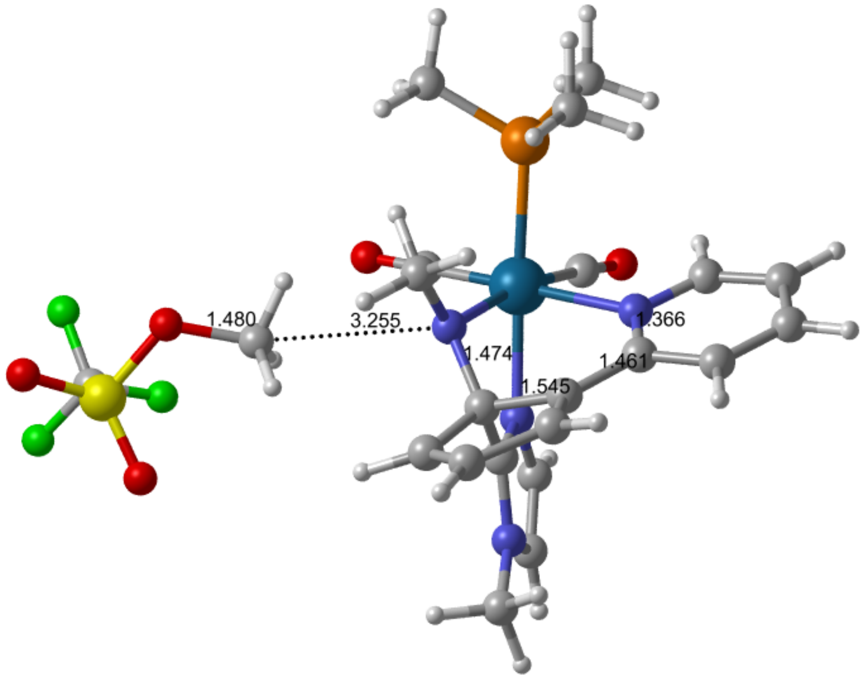

VI'_2

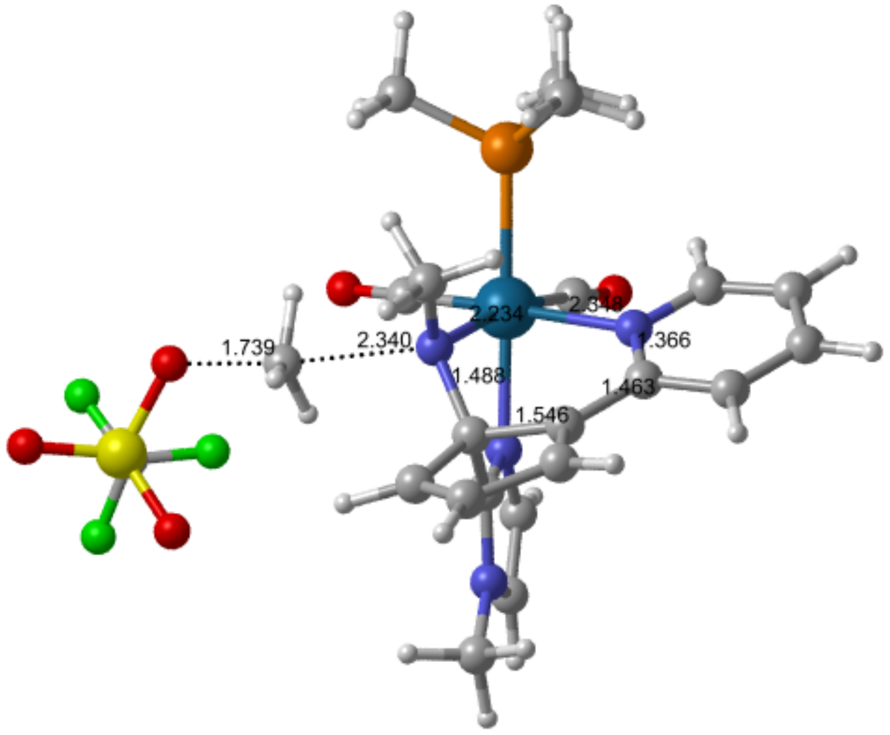

TSVI_2

Figure S1. *(Cont.)*

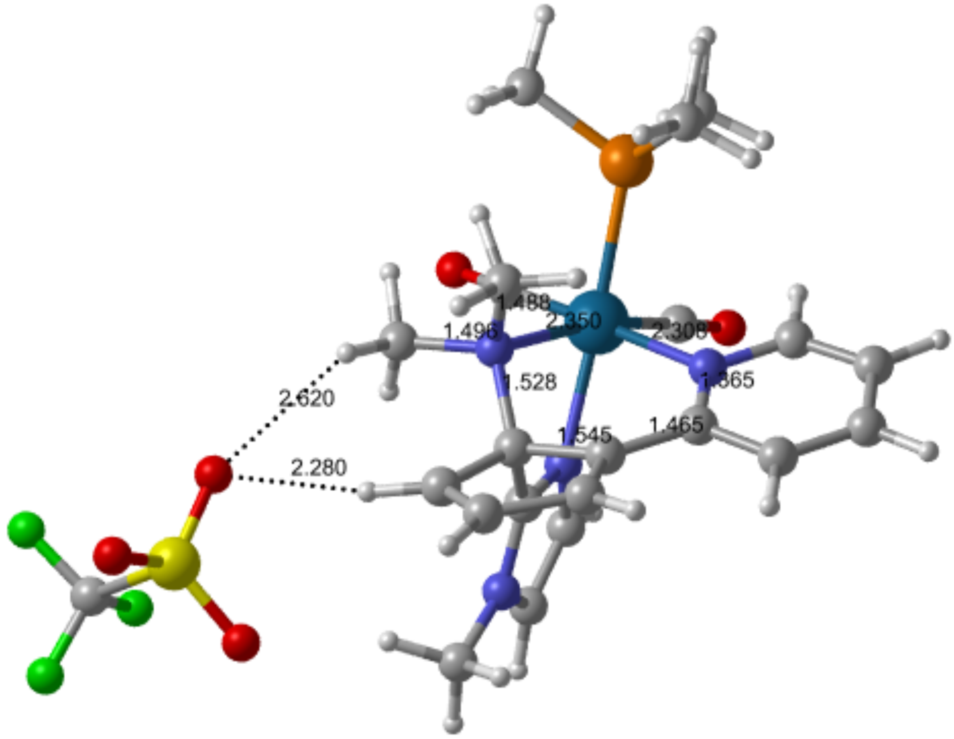

VII'_2

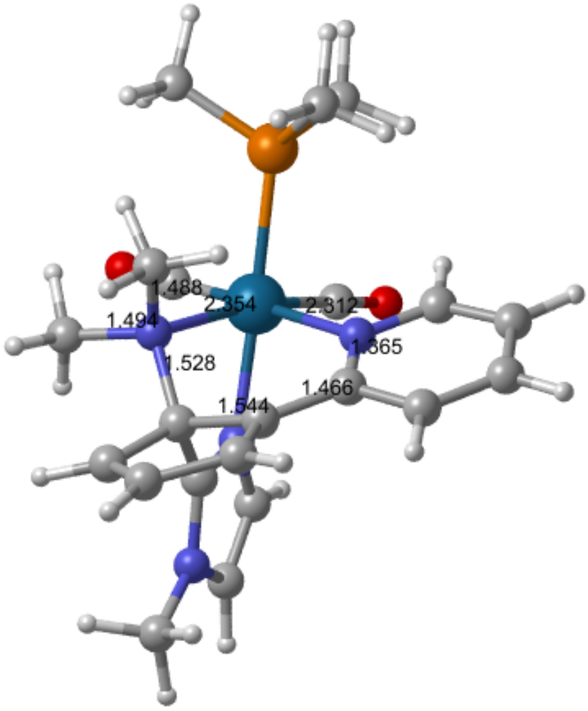

VII_2

Figure S1. *(Cont.)*

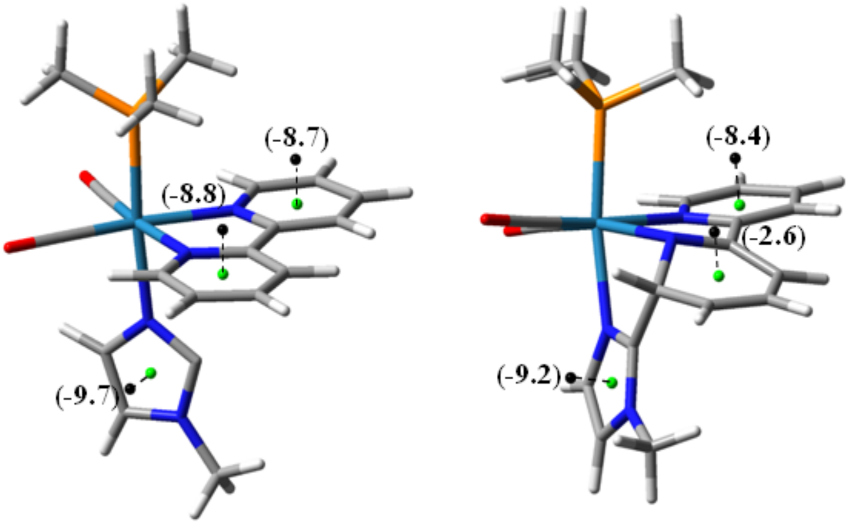

I **II**

Figure S2. Nucleus-independent chemical shifts (black dots: in parenthesis in ppm) obtained at 1 Å (NICS(1)) above the ring critical points (RCP, green dots) of electron density located on the imidazole and pyridine rings of complexes **I** and **II**, involved in the dearomatization of one bipy pyridine ring, at the B3LYP/6-311+G(d,p)-LANL2DZ level of theory using the corresponding CPCM-B3LYP/6-31+G(d)-LANL2DZ optimized geometries.

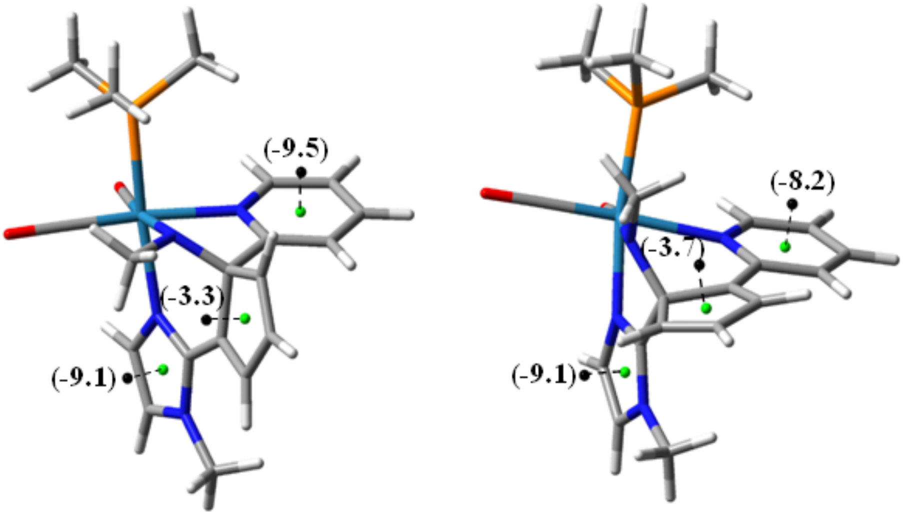

VI_1 **VI_2**

Figure S3. Nucleus-independent chemical shifts (black dots: in parenthesis in ppm) obtained at 1 Å (NICS(1)) above the ring critical points (RCP, green dots) of electron density located on the imidazole, cyclopentadiene, and pyridine rings of complexes **VI_1** and **VI_2** at the B3LYP/6-311+G(d,p)-LANL2DZ level of theory using the corresponding CPCM-B3LYP/6-31+G(d)-LANL2DZ optimized geometries.

Discussion about alternative reaction mechanisms

Reaction mechanisms other than the one described in the manuscript (Scheme 2 and Figures 1 and 2) were found for the reaction of the *cis,trans*-[Re(bipy)$(CO)_2$(N-MeIm)(PMe_3)]OTf compound with $KN(SiMe_3)_2$ and MeOTf to give the Re(I) complex featuring a pyridine ring-opening whose nitrogen atom is dimethylated and bonded to the cyclopentadiene carbon attached to the intact pyridine ring of the original bipy ligand (**VII_1** in Figure S4). All the alternative reaction mechanisms ~~found~~ have in common the first three steps (C-C coupling between the imidazole ring and one of the bipy pyridine rings, methylation of the nitrogen atom of the pyridine ring attached to the imidazole one, and deprotonation of the methylated pyridine ring), which are the same as those found along the most favored reaction mechanism described in the manuscript. Therefore, here we will only discuss the steps of the alternative reaction mechanisms starting from intermediate **IV** (see Figures S4 and S5, and Tables S1 and S2). On the one hand, as seen in Figure S4a, this intermediate can undergo the second methylation at the C6 carbon through the transition state (TS) **TSIV_alt1**, 32.8 kcal/mol less stable than **IV**, to give rise, after the release of the triflate ion, to the very stable intermediate **V_alt1**, 36.1 kcal/mol more stable than **IV**. In **TSIV_alt1** the distance between the MeOTf methyl carbon (C_{methyl}) and the C6 carbon is 2.427 Å, while in **V_alt1** a value of 1.542 Å was obtained consistent with the newly formed C_{methyl}-C6 bond. The next step is the cleavage of the bond between the methylated nitrogen ($N_{methylated}$) and the methylated C6 carbon, which involves overcoming a large energy barrier of 40.2 kcal/mol (determined by the TS **TSV_alt1**), in good agreement with the well-known strength of the pyridine C-N bond (binding energy about 133 kcal/mol). The C6-$N_{methylated}$ bond is already practically broken in **TSV_alt1**, distance of 2.594 Å, so that the intermediate to which this TS evolves, **VI_alt1**, has completely open the dimethylated pyridine ring. This intermediate is, however, 1.1 kcal/mol more stable than **V_alt1**. Then, the system evolves via the TS **TSVI_alt1**, 62.2 kcal/mol higher in energy than **VI_alt1**, to the intermediate **VII_alt1**, 57.5 kcal/mol higher in energy than **VI_alt1**, in which a cyclopentadiene ring has been established due to the formation of a new C6-C2 bond at a distance of 1.609 Å. **VII_alt1** undergoes the methyl migration from the C6 carbon to $N_{methylated}$ to finally give the dimethylated product **VII_1**, only 3.4 kcal/mol less stable than **VI_alt1**, through the TS **TSVII_alt1** with a very high energy barrier of 79.3 kcal/mol measured from **VI_alt1**. Therefore, this alternative route leading to **VII_1** comprises of the following steps: (1) C-C coupling between the imidazole ring and one phen bipy ring, (2) first methylation of the bipy pyridine ring attached to the imidazole ring, (3) deprotonation of the methylated pyridine ring, (4) methylation of the C6 carbon of the methylated pyridine ring, (5) cleavage of the C6-$N_{methylated}$ bond, (6) formation of the C6-C2 bond, and (7) 1,3-methyl shift from C6 to $N_{methylated}$. The last step shows the rate-determining energy barrier of this mechanistic route with a value of 79.3 kcal/mol, which would prevent obtaining the product found experimentally.

On the other hand, as seen in Figure S4b, intermediate **IV** can proceed via the second methylation at the C5 carbon of the methylated pyridine ring through the TS **TSIV_alt2**, 44.7 kcal/mol less stable than **IV**, to render, after the release of the triflate ion, the very stable intermediate **V_alt2**, 30.4 kcal/mol more stable than **IV**. The distance between the MeOTf methyl carbon and C5 is 2.520 Å at **TSIV_alt2** (1.538 Å at **V_alt2**), while a value of 1.805 Å was obtained between the former carbon and the MeOTf oxygen to which it was initially attached. It is interesting to note here that **TSIV_alt2** and **V_alt2** have a bicyclic moiety consisting of a pentacarbon ring (defined by the C2, C3, C4, C5, and C6 atoms) fused to another three-membered ring (defined by the C2, $N_{methylated}$, and C6 atoms). That is, the methylated pyridine ring has become a bicyclic moiety, in which the C2···C6 distance is 1.632 and 1.541 Å at **TSIV_alt2** and **V_alt2**, respectively. The next step involves the 1,2-methyl shift from C5 to C6 along with the scission of the C6-$N_{methylated}$ bond through the TS **TSV_alt2**, which has a high energy barrier of 62.8 kcal/mol measured from **V_alt2**. At this TS the methyl carbon initially attached to C5 is 1.914 and 1.808 Å from C5 and C6, respectively, while the C6-$N_{methylated}$ bond is clearly broken as it presents a distance of 2.480 Å. **TSV_alt2** leads to **VII_alt1**, which in turn evolves to **VII_1** via **TSVII_alt1** as explained above.

The two routes just described are also connected through the TS **TSV_alt1_V_alt2** (Figure S5) connecting **V_alt1** with **V_alt2**, which is 60.0 and 54.3 kcal/mol more stable than these intermediates, respectively. **TSV_alt1_alt2** involves a 1,2-methyl shift between C5 and C6 (d(C_{methyl}···C5) = 1.938 Å, d(C_{methyl}···C5) = 2.057 Å) as **TSV_alt2**, but now this rearrangement is connected with the formation of the C6-C2 bond (d(C6···C2) = 2.236 Å) instead of the rupture of the C6-$N_{methylated}$ one. In any case, all these routes evolve through energy barriers higher than 70 kcal/mol, which would not justify the experimentally detected product (**VII_1**). A similar conclusion would be expected if these mechanistic pathways for the formation of the **VII_2** product were analyzed. Similar structures have been found for the reaction of *cis,trans*-$[ReCO)_2$(N-MeIm) (phen)(PMe_3)]OTf but the high energetic profile found preclude a deep study.

a)

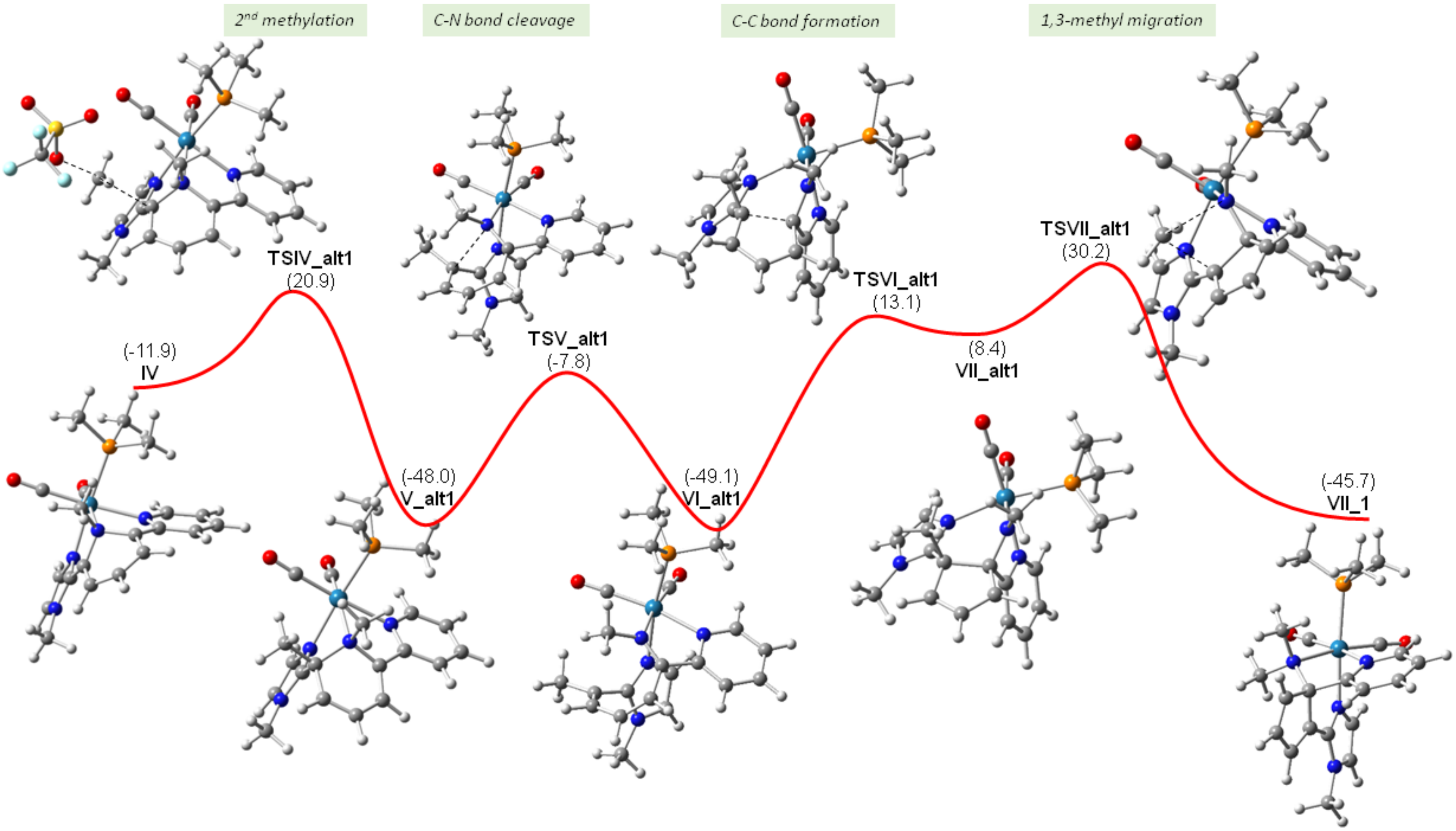

Figure S4. CPCM-B3LYP/6-31+G(d)-LANL2DZ Gibbs energy profile of two alternative routes to steps 4-6 of the most favored reaction mechanism found for the reactivity of *cis*,*trans*-[Re(bipy)(CO)$_2$(N-MeIm)(PMe$_3$)]OTf towards KN(SiMe$_3$)$_2$ and MeOTf to afford the dimethylated-N Re(I) product **VII_1**. All energies are given in kcal/mol and referenced to complex **I.**

b)

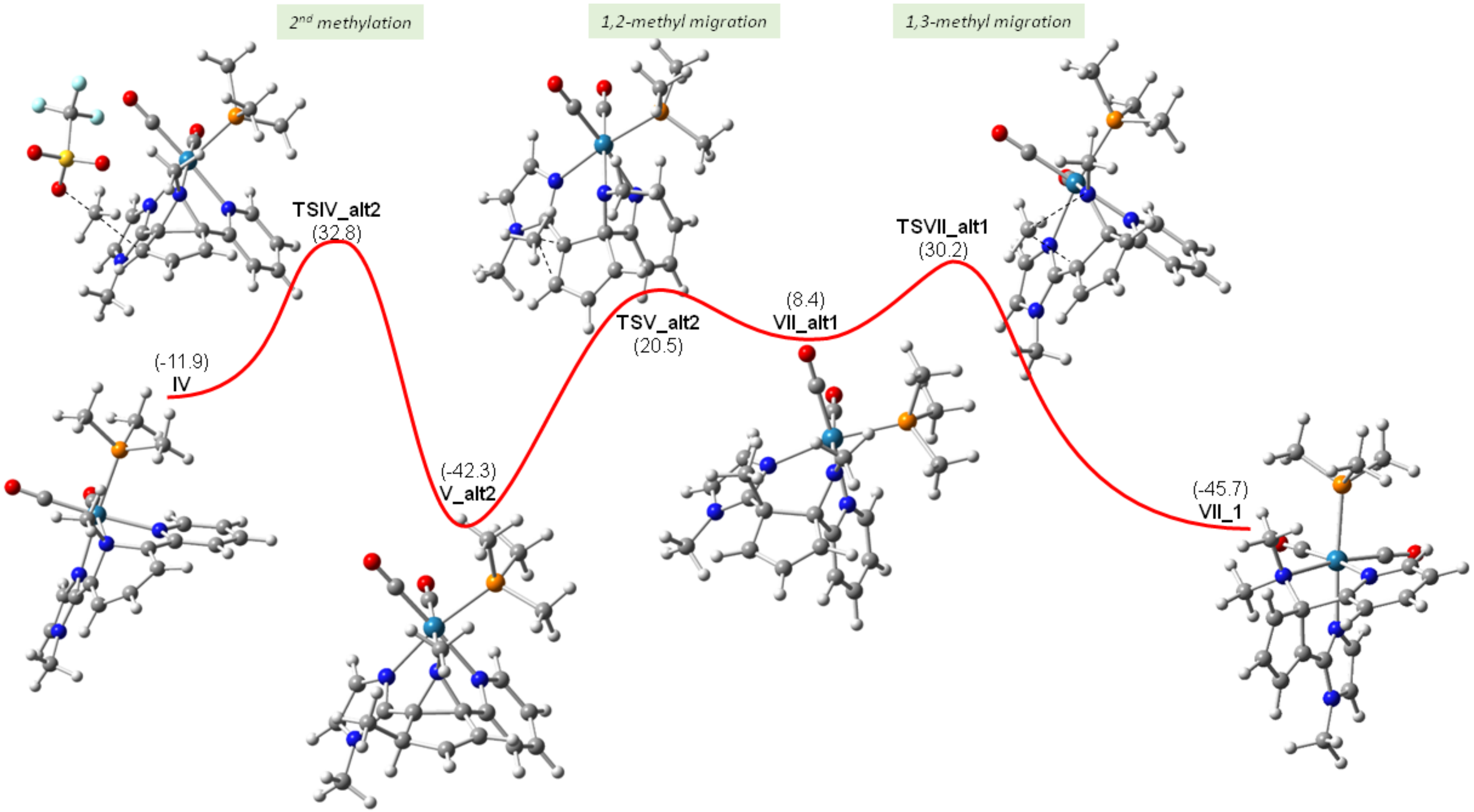

Figure S4. *(Cont.)*

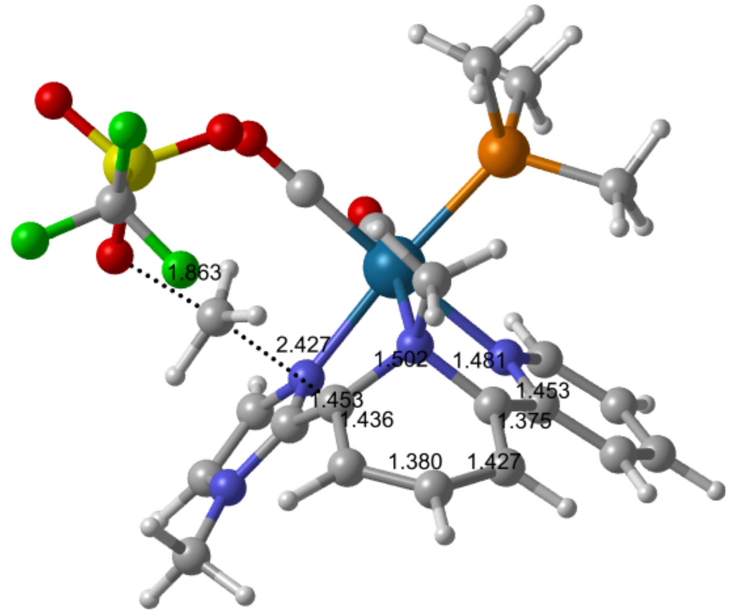

TSIV_alt1

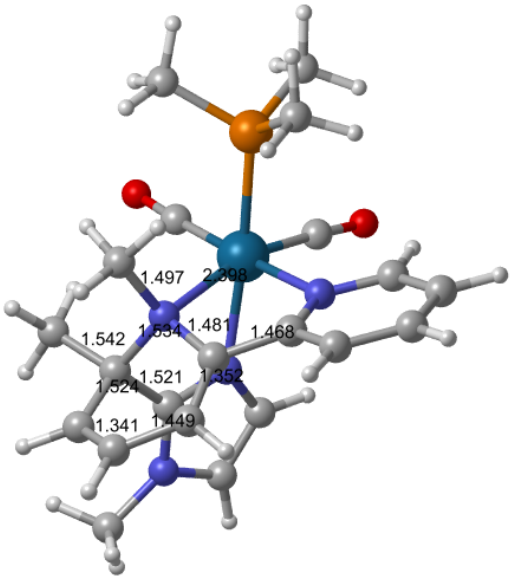

V_alt1

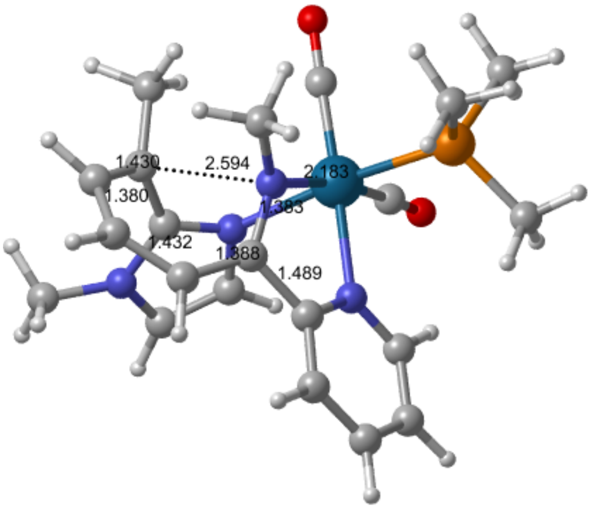

TSV_alt1

Figure S5. CPCM-B3LYP/6-31+G(d)-LANL2DZ optimized structures of the species involved in the alternative reaction mechanisms found for the reaction of the *cis,trans*-[Re(bipy)$(CO)_2$(*N*-MeIm)(PMe_3)]OTf compound with KN$(SiMe_3)_2$ and MeOTf in dichloromethane solution. Some relevant bond distances are included in angstroms.

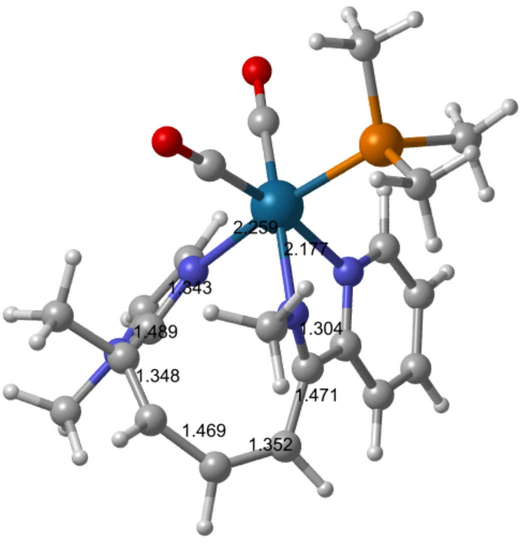

VI_alt1

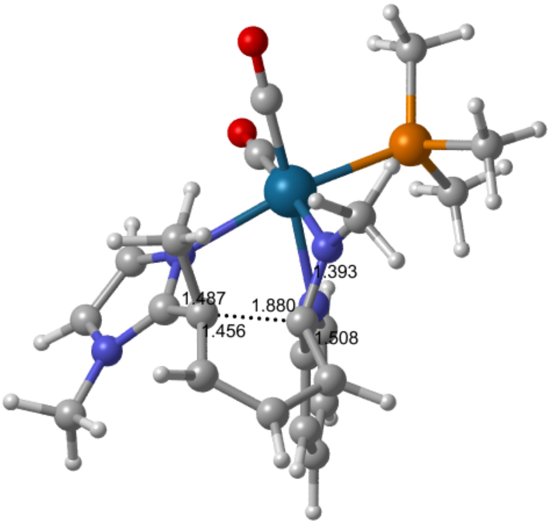

TSVI_alt1

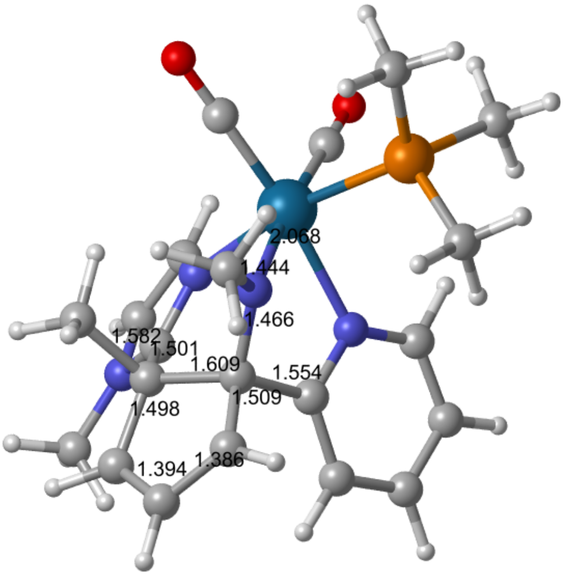

VII_alt1

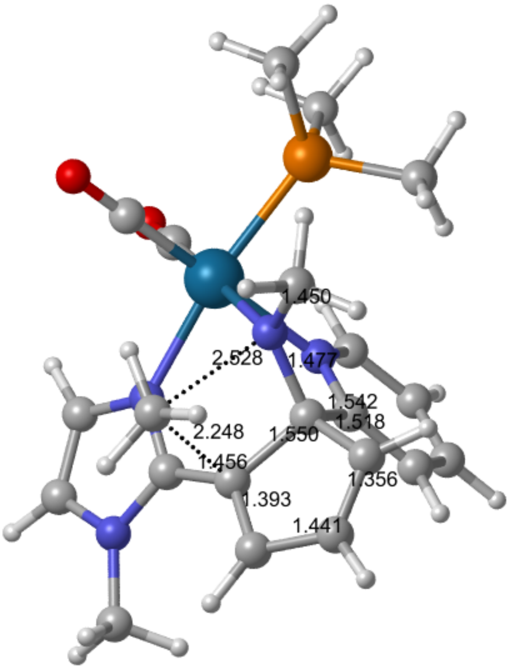

TSVII_alt1

Figure S5. *(cont.)*

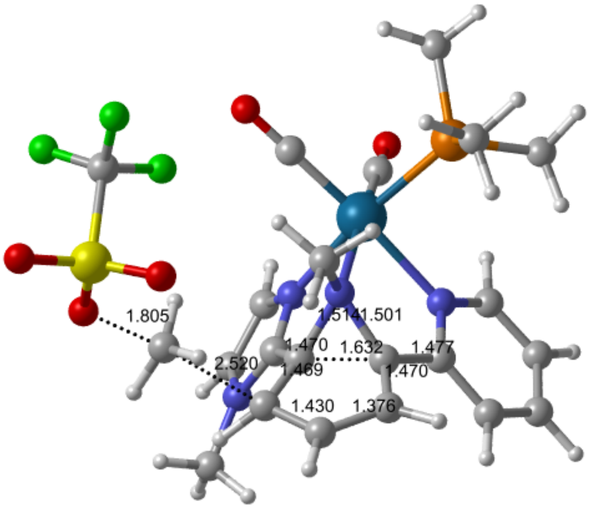

TSIV_alt2

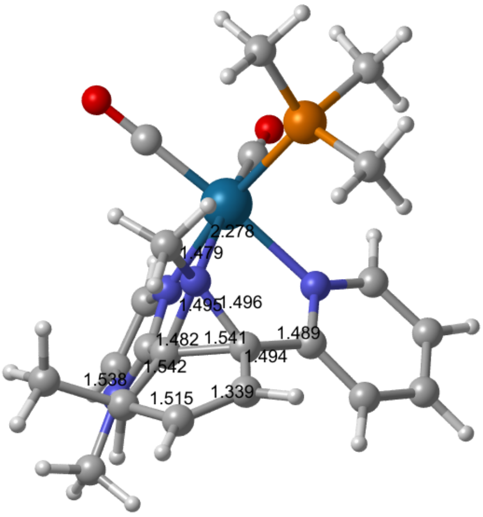

V_alt2

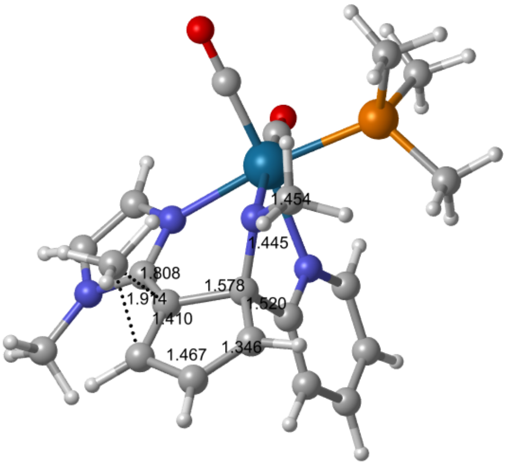

TSV_alt2

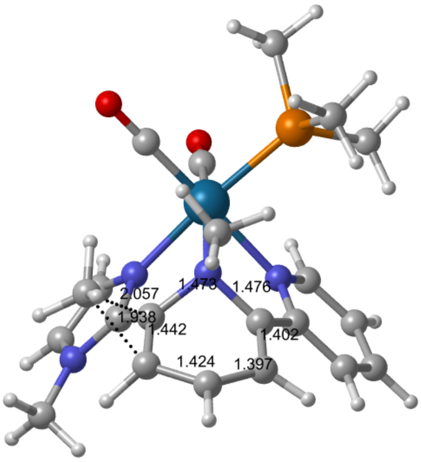

TSV_alt2_V_alt1

Figure S5. *(cont.)*

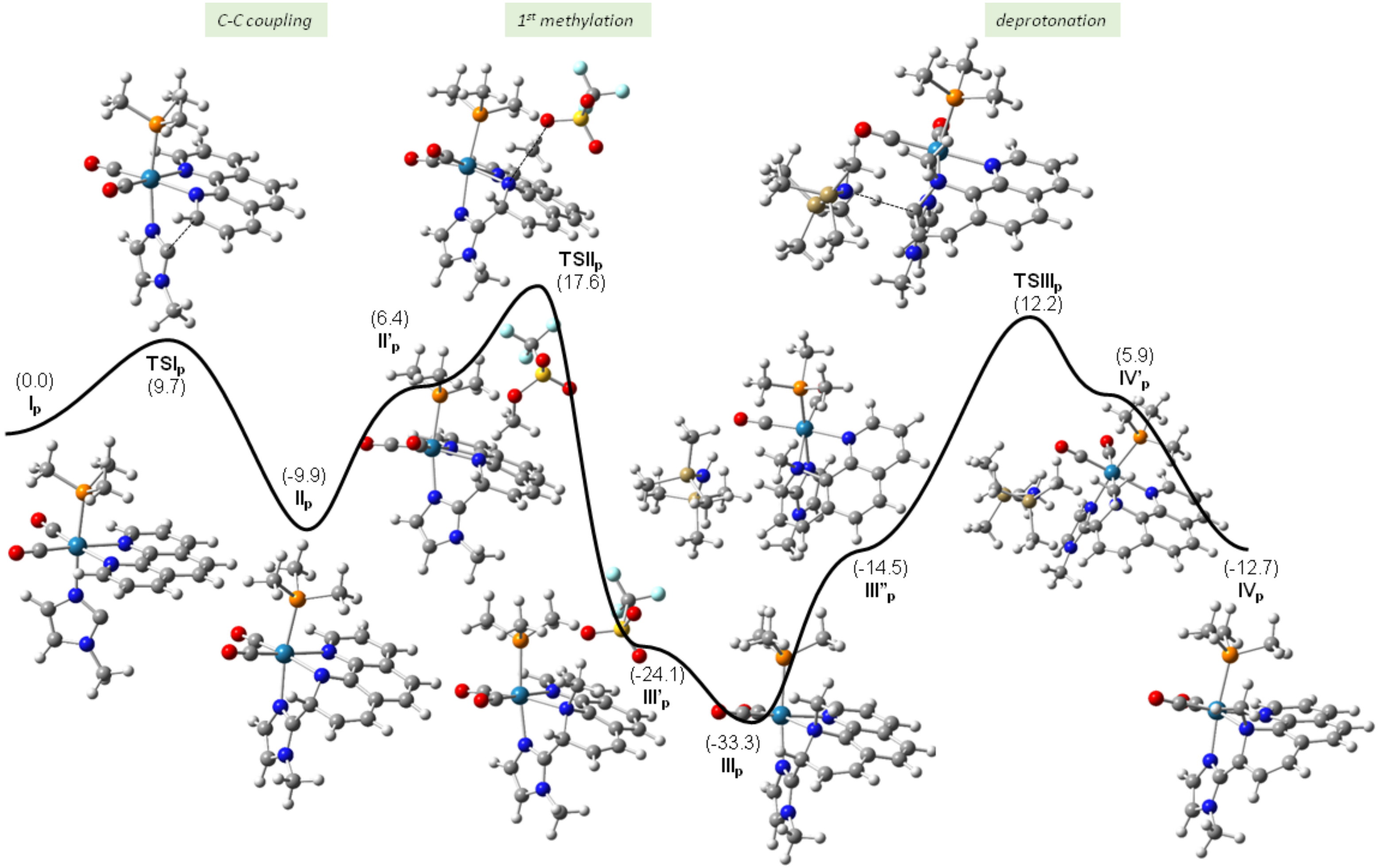

Figure S6. CPCM-B3LYP/6-31+G(d)-LANL2DZ Gibbs energy profile of the steps 1-3 involved in the reaction mechanism for the C-C coupling/methylation/deprotonation sequence of *cis*,*trans*-[Re(CO)$_2$(N-MeIm)(phen)(PMe$_3$)]OTf. All energies are given in kcal/mol and referenced to complex $\mathbf{I_p}$ (see Tables S3 and S4 for more energy details).

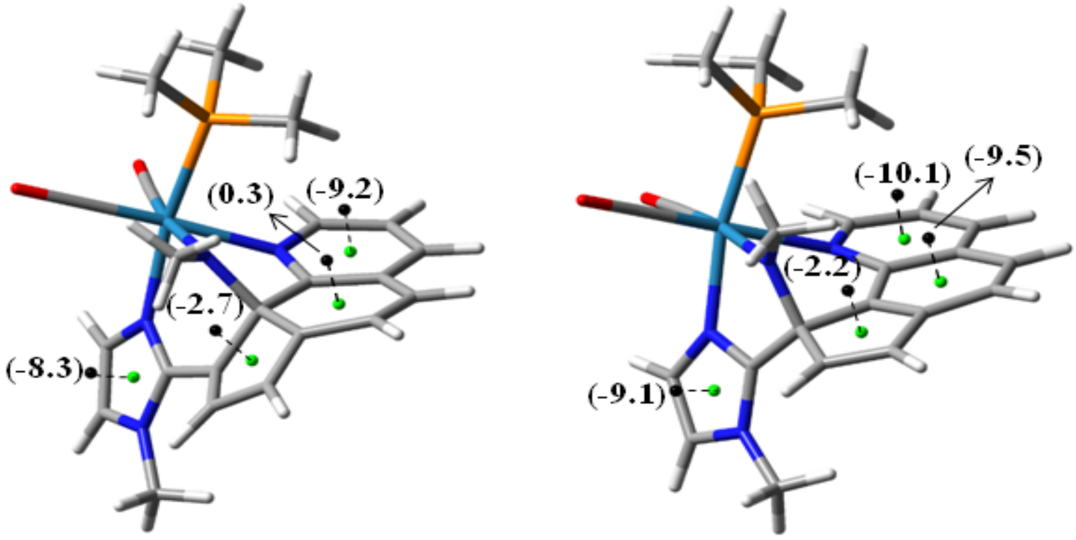

VI_1$_p$ **VI_2$_p$**

Figure S7. Nucleus-independent chemical shifts (black dots: in parenthesis in ppm) obtained at 1 Å (NICS(1)) above the ring critical points (RCP, green dots) of electron density located on the imidazole, cyclopentadiene, and pyridine rings of complexes **VI_I$_p$** and **VI_2$_p$** at the B3LYP/6-311+G(d,p)-LANL2DZ level of theory using the corresponding CPCM-B3LYP/6-31+G(d)-LANL2DZ optimized geometries.

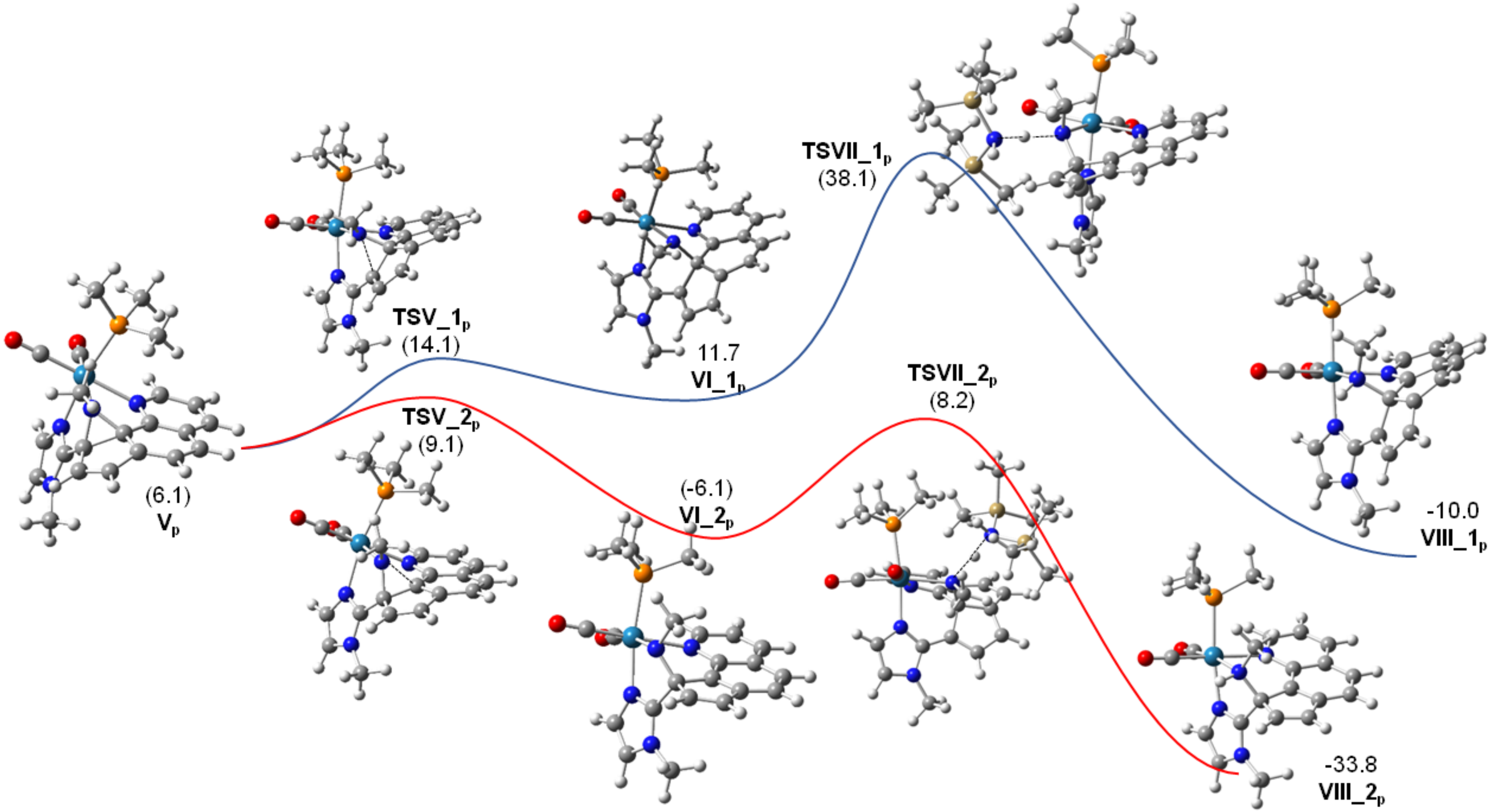

Figure S8. CPCM-B3LYP/6-31+G(d)-LANL2DZ Gibbs energy profile of protonation mechanism found for the reactivity of *cis*,*trans*-[Re(CO)$_2$(N-MeIm)(phen)(PMe$_3$)]OTf towards KN(SiMe$_3$)$_2$ and MeOTf to afford the protonated-N Re(I) products **VIII_1$_p$** (route in blue) and **VIII_2$_p$** (route in red). All energies are given in kcal/mol and referenced to complex **I$_p$** (see Tables S5 and S6 for more energy details).

Table S1. CPCM-B3LYP/6-31+G(d)-LANL2DZ electronic energy (E), enthalpy (H), entropy (S), and Gibbs energy (G) of the species involved in the reaction of the *cis,trans*-[Re(bipy)(CO)$_2$(*N*-MeIm)(PMe$_3$)]OTf compound with KN(SiMe$_3$)$_2$ and MeOTf in dichloromethane solution.

**Species**	**E** (hartree)	**H** (hartree)	**S** (cal k^{-1} mol^{-1})	**G** (hartree)
**I**	-1527.299498	-1526.886849	183.577	-1526.974072
MeOTf	-1001.331472	-1001.254463	98.537	-1001.301281
HN(SiMe$_3$)$_2$	-873.933736	-873.676265	120.395	-873.733468
OTf$^-$	-961.616964	-961.582377	86.050	-961.623262
H$_2$N(SiMe$_3$)$_2$$^+$	-874.375170	-874.102610	123.842	-874.161451
**TSI**	-1527.280693	-1526.869524	179.526	-1526.954823
**II**	-1527.310058	-1526.896816	175.494	-1526.980198
**II'**	-2528.637596	-2528.144910	234.692	-2528.256420
**TSII**	-2528.621965	-2528.130712	228.348	-2528.239207
**III'**	-2528.692775	-2528.198290	231.344	-2528.308209
**III**	-1567.069989	-1566.611752	178.675	-1566.696646
**III'**	-2440.998861	-2440.281133	255.983	-2440.402759
**TSIII**	-2440.954982	-2440.241393	245.235	-2440.357912
**IV'**	-2440.978570	-2440.260315	257.337	-2440.382584
**IV**	-1566.600608	-1566.157762	179.666	-1566.243127
**TSIV**	-1566.568660	-1566.129224	183.662	-1566.216488
**V**	-1566.568637	-1566.128127	188.239	-1566.217565
**TSV**	-1566.567555	-1566.127586	177.939	-1566.212130
**VI**	-1566.594757	-1566.153796	187.269	-1566.242774
**VI'_1**	-2567.923342	-2567.401148	235.568	-2567.513074
**TSVI_1**	-2567.908901	-2567.388606	232.755	-2567.499196
**VII'_1**	-2567.997545	-2567.473253	231.247	-2567.583126
**VII_1**	-1606.373371	-1605.886183	186.783	-1605.974930
**TSV_2**	-1566.563428	-1566.123712	178.515	-1566.208530

**VI_2**	-1566.577993	-1566.136258	184.259	-1566.223805
**VI'_2**	-2567.911188	-2567.389859	239.508	-2567.503657
**TSVI_2**	-2567.901933	-2567.381438	231.302	-2567.491338
**VII'_2**	-2567.991246	-2567.466456	233.537	-2567.577417
**VII_2**	-1606.364898	-1605.877930	187.547	-1605.967040
**TSIV_alt1**	-2567.902903	-2567.382388	231.019	-2567.492152
**V_alt1**	-1606.377021	-1605.889977	186.740	-1605.978703
**TSV_alt1**	-1606.306815	-1605.825428	187.763	-1605.914640
**VI_alt1**	-1606.375701	-1605.889864	190.367	-1605.980314
**TSVI_alt1**	-1606.273864	-1605.792396	187.088	-1605.881288
**VII_alt1**	-1606.282386	-1605.799552	187.777	-1605.888771
**TSVII_alt1**	-1606.246197	-1605.765214	186.820	-1605.853978
**TSIV_alt2**	-2567.880855	-2567.361501	234.888	-2567.473104
**V_alt2**	-1606.368592	-1605.881998	184.405	-1605.969615
**TSV_alt2**	-1606.261682	-1605.780204	187.822	-1605.869444
**TSV_alt2_V_alt1**	-1606.277667	-1605.794100	187.032	-1605.882965

Table S2. CPCM-B3LYP/6-31+G(d)-LANL2DZ relative electronic energy (ΔE), enthalpy (ΔH), entropy contribution (TΔS), and Gibbs energy (ΔG) of the species involved in the reaction of the *cis,trans*-[Re(bipy)(CO)$_2$(*N*-MeIm)(PMe$_3$)]OTf compound with KN(SiMe$_3$)$_2$ and MeOTf in dichloromethane solution. All the thermodynamic magnitudes are in kcal mol^{-1}.

Species	ΔE	ΔH	TΔS	ΔG
**I** + 2 MeOTf + HN(SiMe$_3$)$_2$	0.0	0.0	0.0	0.0
**TSI** + 2 MeOTf + HN(SiMe$_3$)$_2$	11.8	10.9	-1.2	12.1
**II** + 2 MeOTf + HN(SiMe$_3$)$_2$	-6.6	-6.2	-2.4	-3.8
**II'** + MeOTf + HN(SiMe$_3$)$_2$ + OTf$^-$	-4.2	-2.3	-14.1	11.9
**TSII** + MeOTf + HN(SiMe$_3$)$_2$ + OTf$^-$	5.6	6.6	-16.0	22.7
**III'** + MeOTf + HN(SiMe$_3$)$_2$ + OTf$^-$	-38.8	-35.7	-15.1	-20.6
**III +** MeOTf + HN(SiMe$_3$)$_2$ + OTf$^-$	-35.1	-33.1	-5.2	-28.0
**III''** + MeOTf + OTf$^-$	-32.1	-28.8	-18.0	-10.8
**TSIII +** MeOTf + TfO$^-$	-4.5	-3.9	-21.2	17.4
**IV'** + MeOTf + OTf$^-$	-19.3	-15.8	-17.6	1.9
**IV +** MeOTf + H$_2$N(SiMe$_3$)$_2^+$ + OTf$^-$	-17.6	-15.8	-3.9	-11.9
**TSIV +** MeOTf + H$_2$N(SiMe$_3$)$_2^+$ + OTf$^-$	2.4	2.1	-2.7	4.8
**V +** MeOTf + H$_2$N(SiMe$_3$)$_2^+$ + OTf$^-$	2.5	2.8	-1.3	4.1
**TSV_1** + MeOTf + H$_2$N(SiMe$_3$)$_2^+$ + OTf$^-$	3.2	3.1	-4.4	7.5
**VI_1** + MeOTf + H$_2$N(SiMe$_3$)$_2^+$ + OTf$^-$	-13.9	-13.3	-1.6	-11.7
**VI'_1** + H$_2$N(SiMe$_3$)$_2^+$ + OTf$^-$	-12.1	-8.8	-16.6	7.7
**TSVI_1** + H$_2$N(SiMe$_3$)$_2^+$ + OTf$^-$	-3.0	-1.0	-17.4	16.4
**VII'_1** + H$_2$N(SiMe$_3$)$_2^+$ + OTf$^-$	-58.7	-54.1	-17.9	-36.2
**VII_1** + H$_2$N(SiMe$_3$)$_2^+$ + 2 OTf$^-$	-54.2	-51.2	-5.5	-45.7
**TSV_2 +** MeOTf + H$_2$N(SiMe$_3$)$_2^+$ + OTf$^-$	5.7	5.6	-4.2	9.8
**VI_2 +** MeOTf + H$_2$N(SiMe$_3$)$_2^+$ + OTf$^-$	-3.4	-2.3	-2.5	0.2
**VI'_2** + H$_2$N(SiMe$_3$)$_2^+$ + OTf$^-$	-4.5	-1.8	-15.4	13.6
**TSVI_2** + H$_2$N(SiMe$_3$)$_2^+$ + OTf$^-$	1.3	3.5	-17.8	21.4
**VII'_2** + H$_2$N(SiMe$_3$)$_2^+$ + OTf$^-$	-54.7	-49.8	-17.2	-32.6

**VII_2** + $H_2N(SiMe_3)_2^+$ + 2 OTf^-	-48.8	-46.0	-5.2	-40.7
**TSIV_alt1** + $H_2N(SiMe_3)_2^+$ + OTf^-	0.7	2.9	-17.9	20.9
**V_alt1 +** $H_2N(SiMe_3)_2^+$ + 2 OTf^-	-56.4	-53.5	-5.5	-48.0
**TSV_alt1 +** $H_2N(SiMe_3)_2^+$ + 2 OTf^-	-12.4	-13.0	-5.2	-7.8
**VI_alt1 +** $H_2N(SiMe_3)_2^+$ + 2 OTf^-	-55.6	-53.5	-4.4	-49.1
**TSVI_alt1 +** $H_2N(SiMe_3)_2^+$ + 2 OTf^-	8.3	7.7	-5.4	13.1
**VII_alt1 +** $H_2N(SiMe_3)_2^+$ + 2 OTf^-	3.0	3.2	-5.2	8.4
**TSVII_alt1 +** $H_2N(SiMe_3)_2^+$ + 2 OTf^-	25.6	24.8	-5.4	30.2
**TSIV_alt2** + $H_2N(SiMe_3)_2^+$ + OTf^-	14.6	16.0	-16.8	32.8
**V_alt2 +** $H_2N(SiMe_3)_2^+$ + 2 OTf^-	-51.2	-48.5	-6.2	-42.4
**TSV_alt2 +** $H_2N(SiMe_3)_2^+$ + 2 OTf^-	15.9	15.4	-5.2	20.5
**TSV_alt2_V_alt1 +** $H_2N(SiMe_3)_2^+$ + 2 OTf^-	5.9	6.6	-5.4	12.0

Table S3. CPCM-B3LYP/6-31+G(d)-LANL2DZ electronic energy (E), enthalpy (H), entropy (S), and Gibbs energy (G) of the species involved in the reaction of the *cis,trans*-[Re(CO)$_2$(*N*-MeIm)(phen)(PMe$_3$)]OTf compound with KN(SiMe$_3$)$_2$ and MeOTf in dichloromethane solution.

**Species**	**E** (hartree)	**H** (hartree)	**S** (cal k^{-1} mol^{-1})	**G** (hartree)
**I$_p$**	-1603.532359	-1603.107433	183.751	-1603.194739
**TSI$_p$**	-1603.517229	-1603.092826	181.960	-1603.179281
**II$_p$**	-1603.552534	-1603.125691	178.460	-1603.210483
**II'$_p$**	-2604.880873	-2604.374113	235.160	-2604.485845
**TSII$_p$**	-2604.863529	-2604.358536	230.371	-2604.467992
**III'$_p$**	-2604.933500	-2604.425299	229.784	-2604.534476
**III$_p$**	-1643.310782	-1642.839015	182.668	-1642.925806
**III''$_p$**	-2517.239829	-2516.508040	255.307	-2516.629344
**TSIII$_p$**	-2517.195591	-2516.468316	249.225	-2516.586730
**IV'$_p$**	-2517.208040	-2516.476943	252.321	-2516.596829
**IV$_p$**	-1642.833852	-1642.377994	183.031	-1642.464959
**TSIV$_p$**	-1642.801813	-1642.349384	180.455	-1642.435124
**V$_p$**	-1642.802127	-1642.347409	184.314	-1642.434983
**TSV_1$_p$**	-1642.788882	-1642.335968	181.657	-1642.422279
**VI_1$_p$**	-1642.792341	-1642.337935	185.538	-1642.426090
**VI'_1$_p$**	-2644.120704	-2643.586564	240.634	-2643.700897
**TSVI_1$_p$**	-2644.105381	-2643.572843	236.128	-2643.685035
**VII'_1$_p$**	-2644.192417	-2643.655447	234.122	-2643.766686
**VII_1$_p$**	-1682.569420	-1682.069503	188.606	-1682.159116
**TSV_2$_p$**	-1642.797237	-1642.343988	181.546	-1642.430246
**VI_2$_p$**	-1642.822627	-1642.366902	184.274	-1642.454456
**VI'_2$_p$**	-2644.155007	-2643.619681	237.823	-2643.732678
**TSVI_2$_p$**	-2644.146028	-2643.611554	233.060	-2643.722288
**VII'_2$_p$**	-2644.23500	-2643.697178	236.090	-2643.809351

**VII_2p**	-1682.609495	-1682.107979	186.362	-1682.196526

Table S4. CPCM-B3LYP/6-31+G(d)-LANL2DZ relative electronic energy (ΔE), enthalpy (ΔH), entropy contribution (TΔS), and Gibbs energy (ΔG) of the species involved in the reaction of the *cis,trans*-[Re(phen)$(CO)_2$(*N*-MeIm)(PMe_3)]OTf compound with $KN(SiMe_3)_2$ and MeOTf in dichloromethane solution. All the thermodynamic magnitudes are in kcal mol^{-1}.

Species	ΔE	ΔH	TΔS	ΔG
$\mathbf{I_p}$ + 2 MeOTf + $HN(SiMe_3)_2$	0.0	0.0	0.0	0.0
$\mathbf{TSI_p}$ + 2 MeOTf + $HN(SiMe_3)_2$	9.5	9.2	-0.5	9.7
$\mathbf{II_p}$ + 2 MeOTf + $HN(SiMe_3)_2$	-12.7	-11.5	-1.6	-9.9
$\mathbf{II'_p}$ + MeOTf + $HN(SiMe_3)_2$ + OTf^-	-10.7	-7.7	-14.1	6.4
$\mathbf{TSII_p}$ + MeOTf + $HN(SiMe_3)_2$ + OTf^-	0.2	2.1	-15.5	17.6
$\mathbf{III'_p}$ + MeOTf + $HN(SiMe_3)_2$ + OTf^-	-43.7	-39.8	-15.7	-24.1
$\mathbf{III_p}$ + MeOTf + $HN(SiMe_3)_2$ + OTf^-	-40.1	-37.3	-4.0	-33.3
$\mathbf{III''_p}$ + MeOTf + OTf^-	-37.2	-32.8	-18.3	-14.5
$\mathbf{TSIII_p}$ + MeOTf + TfO^-	-9.4	-7.9	-20.1	12.2
$\mathbf{IV'_p}$ + MeOTf + OTf^-	-17.2	-13.3	-19.2	5.9
$\mathbf{IV_p}$ + MeOTf + $H_2N(SiMe_3)_2^+$ + OTf^-	-17.8	-15.6	-2.9	-12.7
$\mathbf{TSIV_p}$ + MeOTf + $H_2N(SiMe_3)_2^+$ + OTf^-	2.3	2.4	-3.7	6.1
$\mathbf{V_p}$ + MeOTf + $H_2N(SiMe_3)_2^+$ + OTf^-	2.1	3.6	-2.5	6.1
$\mathbf{TSV\_1_p}$ + MeOTf + $H_2N(SiMe_3)_2^+$ + OTf^-	10.4	10.8	-3.3	14.1
$\mathbf{VI\_1_p}$ + MeOTf + $H_2N(SiMe_3)_2^+$ + OTf^-	8.2	9.6	-2.2	11.7
$\mathbf{VI'\_1_p}$ + $H_2N(SiMe_3)_2^+$ + OTf^-	10.2	13.2	-15.1	28.3
$\mathbf{TSVI\_1_p}$ + $H_2N(SiMe_3)_2^+$ + OTf^-	19.8	21.8	-16.5	38.3
$\mathbf{VII''\_1_p}$ + $H_2N(SiMe_3)_2^+$ + OTf^-	-34.8	-30.0	-17.1	-12.9
$\mathbf{VII\_1_p}$ + $H_2N(SiMe_3)_2^+$ + 2 OTf^-	-31.0	-27.8	-5.0	-22.8
$\mathbf{TSV\_2_p}$ + MeOTf + $H_2N(SiMe_3)_2^+$ + OTf^-	5.1	5.8	-3.4	9.1
$\mathbf{VI\_2_p}$ + MeOTf + $H_2N(SiMe_3)_2^+$ + OTf^-	-10.8	-8.6	-2.5	-6.1
$\mathbf{VI'\_2_p}$ + $H_2N(SiMe_3)_2^+$ + OTf^-	-11.4	-7.6	-16.0	8.4
$\mathbf{TSVI\_2_p}$ + $H_2N(SiMe_3)_2^+$ + OTf^-	-5.7	-2.5	-17.4	14.9
$\mathbf{VII''\_2_p}$ + $H_2N(SiMe_3)_2^+$ + OTf^-	-61.6	-56.2	-16.5	-39.7

**VII_2$_p$** + $H_2N(SiMe_3)_2^+$ + 2 OTf^-	-56.2	-51.9	-5.6	-46.3

Table S5. CPCM-B3LYP/6-31+G(d)-LANL2DZ electronic energy (E), enthalpy (H), entropy (S), and Gibbs energy (G) of the species involved in the protonation sequence of the reaction of the *cis,trans*-[Re(CO)$_2$(*N*-MeIm)(phen)(PMe$_3$)]OTf compound with KN(SiMe$_3$)$_2$ and MeOTf in dichloromethane solution.

**Species**	**E** (hartree)	**H** (hartree)	**S** (cal k^{-1} mol^{-1})	**G** (hartree)
**TSVII_1$_p$**	-2517.150997	-2516.424728	254.220	-2516.545516
**VIII_1$_p$**	-1643.272120	-1642.801536	183.447	-1642.888698
**TSVII_2$_p$**	-2517.202766	-2516.475064	248.480	-2516.593125
**VIII_2$_p$**	-1643.311175	-1642.839305	183.879	-1642.926672

Table S6. CPCM-B3LYP/6-31+G(d)-LANL2DZ relative electronic energy (ΔE), enthalpy (ΔH), entropy contribution (TΔS), and Gibbs energy (ΔG) of the species involved in the protonation sequence of the reaction of the *cis,trans*-[Re(CO)$_2$(*N*-MeIm)(phen)(PMe$_3$)]OTf compound with KN(SiMe$_3$)$_2$ and MeOTf in dichloromethane solution. All the thermodynamic magnitudes are in kcal mol^{-1}.

**Species**	**ΔE**	**ΔH**	**TΔS**	**ΔG**
**TSVII_1$_p$** + OTf^- + $HN(SiMe_3)_2$	18.6	19.5	-18.6	38.1
**VIII_1$_p$** + OTf^- + $HN(SiMe_3)_2$	-15.8	-13.8	-3.8	-10.0
**TSVII_2$_p$** + OTf^- + $HN(SiMe_3)_2$	-13.9	-12.1	-20.3	8.2
**VIII_2$_p$** + $MeOTf^-$ + $HN(SiMe_3)_2$	-40.4	-37.5	-3.7	-33.8

Discussion about the effect of solvent on the C-C coupling step

The substitution of the experimentally used solvent tetrahydrofuran (THF, $\varepsilon = 7.4257$) by dichloromethane (CH_2Cl_2, $\varepsilon = 8.93$) in the deprotonation of the *cis*,*trans*-[Re(CO)$_2$(N-N)(N-MeIm)(PMe$_3$)]OTf (N-N = bipy, phen) complexes to give rise the respective C-C coupling compounds was analyzed in order to check its influence on the Gibbs energy profiles displayed in Figures 1-3 and S6 for the formation of the N-dimethylated Re(I) products. Figures S9 and S10 collect the optimized geometries of the species involved in the C-C coupling step for both the bipy case (species **I**, **TSI**, and **II**) and phen case (species $\mathbf{I_p}$, $\mathbf{TSI_p}$, and $\mathbf{II_p}$) in THF and CH_2Cl_2 solutions at the CPCM-B3LYP/6-31+G(d)-LANL2DZ level of theory, respectively. As expected given the small difference in dielectric permittivity of these solvents, the optimized geometries obtained do not show significant differences when going from THF to CH_2Cl_2 (compare species in Figure S9 with the analogous ones in Figure S10). All the energy data ascribed to these geometries are collected in Tables S1-S4, S7, and S8. As seen in Table S8, the Gibbs energy barrier of the C-C coupling step with bipy (transformation $\mathbf{1} \rightarrow \mathbf{TSI}$) varies from 12.7 to 12.1 kcal/mol when replacing THF by CH_2Cl_2, while ranges from 10.1 to 9.7 kcal/mol with phen (transformation $\mathbf{I_p} \rightarrow \mathbf{TSI_p}$). In terms of relative Gibbs energies, the C-C coupling complex with bipy, **II**, destabilizes by only 1.1 kcal/mol upon substitution of THF for CH_2Cl_2, whereas a small estabilization of 0.5 kcal/mol was found for the phen $\mathbf{II_p}$ analogue. Therefore, these small energy changes do not have a significant impact on the fact that we have reported the energies of all species involved in the reaction mechanism found for bipy and phen in CH_2Cl_2 solution.

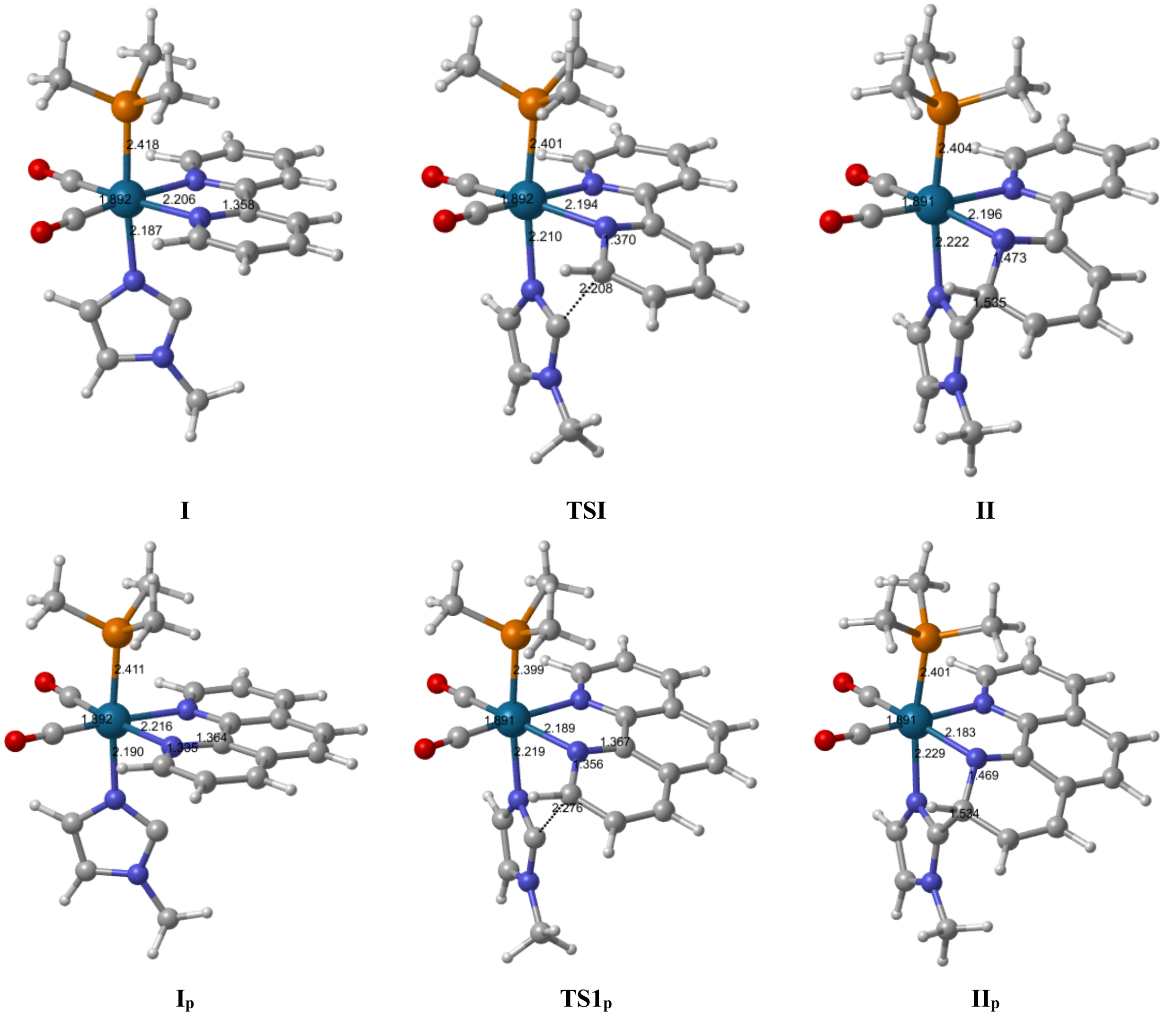

Figure S9. CPCM-B3LYP/6-31+G(d)-LANL2DZ optimized structures of the species involved in the C-C coupling step for the reaction of the *cis,trans*-$[Re(CO)_2(N\text{-}MeIm)(N\text{-}N)(PMe_3)]OTf$ (N-N = bipy, phen) compounds with $KN(SiMe_3)_2$ and MeOTf in tetrahydrofuran solution. Some relevant bond distances are included in angstroms.

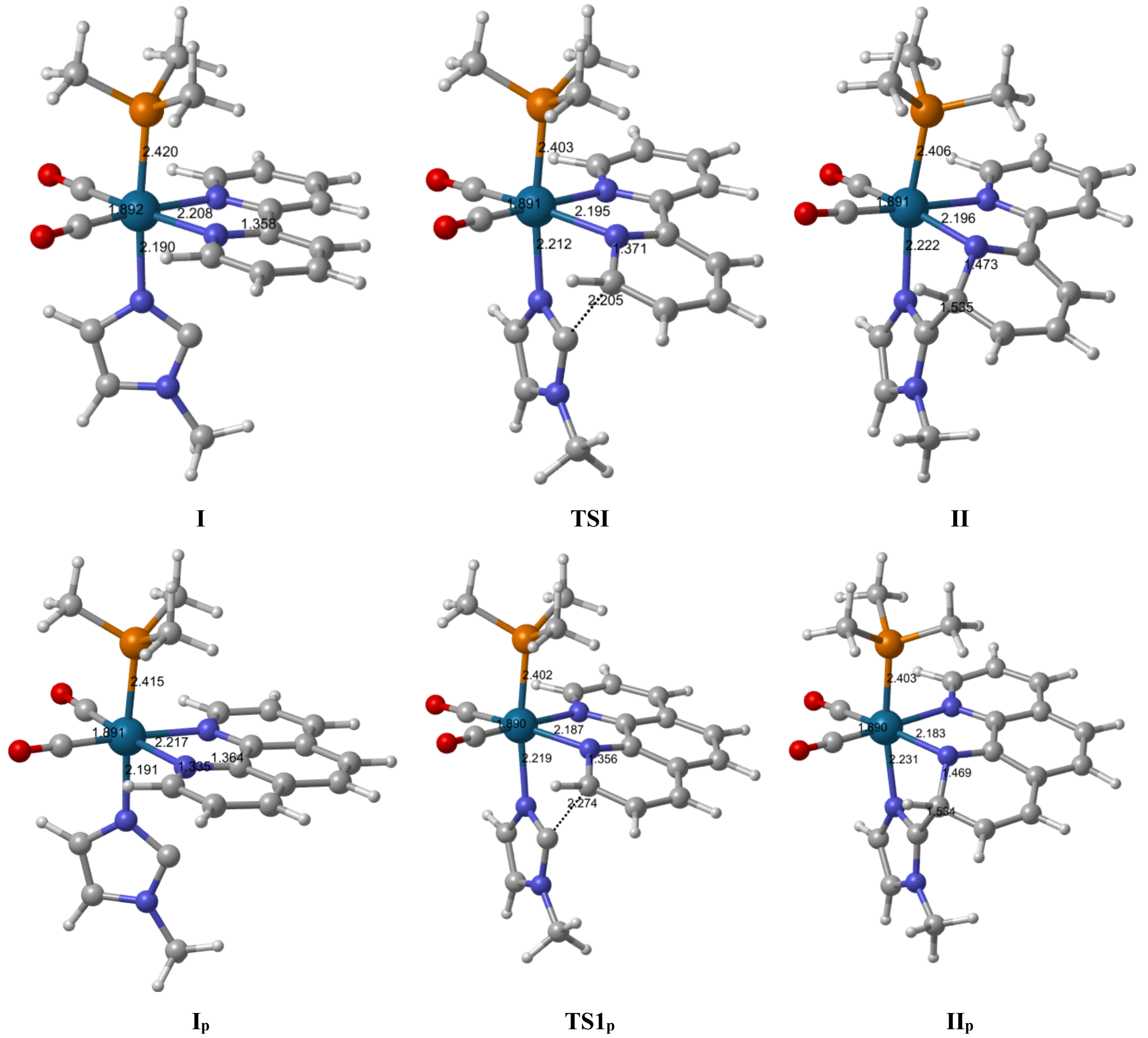

Figure S10. CPCM-B3LYP/6-31+G(d)-LANL2DZ optimized structures of the species involved in the C-C coupling step for the reaction of the *cis,trans*-[Re(CO)$_2$(*N*-MeIm)(N-N)(PMe$_3$)]OTf (N-N = bipy, phen) compounds with KN(SiMe$_3$)$_2$ and MeOTf in dichloromethane solution. Some relevant bond distances are included in angstroms.

Table S7. CPCM-B3LYP/6-31+G(d)-LANL2DZ electronic energy (E), enthalpy (H), entropy (S), and Gibbs energy (G) of the species involved in the C-C coupling step for the reaction of the *cis,trans*-[Re(CO)$_2$(N-N)(*N*-MeIm)(PMe$_3$)]OTf (N-N = bipy, phen) compounds with KN(SiMe$_3$)$_2$ and MeOTf in tetrahydrofuran solution.

**Species**	**E** (hartree)	**H** (hartree)	**S** (cal k^{-1} mol^{-1})	**G** (hartree)
**I**	-1527.291226	-1526.878375	183.786	-1526.965698
**TSI**	-1527.272793	-1526.861213	177.452	-1526.945526
**II**	-1527.302431	-1526.889095	177.745	-1526.973548
**I$_p$**	-1603.523896	-1603.097600	187.600	-1603.186735
**TSI$_p$**	-1603.509013	-1603.084468	181.340	-1603.170628
**II$_p$**	-1603.544755	-1603.117511	177.092	-1603.201653

Table S8. CPCM-B3LYP/6-31+G(d)-LANL2DZ relative electronic energy (ΔE), enthalpy (ΔH), entropy contribution (TΔS), and Gibbs energy (ΔG) of the species involved in the C-C coupling step for the reaction of the *cis,trans*-[Re(CO)$_2$(N-N)(*N*-MeIm)(PMe$_3$)]OTf (N-N = bipy, phen) compounds with KN(SiMe$_3$)$_2$ and MeOTf in tetrahydrofuran solution. All the thermodynamic magnitudes are in kcal mol^{-1}. For comparison purposes values obtained in dichloromethane solution are also included in parenthesis.

**Species**	**ΔE**	**ΔH**	**TΔS**	**ΔG**
**I**	0.0 (0.0)	0.0 (0.0)	0.0 (0.0)	0.0 (0.0)
**TSI**	11.6 (11.8)	10.8 (10.9)	-1.9 (-1.2)	12.7 (12.1)
**II**	-7.0 (-6.6)	-6.7 (-6.2)	-1.8 (-2.4)	-4.9 (-3.8)
**I$_p$**	0.0 (0.0)	0.0 (0.0)	0.0 (0.0)	0.0 (0.0)
**TSI$_p$**	9.3 (9.5)	8.2 (9.2)	-1.9 (-0.5)	10.1 (9.7)
**II$_p$**	-13.1 (-12.7)	-12.5 (-11.5)	-3.1 (-1.6)	-9.4 (-9.9)

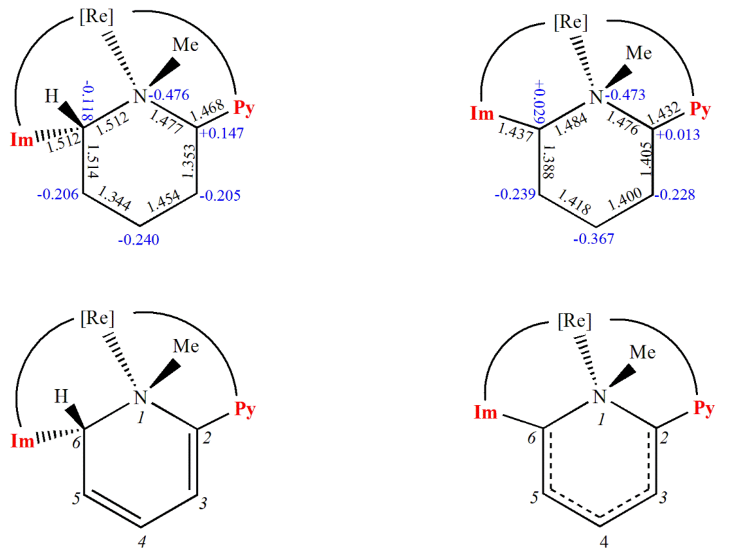

III **IV**

Figure S11. CPCM-B3LYP/6-31+G(d)-LANL2DZ bond lengths in Å (in black) and net natural atomic charges in *e* (in blue) for the non-hydrogen atoms of the dearomatized pyridine ring of the bipy ligand in complexes **III** and **IV** at the top of the image. Lewis structures deduced from the bond lengths are shown at the bottom of the image. Atom numbering for the non-hydrogen atoms is also included in italics.

Table S9. CPCM-B3LYP/6-31+G(d)-LANL2DZ cartesian coordinates of the species involved in the reaction of the *cis,trans*-[Re(bipy)(CO)$_2$(*N*-MeIm)(PMe$_3$)]OTf compound with KN(SiMe$_3$)$_2$ and MeOTf in dichloromethane solution. The imaginary harmonic vibrational frequencies obtained for the TSs located are also included in parentheses.

I

Re	0.046281	0.044051	-0.072025
C	-0.266787	0.070283	1.793344
C	1.915129	0.088437	0.228060
P	0.076028	-2.376084	-0.051260
N	0.079441	0.025299	-2.283828
C	1.206313	0.025606	-3.021251
C	1.198694	0.021169	-4.411891
H	2.136772	0.029770	-2.466649
H	2.136019	0.027198	-4.955100
C	-0.030010	0.002948	-5.073004
C	-1.198650	0.001167	-4.317284
H	-0.079401	-0.009938	-6.156161
H	-2.161240	-0.016859	-4.811617
C	-1.121264	0.025618	-2.920225
C	-2.312216	0.034508	-2.046795
N	-2.066460	0.016531	-0.711362
C	-3.621216	0.051718	-2.540342
C	-3.106977	0.021389	0.142636
C	-4.431002	0.042734	-0.281754
H	-2.856111	0.012199	1.196181
H	-5.229830	0.048226	0.450079
C	-4.692667	0.057079	-1.652208
H	-3.803985	0.071888	-3.606797
H	-5.710711	0.074823	-2.024974
O	3.085446	0.112159	0.377637
O	-0.511928	0.077849	2.947744
C	-1.566137	-3.222671	-0.080617
C	0.954785	-3.224068	-1.437739
C	0.882776	-3.157067	1.411297
H	0.847872	-4.310072	-1.350118
H	0.550506	-2.898429	-2.399579
H	2.017116	-2.967580	-1.404452
H	0.837281	-4.249190	1.346148
H	1.929777	-2.843186	1.457915
H	0.384147	-2.828632	2.327542
H	-1.440350	-4.309657	-0.061121
H	-2.158142	-2.912612	0.784774
H	-2.111591	-2.940848	-0.985413
N	-0.095332	2.221521	-0.253392
C	-0.856665	2.929946	-1.140095
C	0.608413	3.088789	0.572100
C	0.300035	4.370888	0.217916
N	-0.598859	4.253907	-0.831364
H	1.274763	2.733360	1.344900
H	0.629943	5.322746	0.608406
C	-1.202126	5.387998	-1.508880
H	-1.835977	5.003673	-2.308300
H	-1.814360	5.979331	-0.819528
H	-0.434873	6.039998	-1.939682

MeOTf

S	-0.222423	-0.169690	0.182553
O	-0.237995	0.003281	1.631330
O	1.273014	-0.081114	-0.400402
O	-0.968288	-1.261632	-0.434264
C	-0.789725	1.443490	-0.604744
F	-0.058051	2.458757	-0.149642
F	-0.687611	1.368319	-1.930320
F	-2.067545	1.626349	-0.264947
C	2.188609	-1.174372	-0.054632
H	3.117342	-0.903613	-0.548792
H	2.319656	-1.211593	1.026679
H	1.798826	-2.113699	-0.446456

HN(SiMe$_3$)$_2$

C	-1.575339	-1.110259	-1.450948
Si	-1.591195	-0.016877	0.093764
C	-2.088583	1.733294	-0.427289
N	0.002102	-0.037389	0.831958
Si	1.594231	0.009067	0.093424
C	2.839576	0.480981	1.432824
C	2.068397	-1.667465	-0.648687
C	1.575003	1.286729	-1.301621
C	-2.823869	-0.669567	1.365785
H	0.002234	-0.153402	1.841771
H	-3.849587	-0.602297	0.985034
H	-2.625111	-1.719030	1.612290
H	-2.777137	-0.089827	2.295647
H	-3.057376	1.733178	-0.942555
H	-2.169262	2.397242	0.441591
H	-1.345445	2.163786	-1.109371
H	-2.546434	-1.087470	-1.960139
H	-0.818444	-0.774615	-2.170835
H	-1.348409	-2.152039	-1.195737
H	2.534086	1.304896	-1.833094
H	0.795056	1.057551	-2.037934
H	1.380837	2.293213	-0.915060
H	3.043643	-1.621721	-1.149961
H	2.122674	-2.443318	0.124904
H	1.323312	-1.986852	-1.387431
H	3.856232	0.524054	1.024749
H	2.608974	1.460960	1.865785
H	2.841818	-0.255522	2.245611

OTf$^-$

S	-0.918241	-0.000066	-0.000188
O	-1.250299	1.389124	-0.400379
O	-1.251403	-1.041900	-1.001577
O	-1.249611	-0.346630	1.403181
C	0.957012	-0.000278	-0.000320
F	1.442880	-1.206245	0.356446
F	1.443140	0.911459	0.866015
F	1.442901	0.294561	-1.223003

H$_2$N(SiMe$_3$)$_2^+$

N	0.000157	-0.045451	0.881155
H	0.000423	0.711151	1.574510
H	-0.005136	-0.895760	1.457114
Si	-1.697478	0.008227	0.023786
Si	1.697856	-0.004452	0.023908
C	1.661200	1.374376	-1.238761

C	2.874224	0.345053	1.438405
C	1.900688	-1.713550	-0.706014
H	3.900547	0.398707	1.057616
H	2.649958	1.303178	1.920280
H	2.841101	-0.442097	2.200094
H	2.884201	-1.786461	-1.185235
H	1.850405	-2.489983	0.065484
H	1.143995	-1.930790	-1.465045
H	2.681617	1.525394	-1.609007
H	1.029727	1.144601	-2.102317
H	1.325510	2.320333	-0.801341
C	-1.866970	1.721530	-0.702357
C	-1.693748	-1.368548	-1.242505
C	-2.875166	-0.311087	1.443634
H	-2.711991	-1.487076	-1.630572
H	-1.040282	-1.159041	-2.094922
H	-1.395295	-2.326014	-0.802809
H	-2.877653	1.827658	-1.113811
H	-1.734600	2.501615	0.054562
H	-1.158015	1.900620	-1.514835
H	-3.905061	-0.342225	1.070383
H	-2.667999	-1.272473	1.926754
H	-2.819216	0.477335	2.202890

TSI (ν = 228i cm^{-1})

N	-2.138909	-0.661803	0.590556
C	-1.582531	-0.455562	1.815409
C	-2.254250	-0.833370	2.985839
C	-3.521318	-1.400023	2.901865
C	-4.097351	-1.581244	1.641418
C	-3.371470	-1.205709	0.518298
C	-0.283547	0.243858	1.805087
N	0.336092	0.305665	0.589109
C	1.263724	1.298939	0.410975
C	1.938221	1.852689	1.548802
C	1.414405	1.639499	2.798650
C	0.227220	0.878854	2.928599
C	-0.215411	2.690549	-0.449000
N	-0.684918	3.972229	-0.549563
C	-1.943345	3.975856	-1.134525
C	-2.231104	2.661734	-1.390617
N	-1.170857	1.886419	-0.959008
C	0.041741	5.154567	-0.114605
Re	-0.831199	-0.289170	-1.171448
C	0.456452	-0.002808	-2.526960
O	1.277909	0.174374	-3.356092
P	-0.184383	-2.602423	-1.105543
C	1.538236	-2.893686	-0.507988
C	-2.156891	-0.602080	-2.485005
O	-3.033168	-0.775180	-3.260542
C	-1.152705	-3.758651	-0.037651
C	-0.179556	-3.499935	-2.716793
H	-0.269501	0.795555	3.886954
H	-5.085507	-2.010443	1.525185
H	2.817736	2.468124	1.393502
H	-3.770650	-1.342279	-0.479589
H	-2.504098	4.882334	-1.308724
H	-1.784763	-0.683968	3.950066
H	-3.108844	2.228919	-1.848665
H	1.790764	1.264621	-0.531047
H	-0.534429	5.711298	0.629954
H	0.981723	4.825842	0.329940
H	0.255918	5.814120	-0.961396
H	-4.049009	-1.695940	3.802203
H	1.883423	2.064502	3.680670
H	0.148312	-4.536546	-2.585341
H	-1.185676	-3.489739	-3.144968
H	0.494903	-2.992985	-3.413046
H	-0.749061	-4.773564	-0.109060
H	-1.111446	-3.428910	1.004643
H	-2.199167	-3.766960	-0.353971
H	1.775399	-3.962779	-0.497140
H	2.240074	-2.376266	-1.168497
H	1.649923	-2.485103	0.499615

I

N	-0.219058	3.746135	-0.259658
C	-0.037038	2.404918	-0.171015
N	-0.929547	1.771192	-0.937455
C	-1.707210	2.734100	-1.539498
C	-1.277470	3.968325	-1.126100
C	1.003216	1.548784	0.565321
N	0.332359	0.250945	0.752063
C	-0.487691	0.222061	1.851911
C	-0.274777	0.989697	2.983663
C	0.886327	1.836889	3.012381
C	1.557157	2.096669	1.864504
C	-1.693292	-0.619784	1.727941
C	-2.453079	-1.075861	2.815864
C	-3.632957	-1.774013	2.591384
C	-4.037307	-2.013303	1.272682
C	-3.226204	-1.565171	0.239065
N	-2.076694	-0.888731	0.449554
Re	-0.575039	-0.413860	-1.134532
P	0.082349	-2.719243	-0.925999
C	0.183254	-3.350628	0.808124
C	0.581797	4.792169	0.374757
C	-1.718954	-0.748672	-2.607340
O	-2.485837	-0.914556	-3.494268
C	0.858851	-0.097548	-2.325902
O	1.764744	0.074859	-3.064189
C	-1.007016	-3.979186	-1.718595
C	1.744383	-3.176550	-1.583642
H	-0.939931	0.928616	3.835358
H	-4.953480	-2.546139	1.047360
H	2.473870	2.677972	1.847078
H	-3.486194	-1.742845	-0.798087
H	-1.627311	4.965034	-1.344888
H	-2.110786	-0.882740	3.825268
H	-2.506780	2.478977	-2.217689
H	1.826528	1.394058	-0.142606
H	0.037280	5.734246	0.302594
H	0.746825	4.542850	1.422494
H	1.545674	4.895896	-0.129606
H	-4.228427	-2.127129	3.426444
H	1.232617	2.240066	3.961026
H	-0.626901	-4.991605	-1.548959
H	-2.016124	-3.899451	-1.302298
H	-1.064474	-3.785158	-2.793371
H	0.580104	-4.370130	0.824082
H	0.833437	-2.694224	1.392934
H	-0.809889	-3.348101	1.266637
H	1.936566	-4.244764	-1.438845
H	1.799409	-2.939603	-2.649219
H	2.514757	-2.598839	-1.064511

II’

N	-2.622707	1.813112	-0.184478
C	-1.581351	2.649398	-0.158863
N	-2.018585	3.933234	-0.130766
C	-3.404202	3.903065	-0.138047
C	-3.764705	2.581113	-0.171865
C	-0.174386	2.040548	-0.227445
N	-0.337698	0.684101	0.327565
C	-0.276595	0.649032	1.698700
C	0.450118	1.549940	2.456069
C	1.189928	2.570056	1.763621
C	0.939434	2.810687	0.454779
C	-1.128775	-0.364015	2.351369

N	-2.173559	-0.806007	1.599585
C	-3.064108	-1.657133	2.152879
C	-2.951039	-2.124808	3.454795
C	-1.860856	-1.702259	4.224708
C	-0.947549	-0.817276	3.666726
Re	-2.086254	-0.304708	-0.572591
C	-3.815078	-0.959377	-0.987857
O	-4.920361	-1.334846	-1.182194
C	2.779857	-0.032219	-0.400198
C	-1.206781	5.149130	-0.158760
C	-1.793617	0.075670	-2.401820
O	-1.597731	0.291014	-3.547015
P	-1.033676	-2.446958	-0.868904
C	-2.095708	-3.900764	-1.269634
C	0.227438	-2.542085	-2.212447
C	-0.066928	-3.077412	0.576100
H	0.482201	1.477445	3.535219
H	-3.692457	-2.810000	3.848547
H	1.513188	3.530650	-0.120198
H	-3.879300	-1.971908	1.511575
H	-3.986067	4.810695	-0.103682
H	-0.090522	-0.475984	4.234495
H	-4.748471	2.137547	-0.188247
H	0.061537	1.932067	-1.294343
H	-1.862415	5.999875	0.027488
H	-0.437697	5.103730	0.613114
H	-0.732349	5.269065	-1.135939
H	-1.727086	-2.061531	5.238972
H	1.961848	3.125135	2.291367
H	2.251032	0.265490	-1.303360
H	2.936479	0.821944	0.254593
H	2.255674	-0.830772	0.117471
H	-1.483401	-4.798689	-1.403142
H	-2.815779	-4.070749	-0.464611
H	-2.651451	-3.701097	-2.189892
H	0.434517	-4.015898	0.319412
H	0.682000	-2.333284	0.863779
H	-0.728349	-3.245290	1.431076
H	0.649205	-3.550479	-2.268570
H	-0.234127	-2.288319	-3.170152
H	1.031932	-1.827308	-2.019670
O	4.070264	-0.623088	-0.809462
S	5.385934	0.284152	-0.871460
O	6.119007	-0.043004	-2.085239
O	5.104432	1.662130	-0.484357
C	6.347062	-0.497586	0.540029
F	7.540326	0.096403	0.624071
F	5.684418	-0.327697	1.686784
F	6.515160	-1.801180	0.311561

TSII ($\nu = 552i\ cm^{-1}$)

N	-2.783582	1.345058	-0.175611
C	-2.039978	2.441680	-0.321327
N	-2.824678	3.547168	-0.280271
C	-4.129270	3.121629	-0.090915
C	-4.090347	1.753100	-0.031442
C	-0.540639	2.270323	-0.536243
N	-0.199856	0.975549	0.103509
C	-0.060347	1.106187	1.492423
C	0.423257	2.246980	2.078870
C	0.764315	3.374805	1.241255
C	0.321128	3.411028	-0.032514
C	-0.546618	-0.022262	2.305926
N	-1.490479	-0.801971	1.711063
C	-2.095172	-1.764728	2.438758
C	-1.776392	-2.019743	3.765708
C	-0.769299	-1.258727	4.369361
C	-0.152431	-0.255560	3.632436
Re	-1.728424	-0.558568	-0.487543
C	-3.296938	-1.600026	-0.671656
O	-4.315241	-2.194475	-0.739762
C	1.891622	0.537292	-0.663866
C	-2.407339	4.942579	-0.414807
C	-1.654463	-0.307279	-2.362772
O	-1.568604	-0.165485	-3.531022
P	-0.350937	-2.538659	-0.747903
C	-1.275043	-4.098869	-1.092480
C	0.864264	-2.565898	-2.139735
C	0.708208	-3.057395	0.679206
H	0.531263	2.316588	3.154094
H	-2.296560	-2.803095	4.304110
H	0.560579	4.226219	-0.707238
H	-2.851449	-2.342784	1.921235
H	-4.944443	3.823743	-0.012447
H	0.632214	0.349374	4.070727
H	-4.895222	1.046443	0.098645
H	-0.379486	2.138944	-1.614258
H	-3.302545	5.556140	-0.511675
H	-1.838759	5.257948	0.461415
H	-1.792961	5.066732	-1.307430
H	-0.472973	-1.445757	5.395754
H	1.365659	4.181998	1.648644
H	1.383360	0.486624	-1.612299
H	2.169709	1.498591	-0.261157
H	1.838485	-0.308930	-0.000471
H	-0.585228	-4.944120	-1.181151
H	-1.991620	-4.295307	-0.290985
H	-1.830911	-3.987584	-2.027639
H	1.279319	-3.951870	0.411832
H	1.406992	-2.262216	0.954774
H	0.084723	-3.279260	1.549433
H	1.313711	-3.561254	-2.218140
H	0.349304	-2.328732	-3.075050
H	1.660966	-1.834197	-1.988696
O	3.485049	0.088013	-1.396773
S	4.780512	0.757900	-0.909634
O	5.855082	0.616935	-1.896222
O	4.544636	2.069386	-0.291563
C	5.274155	-0.355396	0.515676
F	6.395452	0.102260	1.088193
F	4.296620	-0.376256	1.437829
F	5.487957	-1.607344	0.089217

III’

N	-2.843295	1.268408	-0.437411
C	-2.114387	2.375839	-0.574239
N	-2.920181	3.464432	-0.651646
C	-4.223732	3.014615	-0.550936
C	-4.164369	1.652537	-0.418043
C	-0.607633	2.272586	-0.654344
N	-0.246222	1.023236	0.110774
C	-0.393315	1.282627	1.555035
C	-0.032196	2.488558	2.050347
C	0.418719	3.557332	1.174629
C	0.137425	3.484836	-0.137382
C	-1.000476	0.216495	2.359995
N	-1.795586	-0.666895	1.698722
C	-2.473235	-1.589953	2.414420
C	-2.379857	-1.694131	3.795500
C	-1.530289	-0.817912	4.476934
C	-0.835018	0.141168	3.751661
Re	-1.769258	-0.628328	-0.520143
C	-3.263785	-1.738403	-0.805796
O	-4.231546	-2.393220	-0.954919
C	1.171041	0.627489	-0.178298
C	-2.533312	4.867840	-0.811330
C	-1.511291	-0.509828	-2.396312
O	-1.321472	-0.440557	-3.555009
P	-0.331084	-2.593661	-0.503832
C	-1.156326	-4.159010	-1.022083
C	1.139591	-2.587485	-1.620909

C	0.415803	-3.088004	1.112800
H	-0.128932	2.689809	3.111224
H	-2.949419	-2.454324	4.316392
H	0.425053	4.258207	-0.840027
H	-3.102273	-2.261513	1.842982
H	-5.056818	3.698217	-0.582293
H	-0.160592	0.826723	4.249635
H	-4.963205	0.934746	-0.319221
H	-0.316175	2.072238	-1.691853
H	-3.431567	5.441749	-1.037088
H	-2.077520	5.243722	0.105842
H	-1.828861	4.972856	-1.637153
H	-1.408261	-0.885037	5.552344
H	0.936351	4.410013	1.600082
H	1.249542	0.390202	-1.238658
H	1.876953	1.421100	0.076128
H	1.409864	-0.258943	0.406490
H	-0.447100	-4.991641	-0.984505
H	-2.002465	-4.369959	-0.363400
H	-1.533930	-4.051325	-2.042238
H	1.070187	-3.953303	0.970363
H	0.999913	-2.263564	1.532060
H	-0.370383	-3.348196	1.826625
H	1.590113	-3.585466	-1.628270
H	0.821139	-2.331635	-2.635899
H	1.893819	-1.865388	-1.297530
O	3.978961	-0.616750	-0.929594
S	4.965111	0.487739	-0.837495
O	5.858583	0.637263	-2.010266
O	4.429947	1.754267	-0.282118
C	6.126900	-0.100536	0.511820
F	7.128261	0.778759	0.712707
F	5.470445	-0.250981	1.680399
F	6.679415	-1.288424	0.194389

III

N	-0.512775	1.499215	-0.815616
C	0.276817	2.343934	-0.099711
C	0.223079	3.731772	-0.302765
C	-0.608390	4.251831	-1.286278
C	-1.379522	3.373033	-2.052118
C	-1.308891	2.014421	-1.777630
C	1.239531	1.724140	0.819223
N	0.937075	0.353683	1.280184
C	2.207004	-0.466591	1.248873
C	3.338200	0.222837	1.981722
C	3.398269	1.565459	1.959750
C	2.405713	2.299570	1.191815
C	2.469911	-0.754470	-0.212049
N	3.633804	-1.009023	-0.860766
C	3.318836	-1.245921	-2.185839
C	1.958565	-1.132245	-2.297423
N	1.443555	-0.827269	-1.058765
C	4.989673	-1.054276	-0.310324
Re	-0.553334	-0.638545	-0.224036
C	-0.535609	-2.382133	0.523736
O	-0.538399	-3.448153	1.020564
C	0.397780	0.410452	2.675805
P	-2.678577	-0.234507	0.889464
C	-2.936772	-1.055347	2.522425
C	-1.472015	-1.304160	-1.728485
O	-1.989694	-1.707593	-2.705166
C	-3.115392	1.521559	1.270773
C	-4.177201	-0.794204	-0.026398
H	2.640700	3.306411	0.866710
H	-2.033766	3.728470	-2.838871
H	4.065697	-0.385581	2.506622
H	-1.904271	1.299149	-2.331092
H	4.084323	-1.472361	-2.910734
H	0.825887	4.389509	0.311342
H	1.324003	-1.253670	-3.160803
H	1.955721	-1.404256	1.756894
H	5.651346	-1.442526	-1.083749
H	5.316144	-0.055358	-0.017561
H	5.024623	-1.719984	0.552718
H	-0.656373	5.322134	-1.453375
H	4.195636	2.109341	2.454441
H	0.150832	-0.603785	2.992932
H	1.127808	0.847188	3.362121
H	-0.501613	1.023554	2.675619
H	-5.081221	-0.566031	0.546877
H	-4.229284	-0.297415	-0.998782
H	-4.119125	-1.872846	-0.194874
H	-4.075040	1.563376	1.795601
H	-2.349100	1.987134	1.896513
H	-3.192171	2.096001	0.344312
H	-3.944080	-0.843575	2.893836
H	-2.814370	-2.136126	2.411734
H	-2.207968	-0.698522	3.253271

III''

N	-3.121568	0.736371	0.185013
C	-2.900652	1.397704	1.352892
C	-3.959752	1.951835	2.086641
C	-5.254649	1.874441	1.588725
C	-5.467613	1.241049	0.361521
C	-4.380868	0.682634	-0.298622
C	-1.499815	1.558433	1.757741
N	-0.541428	0.558956	1.244702
C	0.738911	1.263432	0.851910
C	1.261280	2.139113	1.970357
C	0.375597	2.759899	2.768156
C	-1.044455	2.586504	2.510013
C	0.405953	2.002715	-0.425547
N	0.918995	3.149905	-0.940805
C	0.284510	3.373983	-2.148742
C	-0.603058	2.347002	-2.329070
N	-0.517326	1.502920	-1.246627
C	1.958348	4.019857	-0.384916
Re	-1.423915	-0.394624	-0.701968
C	-2.231004	-0.744424	-2.368687
O	-2.747505	-0.898225	-3.414158
C	-0.285235	-0.451204	2.318205
C	0.093892	-1.396674	-1.226666
O	1.037972	-2.039492	-1.515467
P	-2.394559	-2.445837	0.183174
C	-3.158726	-3.552636	-1.077124
C	-1.249439	-3.619224	1.034609
C	-3.758436	-2.277772	1.418477
N	3.719236	-0.813982	-0.186438
Si	4.734530	0.350868	-1.022617
C	5.266679	1.658959	0.236027
Si	4.015605	-1.673048	1.318956
C	2.887975	-3.188496	1.327727
C	5.831602	-2.180811	1.465464
C	3.629562	-0.573724	2.812919
C	6.298986	-0.414940	-1.765720
C	3.722916	1.096384	-2.431349
H	-1.740610	3.324303	2.893175
H	-6.456693	1.163921	-0.073831
H	2.334543	2.231941	2.100707
H	-4.505825	0.162300	-1.240438
H	0.526137	4.231322	-2.756077
H	-3.761099	2.426487	3.039760
H	-1.281797	2.156481	-3.145072
H	2.177253	4.787647	-1.125995
H	1.610263	4.488796	0.535795
H	-6.082785	2.295430	2.147597
H	0.693080	3.414250	3.572612

H	0.398019	-1.208869	1.931971
H	0.142265	0.018840	3.208095
H	-1.233163	-0.915926	2.585662
H	-3.564002	-4.451142	-0.601967
H	-3.961748	-3.025693	-1.599340
H	-2.402792	-3.841676	-1.812497
H	-4.086159	-3.267056	1.752277
H	-3.419796	-1.702223	2.285006
H	-4.607091	-1.753194	0.972230
H	-1.775714	-4.548034	1.276135
H	-0.401693	-3.844998	0.381639
H	-0.866529	-3.187108	1.961288
H	1.470467	0.473800	0.630033
H	3.001291	-1.244661	-0.767218
H	2.937787	-3.708436	2.291561
H	1.840195	-2.918870	1.143639
H	3.183326	-3.901059	0.549087
H	3.933808	-1.067272	3.744027
H	4.166558	0.380580	2.751908
H	2.560228	-0.349887	2.893079
H	6.004883	-2.739320	2.393426
H	6.144233	-2.809875	0.624824
H	6.484434	-1.299354	1.484318
H	6.913711	0.354308	-2.250186
H	6.913781	-0.901842	-1.000515
H	6.050339	-1.168473	-2.522351
H	4.327879	1.803168	-3.011344
H	3.390425	0.309224	-3.119093
H	2.831813	1.626394	-2.078532
H	5.859534	2.449574	-0.238424
H	4.403317	2.126864	0.724077
H	5.886232	1.213010	1.024210
H	2.867663	3.449505	-0.187615

TSIII (ν = 1462i cm^{-1})

N	-2.892936	0.856109	0.227566
C	-2.596741	1.481985	1.399864
C	-3.603411	2.147579	2.126983
C	-4.896572	2.199303	1.631383
C	-5.178570	1.594095	0.400133
C	-4.152814	0.933790	-0.257875
C	-1.198062	1.476833	1.819944
N	-0.276635	0.534801	1.149517
C	0.938526	1.302610	0.648760
C	1.502669	2.188044	1.680088
C	0.766149	2.576040	2.758589
C	-0.656534	2.362635	2.702534
C	0.548192	1.918814	-0.637150
N	0.976669	3.064950	-1.242463
C	0.282428	3.196682	-2.437833
C	-0.568209	2.131837	-2.517391
N	-0.397188	1.347951	-1.397056
C	1.992182	4.010327	-0.789498
Re	-1.332677	-0.453337	-0.667445
C	-2.240708	-0.876320	-2.267923
O	-2.805998	-1.079047	-3.280675
C	0.140152	-0.496752	2.139519
C	0.074901	-1.600498	-1.165678
O	0.979340	-2.325037	-1.413154
P	-2.433895	-2.322540	0.445111
C	-3.564972	-3.318919	-0.617339
C	-1.350890	-3.647266	1.140393
C	-3.537051	-1.933788	1.877119
N	3.036775	-0.554508	0.153073
Si	3.982416	-0.108773	-1.378360
C	5.259331	1.189564	-0.906627
Si	3.982235	-1.072509	1.666282
C	2.992354	-2.403837	2.559993
C	5.644044	-1.840974	1.222882
C	4.248501	0.455946	2.722107
C	4.726319	-1.687134	-2.084453
C	2.791238	0.573238	-2.658333
H	-1.298292	3.024493	3.274232
H	-6.170397	1.623369	-0.035019
H	2.529591	2.523975	1.573176
H	-4.324711	0.435825	-1.204734
H	0.469349	4.025584	-3.101432
H	-3.363369	2.614820	3.073629
H	-1.280893	1.873819	-3.284736
H	2.019312	4.835987	-1.500265
H	1.741369	4.395797	0.199363
H	-5.675500	2.706006	2.190788
H	1.192704	3.178942	3.552641
H	0.673692	-1.289743	1.620450
H	0.776243	-0.064831	2.918313
H	-0.750152	-0.911648	2.611644
H	-4.012404	-4.133043	-0.038968
H	-4.357116	-2.681799	-1.018928
H	-3.002734	-3.735961	-1.456609
H	-3.976900	-2.855616	2.269669
H	-2.977439	-1.438663	2.675273
H	-4.339927	-1.264510	1.556435
H	-1.961674	-4.457527	1.551168
H	-0.712610	-4.045046	0.346472
H	-0.710363	-3.248622	1.930420
H	2.104434	0.290792	0.392636
H	2.473448	-1.366281	-0.134037
H	3.679116	-2.948191	3.219291
H	2.187260	-2.005124	3.181920
H	2.562208	-3.134546	1.865584
H	4.920448	0.204275	3.551164
H	4.714763	1.265823	2.150035
H	3.312844	0.831673	3.147307
H	6.137975	-2.096025	2.168724
H	5.526784	-2.769506	0.655446
H	6.316153	-1.179321	0.670275
H	5.068907	-1.482517	-3.106050
H	5.575039	-2.075041	-1.516786
H	3.961111	-2.471464	-2.144866
H	3.253842	0.450892	-3.645128
H	1.835736	0.039860	-2.670596
H	2.585613	1.637276	-2.519048
H	5.796759	1.502168	-1.809819
H	4.784527	2.082726	-0.484908
H	6.002960	0.832381	-0.188358
H	2.978121	3.539396	-0.759929

IV’

N	-3.079390	-0.452506	-0.895565
C	-4.010845	-0.139660	0.065765
C	-5.389234	-0.363706	-0.208517
C	-5.783436	-0.874609	-1.428309
C	-4.816211	-1.147979	-2.414632
C	-3.489874	-0.916379	-2.097710
C	-3.543885	0.467350	1.277241
N	-2.097670	0.758743	1.363222
C	-2.006343	2.228053	1.176526
C	-2.797087	3.000589	2.013842
C	-3.897630	2.406601	2.685162
C	-4.355982	1.188578	2.174381
C	-1.269916	2.603493	0.003767
N	-0.962542	3.842774	-0.488293
C	-0.287432	3.663158	-1.686648
C	-0.203452	2.313803	-1.893208
N	-0.813782	1.662550	-0.845686
C	-1.276559	5.135624	0.105856
Re	-0.948390	-0.414449	-0.247917
C	-0.206799	-1.151776	-1.819436
O	0.229328	-1.572073	-2.832121
C	-1.536838	0.399574	2.704091
C	0.759804	-0.416277	0.531990

O	1.834792	-0.441612	1.043097
P	-1.362471	-2.692292	0.510857
C	-2.386861	-3.714262	-0.636891
C	0.149923	-3.734014	0.665656
C	-2.218528	-3.035537	2.115238
N	4.517995	0.594920	0.374933
Si	4.665156	0.582634	-1.511795
C	6.490138	0.598130	-1.932988
Si	5.607055	-0.436796	1.527400
C	4.689707	-0.404203	3.158335
C	5.714993	-2.153505	0.789680
C	7.256053	0.446832	1.598326
C	3.790383	-0.973008	-2.070490
C	3.799093	2.152638	-2.049527
H	-5.379299	0.876110	2.362139
H	-5.086817	-1.527077	-3.392484
H	-2.671928	4.076267	2.058579
H	-2.701269	-1.118783	-2.813715
H	0.067288	4.503324	-2.261898
H	-6.127422	-0.112819	0.543868
H	0.249867	1.772085	-2.708621
H	-0.820359	5.913481	-0.505844
H	-2.357723	5.295480	0.135584
H	-6.836637	-1.047551	-1.628253
H	-4.488817	2.980338	3.389505
H	-0.470793	0.628445	2.699202
H	-2.027667	0.963945	3.499941
H	-1.687184	-0.663627	2.874021
H	-2.423531	-4.747851	-0.278237
H	-3.403424	-3.317716	-0.695179
H	-1.951132	-3.693957	-1.639593
H	-2.467839	-4.099973	2.174316
H	-1.567447	-2.784706	2.956999
H	-3.135305	-2.443090	2.187470
H	-0.111287	-4.745680	0.991605
H	0.653817	-3.787340	-0.303736
H	0.838905	-3.286623	1.387329
H	4.608373	1.570608	0.679609
H	3.536297	0.349052	0.595808
H	5.215202	-1.026755	3.891307
H	4.627948	0.610769	3.565145
H	3.672774	-0.794412	3.047716
H	7.919422	-0.098465	2.279671
H	7.749979	0.497037	0.623461
H	7.151882	1.465218	1.986782
H	6.297155	-2.784419	1.471576
H	4.729027	-2.614097	0.671724
H	6.223264	-2.164697	-0.179930
H	3.798020	-1.008157	-3.165684
H	4.283611	-1.878945	-1.705309
H	2.745137	-0.991360	-1.747315
H	3.843689	2.234519	-3.141537
H	2.743197	2.160967	-1.759049
H	4.283309	3.042860	-1.632848
H	6.590616	0.632312	-3.024294
H	6.999948	1.477525	-1.526387
H	7.011584	-0.298147	-1.583177
H	-0.873426	5.199324	1.119114

IV

N	-3.806404	-0.912540	0.701175
C	-2.619719	-0.462539	0.189059
N	-1.601658	-1.036024	0.857760
C	-2.148627	-1.863438	1.811368
C	-3.512230	-1.800094	1.726588
C	-2.363294	0.507502	-0.839476
N	-0.917848	0.706157	-1.108374
C	-0.656161	2.101073	-0.702208
C	-1.478830	3.069785	-1.302204
C	-2.722620	2.693807	-1.822325
C	-3.226683	1.435185	-1.406003
C	0.164542	2.309554	0.453015
C	0.481095	3.604806	0.948390
C	1.262506	3.750638	2.076043
C	1.726549	2.605559	2.750736
C	1.386007	1.368644	2.231724
N	0.636992	1.203493	1.117667
Re	0.405338	-0.763122	0.084178
P	2.562299	-0.222085	-0.896741
C	2.934874	1.546622	-1.293286
C	-0.643297	0.503280	-2.566037
C	-5.149946	-0.539571	0.282775
C	1.258507	-1.839112	1.372618
O	1.745812	-2.498901	2.223304
C	0.249494	-2.274285	-1.041337
O	0.184787	-3.196251	-1.776615
C	4.019842	-0.643063	0.154492
C	2.981148	-1.052015	-2.490453
H	-1.216232	4.122378	-1.250316
H	2.330459	2.674750	3.646846
H	-4.298865	1.272881	-1.400273
H	1.723834	0.454154	2.705929
H	-4.293602	-2.295141	2.280939
H	0.105541	4.477325	0.427430
H	-1.529730	-2.449216	2.473059
H	-5.863204	-1.128269	0.858828
H	-5.329575	0.522801	0.469808
H	-5.293432	-0.748881	-0.780206
H	1.507820	4.742260	2.442272
H	-3.368178	3.414945	-2.310760
H	-0.873703	-0.532770	-2.816851
H	-1.250016	1.172310	-3.180785
H	0.412177	0.701072	-2.755498
H	4.953544	-0.363013	-0.343423
H	3.941230	-0.107979	1.106485
H	4.025789	-1.716257	0.363555
H	3.932663	1.627467	-1.737031
H	2.195726	1.948885	-1.991445
H	2.900638	2.144625	-0.378824
H	4.008931	-0.822178	-2.787978
H	2.864382	-2.133743	-2.381679
H	2.297674	-0.713382	-3.274292

TSIV (ν = 64i cm^{-1})

N	3.410672	-1.488936	-0.715352
C	2.304746	-0.959229	-0.117251
N	1.205713	-1.391016	-0.747410
C	1.610491	-2.228722	-1.760240
C	2.977256	-2.303066	-1.747229
C	2.228260	-0.000445	0.985465
C	1.474279	1.467277	0.852358
C	2.211953	2.303258	1.789880
C	3.191677	1.549965	2.437867
C	3.269504	0.233198	1.960044
N	0.750789	0.242433	1.371992
C	0.927836	1.993131	-0.411594
C	1.399890	3.198436	-0.958357
C	0.893301	3.658027	-2.166903
C	-0.064921	2.887798	-2.836446
C	-0.480159	1.700046	-2.252304
N	-0.012646	1.257769	-1.064610
Re	-0.712299	-0.632876	-0.091891
C	-1.152537	-2.165896	0.922115
O	-1.414637	-3.110628	1.578840
C	0.421734	0.224076	2.804634
C	4.814218	-1.279522	-0.369163
P	-2.683785	0.532050	0.673871
C	-3.533441	1.559478	-0.606594
C	-1.724205	-1.319314	-1.532669

O	-2.316907	-1.755384	-2.458377
C	-4.064572	-0.549797	1.243328
C	-2.543912	1.743677	2.063210
H	-4.444609	2.005413	-0.194095
H	-2.871885	2.358613	-0.951470
H	-3.797093	0.934467	-1.465512
H	-4.333748	-1.238095	0.436863
H	-4.941758	0.041528	1.525827
H	-3.733383	-1.141499	2.101722
H	-3.490408	2.278231	2.196309
H	-1.752086	2.466114	1.843711
H	-2.292149	1.226148	2.992500
H	-1.220316	1.068862	-2.729658
H	-0.481915	3.194352	-3.788491
H	1.241606	4.595498	-2.588341
H	2.162020	3.750745	-0.421632
H	4.967964	-0.240004	-0.081641
H	5.112144	-1.926197	0.459716
H	5.419548	-1.514827	-1.245294
H	0.900428	-2.707216	-2.416266
H	3.675096	-2.849374	-2.361963
H	-0.381243	-0.494148	2.971564
H	0.102122	1.220916	3.123791
H	1.297900	-0.060622	3.397842
H	2.070709	3.373943	1.873154
H	3.840753	1.945701	3.214270
H	4.031720	-0.491544	2.213890

V

N	3.410286	-1.487929	-0.719806
C	2.304999	-0.958115	-0.120753
N	1.205206	-1.392006	-0.747892
C	1.608769	-2.230435	-1.760620
C	2.975625	-2.303512	-1.749990
C	2.230180	0.001357	0.982112
C	1.478611	1.463319	0.851771
C	2.215359	2.300576	1.789810
C	3.196347	1.547264	2.435664
C	3.272772	0.230851	1.956818
N	0.750468	0.239985	1.372956
C	0.929355	1.991322	-0.410914
C	1.399197	3.198083	-0.955640
C	0.890527	3.659635	-2.162669
C	-0.067659	2.889671	-2.832338
C	-0.481464	1.700641	-2.249684
N	-0.011864	1.256734	-1.063465
Re	-0.712137	-0.633975	-0.090445
C	-1.155235	-2.166645	0.922811
O	-1.421345	-3.110661	1.578938
C	0.425719	0.222514	2.806015
C	4.814425	-1.278314	-0.375594
P	-2.682599	0.532906	0.674734
C	-3.548948	1.523574	-0.623784
C	-1.727415	-1.315968	-1.530927
O	-2.324813	-1.747056	-2.456002
C	-4.054017	-0.541628	1.282231
C	-2.534538	1.779501	2.032622
H	-4.465752	1.966534	-0.220432
H	-2.897404	2.323090	-0.986756
H	-3.803541	0.874914	-1.467848
H	-4.342174	-1.235379	0.486763
H	-4.922497	0.056850	1.576003
H	-3.708722	-1.126261	2.139668
H	-3.486008	2.303911	2.168138
H	-1.756814	2.506832	1.782483
H	-2.259789	1.289738	2.969786
H	-1.221566	1.069578	-2.727233
H	-0.485962	3.197638	-3.783348
H	1.237202	4.598300	-2.582678
H	2.160983	3.750545	-0.418701
H	4.972180	-0.235424	-0.101851
H	5.110105	-1.915244	0.461678
H	5.419027	-1.527258	-1.248623
H	0.897794	-2.710301	-2.414653
H	3.672838	-2.849789	-2.365415
H	-0.382672	-0.489116	2.974017
H	0.116670	1.221906	3.128153
H	1.301061	-0.070859	3.396218
H	2.070775	3.370494	1.876259
H	3.846096	1.942304	3.211889
H	4.032367	-0.496346	2.211138

TSV_1 ($\nu = 322i\ cm^{-1}$)

C	1.571288	3.097523	-0.986375
C	1.017709	1.937407	-0.430818
N	0.039511	1.252686	-1.075351
C	-0.394738	1.704831	-2.270471
C	0.104678	2.852813	-2.871301
C	1.107706	3.567815	-2.211207
C	1.511779	1.387050	0.863778
N	0.637979	0.264751	1.397441
C	0.406006	0.264052	2.829055
Re	-0.734230	-0.602350	-0.088951
C	-1.715334	-1.265024	-1.567871
O	-2.300738	-1.673565	-2.511620
N	1.194344	-1.393399	-0.702030
C	2.310724	-0.953546	-0.107879
N	3.394918	-1.570655	-0.663389
C	2.930970	-2.439528	-1.633949
C	1.568502	-2.315038	-1.652933
C	4.806724	-1.390617	-0.333081
C	2.315639	0.059279	0.944637
C	3.313159	0.241362	1.943274
C	3.200972	1.559677	2.453063
C	2.198063	2.276015	1.826339
C	-1.236698	-2.125181	0.910808
O	-1.539042	-3.067066	1.555482
P	-2.715792	0.559151	0.640389
C	-2.821451	1.206420	2.368296
C	-3.187654	2.063174	-0.324837
C	-4.258624	-0.446198	0.538767
H	-4.142839	2.461837	0.032122
H	-2.417144	2.832397	-0.220348
H	-3.277729	1.812682	-1.385638
H	-4.421414	-0.760472	-0.495949
H	-5.124854	0.126063	0.886187
H	-4.144567	-1.342277	1.154766
H	-3.799479	1.669689	2.533964
H	-2.038807	1.948972	2.541279
H	-2.685364	0.389430	3.082129
H	-1.173693	1.117767	-2.742298
H	-0.286486	3.170842	-3.830522
H	1.521078	4.471857	-2.646253
H	2.359340	3.609222	-0.446081
H	5.009492	-0.336425	-0.146390
H	5.076174	-1.966864	0.554975
H	5.397870	-1.737490	-1.181035
H	0.841297	-2.816149	-2.272253
H	3.607709	-3.050496	-2.210052
H	-0.384229	-0.453985	3.052340
H	0.117379	1.262245	3.183844
H	1.316107	-0.025773	3.378003
H	1.976963	3.329450	1.944734
H	3.830168	1.955502	3.245475
H	4.001624	-0.521407	2.281141

VI_1

C	1.726701	2.988572	-0.866804
C	1.064498	1.869244	-0.353189
N	0.062029	1.277874	-1.039696

C	-0.313093	1.788804	-2.231237
C	0.295114	2.903232	-2.794267
C	1.340633	3.513822	-2.096855
C	1.431682	1.204023	0.979090
N	0.230691	0.555497	1.495807
C	0.435276	-0.055910	2.800352
Re	-0.791023	-0.544982	-0.081331
C	-1.582040	-1.269898	-1.650450
O	-2.037211	-1.714423	-2.653603
N	1.220488	-1.377792	-0.509933
C	2.426094	-0.944222	-0.088764
N	3.398044	-1.804950	-0.539517
C	2.777836	-2.803704	-1.254983
C	1.436885	-2.527476	-1.231136
C	4.838463	-1.703013	-0.340711
C	2.626750	0.234620	0.713271
C	3.715712	0.669442	1.405970
C	3.373008	1.892751	2.137455
C	2.080281	2.206220	1.923885
C	-1.340945	-2.060930	0.899334
O	-1.667489	-3.005575	1.532899
P	-2.810668	0.574422	0.536616
C	-2.966665	1.002368	2.324311
C	-3.159749	2.201582	-0.266880
C	-4.379565	-0.343719	0.227415
H	-4.098485	2.623567	0.107130
H	-2.342297	2.897170	-0.057217
H	-3.231111	2.073873	-1.351030
H	-4.481459	-0.546349	-0.842585
H	-5.246226	0.229685	0.572096
H	-4.346104	-1.301080	0.756446
H	-3.875833	1.583714	2.509082
H	-2.085333	1.572569	2.627504
H	-3.000073	0.081835	2.913500
H	-1.128574	1.276733	-2.728544
H	-0.044721	3.279684	-3.752223
H	1.843778	4.385865	-2.501156
H	2.530087	3.438123	-0.295407
H	5.177881	-0.692151	-0.573444
H	5.104888	-1.946257	0.691174
H	5.326473	-2.408262	-1.013355
H	0.619800	-3.071450	-1.677049
H	3.340382	-3.602584	-1.711397
H	-0.510810	-0.491326	3.135579
H	0.751416	0.679659	3.559028
H	1.191966	-0.866464	2.809669
H	1.532266	3.046359	2.334415
H	4.071916	2.434140	2.765112
H	4.684538	0.190998	1.477369

VI’_1

C	1.223634	2.135521	0.314799
C	-0.133520	1.447363	0.240896
C	-1.027858	2.620474	-0.274547
C	-0.255906	3.731117	-0.435036
C	1.126406	3.426355	-0.059178
C	-0.621932	1.073305	1.646554
C	-0.201980	1.722785	2.811877
C	-0.735356	1.346978	4.042037
C	-1.683108	0.321239	4.082413
C	-2.056304	-0.281895	2.889219
N	-1.539862	0.083354	1.696960
Re	-2.080341	-0.807611	-0.257796
P	-0.748104	-2.743998	0.205065
C	-1.580029	-4.310142	0.711511
N	-0.179571	0.240200	-0.587238
C	0.203776	0.494342	-1.970925
C	-2.414443	2.389338	-0.588413
N	-2.994299	1.171867	-0.608581
C	-4.307913	1.357390	-0.966269
C	-4.539053	2.691483	-1.170243
N	-3.348453	3.338438	-0.931147
C	-3.165036	4.781597	-1.022205
C	-3.700961	-1.608948	0.322756
O	-4.722349	-2.065545	0.719960
C	-2.412071	-1.403334	-2.020510
O	-2.610518	-1.756183	-3.131925
C	0.299769	-3.314520	-1.202761
C	0.504284	-2.525299	1.544521
H	1.143148	-3.410153	1.628072
H	1.113525	-1.646981	1.312852
H	0.003866	-2.354463	2.502244
H	-2.182284	-4.132022	1.606615
H	-0.843892	-5.093426	0.919862
H	-2.247051	-4.646461	-0.086944
H	0.943276	-4.150552	-0.909441
H	0.918688	-2.482737	-1.548355
H	-0.346212	-3.626877	-2.028334
H	-2.788178	-1.081153	2.861485
H	-2.127210	-0.006762	5.014899
H	-0.416131	1.842711	4.952767
H	0.535376	2.513522	2.741689
H	-2.726801	5.166282	-0.099335
H	-2.518957	5.038199	-1.865056
H	-4.142443	5.238820	-1.171299
H	-4.988289	0.525690	-1.050191
H	-5.428135	3.232675	-1.453672
H	0.292265	-0.467311	-2.488937
H	1.175838	1.008222	-2.050245
H	-0.524326	1.101114	-2.543070
H	2.114840	1.622761	0.657520
H	1.933325	4.149894	-0.096817
H	-0.562742	4.693688	-0.822570
C	3.323824	-0.880681	-1.139403
H	3.132955	-1.653163	-0.396229
H	2.865625	-1.130842	-2.093160
H	2.982675	0.094364	-0.796871
O	4.760961	-0.827927	-1.444553
S	5.823182	-0.793099	-0.247105
C	5.966702	1.060566	0.043228
O	5.264951	-1.361205	0.973149
O	7.096269	-1.222047	-0.801726
F	6.880743	1.277336	0.990712
F	6.334546	1.671771	-1.081898
F	4.785456	1.545494	0.447157

TSVI_1 (ν = 497i cm^{-1})

C	1.020944	2.723233	-0.127446
C	-0.149540	1.752143	0.002361
C	-1.349227	2.701000	-0.295392
C	-0.877792	3.954959	-0.536403
C	0.580095	3.961159	-0.421973
C	-0.288850	1.267200	1.450616
C	0.186985	1.987311	2.551252
C	-0.039492	1.509505	3.839231
C	-0.740171	0.312428	4.002607
C	-1.180937	-0.354955	2.868696
N	-0.962032	0.106967	1.618386
Re	-1.651157	-0.900593	-0.227307
P	-0.207233	-2.821104	0.139006
C	-1.090931	-4.319968	0.766235
N	-0.092421	0.567417	-0.886552
C	-0.190956	0.953170	-2.299109
C	-2.685972	2.177030	-0.384121
N	-2.978487	0.861961	-0.418031
C	-4.340667	0.759690	-0.577842
C	-4.886112	2.013575	-0.633449
N	-3.845543	2.903150	-0.504911
C	-4.004967	4.352281	-0.473428
C	-3.064974	-1.884703	0.560043
O	-3.983700	-2.427173	1.071597

C	-2.119501	-1.586255	-1.928633
O	-2.403073	-1.998280	-2.997370
C	0.664135	-3.582259	-1.307058
C	1.141376	-2.703437	1.400539
H	1.630884	-3.676818	1.510283
H	1.892980	-1.961189	1.125724
H	0.710868	-2.413594	2.363319
H	-1.580258	-4.093660	1.716996
H	-0.385210	-5.144601	0.910201
H	-1.859625	-4.621748	0.049622
H	1.139822	-4.520220	-1.002746
H	1.428930	-2.923122	-1.722724
H	-0.069666	-3.791739	-2.090451
H	-1.729852	-1.286382	2.938194
H	-0.939886	-0.100732	4.984090
H	0.325260	2.060111	4.699590
H	0.727566	2.912197	2.389795
H	-3.438076	4.771684	0.358946
H	-3.667888	4.803000	-1.410177
H	-5.062916	4.573655	-0.333106
H	-4.832324	-0.198013	-0.635832
H	-5.904530	2.351276	-0.739520
H	0.063534	0.086036	-2.916784
H	0.506068	1.766224	-2.555625
H	-1.199100	1.278017	-2.597341
H	2.048710	2.436872	0.051735
H	1.192786	4.844731	-0.557439
H	-1.451955	4.827646	-0.816547
C	2.164989	-0.119682	-0.968879
H	2.126571	-0.050939	0.106505
H	1.680733	-0.956603	-1.438098
H	2.250984	0.790094	-1.539367
O	3.782683	-0.686068	-1.217900
S	4.849160	-0.609779	-0.101728
C	5.490520	1.142113	-0.304090
O	4.261646	-0.644419	1.242369
O	5.983447	-1.476475	-0.420951
F	6.441471	1.390227	0.604351
F	6.001913	1.320428	-1.527454
F	4.483495	2.016563	-0.126387

VII’_1

C	0.609331	3.044037	-0.261832
C	-0.384503	1.904804	-0.073570
C	-1.732733	2.606971	-0.369961
C	-1.484526	3.920532	-0.623282
C	-0.048031	4.179408	-0.559763
C	-0.406527	1.411359	1.381020
C	-0.027790	2.230844	2.446161
C	-0.154388	1.762769	3.751554
C	-0.658028	0.478657	3.961421
C	-1.011743	-0.284037	2.856875
N	-0.891102	0.165977	1.588413
Re	-1.492419	-1.007593	-0.191239
P	0.232068	-2.716105	0.148884
C	-0.498671	-4.386539	0.443829
N	-0.145141	0.721253	-0.997092
C	-0.486135	1.065778	-2.408148
C	-2.975459	1.880018	-0.377405
N	-3.062806	0.534751	-0.359973
C	-4.399154	0.221451	-0.447117
C	-5.130854	1.375909	-0.512889
N	-4.235390	2.417326	-0.464843
C	-4.616632	3.826521	-0.481411
C	-2.695058	-2.172259	0.669926
O	-3.479778	-2.852330	1.226628
C	-1.927367	-1.814555	-1.852697
O	-2.197122	-2.291856	-2.893200
C	1.420835	-3.126493	-1.210648
C	1.372481	-2.548653	1.591275
H	1.956099	-3.469050	1.695317
H	2.064306	-1.713851	1.446003
H	0.802595	-2.386484	2.510001
H	-1.143150	-4.375381	1.325780
H	0.297372	-5.123462	0.589125
H	-1.102519	-4.675112	-0.421315
H	1.855309	-4.112626	-1.017813
H	2.234082	-2.398374	-1.263411
H	0.894439	-3.153518	-2.168941
H	-1.409183	-1.285138	2.967848
H	-0.776127	0.069198	4.957342
H	0.136005	2.389157	4.587867
H	0.360229	3.221662	2.247228
H	-4.135191	4.356213	0.342314
H	-4.341484	4.291789	-1.430516
H	-5.697149	3.883391	-0.357102
H	-4.738663	-0.801106	-0.453123
H	-6.193859	1.547019	-0.578357
H	-0.156267	0.241483	-3.040961
H	0.027968	1.982915	-2.716697
H	-1.561500	1.185604	-2.521361
H	1.674898	2.933621	-0.112404
H	0.398011	5.152915	-0.722299
H	-2.213304	4.680521	-0.863196
C	1.302048	0.360973	-0.974949
H	1.634375	0.165452	0.044586
H	1.439082	-0.529528	-1.577999
H	1.911684	1.159677	-1.404934
O	4.125005	-0.788468	-1.333587
S	4.906274	-0.458381	-0.117428
C	5.289544	1.364389	-0.344361
O	4.123216	-0.476743	1.142564
O	6.250204	-1.076420	-0.039028
F	6.021153	1.841218	0.681088
F	5.979001	1.581300	-1.480940
F	4.151064	2.089507	-0.410283

VII_1

N	-3.354720	-1.914551	0.625209
C	-2.434383	-1.012252	0.155370
N	-1.190933	-1.482762	0.379874
C	-1.333447	-2.709353	0.986479
C	-2.664957	-2.982937	1.144563
C	-2.719264	0.215453	-0.540806
C	-1.617882	1.265845	-0.828865
C	-2.384572	2.307866	-1.632334
C	-3.673394	1.940558	-1.749082
C	-3.886942	0.660405	-1.078514
C	-1.115855	1.829856	0.508300
N	-0.093360	1.165584	1.095502
C	0.341520	1.576496	2.306754
C	-0.216811	2.654746	2.979556
C	-1.269035	3.344521	2.376969
C	-1.720923	2.925398	1.127326
Re	0.773462	-0.557032	0.004152
C	1.513892	-1.469800	1.476992
O	1.935929	-2.037419	2.417433
N	-0.437997	0.664693	-1.581921
C	-0.901944	-0.215375	-2.693925
C	-4.810597	-1.804216	0.629631
P	2.948037	0.531611	-0.270111
C	3.105357	2.343545	0.067720
C	1.361497	-1.952976	-1.140524
O	1.711871	-2.806323	-1.868866
C	0.358135	1.764583	-2.198110
C	3.798375	0.361232	-1.902817
C	4.256250	-0.131914	0.850997
H	1.157371	1.007157	2.733770
H	0.170625	2.939598	3.950268
H	-1.729531	4.194049	2.868821

H	-2.536131	3.439019	0.632887
H	-1.938577	3.223873	-1.993165
H	-4.455273	2.497394	-2.251113
H	-4.842997	0.158285	-1.052537
H	0.601129	2.514552	-1.445902
H	-0.196718	2.237656	-3.014360
H	1.273947	1.340325	-2.603189
H	-1.610914	0.317347	-3.337334
H	-1.363606	-1.115458	-2.294357
H	-0.028296	-0.500436	-3.280428
H	-0.481032	-3.303498	1.271716
H	-3.177790	-3.826145	1.579684
H	-5.214366	-1.945633	-0.375076
H	-5.112242	-0.829442	1.015917
H	-5.202858	-2.582675	1.282154
H	3.255356	0.880566	-2.695815
H	3.857560	-0.699018	-2.163379
H	4.809926	0.774858	-1.842879
H	3.976326	0.037656	1.893869
H	5.211463	0.362967	0.650607
H	4.368838	-1.208164	0.695621
H	2.687874	2.577107	1.050765
H	2.574577	2.932117	-0.683100
H	4.161663	2.628265	0.053541

TSV_2 (ν = 325i cm^{-1})

N	0.696638	-0.000251	1.419169
C	1.713703	1.407223	0.857096
C	2.422499	2.133094	1.843259
C	3.302142	1.241400	2.517722
C	3.213656	-0.040434	2.017374
C	2.154935	-0.063321	0.978852
C	2.266067	-0.952503	-0.201903
N	1.171234	-1.384419	-0.829827
C	1.580329	-2.163890	-1.885865
C	2.950143	-2.196199	-1.898516
N	3.376025	-1.419334	-0.836280
C	4.778644	-1.187151	-0.492748
C	1.046620	1.995518	-0.313507
N	0.070608	1.310210	-0.969044
C	-0.454877	1.845723	-2.092385
C	-0.080670	3.081146	-2.600458
C	0.894341	3.811028	-1.914359
C	1.464673	3.256726	-0.775470
C	0.499427	-0.077547	2.848894
H	0.871604	-2.631113	-2.551236
H	3.653440	-2.689556	-2.550899
H	5.116866	-1.906514	0.256426
H	5.374710	-1.300792	-1.398501
H	4.896441	-0.179588	-0.097469
H	3.841233	-0.885748	2.267800
H	3.959859	1.538672	3.329528
H	2.266365	3.178290	2.077649
H	-1.216606	1.251428	-2.582397
H	-0.548463	3.455612	-3.503341
H	1.213426	4.785169	-2.270872
H	2.250983	3.770570	-0.235913
C	-1.266491	-2.210551	0.753415
O	-1.576899	-3.194600	1.327626
H	0.803079	0.857600	3.351704
H	1.104063	-0.885103	3.286425
H	-0.555817	-0.257194	3.046781
P	-2.674607	0.533905	0.720846
C	-3.090773	2.138386	-0.099022
H	-3.236069	1.982885	-1.171862
H	-2.274111	2.852552	0.036853
H	-4.008067	2.556149	0.328224
C	-2.776438	1.029020	2.500526
H	-1.936574	1.676876	2.764707
H	-4.412547	-0.618842	-0.537700
H	-2.744138	0.141393	3.137358
H	-3.715518	1.561878	2.683222
C	-4.254957	-0.397404	0.521861
H	-5.102643	0.181278	0.902805
H	-4.189800	-1.345150	1.064532
Re	-0.731767	-0.626057	-0.120960
C	-1.741224	-1.116553	-1.647489
O	-2.340866	-1.416745	-2.623413

VI_2

C	1.726701	2.988572	-0.866804
C	1.064498	1.869244	-0.353189
N	0.062029	1.277874	-1.039696
C	-0.313093	1.788804	-2.231237
C	0.295114	2.903232	-2.794267
C	1.340633	3.513822	-2.096855
C	1.431682	1.204023	0.979090
N	0.230691	0.555497	1.495807
C	0.435276	-0.055910	2.800352
Re	-0.791023	-0.544982	-0.081331
C	-1.582040	-1.269898	-1.650450
O	-2.037211	-1.714423	-2.653603
N	1.220488	-1.377792	-0.509933
C	2.426094	-0.944222	-0.088764
N	3.398044	-1.804950	-0.539517
C	2.777836	-2.803704	-1.254983
C	1.436885	-2.527476	-1.231136
C	4.838463	-1.703013	-0.340711
C	2.626750	0.234620	0.713271
C	3.715712	0.669442	1.405970
C	3.373008	1.892751	2.137455
C	2.080281	2.206220	1.923885
C	-1.340945	-2.060930	0.899334
O	-1.667489	-3.005575	1.532899
P	-2.810668	0.574422	0.536616
C	-2.966665	1.002368	2.324311
C	-3.159749	2.201582	-0.266880
C	-4.379565	-0.343719	0.227415
H	-4.098485	2.623567	0.107130
H	-2.342297	2.897170	-0.057217
H	-3.231111	2.073873	-1.351030
H	-4.481459	-0.546349	-0.842585
H	-5.246226	0.229685	0.572096
H	-4.346104	-1.301080	0.756446
H	-3.875833	1.583714	2.509082
H	-2.085333	1.572569	2.627504
H	-3.000073	0.081835	2.913500
H	-1.128574	1.276733	-2.728544
H	-0.044721	3.279684	-3.752223
H	1.843778	4.385865	-2.501156
H	2.530087	3.438123	-0.295407
H	5.177881	-0.692151	-0.573444
H	5.104888	-1.946257	0.691174
H	5.326473	-2.408262	-1.013355
H	0.619800	-3.071450	-1.677049
H	3.340382	-3.602584	-1.711397
H	-0.510810	-0.491326	3.135579
H	0.751416	0.679659	3.559028
H	1.191966	-0.866464	2.809669
H	1.532266	3.046359	2.334415
H	4.071916	2.434140	2.765112
H	4.684538	0.190998	1.477369

VI'_2

N	0.420098	3.119389	1.703143
C	0.579788	2.039379	0.892576
N	0.792004	0.949811	1.624490
C	0.750908	1.323956	2.948457
C	0.521083	2.672663	3.010023
C	0.636287	1.926775	-0.614220

C	2.055851	2.298698	-1.098479
C	1.957322	3.317329	-1.995969
C	0.559043	3.732888	-2.126294
C	-0.216063	2.961441	-1.338671
C	3.272193	1.595750	-0.696205
C	4.526323	2.177859	-0.985668
C	5.703710	1.548422	-0.618951
C	5.620830	0.325396	0.052952
C	4.361919	-0.195595	0.314342
N	3.199772	0.397185	-0.044705
Re	1.253662	-0.833515	0.498687
P	1.901092	-2.560265	-1.065594
C	0.541463	-3.525034	-1.864296
N	0.299110	0.525998	-0.926607
C	0.162882	0.299389	-2.352717
C	0.226001	4.515881	1.317422
C	2.110631	-1.745751	1.925961
O	2.642513	-2.269587	2.847266
C	-0.321627	-1.768038	0.894330
O	-1.326739	-2.347313	1.129555
C	2.940990	-3.933758	-0.396896
C	2.917390	-2.044828	-2.521781
H	0.891972	0.610715	3.745151
H	0.413886	3.349683	3.842600
H	-0.761753	4.660694	0.876705
H	0.316204	5.126826	2.215232
H	0.988006	4.812518	0.595870
H	-1.292374	3.014612	-1.222413
H	0.222983	4.534739	-2.773683
H	2.766040	3.749453	-2.574805
H	4.263278	-1.139383	0.836264
H	6.505038	-0.212242	0.374135
H	6.663732	2.001734	-0.841996
H	4.556018	3.139243	-1.484202
H	1.063301	0.544149	-2.950275
H	-0.664586	0.884469	-2.789696
H	-0.059761	-0.756701	-2.526013
H	3.858674	-1.608645	-2.175985
H	2.382478	-1.290912	-3.103917
H	3.135380	-2.908195	-3.158657
H	-0.133746	-2.858572	-2.406758
H	3.864855	-3.526948	0.024905
H	-0.035423	-4.034459	-1.087126
H	0.946569	-4.266962	-2.559837
H	3.192281	-4.652272	-1.184422
H	2.399896	-4.448958	0.401494
C	-2.908841	-0.012788	-0.801886
H	-2.665669	1.027279	-1.007017
H	-2.247257	-0.677300	-1.347655
H	-2.886889	-0.238115	0.262332
O	-4.247488	-0.323616	-1.350948
S	-5.525813	0.467575	-0.812986
O	-5.151485	1.741179	-0.208696
O	-6.556139	0.366330	-1.834836
C	-6.065219	-0.657346	0.596450
F	-7.169451	-0.152918	1.151640
F	-6.319798	-1.881303	0.133753
F	-5.096366	-0.721200	1.516169

TSVI_2 (ν = 500i cm^{-1})

N	-0.383778	2.962582	1.595593
C	0.047599	1.957958	0.784496
N	0.335224	0.881221	1.509972
C	0.072051	1.183127	2.826478
C	-0.374272	2.475473	2.890886
C	0.332131	1.923595	-0.701315
C	1.760651	2.471306	-0.923214
C	1.681292	3.577063	-1.711627
C	0.286675	3.848559	-2.058422
C	-0.508044	2.921207	-1.490310
C	2.983069	1.844139	-0.420378
C	4.194799	2.566313	-0.488781
C	5.385557	1.997148	-0.072163
C	5.357747	0.691084	0.424353
C	4.136512	0.038422	0.488239
N	2.959227	0.575967	0.087200
Re	1.097739	-0.812454	0.433531
P	1.972162	-2.533851	-1.030944
C	0.724539	-3.471669	-2.022619
N	0.192336	0.505050	-1.127203
C	0.586063	0.348354	-2.526779
C	-0.759381	4.330070	1.235691
C	1.890654	-1.644112	1.941450
O	2.372182	-2.114804	2.913509
C	-0.417251	-1.880790	0.739479
O	-1.369336	-2.547776	0.950060
C	2.844156	-3.923707	-0.181634
C	3.217799	-2.090331	-2.324850
H	0.219251	0.463264	3.615746
H	-0.689927	3.086321	3.721578
H	-1.626112	4.326947	0.574694
H	-1.006626	4.861594	2.154392
H	0.071601	4.831495	0.737784
H	-1.587561	2.864245	-1.549650
H	-0.033475	4.678615	-2.677222
H	2.512469	4.167052	-2.079854
H	4.082094	-0.969629	0.878770
H	6.254618	0.187911	0.765383
H	6.312965	2.556940	-0.127408
H	4.180926	3.581233	-0.865204
H	1.662560	0.499912	-2.703842
H	0.047179	1.046517	-3.185983
H	0.339046	-0.665083	-2.854227
H	4.078091	-1.596566	-1.864780
H	2.778261	-1.407923	-3.055528
H	3.556726	-2.995250	-2.838959
H	0.158706	-2.794554	-2.668212
H	3.714095	-3.538707	0.358582
H	0.021143	-3.959329	-1.341478
H	1.214010	-4.231696	-2.640316
H	3.173332	-4.674962	-0.907055
H	2.170494	-4.388819	0.542984
C	-2.074956	-0.047076	-1.296265
H	-2.063501	0.713115	-2.059529
H	-1.635093	-1.002723	-1.527693
H	-2.097769	0.255217	-0.262190
O	-3.712330	-0.587819	-1.523538
S	-4.881233	0.216349	-0.910411
O	-4.473916	1.558649	-0.478894
O	-6.102145	0.051569	-1.701805
C	-5.188816	-0.734872	0.678471
F	-6.102841	-0.094575	1.421514
F	-5.638860	-1.967030	0.409506
F	-4.050384	-0.833371	1.379472

VII’_2

N	-1.347655	1.390653	2.059208
C	-0.478043	0.939048	1.116588
N	0.524179	0.277442	1.695085
C	0.296445	0.289695	3.051113
C	-0.861379	0.981995	3.285684
C	-0.461207	1.113092	-0.385760
C	0.403784	2.342986	-0.740158
C	-0.397063	3.234274	-1.383251
C	-1.745087	2.697534	-1.537526
C	-1.814729	1.475795	-0.978075
C	1.823850	2.538125	-0.439376
C	2.366158	3.836341	-0.555399
C	3.716371	4.062471	-0.353723
C	4.531749	2.973262	-0.035188
C	3.941847	1.726662	0.088047

N	2.618661	1.484690	-0.091333
Re	1.971723	-0.671875	0.416276
P	3.439402	-1.637847	-1.269420
C	2.762258	-3.105223	-2.162767
N	0.133256	-0.171822	-0.959488
C	0.458169	0.021165	-2.398829
C	-2.571645	2.184564	1.896692
C	3.309256	-0.861304	1.728677
O	4.117535	-0.953244	2.580056
C	1.408246	-2.416596	0.863281
O	1.069244	-3.504132	1.157512
C	5.029502	-2.311958	-0.619766
C	4.040864	-0.580486	-2.662865
H	0.963956	-0.197094	3.744168
H	-1.383354	1.218567	4.198964
H	-3.312532	1.650690	1.298862
H	-2.973452	2.362949	2.893492
H	-2.341632	3.141231	1.427385
H	-2.681011	0.828814	-0.924380
H	-2.556312	3.223070	-2.025809
H	-0.097523	4.203870	-1.760133
H	4.545549	0.866661	0.345738
H	5.597255	3.082621	0.128643
H	4.125079	5.063218	-0.440417
H	1.710836	4.663866	-0.792831
H	1.289175	0.716021	-2.505580
H	-0.412427	0.398594	-2.946407
H	0.742741	-0.944738	-2.815490
H	4.530318	0.316271	-2.273266
H	3.208982	-0.269965	-3.299541
H	4.757199	-1.143048	-3.269520
H	1.848116	-2.839235	-2.700169
H	5.610479	-1.509881	-0.155483
H	2.516016	-3.883923	-1.435194
H	3.494797	-3.494164	-2.876884
H	5.615852	-2.761824	-1.427009
H	4.820322	-3.067762	0.141503
C	-0.864515	-1.282485	-0.861629
H	-1.739180	-1.090172	-1.489382
H	-0.377722	-2.201647	-1.187858
H	-1.183484	-1.400907	0.173867
O	-4.308798	-0.757835	-1.099594
S	-5.526264	0.052396	-0.840511
O	-5.298182	1.238090	0.022257
O	-6.396106	0.279717	-2.016909
C	-6.560975	-1.073197	0.245332
F	-7.738537	-0.497555	0.556674
F	-6.822572	-2.239213	-0.376349
F	-5.921099	-1.353692	1.397902

VII_2

Re	-0.796843	-0.493802	-0.295481
P	-2.777444	0.297121	0.892083
N	1.091521	-1.066304	-1.150007
N	0.704463	-0.640291	1.511388
N	0.053743	1.645885	-0.499661
N	3.283474	-1.318808	-1.094419
C	1.485077	-1.619197	-2.346320
H	0.774503	-1.859660	-3.120942
O	-1.829704	-3.364201	-0.018178
C	-1.737424	-0.409468	-1.925350
C	1.265892	2.117205	-0.084326
C	4.690959	-1.281589	-0.689495
H	4.861281	-1.937347	0.165125
H	4.982254	-0.263217	-0.432862
H	5.286215	-1.626833	-1.533685
C	2.185360	-0.902745	-0.408221
C	2.151593	1.294195	0.744973
C	0.360635	0.214806	2.679758
H	0.165962	1.235827	2.358005
H	1.170545	0.208404	3.417599
H	-0.536955	-0.195182	3.141449
C	2.844830	-1.775797	-2.322481
H	3.534748	-2.170240	-3.051315
C	-1.434010	-2.261275	-0.123207
C	-0.387745	3.785120	-1.552524
H	-1.077639	4.391609	-2.126648
C	2.063073	-0.234220	0.943224
C	1.682586	3.422410	-0.419727
C	0.756554	-2.055019	1.990120
H	1.102116	-2.706353	1.187315
H	-0.248712	-2.355716	2.282139
H	1.418396	-2.149492	2.856059
C	-4.285579	0.444073	-0.160328
H	-4.110398	1.164260	-0.964878
H	-5.140866	0.772469	0.438050
H	-4.510509	-0.525155	-0.613028
C	3.867541	0.637309	2.160071
H	4.737913	0.749837	2.794008
C	3.225290	-0.512519	1.885503
H	3.481105	-1.499928	2.243603
C	-3.402321	-0.780910	2.254472
H	-2.682454	-0.822899	3.075834
H	-3.550768	-1.794462	1.872753
H	-4.352184	-0.396246	2.638837
C	3.223339	1.740795	1.452031
C	-0.735066	2.488033	-1.213227
H	-1.689598	2.086592	-1.525871
C	0.861176	4.263827	-1.150677
H	1.188410	5.265239	-1.407296
C	-2.742882	1.955657	1.708603
H	-1.998935	1.975979	2.508644
H	-3.725367	2.175952	2.137493
H	-2.490548	2.731884	0.981954
O	-2.289630	-0.377412	-2.964037
H	2.662386	3.762343	-0.110554
H	3.562953	2.766035	1.517419

TSIV_alt1 (ν = 606i cm^{-1})

N	-0.187490	3.182347	1.810475
C	0.085864	2.120411	0.993348
N	0.957861	1.303082	1.605951
C	1.246033	1.849925	2.837743
C	0.539575	3.009829	2.981291
C	-0.415844	1.809376	-0.334616
C	-0.940495	2.790991	-1.241523
C	-0.377514	2.953998	-2.491389
C	0.931630	2.416842	-2.677561
C	1.424417	1.465270	-1.815494
N	0.427239	0.750869	-0.985537
Re	1.485849	-0.548001	0.624761
C	-0.016340	-1.469325	1.309201
O	-0.957905	-2.050445	1.718119
C	-0.400243	-0.121876	-1.876136
C	2.831881	1.250083	-1.528180
N	3.139913	0.524446	-0.414802
C	4.438153	0.366814	-0.069623
C	5.489745	0.885273	-0.806272
C	5.188800	1.614163	-1.966497
C	3.864167	1.804790	-2.318543
C	-1.040661	4.333065	1.536755
P	2.054634	-2.483532	-0.736783
C	3.158556	-3.725950	0.065021
C	2.514528	-1.232530	2.046071
O	3.178537	-1.589637	2.953327
C	0.661458	-3.565702	-1.285615
C	2.952470	-2.165449	-2.323237
C	-2.570094	0.888385	0.298588
H	3.366604	-4.555132	-0.618553

H	4.098950	-3.254230	0.361490
H	2.669398	-4.110771	0.963920
H	0.112603	-3.917193	-0.407291
H	1.045876	-4.428844	-1.838613
H	-0.032108	-3.012467	-1.923047
H	3.095994	-3.101723	-2.871675
H	3.928853	-1.720480	-2.111017
H	2.388393	-1.467845	-2.948565
H	4.617901	-0.209760	0.829394
H	6.510325	0.721040	-0.483318
H	5.981030	2.032569	-2.578140
H	3.613613	2.378937	-3.201707
H	-0.730652	4.823252	0.612035
H	-2.089223	4.036404	1.456624
H	-0.937874	5.033106	2.365707
H	1.926026	1.369469	3.523517
H	0.480780	3.726387	3.785071
H	0.276626	-0.747995	-2.456300
H	-0.999288	0.475852	-2.568193
H	-1.055107	-0.751840	-1.274638
H	1.582037	2.883769	-3.410151
H	-0.799499	3.641797	-3.214917
H	-1.761203	3.429848	-0.930960
H	-2.686002	1.815541	0.835465
H	-2.817321	0.857076	-0.749210
H	-1.995637	0.093201	0.742026
O	-4.211336	0.279947	0.937189
S	-4.831313	-0.970692	0.302415
O	-3.905047	-1.663835	-0.605098
O	-5.597905	-1.756966	1.275230
C	-6.121402	-0.211609	-0.824768
F	-6.778530	-1.173991	-1.488411
F	-7.007366	0.504950	-0.119137
F	-5.528521	0.598573	-1.717678

V_alt1

N	3.602526	-0.741196	-1.111488
C	2.468388	-0.542406	-0.390885
N	1.399697	-0.634342	-1.185002
C	1.858221	-0.890936	-2.457253
C	3.224757	-0.957485	-2.423367
C	2.310294	-0.339807	1.108043
N	0.973590	0.403743	1.220667
C	1.154878	1.801339	0.764995
C	2.304254	2.463778	1.024921
C	3.413915	1.813076	1.690852
C	3.440014	0.472795	1.728868
C	0.062431	2.412171	-0.002231
C	-0.121830	3.802845	-0.059452
C	-1.077045	4.335083	-0.916144
C	-1.837667	3.464569	-1.701293
C	-1.636122	2.098174	-1.564505
N	-0.719857	1.570325	-0.724113
Re	-0.587124	-0.603457	-0.295271
P	-2.668402	-0.429828	0.952716
C	-3.315683	1.255889	1.345019
C	0.473842	0.453712	2.630600
C	4.988628	-0.742944	-0.640673
C	-1.555998	-1.122752	-1.818040
O	-2.129647	-1.407491	-2.805621
C	-0.545295	-2.429671	0.215602
O	-0.574999	-3.560974	0.538527
C	-4.128905	-1.208419	0.143973
C	-2.705768	-1.256044	2.604228
H	2.419336	3.488188	0.691779
H	-2.584825	3.830067	-2.395292
H	4.244758	-0.072416	2.210405
H	-2.223009	1.387743	-2.133363
H	3.956125	-1.135267	-3.195403
H	0.476179	4.451355	0.568785
H	1.182258	-1.015266	-3.288127
C	2.273753	-1.703632	1.825910
H	5.618588	-1.096776	-1.455823
H	5.294920	0.262720	-0.351064
H	5.099692	-1.418739	0.208557
H	-1.228643	5.407531	-0.968745
H	4.211786	2.413166	2.114447
H	0.219132	-0.551175	2.961243
H	1.222807	0.889847	3.298098
H	-0.421291	1.074630	2.649360
H	-5.018260	-1.086242	0.769395
H	-4.303049	-0.747775	-0.831590
H	-3.938098	-2.274486	-0.008227
H	-4.218910	1.170180	1.956286
H	-2.569743	1.841384	1.889396
H	-3.561942	1.788248	0.422818
H	-3.706747	-1.176768	3.040769
H	-2.448430	-2.312047	2.483835
H	-1.985755	-0.800782	3.287322
H	3.209955	-2.227205	1.611166
H	2.214916	-1.572566	2.909025
H	1.449675	-2.328854	1.490843

TSV_alt1 (ν = 2504i cm^{-1})

N	3.487983	-0.043570	-1.288198
C	2.440685	-0.688266	-0.675590
N	1.285364	-0.403381	-1.329235
C	1.623647	0.400990	-2.378531
C	2.986406	0.616584	-2.371883
C	2.563267	-1.559052	0.467418
C	1.993960	-2.937012	0.377454
Re	-0.731384	-0.435499	-0.409824
C	-1.022089	-2.305946	-0.446292
O	-1.229147	-3.464368	-0.441488
C	4.916473	-0.127893	-0.963688
C	3.444846	-1.274507	1.556774
C	3.428538	-0.072209	2.234297
C	2.507776	0.998646	1.997134
C	1.225359	0.862530	1.483402
C	0.580739	2.050311	0.857496
N	-0.316432	1.752270	-0.117680
C	-0.884377	2.752863	-0.811539
C	-0.623780	4.095131	-0.544700
C	0.264038	4.411819	0.482999
C	0.879723	3.377442	1.184781
N	0.490373	-0.305302	1.395022
C	0.398580	-1.118048	2.604302
P	-2.831827	-0.180250	0.755493
C	-3.668080	1.454665	0.576584
C	-1.658471	-0.249553	-2.061050
O	-2.213508	-0.085824	-3.088038
C	-4.148767	-1.334343	0.183979
C	-2.882719	-0.416940	2.584615
H	-4.643075	1.430282	1.074225
H	-3.059803	2.245982	1.021577
H	-3.814372	1.682715	-0.483116
H	-4.327766	-1.182995	-0.884158
H	-5.077357	-1.157735	0.735889
H	-3.825625	-2.367280	0.338037
H	-3.902762	-0.259984	2.949226
H	-2.211398	0.292706	3.074082
H	-2.563137	-1.429218	2.842717
H	-1.574134	2.459620	-1.593983
H	-1.116475	4.864653	-1.126582
H	0.476327	5.445487	0.733623
H	1.576139	3.597465	1.984014
H	5.149741	0.516298	-0.115999
H	5.184466	-1.156723	-0.724845
H	5.475204	0.197462	-1.840027
H	0.883229	0.788032	-3.060497

H	3.626024	1.154102	-3.054372
H	-0.297370	-1.938034	2.422145
H	0.020564	-0.527068	3.448682
H	1.362579	-1.549039	2.917595
H	2.872299	1.996550	2.219480
H	4.163558	0.061016	3.026033
H	4.013524	-2.101609	1.972833
H	2.846799	-3.632175	0.377193
H	1.400474	-3.184442	1.263829
H	1.409285	-3.101768	-0.524087

VI_alt1

Re	-0.771605	-0.383150	-0.330941
P	-3.014164	-0.275857	0.533883
C	-3.845954	1.365440	0.399959
C	-4.213632	-1.395259	-0.300637
C	-3.275549	-0.677304	2.315964
C	-1.484714	-0.328260	-2.098547
O	-1.930203	-0.263012	-3.183241
C	-0.848302	-2.280961	-0.360945
O	-0.871377	-3.455738	-0.347444
H	-4.869676	1.297047	0.779535
H	-3.299424	2.112864	0.981076
H	-3.874646	1.690680	-0.643651
H	-4.255681	-1.154342	-1.366680
H	-5.212743	-1.292177	0.134011
H	-3.874480	-2.429308	-0.194161
H	-4.318761	-0.493062	2.591566
H	-2.625662	-0.056889	2.939081
H	-3.041790	-1.729354	2.502753
N	-0.410614	1.770468	-0.007084
C	-0.900937	2.792335	-0.728251
C	-0.479637	4.107735	-0.547143
C	0.508478	4.372024	0.400187
C	1.033729	3.313806	1.141633
C	0.544995	2.022586	0.929832
H	-1.648361	2.535571	-1.469230
H	-0.914468	4.897956	-1.147211
H	0.869077	5.381479	0.559917
H	1.805616	3.492006	1.878969
N	1.344265	-0.174665	-1.093172
C	2.543421	-0.530310	-0.604248
N	3.533572	0.026931	-1.360178
C	2.943250	0.762720	-2.362757
C	1.594745	0.633788	-2.187552
C	4.972308	-0.184949	-1.203680
H	5.235323	-0.162019	-0.146821
H	5.264549	-1.146395	-1.633145
H	5.496339	0.615978	-1.723915
H	0.793483	1.072756	-2.759557
H	3.527468	1.304615	-3.089446
C	0.964601	0.847821	1.732353
N	0.216176	-0.212561	1.601553
C	2.197676	0.925855	2.529834
C	3.178808	-0.001872	2.464730
C	3.197302	-1.264446	1.714352
C	2.867898	-1.543571	0.436973
C	0.284460	-1.336652	2.532684
H	-0.741495	-1.596208	2.800212
H	0.851228	-1.094670	3.433900
H	0.732737	-2.206288	2.046244
H	2.365791	1.812666	3.132928
H	4.048664	0.161085	3.098618
H	3.542755	-2.111319	2.307737
C	2.905576	-2.962760	-0.082888
H	3.602410	-3.060285	-0.924000
H	3.206329	-3.660278	0.703041
H	1.915993	-3.255830	-0.452939

TSVI_alt1 ($\nu = 421i\ cm^{-1}$)

Re	-0.906419	-0.118768	-0.458280
P	-2.975955	0.018201	0.770346
C	-3.288338	1.671266	1.524745
C	-4.484754	-0.248243	-0.247109
C	-3.272695	-1.103666	2.203261
C	-1.698377	1.127401	-1.694938
O	-2.176764	1.898242	-2.436944
C	-1.599779	-1.584801	-1.452091
O	-2.026385	-2.511273	-2.033291
H	-4.260663	1.676715	2.028301
H	-2.508230	1.902857	2.254673
H	-3.282724	2.444007	0.749903
H	-4.517218	0.487612	-1.055501
H	-5.383565	-0.149636	0.368685
H	-4.454685	-1.248021	-0.689286
H	-4.193746	-0.802639	2.711742
H	-2.435661	-1.049010	2.903611
H	-3.379589	-2.135302	1.858778
N	0.167829	1.339665	0.799503
C	-0.008528	2.671431	0.857301
C	0.891248	3.516512	1.499425
C	2.030292	2.958638	2.078234
C	2.217159	1.577255	2.011920
C	1.261109	0.785053	1.376030
H	-0.894435	3.054372	0.364872
H	0.701919	4.582895	1.529495
H	2.764358	3.584728	2.573188
H	3.092771	1.117126	2.453108
N	1.139862	0.180522	-1.468439
C	2.324921	-0.193725	-0.966410
N	3.329391	0.517120	-1.545366
C	2.752766	1.378344	-2.460174
C	1.403565	1.162909	-2.400530
C	4.768934	0.398992	-1.326549
H	4.955445	-0.336342	-0.544629
H	5.257296	0.081629	-2.249496
H	5.170302	1.363963	-1.011913
H	0.615468	1.649322	-2.953021
H	3.350887	2.047325	-3.058537
C	1.330769	-0.743483	1.350212
N	0.080819	-1.266956	1.025711
C	2.159658	-1.223668	2.514483
C	3.408915	-1.655888	2.154837
C	3.563849	-1.797251	0.760629
C	2.434134	-1.354502	-0.043831
C	-0.209803	-2.601331	1.509413
H	-1.284732	-2.770454	1.463742
H	0.157368	-2.711292	2.543526
H	0.272710	-3.391149	0.922304
H	1.857320	-1.016270	3.536119
H	4.154838	-1.990880	2.867292
H	4.219080	-2.577622	0.375759
C	1.959611	-2.582476	-0.875373
H	2.641929	-2.702339	-1.724017
H	1.978895	-3.499830	-0.287838
H	0.957375	-2.417989	-1.273892

VII_alt1

C	-3.670537	0.650253	-1.493016
C	-2.421146	-0.072607	-1.092752
C	-1.345773	1.120525	-0.998259
C	-2.017267	2.203087	-1.807573
C	-3.367606	1.929208	-1.956022
C	-2.317718	-0.964242	0.110468
N	-1.133473	-1.429956	0.520707
C	-1.388942	-2.343607	1.522736
C	-2.739894	-2.430525	1.711200
N	-3.320636	-1.553611	0.814606
Re	0.869385	-0.542422	-0.005721

N	-0.112761	1.095454	1.125945
C	0.165625	1.518043	2.373561
C	-0.603216	2.477563	3.023081
C	-1.709059	3.005137	2.356909
C	-1.994100	2.567413	1.062923
C	-1.170998	1.613747	0.464568
C	-4.762813	-1.340393	0.686740
N	-0.025579	0.655015	-1.434435
C	0.330153	0.826392	-2.823563
P	2.920069	0.737357	-0.201606
C	4.108863	0.261700	-1.523478
C	1.665550	-1.507934	1.487343
O	2.148335	-2.095921	2.375269
C	1.517851	-1.866325	-1.204898
O	1.909877	-2.657266	-1.978030
C	2.651836	2.534934	-0.500638
C	3.974803	0.747528	1.307073
H	4.841072	1.398929	1.157224
H	3.393923	1.111022	2.159524
H	4.317323	-0.265676	1.530624
H	4.408393	-0.780118	-1.385271
H	4.994053	0.903174	-1.488068
H	3.634024	0.356292	-2.503965
H	3.607210	3.068210	-0.481679
H	1.995849	2.936992	0.275862
H	2.170580	2.684220	-1.470507
H	1.028820	1.067090	2.850629
H	-0.338282	2.795909	4.024097
H	-2.337039	3.750763	2.832169
H	-2.839864	2.970797	0.520373
H	-4.988799	-0.273960	0.682157
H	-5.144535	-1.805431	-0.224994
H	-5.246518	-1.798621	1.548327
H	-0.595536	-2.857839	2.040925
H	-3.335925	-3.015582	2.393201
H	-0.072073	1.774021	-3.219709
H	-0.044774	0.031271	-3.485347
H	1.420393	0.822566	-2.913710
H	-1.530691	3.136130	-2.064456
H	-4.083707	2.587814	-2.433266
H	-4.593144	0.135573	-1.737020
C	-2.173237	-1.037039	-2.322623
H	-3.000966	-1.748574	-2.384271
H	-2.134016	-0.469677	-3.253033
H	-1.243512	-1.593972	-2.185011

TSVII_alt1 (ν = 444i cm^{-1})

C	3.689453	0.735702	1.279876
C	2.538691	0.220249	0.688392
C	1.389886	1.234287	0.919305
C	2.056060	2.250654	1.829508
C	3.373603	1.961877	1.967707
C	2.375456	-0.855167	-0.280159
N	1.183376	-1.372502	-0.624853
C	1.435349	-2.425705	-1.471417
C	2.787492	-2.547877	-1.646208
N	3.376604	-1.555796	-0.895450
Re	-0.786240	-0.602414	-0.044289
N	-0.023684	1.212763	-1.074553
C	-0.454443	1.688157	-2.261115
C	0.077686	2.831826	-2.843947
C	1.097949	3.509386	-2.174338
C	1.543208	3.018203	-0.947794
C	0.961040	1.865854	-0.420288
C	4.816277	-1.302403	-0.851827
N	0.272287	0.452135	1.486118
C	-0.235569	0.773350	2.805399
P	-2.872803	0.572116	0.393182
C	-3.949130	-0.044599	1.758119
C	-1.617803	-1.400543	-1.578799
O	-2.101292	-1.887561	-2.531295
C	-1.327151	-2.078241	1.022201
O	-1.658427	-2.977295	1.703119
C	-2.724851	2.372868	0.776319
C	-4.055283	0.610553	-1.021556
H	-4.950385	1.179401	-0.751349
H	-3.580784	1.080757	-1.887703
H	-4.341612	-0.408320	-1.294422
H	-4.216245	-1.087014	1.564933
H	-4.860043	0.558133	1.824112
H	-3.421606	0.001566	2.714380
H	-3.721809	2.813308	0.878898
H	-2.191691	2.877716	-0.033880
H	-2.169684	2.528344	1.704329
H	-1.247730	1.125285	-2.739264
H	-0.305532	3.178588	-3.796192
H	1.538002	4.406154	-2.596916
H	2.328610	3.523466	-0.397639
H	5.009944	-0.235249	-0.967830
H	5.247458	-1.656499	0.086998
H	5.277931	-1.839408	-1.679355
H	0.637155	-3.012302	-1.896150
H	3.374130	-3.231337	-2.238886
H	0.086087	1.762065	3.152220
H	0.105639	0.046808	3.562561
H	-1.325291	0.721178	2.811173
H	1.544070	3.118915	2.222630
H	4.091554	2.535808	2.540188
H	4.636735	0.221350	1.378234
C	2.025775	-1.106158	2.429232
H	2.913476	-1.693205	2.226272
H	2.108856	-0.396433	3.242113
H	1.088302	-1.630787	2.309369

TSIV_alt2 (ν = 592i cm^{-1})

N	0.585156	3.193402	1.940082
C	0.565399	2.095242	1.131381
N	1.275206	1.111503	1.694067
C	1.747531	1.577034	2.898032
C	1.320289	2.866771	3.064281
C	-0.043646	1.923145	-0.195924
C	-1.179465	2.708479	-0.697354
C	-1.021099	2.850180	-2.111954
C	0.112304	2.237727	-2.596666
C	0.859977	1.583865	-1.512358
N	0.184105	0.515293	-0.704325
C	-3.460899	1.772537	-0.177765
Re	1.618403	-0.697218	0.556352
C	0.307463	-1.734338	1.442921
O	-0.527356	-2.377232	1.970957
C	-0.944471	-0.209661	-1.310539
C	2.335970	1.528094	-1.496095
N	2.935732	0.644796	-0.654207
C	4.283751	0.630939	-0.576491
C	5.094942	1.464081	-1.334909
C	4.486319	2.365800	-2.213454
C	3.098844	2.403780	-2.282518
C	-0.018374	4.507703	1.720900
P	2.114906	-2.541622	-0.937680
C	3.828449	-2.552774	-1.630987
C	2.944560	-1.391581	1.707356
O	3.782600	-1.773123	2.446442
C	2.044733	-4.193341	-0.120707
C	1.133093	-2.865756	-2.472372
H	3.984247	-3.453565	-2.232933
H	3.989535	-1.671413	-2.257184
H	4.557774	-2.536081	-0.815702
H	2.760425	-4.219266	0.705704
H	2.286770	-4.989079	-0.832513
H	1.043920	-4.361474	0.285574

H	1.545355	-3.738321	-2.989897
H	1.177121	-2.004769	-3.145211
H	0.087261	-3.067278	-2.225211
H	4.708417	-0.087696	0.114331
H	6.172066	1.405348	-1.234223
H	5.084542	3.031983	-2.825949
H	2.588001	3.104500	-2.931796
H	0.151712	4.829244	0.693997
H	-1.090886	4.481411	1.923814
H	0.457944	5.212786	2.402137
H	2.352394	0.961968	3.545421
H	1.464779	3.575394	3.864291
H	-0.539739	-0.946675	-2.001315
H	-1.618827	0.452150	-1.852824
H	-1.493659	-0.722914	-0.519637
H	0.440316	2.225387	-3.628584
H	-1.730204	3.383947	-2.738470
H	-1.579807	3.514346	-0.098534
H	-2.904757	1.017873	0.353437
H	-3.574926	2.740475	0.279612
H	-3.555735	1.694428	-1.249095
O	-5.119307	1.250789	0.305903
S	-5.865474	0.171868	-0.499551
C	-5.255807	-1.405515	0.310433
O	-7.304440	0.210406	-0.222900
O	-5.405669	0.076498	-1.891080
F	-5.767666	-2.470122	-0.321063
F	-3.913952	-1.467643	0.241977
F	-5.621433	-1.450577	1.597660

V_alt2

N	-0.293684	1.459334	-0.857771
C	0.718774	2.083386	-0.207091
C	1.116705	3.379345	-0.541045
C	0.455330	4.055987	-1.564272
C	-0.589226	3.413292	-2.229302
C	-0.927905	2.119838	-1.848220
C	1.408755	1.329336	0.875514
N	0.792862	0.016918	1.246603
C	2.165874	0.005011	0.654391
C	3.402555	0.046933	1.574607
C	3.203259	1.316921	2.375871
C	2.159898	2.040885	1.952674
C	2.184312	-0.694863	-0.652114
N	3.229691	-1.018338	-1.468430
C	2.703687	-1.664070	-2.569855
C	1.346810	-1.710365	-2.397713
N	1.038061	-1.105484	-1.204647
C	4.662611	-0.754131	-1.311100
Re	-0.825660	-0.618355	-0.225669
C	-1.130730	-2.345173	0.491858
O	-1.314549	-3.407082	0.962906
C	0.558919	-0.362627	2.657217
P	-2.719956	0.295207	0.985349
C	-3.361949	-0.671841	2.418239
C	-1.961997	-1.042207	-1.671794
O	-2.636722	-1.296098	-2.602708
C	-2.477339	1.971011	1.727073
C	-4.243740	0.560041	-0.017047
H	1.839349	2.998410	2.345888
H	-1.138347	3.897855	-3.027795
H	4.265617	0.232870	0.926708
H	-1.733645	1.582710	-2.333890
H	3.340845	-2.029387	-3.359370
H	1.933672	3.844272	-0.001861
H	0.585014	-2.130834	-3.034443
H	5.158969	-1.087191	-2.221349
H	4.845084	0.314124	-1.184625
H	5.068744	-1.311859	-0.465773
H	0.748955	5.064774	-1.832618
H	3.868154	1.586453	3.189349
H	0.519905	-1.451243	2.707322
H	1.324730	0.012261	3.335321
H	-0.404733	0.045783	2.959822
H	-5.029840	1.020415	0.589120
H	-4.012789	1.209193	-0.866630
H	-4.598887	-0.398099	-0.405530
H	-3.404396	2.312640	2.198058
H	-1.687164	1.948548	2.483978
H	-2.189744	2.684546	0.950140
H	-4.267208	-0.206432	2.820400
H	-3.589905	-1.691573	2.097447
H	-2.606213	-0.721958	3.207647
C	3.719744	-1.234205	2.364885
H	3.856731	-2.077669	1.681592
H	4.651056	-1.097942	2.922152
H	2.935440	-1.498775	3.075382

TSV_alt2 ($\nu = 418i\ cm^{-1}$)

C	-1.053407	1.718362	0.628308
N	-0.004941	1.076550	1.190607
C	0.377576	1.411122	2.438093
C	-0.257170	2.403988	3.176577
C	-1.343168	3.065749	2.606270
C	-1.750088	2.711071	1.318973
Re	0.863727	-0.566002	-0.030970
C	1.423234	-1.909223	-1.245009
O	1.761482	-2.730726	-2.019040
C	-1.397516	1.256199	-0.818259
C	-2.573415	0.211244	-0.691406
C	-3.741263	0.806372	-1.212116
C	-3.418411	2.144821	-1.719190
C	-2.108269	2.392801	-1.534873
C	-2.376970	-0.948565	0.163770
N	-1.152033	-1.443465	0.416144
C	-1.341838	-2.594133	1.132823
C	-2.688464	-2.804335	1.312135
N	-3.337066	-1.767401	0.697890
C	-2.851600	-0.448667	-2.351610
N	-0.256524	0.645606	-1.462141
C	0.311446	1.382081	-2.579561
C	-4.789839	-1.604516	0.649305
P	2.949687	0.605030	-0.346403
C	2.931334	2.427394	-0.043898
C	1.645902	-1.516542	1.415395
O	2.109919	-2.078649	2.346714
C	4.329484	0.072850	0.757043
C	3.770510	0.501196	-1.998194
H	3.937839	2.838968	-0.169758
H	2.253214	2.917641	-0.746691
H	2.585059	2.634174	0.971975
H	4.031241	0.181785	1.803014
H	5.222858	0.676502	0.569132
H	4.557428	-0.981294	0.576678
H	4.746169	0.997973	-1.967490
H	3.153869	0.975363	-2.764678
H	3.908543	-0.550538	-2.265046
H	1.216077	0.855976	2.840186
H	0.096435	2.642385	4.172486
H	-1.865462	3.845562	3.149405
H	-2.594023	3.206925	0.855575
H	-5.068017	-0.605731	0.988349
H	-5.161317	-1.777913	-0.363214
H	-5.234214	-2.339354	1.318603
H	-0.511537	-3.186498	1.481169
H	-3.229606	-3.583649	1.825404
H	0.831409	2.317479	-2.303297
H	-0.451096	1.652845	-3.328499
H	1.037885	0.735181	-3.076438
H	-1.588275	3.292680	-1.837972

H	-4.148130	2.786970	-2.194241
H	-4.752755	0.436494	-1.101430
H	-3.296768	-1.430450	-2.217330
H	-3.266650	0.090827	-3.201580
H	-1.740654	-0.435210	-2.441871

TSV_alt2_V_alt1 (ν = 740i cm^{-1})

C	3.396733	0.368110	1.572889
C	2.306521	-0.133852	0.773623
N	0.951131	0.248579	1.205040
C	1.072140	1.710336	1.043791
C	2.060977	2.311366	1.834591
C	3.146909	1.565935	2.300608
Re	-0.670993	-0.614012	-0.230407
N	-0.477941	1.535482	-0.791283
C	-1.145463	2.082770	-1.831030
C	-1.113867	3.434968	-2.130696
C	-0.341904	4.273814	-1.314412
C	0.375020	3.725321	-0.265052
C	0.303842	2.337525	-0.006242
C	0.586391	-0.105363	2.618499
C	2.359446	-0.624949	-0.580873
N	1.218653	-0.900234	-1.236345
C	1.560701	-1.314213	-2.490192
C	2.932580	-1.302009	-2.600893
N	3.431619	-0.858027	-1.402651
C	4.857698	-0.683477	-1.122711
P	-2.754131	-0.100192	0.919965
C	-3.967160	-1.488478	0.859796
C	-1.745576	-1.171184	-1.672530
O	-2.381526	-1.493023	-2.610479
C	-0.705517	-2.372286	0.478004
O	-0.715368	-3.447690	0.956009
C	-2.829253	0.343598	2.713413
C	-3.725815	1.285403	0.183863
C	3.103268	-1.466900	2.122089
H	-4.684539	1.389994	0.701619
H	-3.169744	2.222724	0.267956
H	-3.909724	1.086997	-0.875838
H	-4.171285	-1.755678	-0.180556
H	-4.903005	-1.198940	1.347709
H	-3.551421	-2.364009	1.366348
H	-3.876460	0.475108	3.002979
H	-2.295451	1.278498	2.904424
H	-2.390286	-0.449989	3.323153
H	-1.731976	1.392843	-2.425721
H	-1.675535	3.816697	-2.974339
H	-0.291982	5.340207	-1.508012
H	1.002164	4.353628	0.355198
H	5.055392	0.340448	-0.803214
H	5.191730	-1.389279	-0.359534
H	5.406353	-0.876654	-2.043003
H	0.817889	-1.592208	-3.220764
H	3.585338	-1.568064	-3.417261
H	-0.299104	0.475302	2.856423
H	1.373632	0.154204	3.327970
H	0.351959	-1.165534	2.685600
H	2.044444	3.383085	2.009467
H	3.898654	1.983648	2.957347
H	4.418824	0.162635	1.285249
H	3.919819	-1.954591	1.599591
H	3.325576	-1.269118	3.167911
H	2.180073	-2.031700	2.023537

Table S10. CPCM-B3LYP/6-31+G(d)-LANL2DZ cartesian coordinates of the species involved in the reaction of the *cis,trans*-[Re(CO)$_2$(*N*-MeIm)(phen)(PMe$_3$)]OTf compound with KN(SiMe$_3$)$_2$ and MeOTf in dichloromethane solution. The imaginary harmonic vibrational frequencies obtained for the TSs located are also included in parentheses.

I_p

Re	0.046281	0.044051	-0.072025
C	-0.266787	0.070283	1.793344
C	1.915129	0.088437	0.228060
P	0.076028	-2.376084	-0.051260
N	0.079441	0.025299	-2.283828
C	1.206313	0.025606	-3.021251
C	1.198694	0.021169	-4.411891
H	2.136772	0.029770	-2.466649
H	2.136019	0.027198	-4.955100
C	-0.030010	0.002948	-5.073004
C	-1.198650	0.001167	-4.317284
H	-0.079401	-0.009938	-6.156161
H	-2.161240	-0.016859	-4.811617
C	-1.121264	0.025618	-2.920225
C	-2.312216	0.034508	-2.046795
N	-2.066460	0.016531	-0.711362
C	-3.621216	0.051718	-2.540342
C	-3.106977	0.021389	0.142636
C	-4.431002	0.042734	-0.281754
H	-2.856111	0.012199	1.196181
H	-5.229830	0.048226	0.450079
C	-4.692667	0.057079	-1.652208
H	-3.803985	0.071888	-3.606797
H	-5.710711	0.074823	-2.024974
O	3.085446	0.112159	0.377637
O	-0.511928	0.077849	2.947744
C	-1.566137	-3.222671	-0.080617
C	0.954785	-3.224068	-1.437739
C	0.882776	-3.157067	1.411297
H	0.847872	-4.310072	-1.350118
H	0.550506	-2.898429	-2.399579
H	2.017116	-2.967580	-1.404452
H	0.837281	-4.249190	1.346148
H	1.929777	-2.843186	1.457915
H	0.384147	-2.828632	2.327542
H	-1.440350	-4.309657	-0.061121
H	-2.158142	-2.912612	0.784774
H	-2.111591	-2.940848	-0.985413
N	-0.095332	2.221521	-0.253392
C	-0.856665	2.929946	-1.140095
C	0.608413	3.088789	0.572100
C	0.300035	4.370888	0.217916
N	-0.598859	4.253907	-0.831364
H	1.274763	2.733360	1.344900
H	0.629943	5.322746	0.608406
C	-1.202126	5.387998	-1.508880
H	-1.835977	5.003673	-2.308300
H	-1.814360	5.979331	-0.819528
H	-0.434873	6.039998	-1.939682

TSI_p ($\nu = 202i\ cm^{-1}$)

N	0.21649	1.30426	1.04751
C	-0.75198	1.99355	0.36693
C	-1.23940	3.25824	0.79505
C	-0.63356	3.83124	1.93797
C	0.35744	3.13393	2.60305
C	0.74661	1.86540	2.13521
C	-1.32816	1.34067	-0.76665
N	-0.76195	0.16662	-1.17685
C	-1.55401	-0.68180	-1.87892
C	-2.70214	-0.17710	-2.58730
C	-3.13682	1.09645	-2.35840
C	-2.48194	1.89081	-1.36330
C	-2.13766	-1.80930	0.00757
N	-3.09756	-2.44209	0.75275
C	-2.67898	-2.56763	2.06946
C	-1.43580	-1.99640	2.11360
N	-1.11738	-1.53206	0.85047
C	-4.37182	-2.91914	0.23904
Re	0.78802	-0.65039	0.13218
C	1.19551	-2.21787	-0.84253
O	1.44210	-3.18161	-1.47816
P	2.60679	0.46561	-0.96999
C	3.02434	2.19388	-0.46636
C	1.89321	-1.22154	1.56131
O	2.55286	-1.54384	2.48823
C	4.25073	-0.36427	-0.87880
C	2.37666	0.65417	-2.79149
H	0.83303	3.53654	3.48977
H	-3.20685	-0.83116	-3.28991
H	1.50190	1.28893	2.65637
H	-3.28663	-3.03191	2.83211
H	-0.75730	-1.89623	2.94860
H	-1.07196	-1.57533	-2.24573
H	-5.20512	-2.39490	0.71630
H	-4.39631	-2.73104	-0.83475
H	-4.48182	-3.99304	0.41828
H	-0.96513	4.80244	2.29259
H	-3.98893	1.50696	-2.89224
H	3.22665	1.17610	-3.24284
H	2.27724	-0.33571	-3.24690
H	1.45816	1.21479	-2.98561
H	5.00476	0.19985	-1.43752
H	4.55878	-0.44764	0.16704
H	4.17044	-1.37233	-1.29541
H	3.87058	2.56547	-1.05298
H	2.16144	2.84601	-0.62686
H	3.28267	2.22194	0.59549
C	-2.33427	3.85020	0.09041
C	-2.94413	3.17197	-0.93597
H	-2.69980	4.82124	0.40903
H	-3.80949	3.59784	-1.43575

II_p

N	-3.32797	-1.93088	0.67204
C	-2.22320	-1.37056	0.11888
N	-1.21748	-1.37269	1.00091
C	-1.69163	-1.95403	2.15486
C	-3.00305	-2.30518	1.96571

C	-1.94032	-0.81643	-1.28323
N	-0.83231	0.12403	-1.06932
C	-1.24399	1.36786	-0.68957
C	-2.43286	1.97845	-1.15641
C	-3.30232	1.14908	-1.97196
C	-3.09737	-0.18214	-2.03503
C	-0.46870	2.07953	0.28054
C	-0.76773	3.43389	0.61878
C	0.04919	4.04521	1.60308
C	1.03524	3.30878	2.22847
C	1.21748	1.95446	1.88146
N	0.49868	1.35541	0.93225
Re	0.75040	-0.73387	0.16583
P	2.56881	0.17060	-1.11811
C	2.14396	1.65829	-2.12652
C	-4.61625	-2.17327	0.02502
C	1.89048	-1.31223	1.56977
O	2.56030	-1.63869	2.48913
C	0.95020	-2.39781	-0.70795
O	1.10319	-3.41807	-1.28351
C	4.05728	0.75985	-0.19963
C	3.31533	-0.93931	-2.38708
H	1.66049	3.74493	2.99921
H	-3.72752	-0.82278	-2.64475
H	1.95556	1.34349	2.38845
H	-3.72985	-2.76222	2.61931
H	-1.07017	-2.07506	3.02828
H	-1.55695	-1.65579	-1.87709
H	-5.33589	-2.46348	0.79005
H	-4.96053	-1.26588	-0.47109
H	-4.52997	-2.97769	-0.70976
H	-0.12731	5.08242	1.87271
H	-4.11771	1.61564	-2.51944
H	4.79188	1.18827	-0.88936
H	3.76286	1.52078	0.52900
H	4.50899	-0.07721	0.33981
H	2.99507	1.95400	-2.74844
H	1.28255	1.43365	-2.76056
H	1.87519	2.48894	-1.46803
H	4.14485	-0.43963	-2.89747
H	3.67694	-1.85345	-1.90929
H	2.55318	-1.21674	-3.12067
C	-2.70504	3.32815	-0.83099
C	-1.88538	4.06337	0.00711
H	-2.10948	5.09826	0.24560
H	-3.59698	3.78841	-1.24984

II’$_p$

N	-2.84474	0.89966	-1.13066
C	-2.25529	2.09875	-1.08192
N	-3.11827	3.05887	-1.49707
C	-4.31222	2.43541	-1.82035
C	-4.12710	1.09685	-1.58971
C	-0.78614	2.15601	-0.64993
N	-0.61073	0.95074	0.17333
C	-0.97898	1.13246	1.47641
C	-0.79166	2.34327	2.18437
C	-0.31571	3.47412	1.40750
C	-0.31519	3.41356	0.06048
C	-1.64628	0.06461	2.15644
N	-2.08452	-0.97281	1.37192
C	-2.74819	-1.97567	1.94588
C	-3.00573	-2.01883	3.33120
C	-2.60127	-0.96913	4.13141
C	-1.92705	0.13638	3.55434
Re	-1.47692	-0.82241	-0.77419
C	-2.59825	-2.22611	-1.38716
O	-3.34861	-3.07183	-1.73483
C	2.67759	1.45793	-0.66080
C	-2.85458	4.49068	-1.62644
C	-0.74752	-0.62842	-2.50697
O	-0.26257	-0.52022	-3.57866
P	0.29577	-2.32643	-0.16886
C	-0.13868	-4.10188	0.07949
C	1.71844	-2.44332	-1.33715
C	1.14014	-1.93020	1.42702
H	-3.54008	-2.86765	3.74182
H	0.03430	4.24555	-0.54314
H	-3.09599	-2.76433	1.28834
H	-5.16460	2.99875	-2.16652
H	-4.81600	0.27724	-1.72177
H	-0.19134	2.02628	-1.56424
H	-3.77843	4.97830	-1.93630
H	-2.52750	4.90201	-0.67070
H	-2.08596	4.67070	-2.38083
H	-2.81234	-0.96695	5.19700
H	0.02808	4.36554	1.92678
H	0.74171	-4.68213	0.37430
H	-0.90152	-4.18370	0.85962
H	-0.55003	-4.50869	-0.84831
H	2.00553	-2.58263	1.58060
H	1.46817	-0.88700	1.42418
H	0.43770	-2.06299	2.25406
H	2.45746	-3.16263	-0.97038
H	1.35952	-2.76027	-2.32001
H	2.19526	-1.46465	-1.44729
O	3.95611	1.01198	-1.23683
S	5.31262	1.11511	-0.39233
O	6.40272	1.09357	-1.35374
O	5.21783	2.11858	0.66066
C	5.32757	-0.56071	0.46490
F	6.39391	-0.62321	1.26401
F	4.21558	-0.70306	1.19577
F	5.38939	-1.53745	-0.43997
C	-1.05811	2.39617	3.57337
C	-1.57911	1.31721	4.26448
H	-1.77715	1.37432	5.33013
H	-0.85254	3.32435	4.10105
H	2.81524	2.39411	-0.12240
H	2.04127	1.59901	-1.53062
H	2.27378	0.67996	-0.01569

TSII$_p$ (ν = 562i cm^{-1})

N	-2.89837	0.91801	-0.66976
C	-2.27977	2.02713	-1.08082
N	-3.18930	2.99525	-1.35586
C	-4.44402	2.46871	-1.10069
C	-4.24975	1.18002	-0.67728
C	-0.76297	1.99474	-1.21522
N	-0.31642	0.93812	-0.27897
C	-0.24519	1.42168	1.03156
C	0.24968	2.70050	1.33819
C	0.47016	3.60830	0.21931
C	-0.03787	3.31093	-0.99072
C	-0.72557	0.61028	2.09601
N	-1.46950	-0.49437	1.75161
C	-1.98273	-1.24455	2.72402
C	-1.77730	-0.97125	4.09196
C	-1.05611	0.14576	4.45918
C	-0.52804	0.99745	3.45682
Re	-1.62347	-0.87008	-0.43744
C	-3.04671	-2.11550	-0.33990
O	-3.98318	-2.82902	-0.25255
C	1.79756	0.53267	-0.95076
C	-2.93151	4.35300	-1.83369
C	-1.51498	-1.11672	-2.31182
O	-1.41269	-1.28329	-3.47525
P	-0.00934	-2.65290	-0.13276

C	-0.71421	-4.35213	0.02259
C	1.23069	-2.93434	-1.47507
C	1.06631	-2.59444	1.37261
H	-2.21207	-1.63197	4.83288
H	0.08326	3.98108	-1.83544
H	-2.58130	-2.09321	2.41359
H	-5.33742	3.05855	-1.23356
H	-4.97544	0.43645	-0.38707
H	-0.53599	1.63834	-2.22873
H	-3.89128	4.83028	-2.02792
H	-2.38819	4.92581	-1.08039
H	-2.35337	4.32683	-2.75913
H	-0.90604	0.39503	5.50538
H	1.00923	4.53619	0.39071
H	1.29581	0.14781	-1.82278
H	1.97607	1.59194	-0.85923
H	1.85117	-0.08092	-0.06857
H	0.08445	-5.08258	0.18690
H	-1.41769	-4.39113	0.85816
H	-1.25486	-4.60868	-0.89259
H	1.77400	-3.42942	1.35635
H	1.62681	-1.65678	1.42299
H	0.44765	-2.66903	2.27036
H	1.79915	-3.84652	-1.26688
H	0.71056	-3.04541	-2.43117
H	1.92957	-2.09923	-1.55741
O	3.42223	0.06530	-1.61357
S	4.69812	0.74224	-1.08722
O	5.80156	0.61368	-2.04324
O	4.43468	2.04850	-0.46972
C	5.15982	-0.38119	0.34147
F	6.25187	0.08653	0.95993
F	4.15072	-0.43308	1.22982
F	5.40881	-1.62294	-0.09457
C	0.45905	3.07258	2.69076
C	0.11855	2.23208	3.73092
H	0.28798	2.52228	4.76268
H	0.88934	4.04846	2.89798

III’$_{p}$

N	-2.93663	0.57796	-0.72993
C	-2.46199	1.69909	-1.27525
N	-3.47818	2.54872	-1.56616
C	-4.65196	1.92868	-1.18039
C	-4.30434	0.70950	-0.66085
C	-0.97618	1.83468	-1.51988
N	-0.32756	0.95771	-0.47940
C	-0.44051	1.63854	0.81544
C	-0.14457	2.99387	0.91471
C	0.01525	3.76393	-0.31875
C	-0.42421	3.24838	-1.47827
C	-0.89684	0.91852	1.93817
N	-1.41853	-0.34151	1.74840
C	-1.86386	-1.00606	2.81236
C	-1.82400	-0.48733	4.12220
C	-1.33802	0.78583	4.32913
C	-0.87576	1.54084	3.22433
Re	-1.51537	-1.03941	-0.35739
C	-2.77833	-2.40947	-0.08299
O	-3.60840	-3.22333	0.10766
C	1.11834	0.73780	-0.81754
C	-3.39673	3.87487	-2.18071
C	-1.42782	-1.49754	-2.19637
O	-1.34539	-1.78287	-3.33449
P	0.28070	-2.62303	0.09162
C	-0.26864	-4.36779	0.32680
C	1.57380	-2.82487	-1.20989
C	1.30620	-2.35238	1.60486
H	-2.19853	-1.09047	4.94024

H	-0.37237	3.80331	-2.40822
H	-2.28536	-1.98655	2.62425
H	-5.60857	2.41006	-1.30826
H	-4.92949	-0.07073	-0.25682
H	-0.72997	1.37778	-2.48554
H	-4.41271	4.22988	-2.34691
H	-2.87042	4.56607	-1.52148
H	-2.87785	3.81636	-3.13891
H	-1.32022	1.22485	5.32154
H	0.42719	4.76655	-0.26171
H	1.16967	0.20423	-1.76612
H	1.67078	1.67624	-0.89219
H	1.56942	0.12945	-0.03682
H	0.59239	-5.01855	0.50738
H	-0.95678	-4.43215	1.17374
H	-0.79690	-4.70437	-0.56915
H	2.01521	-3.17796	1.72074
H	1.86926	-1.41816	1.52738
H	0.66599	-2.30148	2.48918
H	2.25964	-3.62912	-0.92381
H	1.09619	-3.08216	-2.15994
H	2.15010	-1.90556	-1.34295
O	3.93951	-0.21399	-1.77062
S	4.87702	0.73816	-1.12650
O	6.21974	0.82270	-1.74697
O	4.27575	2.03548	-0.73311
C	5.21924	-0.08322	0.52369
F	6.06616	0.64906	1.27184
F	4.07672	-0.24006	1.22950
F	5.76544	-1.30280	0.35465
C	-0.11435	3.60460	2.19798
C	-0.43860	2.88852	3.32747
H	-0.40630	3.35571	4.30676
H	0.15473	4.65318	2.27002

III$_{p}$

N	-0.20029	1.45900	-0.83663
C	0.94122	1.91513	-0.21685
C	1.56340	3.14451	-0.59344
C	0.91885	3.93621	-1.57454
C	-0.24031	3.47583	-2.16034
C	-0.75444	2.22043	-1.77768
C	1.55441	1.10505	0.76095
N	0.85231	-0.08817	1.25219
C	1.82248	-1.24353	1.22494
C	3.11213	-0.90043	1.94850
C	3.54702	0.36973	1.98547
C	2.83278	1.39634	1.22527
C	1.95839	-1.60898	-0.23562
N	2.97090	-2.22913	-0.89079
C	2.57710	-2.37216	-2.20844
C	1.32338	-1.82920	-2.30866
N	0.94811	-1.36071	-1.07049
C	4.25134	-2.68763	-0.34733
Re	-0.89650	-0.55451	-0.21816
C	-1.39722	-2.23467	0.50995
O	-1.71765	-3.26065	0.98671
C	0.37074	0.14411	2.65126
P	-2.78834	0.45795	0.92921
C	-3.22833	-0.23459	2.58460
C	-1.99612	-0.86906	-1.71534
O	-2.62978	-1.05573	-2.68913
C	-2.72081	2.27100	1.28363
C	-4.40526	0.32061	0.05296
H	-0.75347	4.04512	-2.92569
H	3.64295	-1.70086	2.45148
H	-1.63803	1.81977	-2.26074
H	3.21881	-2.84019	-2.93811
H	0.67356	-1.74795	-3.16524

H	1.31981	-2.06329	1.75147
H	4.74194	-3.29207	-1.10885
H	4.88512	-1.83682	-0.09319
H	4.08653	-3.30132	0.53926
H	1.35908	4.88439	-1.86615
H	4.45842	0.64262	2.50759
H	-0.17708	-0.74189	2.97472
H	1.20170	0.33523	3.33487
H	-0.29388	1.00552	2.64875
H	-5.19800	0.79149	0.64274
H	-4.34225	0.80802	-0.92397
H	-4.64892	-0.73370	-0.10303
H	-3.64217	2.58431	1.78435
H	-1.86978	2.50956	1.92787
H	-2.61367	2.83216	0.35219
H	-4.14268	0.23854	2.95543
H	-3.38980	-1.31281	2.49775
H	-2.42667	-0.06205	3.30600
C	3.44260	2.62561	0.85321
C	2.80935	3.49293	-0.00709
H	3.27198	4.43349	-0.28771
H	4.42475	2.86218	1.24907

III''$_p$

N	3.08998	0.31250	0.09047
C	2.89227	1.36976	-0.76878
C	3.94959	2.26379	-1.12152
C	5.24505	1.98090	-0.62424
C	5.42815	0.89627	0.20589
C	4.31935	0.09890	0.55421
C	1.59065	1.61969	-1.24971
N	0.52601	0.63845	-0.99829
C	-0.67527	1.38832	-0.47129
C	-1.05726	2.53278	-1.39181
C	-0.11249	3.16029	-2.11121
C	1.29323	2.80611	-1.91187
C	-0.29814	1.77119	0.94260
N	-0.71035	2.80771	1.71617
C	-0.09668	2.66689	2.94767
C	0.68135	1.54217	2.87770
N	0.54637	0.99376	1.62347
C	-1.64234	3.88493	1.37225
Re	1.26520	-0.82700	0.65874
C	1.97157	-1.63588	2.20924
O	2.41710	-2.07985	3.20294
C	0.18113	-0.07114	-2.27028
C	-0.36863	-1.74117	0.93217
O	-1.38654	-2.32145	1.05865
P	2.04065	-2.71473	-0.67001
C	2.78727	-4.10107	0.28917
C	0.75851	-3.60838	-1.65402
C	3.34868	-2.40307	-1.93804
N	-3.84002	-0.61994	-0.03179
Si	-4.77315	0.34993	1.09743
C	-5.26600	1.95497	0.22409
Si	-4.20426	-1.03250	-1.70036
C	-3.24298	-2.60119	-2.13181
C	-6.05631	-1.32601	-1.95006
C	-3.70060	0.38270	-2.85219
C	-6.35271	-0.51910	1.67483
C	-3.69518	0.67384	2.61315
H	6.40015	0.65543	0.61839
H	-2.10764	2.79292	-1.46953
H	4.43545	-0.72875	1.24406
H	-0.26799	3.37291	3.74415
H	1.31277	1.09523	3.62921
H	-1.75046	4.52346	2.24763
H	-1.25329	4.47187	0.54027
H	6.07268	2.63112	-0.88944
H	-0.35686	3.97211	-2.78847
H	-0.59772	-0.80341	-2.05412
H	-0.17259	0.62408	-3.03561
H	1.07164	-0.57920	-2.63432
H	3.07326	-4.91726	-0.38123
H	3.67144	-3.74729	0.82590
H	2.06481	-4.47196	1.02091
H	3.61610	-3.34004	-2.43600
H	2.99768	-1.69145	-2.69073
H	4.23923	-1.98678	-1.46025
H	1.19718	-4.49310	-2.12629
H	-0.05802	-3.91693	-0.99515
H	0.34601	-2.96201	-2.43296
H	-1.50252	0.66514	-0.43590
H	-3.16165	-1.24217	0.40607
H	-3.37210	-2.84690	-3.19243
H	-2.16773	-2.49980	-1.94068
H	-3.60162	-3.45538	-1.54591
H	-4.02867	0.18674	-3.88005
H	-4.15982	1.32360	-2.52525
H	-2.61520	0.53416	-2.87097
H	-6.26954	-1.55699	-3.00099
H	-6.41627	-2.16139	-1.33883
H	-6.64360	-0.43922	-1.68256
H	-6.92783	0.11856	2.35765
H	-7.00057	-0.77675	0.82922
H	-6.11380	-1.44818	2.20604
H	-4.23635	1.26990	3.35726
H	-3.41576	-0.27291	3.09074
H	-2.77000	1.20517	2.36425
H	-5.84167	2.60524	0.89284
H	-4.38974	2.51391	-0.12522
H	-5.89309	1.74915	-0.65265
H	-2.61978	3.47469	1.11055
C	3.65003	3.40572	-1.91162
C	2.35107	3.68587	-2.26851
H	4.45695	4.07606	-2.18883
H	2.11250	4.59327	-2.81409

TSIII$_p$ (ν=1405i cm^{-1})

N	2.88814	0.48772	0.32543
C	2.61477	1.66290	-0.33527
C	3.60333	2.68594	-0.48882
C	4.89833	2.43051	0.01643
C	5.15084	1.24313	0.67565
C	4.11141	0.30594	0.82138
C	1.30690	1.88763	-0.81281
N	0.30452	0.81983	-0.76018
C	-0.90974	1.35976	-0.01571
C	-1.33507	2.67254	-0.54827
C	-0.50339	3.45655	-1.28374
C	0.91678	3.15423	-1.24967
C	-0.57282	1.26972	1.41893
N	-1.01131	2.01013	2.47812
C	-0.37836	1.53091	3.61914
C	0.44124	0.51634	3.21554
N	0.31158	0.35723	1.85207
C	-2.00105	3.08373	2.47399
Re	1.21250	-0.97284	0.39621
C	1.96631	-2.18581	1.63190
O	2.42124	-2.90757	2.44367
C	-0.05756	0.43158	-2.15266
C	-0.28187	-2.09834	0.17690
O	-1.22510	-2.77736	-0.04989
P	2.33158	-2.14726	-1.42381
C	3.42335	-3.54879	-0.92566
C	1.26073	-2.96107	-2.68779
C	3.48217	-1.15113	-2.47542
N	-3.17750	-0.30351	-0.42822

Si	-4.02341	-0.84393	1.13997
C	-5.36264	0.40830	1.56754
Si	-4.23974	0.24872	-1.84871
C	-3.24874	0.05168	-3.43475
C	-5.77206	-0.83956	-1.98786
C	-4.74363	2.03907	-1.57573
C	-4.69717	-2.57446	0.84408
C	-2.79332	-0.93423	2.55042
H	6.12497	1.02277	1.09482
H	-2.35175	3.00098	-0.35539
H	4.27802	-0.62235	1.35604
H	-0.57773	1.95222	4.59143
H	1.10027	-0.10759	3.79876
H	-2.09690	3.46229	3.49114
H	-1.68216	3.89642	1.81997
H	5.67352	3.18220	-0.09727
H	-0.83983	4.38260	-1.73868
H	-0.75812	-0.40209	-2.12068
H	-0.50740	1.26772	-2.69398
H	0.84564	0.12261	-2.67643
H	3.86262	-4.02618	-1.80731
H	4.22363	-3.18252	-0.27717
H	2.84003	-4.28582	-0.36775
H	3.90793	-1.77974	-3.26330
H	2.95613	-0.31104	-2.93702
H	4.29284	-0.74988	-1.86210
H	1.87739	-3.46101	-3.44119
H	0.62115	-3.69941	-2.19671
H	0.62060	-2.22562	-3.18147
H	-2.17792	0.44966	-0.24482
H	-2.69062	-1.15466	-0.73961
H	-3.93844	0.15066	-4.28139
H	-2.47579	0.81626	-3.54678
H	-2.77535	-0.93387	-3.51152
H	-5.57531	2.26678	-2.25293
H	-5.08573	2.23376	-0.55559
H	-3.93089	2.73314	-1.80766
H	-6.34723	-0.48208	-2.85097
H	-5.51458	-1.88718	-2.17578
H	-6.42976	-0.79753	-1.11517
H	-5.11669	-2.95057	1.78526
H	-5.48083	-2.62207	0.08392
H	-3.88595	-3.24995	0.54958
H	-3.27169	-1.49452	3.36355
H	-1.87510	-1.46004	2.27718
H	-2.52037	0.04801	2.94268
H	-5.81906	0.10076	2.51622
H	-4.94398	1.41021	1.71562
H	-6.16375	0.47747	0.82582
H	-2.97477	2.71239	2.14402
C	3.22714	3.91475	-1.10067
C	1.91705	4.15151	-1.44388
H	3.98292	4.68132	-1.24028
H	1.61801	5.11982	-1.83355

IV’$_p$

N	2.98631	0.29778	0.50268
C	2.88614	1.51090	-0.13897
C	4.00368	2.39981	-0.25472
C	5.22916	1.98179	0.29574
C	5.30957	0.75841	0.94716
C	4.16419	-0.04646	1.03330
C	1.64572	1.89776	-0.67569
N	0.51788	0.97296	-0.63952
C	-0.51152	1.68013	0.18048
C	-0.82974	2.98873	-0.27135
C	0.05316	3.68859	-1.08991
C	1.40687	3.21941	-1.10361
C	-0.49376	1.22516	1.54502
N	-1.04378	1.78172	2.66984
C	-0.63135	1.02615	3.76293
C	0.15947	0.02694	3.27234
N	0.23808	0.14859	1.90254
C	-1.88798	2.96444	2.75536
Re	1.17322	-1.00957	0.33603
C	1.75629	-2.42253	1.44717
O	2.11770	-3.26205	2.19034
C	0.01162	0.71247	-2.01903
C	-0.39771	-1.93861	-0.10638
O	-1.40348	-2.46178	-0.46493
P	2.34084	-2.01153	-1.55597
C	3.46635	-3.41140	-1.13827
C	1.29681	-2.75579	-2.88199
C	3.48742	-0.93078	-2.52642
N	-3.50138	-0.24482	-0.49973
Si	-4.40175	-0.90063	1.03873
C	-5.61914	0.41902	1.57686
Si	-4.44529	0.58453	-1.92051
C	-3.40371	0.33852	-3.45595
C	-6.10399	-0.26391	-2.11296
C	-4.57927	2.37258	-1.38419
C	-5.16556	-2.51061	0.46253
C	-3.09475	-1.20850	2.33519
H	6.23453	0.41688	1.39597
H	-1.72714	3.48362	0.08850
H	4.19717	-1.00575	1.53880
H	-0.95325	1.27041	4.76267
H	0.66236	-0.77096	3.79627
H	-2.06937	3.17455	3.80968
H	-1.38841	3.82333	2.30176
H	6.09813	2.62810	0.21889
H	-0.19143	4.67145	-1.47747
H	-0.79605	-0.01680	-1.96175
H	-0.35138	1.63018	-2.48924
H	0.82422	0.30602	-2.61990
H	3.97659	-3.77624	-2.03524
H	4.20962	-3.07418	-0.41003
H	2.89141	-4.22549	-0.68953
H	3.91967	-1.49869	-3.35609
H	2.95965	-0.05962	-2.92348
H	4.29473	-0.57491	-1.88055
H	1.92557	-3.20702	-3.65576
H	0.65203	-3.52226	-2.44363
H	0.66003	-1.99304	-3.33812
H	-2.97383	-1.05522	-0.85651
H	-3.96256	0.71723	-4.32002
H	-2.45440	0.87916	-3.41023
H	-3.19443	-0.72123	-3.63980
H	-5.12862	2.94162	-2.14262
H	-5.11638	2.47472	-0.43557
H	-3.59057	2.83099	-1.27208
H	-6.63288	0.23187	-2.93601
H	-5.99393	-1.31911	-2.38082
H	-6.73896	-0.19470	-1.22600
H	-5.57434	-3.04075	1.33005
H	-5.97229	-2.37848	-0.26276
H	-4.39620	-3.15238	0.01689
H	-3.54826	-1.80416	3.13684
H	-2.24438	-1.76967	1.93909
H	-2.71752	-0.28554	2.78234
H	-6.13490	0.06102	2.47613
H	-5.10445	1.34890	1.84301
H	-6.38364	0.64775	0.82920
H	-2.84780	2.79764	2.25735
C	3.80109	3.66542	-0.89116
C	2.54854	4.06608	-1.28567
H	4.65355	4.32784	-1.00558
H	2.39739	5.06416	-1.68685
H	-2.75398	0.40938	-0.20634

IV_p

N	-0.46452	1.11371	-1.15990
C	0.32891	2.04601	-0.53125
C	0.39536	3.40610	-0.98282
C	-0.40305	3.76936	-2.07967
C	-1.19561	2.81274	-2.70525
C	-1.18844	1.49947	-2.21739
C	1.10577	1.65019	0.56988
N	0.99310	0.29000	1.08541
C	2.34511	-0.28074	0.86008
C	3.41310	0.46084	1.38869
C	3.25228	1.82215	1.69107
C	2.14887	2.46916	1.06567
C	2.35629	-1.26602	-0.17916
N	3.39975	-1.97879	-0.70677
C	2.89961	-2.75569	-1.74559
C	1.56122	-2.49108	-1.82254
N	1.22731	-1.56971	-0.85451
C	4.79129	-1.95090	-0.28333
Re	-0.66091	-0.84025	-0.07702
C	-1.74912	-1.72046	-1.33623
O	-2.3748	-2.28045	-2.16722
C	0.66617	0.27850	2.54528
C	-0.82292	-2.31805	1.08793
O	-0.93821	-3.21288	1.84927
P	-2.62848	0.20816	0.90303
C	-4.16711	0.07972	-0.10857
C	-3.19627	-0.42363	2.54223
C	-2.58441	2.03433	1.21102
H	-1.81321	3.06080	-3.56023
H	4.40076	0.01754	1.46844
H	-1.79599	0.73059	-2.68250
H	3.53990	-3.41432	-2.31059
H	0.81891	-2.90043	-2.49003
H	5.33890	-2.69736	-0.85849
H	5.23250	-0.96705	-0.46501
H	-0.38249	4.79403	-2.43956
H	4.06247	2.40092	2.12109
H	0.60835	-0.76050	2.86992
H	1.42810	0.80448	3.12552
H	-0.29706	0.76557	2.69313
H	-5.00119	0.58968	0.38405
H	-3.99219	0.53642	-1.08732
H	-4.42162	-0.97211	-0.26284
H	-3.53255	2.35874	1.65191
H	-1.76830	2.29337	1.89084
H	-2.42746	2.57004	0.27038
H	-4.12565	0.07026	2.84340
H	-3.36086	-1.50310	2.48351
H	-2.43028	-0.23474	3.29962
H	4.87553	-2.19184	0.77905
C	1.28447	4.30258	-0.30460
C	2.13713	3.84922	0.67037
H	1.31246	5.34015	-0.62320
H	2.86924	4.52238	1.10899

$TSIV_p$ ($\nu = 75i\ cm^{-1}$)

N	-0.001194	0.933542	1.261765
C	-1.006920	1.629309	0.668386
C	-1.392500	2.933780	1.107003
C	-0.773932	3.411620	2.284815
C	0.184866	2.648077	2.931417
C	0.570922	1.423455	2.373486
C	-1.642279	1.044725	-0.509036
N	-0.800451	0.040198	-1.255059
C	-2.130309	-0.515984	-0.793876
C	-3.279443	-0.341369	-1.679406
C	-3.510018	0.990326	-1.957391
C	-2.649824	1.847497	-1.211916
C	-1.963704	-1.608650	0.160415
N	-2.913515	-2.396403	0.742296
C	-2.266042	-3.246182	1.623057
C	-0.934918	-2.932439	1.567307
N	-0.759776	-1.912580	0.660374
C	-4.355952	-2.394686	0.510939
Re	0.918836	-0.712381	0.023249
C	2.150673	-1.332576	1.312114
O	2.888315	-1.722588	2.148354
C	-0.605342	0.202399	-2.710838
C	1.577320	-1.982958	-1.209311
O	1.977814	-2.765225	-1.997140
P	2.485628	0.972040	-0.727942
C	3.787289	1.477508	0.478238
C	3.482998	0.592333	-2.231561
C	1.745853	2.612034	-1.155640
H	0.667938	2.996985	3.837154
H	-3.889803	-1.165952	-2.025739
H	1.367965	0.830721	2.805252
H	-2.809479	-3.980239	2.196723
H	-0.104432	-3.355593	2.109922
H	-4.848472	-2.757391	1.413668
H	-4.689830	-1.379993	0.297030
H	-1.052297	4.389446	2.667952
H	-4.309306	1.351667	-2.596759
H	-1.252884	-0.488302	-3.256401
H	-0.853310	1.222226	-3.021932
H	0.436488	-0.008094	-2.950431
H	4.401129	2.286246	0.069253
H	3.313096	1.815389	1.403873
H	4.424981	0.620394	0.711275
H	2.508388	3.277601	-1.572557
H	0.939121	2.487684	-1.882882
H	1.325528	3.070145	-0.255238
H	4.176002	1.409535	-2.455283
H	4.048523	-0.329777	-2.072703
H	2.816922	0.442177	-3.086132
H	-4.617438	-3.044085	-0.328156
C	-2.327117	3.704711	0.344270
C	-2.919244	3.164917	-0.782546
H	-2.578072	4.703050	0.688248
H	-3.676615	3.736259	-1.315582

V_p

N	-0.03191	0.92314	1.25354
C	-1.04705	1.60145	0.65827
C	-1.44977	2.90280	1.09188
C	-0.84035	3.38895	2.27258
C	0.12546	2.63894	2.92330
C	0.53085	1.41954	2.36780
C	-1.67262	0.99952	-0.52550
N	-0.78106	0.02389	-1.27022
C	-2.11205	-0.51467	-0.79778
C	-3.26761	-0.39149	-1.69712
C	-3.53436	0.93062	-1.97391
C	-2.68615	1.80457	-1.23136
C	-1.94261	-1.61052	0.16340
N	-2.89039	-2.39344	0.75323
C	-2.24043	-3.23527	1.63884
C	-0.90928	-2.92258	1.57632
N	-0.73799	-1.91083	0.65986
C	-4.33408	-2.39827	0.52503
Re	0.93010	-0.70177	0.01919
C	1.61504	-1.95521	-1.21723
O	2.03220	-2.72576	-2.00787
C	-0.57709	0.14894	-2.72121

P	2.48792	1.00393	-0.70438
C	3.47637	0.67053	-2.22557
C	2.16021	-1.31525	1.31390
O	2.89815	-1.70501	2.14976
C	1.74907	2.65596	-1.08573
C	3.79346	1.47932	0.51019
H	0.60135	2.99521	3.83004
H	1.33671	0.83956	2.80029
H	-2.78207	-3.96416	2.22070
H	-0.07699	-3.34047	2.12013
H	-4.67192	-1.38629	0.30588
H	-4.59385	-3.05377	-0.30969
H	-4.82252	-2.75848	1.43091
H	-1.13160	4.36394	2.65369
H	-4.34241	1.27422	-2.61222
H	-0.59555	-0.85066	-3.16421
H	-1.34306	0.76689	-3.19534
H	0.40083	0.59818	-2.89755
H	4.42051	2.28309	0.11145
H	3.31863	1.81722	1.43574
H	4.41756	0.61180	0.74136
H	2.52572	3.34602	-1.43079
H	0.98252	2.56297	-1.86038
H	1.27841	3.06727	-0.18803
H	4.16225	1.49804	-2.43261
H	4.04800	-0.25209	-2.09565
H	2.80518	0.53941	-3.07906
C	-2.38858	3.66431	0.32691
C	-2.97124	3.11547	-0.80260
H	-2.65186	4.65983	0.66967
H	-3.73513	3.67677	-1.33728
H	-3.84409	-1.23952	-2.04323

TSV_1$_p$ (ν = 210i cm^{-1})

N	-0.02217	0.86492	1.33692
C	-0.98104	1.61081	0.74934
C	-1.24902	2.94217	1.16112
C	-0.63265	3.37668	2.35144
C	0.24500	2.53927	3.02940
C	0.56277	1.30207	2.46980
C	-1.61203	1.03421	-0.48231
N	-0.58291	0.25505	-1.26689
C	-2.39746	-0.28837	-0.56412
C	-3.44853	-0.10438	-1.47413
C	-3.52269	1.25876	-1.84214
C	-2.53272	1.99674	-1.19911
C	-2.09385	-1.49141	0.18431
N	-3.00755	-2.41643	0.61641
C	-2.31823	-3.40276	1.29572
C	-0.99751	-3.04781	1.26833
N	-0.86663	-1.86431	0.57787
C	-4.45759	-2.40076	0.44953
Re	0.88196	-0.72543	0.01303
C	1.52928	-1.98490	-1.23332
O	1.92899	-2.77025	-2.01999
C	-0.60941	0.43270	-2.69294
P	2.54408	0.87651	-0.70048
C	3.22973	0.64982	-2.39931
C	2.03930	-1.45894	1.32858
O	2.74609	-1.90319	2.16630
C	1.96605	2.62918	-0.77673
C	4.08695	1.03251	0.29897
H	0.72531	2.85346	3.94902
H	1.31535	0.65453	2.90262
H	-2.83034	-4.24599	1.73146
H	-0.14187	-3.55494	1.68499
H	-4.89798	-3.00552	1.24225
H	-4.82745	-1.37836	0.52272
H	-4.74297	-2.81717	-0.51951
H	-0.83343	4.37991	2.71581
H	-4.30599	1.68869	-2.45798
H	-1.57229	0.12029	-3.13963
H	-0.46834	1.49104	-2.97450
H	0.18145	-0.17356	-3.13963
H	4.74800	1.79589	-0.12438
H	3.83602	1.30401	1.32797
H	4.61017	0.07238	0.31686
H	1.08350	2.69125	-1.41932
H	1.68934	2.98074	0.22145
H	2.75507	3.27317	-1.17821
H	3.96121	1.43236	-2.62585
H	3.71107	-0.32936	-2.47537
H	2.42081	0.69215	-3.13418
C	-2.04874	3.82208	0.33750
C	-2.62986	3.36406	-0.81476
H	-2.18268	4.84680	0.66857
H	-3.26961	4.02165	-1.39944
H	-4.08471	-0.89115	-1.85753

VI_1$_p$

N	-0.01071	0.83045	1.37070
C	-0.85040	1.66843	0.73558
C	-0.90809	3.03950	1.07474
C	-0.26984	3.44126	2.25915
C	0.43570	2.51052	3.01936
C	0.59070	1.22440	2.51326
C	-1.52608	1.11615	-0.50653
N	-0.38949	0.55626	-1.29946
C	-2.57052	-0.02922	-0.37867
C	-3.65144	0.27745	-1.16147
C	-3.55557	1.64528	-1.62015
C	-2.38803	2.19574	-1.18027
C	-2.27574	-1.31345	0.20281
N	-3.20566	-2.24753	0.59483
C	-2.52345	-3.33319	1.10171
C	-1.19089	-3.03756	1.00931
N	-1.04327	-1.78649	0.45546
C	-4.65977	-2.14108	0.56193
Re	0.81592	-0.76353	-0.00201
C	1.37686	-2.00468	-1.30289
O	1.71531	-2.78373	-2.12627
C	-0.71180	0.21697	-2.67396
P	2.65384	0.67657	-0.61196
C	3.04062	0.71447	-2.41760
C	1.81932	-1.69680	1.31241
O	2.42735	-2.27367	2.15114
C	2.44503	2.47992	-0.27252
C	4.31428	0.34047	0.11803
H	0.92041	2.79143	3.94713
H	1.23102	0.49279	2.99145
H	-3.04615	-4.19488	1.48598
H	-0.33589	-3.62623	1.30109
H	-5.06913	-2.86254	1.26880
H	-4.96636	-1.13553	0.85426
H	-5.04695	-2.35974	-0.43603
H	-0.31325	4.48275	2.56280
H	-4.34926	2.17960	-2.13090
H	-1.47174	-0.58037	-2.79464
H	-1.08104	1.08910	-3.23736
H	0.20360	-0.13440	-3.15982
H	5.05519	1.05003	-0.26492
H	4.26124	0.42748	1.20639
H	4.62795	-0.67725	-0.12841
H	1.54274	2.82694	-0.78243
H	2.32997	2.66269	0.79916
H	3.31351	3.03514	-0.64099
H	3.87404	1.39509	-2.62141
H	3.29918	-0.29025	-2.76317

H	2.15688	1.05292	-2.96465
C	-1.50068	4.00163	0.15190
C	-2.14959	3.59919	-0.97065
H	-1.40480	5.05572	0.39603
H	-2.61810	4.32726	-1.62765
H	-4.45561	-0.39408	-1.43336

VI'_1$_p$

N	-2.03866	0.09627	1.64552
C	-1.31340	1.22901	1.66082
C	-0.74966	1.71302	2.86333
C	-1.18350	1.12956	4.06465
C	-2.10181	0.08211	4.03940
C	-2.46413	-0.44694	2.80539
C	-0.93960	1.79612	0.30135
N	-0.34500	0.61907	-0.41001
C	-2.04401	2.37944	-0.63042
C	-1.60980	3.58130	-1.12152
C	-0.40851	3.99398	-0.43162
C	-0.04732	3.03741	0.47066
C	-3.15386	1.60654	-1.12518
N	-4.27156	2.10596	-1.75328
C	-5.08371	1.03997	-2.07518
C	-4.44533	-0.08778	-1.63728
N	-3.25540	0.26820	-1.04733
C	-4.61326	3.49948	-2.01485
Re	-1.76237	-1.08276	-0.24708
C	-1.42633	-1.92504	-1.89980
O	-1.22926	-2.44037	-2.94575
C	3.21392	1.26846	0.29544
P	0.09382	-2.28659	0.70857
C	1.54397	-2.48139	-0.41786
C	-3.01801	-2.43615	0.19003
O	-3.82034	-3.25437	0.49377
C	0.89375	-1.55142	2.20164
C	-0.19424	-4.03081	1.23569
H	-2.46812	-0.36963	4.95381
H	-3.06636	-1.34303	2.71567
H	-6.03394	1.18325	-2.56509
H	-4.75388	-1.11856	-1.69884
H	-5.68702	3.55979	-2.19332
H	-4.35801	4.11496	-1.15140
H	-4.08388	3.87311	-2.89492
H	-0.78181	1.48798	5.00766
H	0.05186	4.97116	-0.53103
H	2.79168	0.47216	-0.31450
H	3.26980	2.20542	-0.25558
H	2.64818	1.39147	1.21553
H	0.72957	-4.49182	1.60082
H	-0.95012	-4.05737	2.02583
H	-0.57226	-4.60312	0.38395
H	1.21439	-0.53304	1.96592
H	0.18153	-1.50733	3.02961
H	1.76060	-2.14740	2.50478
H	2.35759	-3.01707	0.08148
H	1.23960	-3.03371	-1.31151
H	1.90008	-1.49642	-0.72996
C	0.33180	2.69062	2.82968
C	0.71708	3.27573	1.66735
H	0.81198	2.94253	3.77057
H	1.49535	4.03522	1.66210
H	-2.06389	4.14581	-1.92519
O	4.55786	0.88434	0.75445
S	5.79592	0.95326	-0.26009
O	5.51155	1.83279	-1.38729
O	6.99902	1.08313	0.54555
C	5.79629	-0.80388	-0.93534
F	6.05535	-1.66930	0.04458
F	6.73599	-0.90059	-1.87746
F	4.60062	-1.07976	-1.46746
C	0.26082	0.93306	-1.69510
H	1.01461	1.73291	-1.61627
H	0.76777	0.03275	-2.05777
H	-0.45012	1.24552	-2.48340

TSVI_1$_p$ (ν = 516i cm^{-1})

N	-1.66803	0.09335	1.66868
C	-1.02621	1.27792	1.64788
C	-0.45825	1.81686	2.82566
C	-0.80190	1.21496	4.04772
C	-1.63566	0.10121	4.06482
C	-2.00828	-0.46396	2.84981
C	-0.76991	1.87733	0.27060
N	-0.12621	0.74877	-0.50697
C	-1.97416	2.40031	-0.56887
C	-1.68051	3.65256	-1.02811
C	-0.46155	4.13370	-0.41938
C	0.04124	3.18186	0.41445
C	-3.04565	1.55828	-1.02476
N	-4.22385	1.98179	-1.59282
C	-4.94982	0.86163	-1.93108
C	-4.20096	-0.22126	-1.56083
N	-3.02553	0.21523	-0.99295
C	-4.70838	3.34840	-1.75969
Re	-1.45339	-1.07591	-0.22539
C	-1.12861	-1.88352	-1.90248
O	-0.94247	-2.37013	-2.96057
C	2.11098	0.68716	0.17802
P	0.26503	-2.52429	0.74368
C	1.62663	-3.09834	-0.37257
C	-2.73469	-2.41196	0.16964
O	-3.57574	-3.20361	0.41968
C	1.20987	-2.02002	2.25751
C	-0.37634	-4.16212	1.31471
H	-1.93670	-0.36584	4.99502
H	-2.55528	-1.39748	2.79641
H	-5.92664	0.93856	-2.38187
H	-4.42536	-1.27120	-1.65572
H	-5.78998	3.31053	-1.89003
H	-4.47506	3.93806	-0.87213
H	-4.26054	3.81987	-2.63795
H	-0.39758	1.61694	4.97164
H	-0.09337	5.14951	-0.50352
H	2.05280	-0.22250	-0.39251
H	2.16072	1.62147	-0.35955
H	1.69745	0.69542	1.17035
H	0.44874	-4.78991	1.66605
H	-1.09018	-4.01876	2.13055
H	-0.89115	-4.66699	0.49344
H	1.93186	-1.23405	2.03117
H	0.52210	-1.65393	3.02482
H	1.75320	-2.88580	2.64939
H	2.27587	-3.80162	0.15903
H	1.18767	-3.60142	-1.23889
H	2.23384	-2.26620	-0.73652
C	0.53590	2.88167	2.75451
C	0.84330	3.47777	1.57708
H	1.02751	3.17297	3.67769
H	1.56633	4.28809	1.53491
H	-2.24883	4.21446	-1.75689
O	3.75364	0.55993	0.73105
S	4.93329	1.04912	-0.14148
O	4.53255	2.07520	-1.10963
O	6.12729	1.25410	0.67979
C	5.30443	-0.47654	-1.16824
F	5.64792	-1.50266	-0.37984
F	6.31249	-0.22051	-2.01042
F	4.22021	-0.82648	-1.88132

C	0.14503	1.07898	-1.91124
H	0.68009	2.03438	-2.02153
H	0.78323	0.28966	-2.32242
H	-0.75600	1.12957	-2.53892

VII’_1$_p$

N	-1.50450	0.17347	1.66925
C	-0.91060	1.37651	1.53498
C	-0.25271	1.99592	2.61979
C	-0.45259	1.44646	3.89731
C	-1.24100	0.31056	4.04785
C	-1.71003	-0.32911	2.9048
C	-0.81879	1.93695	0.11746
N	-0.14150	0.81386	-0.70351
C	-2.12363	2.33645	-0.62879
C	-1.95772	3.59162	-1.13360
C	-0.70639	4.16341	-0.68104
C	-0.06476	3.27804	0.12534
C	-3.20730	1.43250	-0.90118
N	-4.46665	1.78170	-1.32371
C	-5.17335	0.61954	-1.52912
C	-4.33163	-0.41539	-1.22735
N	-3.11654	0.09285	-0.82832
C	-5.03349	3.11828	-1.48091
Re	-1.43624	-1.11103	-0.16186
C	-1.23021	-2.00862	-1.81729
O	-1.10843	-2.54614	-2.85557
C	1.29214	0.71900	-0.29779
P	0.41685	-2.47087	0.72279
C	1.66063	-3.07571	-0.50327
C	-2.61387	-2.46222	0.40906
O	-3.38050	-3.27942	0.77050
C	1.50977	-1.83754	2.07620
C	-0.14304	-4.07567	1.44698
H	-1.43201	-0.11895	5.02368
H	-2.22874	-1.27856	2.95351
H	-6.20224	0.63556	-1.85045
H	-4.51993	-1.47609	-1.25484
H	-6.11879	3.03284	-1.43811
H	-4.69347	3.76465	-0.67133
H	-4.74864	3.55340	-2.44152
H	0.02834	1.90506	4.75573
H	-0.41077	5.19297	-0.84173
H	1.72874	-0.16532	-0.75546
H	1.85310	1.59659	-0.63278
H	1.37997	0.63976	0.78274
H	0.72095	-4.65961	1.77856
H	-0.80333	-3.89715	2.29966
H	-0.69790	-4.64690	0.69831
H	2.24845	-1.13200	1.68762
H	0.91609	-1.34647	2.85213
H	2.04501	-2.68193	2.52228
H	2.38548	-3.72702	-0.00464
H	1.15172	-3.63939	-1.29013
H	2.19777	-2.24396	-0.96534
C	0.69860	3.08074	2.39820
C	0.85829	3.63883	1.17603
H	1.27945	3.41801	3.25027
H	1.54977	4.46049	1.01952
H	-2.64590	4.11133	-1.78519
O	4.13917	0.16162	1.02342
S	5.03478	0.89931	0.09865
O	4.42518	2.09197	-0.53686
O	6.42795	1.07373	0.57049
C	5.22144	-0.29506	-1.33591
F	5.80986	-1.44088	-0.94153
F	5.97067	0.23754	-2.31974
F	4.01685	-0.61306	-1.86076
C	-0.14785	1.06613	-2.17309
H	0.28315	2.04617	-2.40513
H	0.46755	0.29323	-2.63523
H	-1.15577	0.99573	-2.57502

VII_1$_p$

C	-1.05092	1.58813	0.73221
N	-0.09569	0.83852	1.31951
C	0.48326	1.27834	2.45642
C	0.19660	2.52996	2.99277
C	-0.63444	3.38515	2.27759
C	-1.26528	2.93168	1.10708
Re	0.86880	-0.66332	-0.03217
C	1.53210	-1.83889	-1.36191
O	1.91610	-2.57509	-2.19300
C	-1.75988	0.96986	-0.47010
C	-2.65581	-0.28469	-0.25431
C	-3.86225	-0.05088	-0.84343
C	-3.93848	1.31018	-1.33143
C	-2.78124	1.96382	-1.05032
C	-2.18208	-1.53181	0.28130
N	-0.88456	-1.87237	0.38407
C	-0.84331	-3.16371	0.85658
C	-2.12131	-3.60819	1.05864
N	-2.96242	-2.58200	0.69819
N	-0.62287	0.56220	-1.43770
C	-0.02625	1.78884	-2.03734
C	-4.41705	-2.63975	0.81927
P	2.85949	0.73425	-0.38419
C	2.86258	2.52640	0.07967
C	1.85343	-1.60200	1.26877
O	2.43231	-2.19811	2.10102
C	4.33855	0.14969	0.55302
C	3.56160	0.78679	-2.09481
H	2.15699	3.09447	-0.53020
H	2.59023	2.64897	1.13119
H	3.86596	2.93592	-0.07494
H	4.14230	0.18154	1.62784
H	5.20030	0.78479	0.32567
H	4.56753	-0.88311	0.27757
H	4.51636	1.32224	-2.08781
H	2.88479	1.28806	-2.79007
H	3.72463	-0.23396	-2.45060
H	1.21653	0.61998	2.90568
H	0.67172	2.84409	3.91419
H	-0.78929	4.40926	2.60148
H	-4.79376	-1.69119	1.20352
H	-4.88141	-2.85764	-0.14576
H	-4.66760	-3.43379	1.52205
H	0.08996	-3.67533	1.02547
H	-2.50233	-4.54345	1.43725
H	0.26171	2.49391	-1.26001
H	-0.74070	2.27169	-2.71201
H	0.85217	1.49575	-2.60782
H	-4.83948	1.76949	-1.72040
H	-4.66654	-0.76419	-0.95144
C	-2.01047	3.84155	0.24182
C	-2.67142	3.39069	-0.84953
H	-2.01353	4.89329	0.50823
H	-3.25417	4.06317	-1.47176
C	-1.09190	-0.27288	-2.58166
H	-1.91994	0.21592	-3.10724
H	-0.25119	-0.38411	-3.26783
H	-1.39785	-1.25915	-2.24147

TSV_2$_p$ (ν = 255i cm^{-1})

N	-0.08288	0.92446	1.27409
C	-1.12828	1.58197	0.69668

C	-1.46521	2.92808	1.05734
C	-0.78940	3.47395	2.17439
C	0.17996	2.73757	2.83164
C	0.53283	1.47747	2.32880
C	-1.84936	0.95977	-0.39396
N	-0.70182	-0.07405	-1.30921
C	-2.05497	-0.50497	-0.77521
C	-3.21386	-0.46567	-1.71365
C	-3.60478	0.82831	-1.91726
C	-2.83484	1.73340	-1.10743
C	-1.90513	-1.62269	0.18777
N	-2.85474	-2.41473	0.75746
C	-2.20739	-3.27843	1.62214
C	-0.87411	-2.97024	1.56339
N	-0.70276	-1.93704	0.67150
C	-4.29913	-2.40965	0.52764
Re	0.94640	-0.69678	0.03245
C	1.68592	-1.95544	-1.15879
O	2.13549	-2.73830	-1.91992
C	-0.62126	0.10785	-2.74824
P	2.45640	1.02159	-0.74517
C	3.46098	0.64150	-2.24595
C	2.17379	-1.24083	1.36767
O	2.91311	-1.59224	2.22126
C	1.66815	2.62759	-1.21243
C	3.75250	1.59013	0.44040
H	0.70536	3.13409	3.69294
H	1.35344	0.91494	2.75761
H	-2.75126	-4.01723	2.18956
H	-0.04247	-3.40487	2.09505
H	-4.63072	-1.39522	0.31250
H	-4.56037	-3.05982	-0.31045
H	-4.79090	-2.77030	1.43143
H	-1.03385	4.48110	2.50016
H	-4.41366	1.13777	-2.57131
H	-1.03368	-0.75924	-3.28295
H	-1.17822	0.99672	-3.08418
H	0.42750	0.23700	-3.02194
H	4.36498	2.38077	-0.00483
H	3.27344	1.97149	1.34657
H	4.39225	0.74799	0.71866
H	2.43045	3.34255	-1.53855
H	0.95068	2.47279	-2.02256
H	1.13173	3.04093	-0.35360
H	4.10308	1.48842	-2.50814
H	4.08060	-0.23885	-2.05676
H	2.79893	0.41707	-3.08742
C	-2.41869	3.67255	0.29405
C	-3.06617	3.08427	-0.77713
H	-2.63083	4.69642	0.58516
H	-3.81563	3.64435	-1.33145
H	-3.67968	-1.34906	-2.13077

VI_2_p

N	0.28737	1.20110	-0.91840
C	1.54870	1.50423	-0.43902
C	2.22869	2.71541	-0.81856
C	1.59715	3.56760	-1.75382
C	0.36387	3.21942	-2.25877
C	-0.25127	2.03562	-1.80497
C	2.21446	0.64540	0.45539
N	0.36549	-0.64579	1.55126
C	1.73087	-0.69820	1.01527
C	2.84553	-1.00208	2.01920
C	3.79422	-0.04637	2.00425
C	3.43210	0.99577	1.02997
C	1.67756	-1.69133	-0.13201
N	2.63124	-2.47962	-0.69996
C	2.04420	-3.12442	-1.77669
C	0.74532	-2.69580	-1.83353
N	0.53758	-1.80338	-0.80785
C	4.03440	-2.64102	-0.31897
Re	-1.08120	-0.55840	-0.11822
C	-2.16014	-1.94651	0.53347
O	-2.84337	-2.81980	0.94722
C	0.28088	0.20788	2.72412
P	-2.61304	1.05896	0.82642
C	-3.53484	0.56169	2.34973
C	-2.06103	-0.59618	-1.74076
O	-2.62283	-0.63652	-2.78439
C	-1.94935	2.71418	1.32329
C	-4.00969	1.57970	-0.26500
H	-0.15146	3.83993	-2.98221
H	-1.23634	1.76902	-2.17157
H	2.60088	-3.81968	-2.38459
H	-0.03916	-2.95894	-2.52551
H	4.53597	-1.67294	-0.29408
H	4.11736	-3.11396	0.66092
H	4.51087	-3.27702	-1.06476
H	2.09304	4.48327	-2.06191
H	4.69231	-0.02128	2.61062
H	0.96388	-0.11809	3.52524
H	0.50758	1.27723	2.53607
H	-0.73453	0.15863	3.12691
H	-4.64380	2.31479	0.24146
H	-3.61220	2.01985	-1.18417
H	-4.61045	0.70762	-0.53643
H	-2.76529	3.35682	1.67019
H	-1.21409	2.60238	2.12405
H	-1.45921	3.19053	0.46922
H	-4.25815	1.33360	2.63253
H	-4.06286	-0.37771	2.16308
H	-2.83949	0.40471	3.17812
C	3.49975	3.02479	-0.25500
C	4.09437	2.19314	0.67151
H	3.98729	3.94379	-0.56649
H	5.05223	2.45065	1.11248
H	2.83490	-1.89110	2.63870

VI'_2_p

C	-1.35059	2.08064	-1.23267
C	-0.25362	1.31913	-0.48083
C	0.89031	2.33970	-0.53565
C	0.46805	3.49974	-1.17680
C	-0.92648	3.30451	-1.60193
C	-0.59876	1.07278	0.97772
N	0.02929	0.08527	1.61038
C	-0.39351	0.09173	2.91924
C	-1.29139	1.11313	3.07806
N	-1.41375	1.73337	1.84554
Re	1.43203	-1.03808	0.41480
N	2.66571	0.98463	0.42692
C	3.92308	0.93412	0.86235
C	4.83460	2.00659	0.79016
C	4.42937	3.18925	0.21200
C	3.10190	3.30995	-0.26038
C	2.22136	2.18087	-0.10598
C	-2.26437	2.89802	1.59815
C	0.33670	0.05322	-2.45322
P	2.99058	-1.98303	-1.17174
C	2.34476	-3.26624	-2.33660
C	2.45779	-1.70658	1.86362
O	3.09033	-2.07553	2.79593
C	0.44344	-2.62819	0.38793
O	-0.19523	-3.62515	0.35909
C	3.86132	-0.83657	-2.33434
C	4.42466	-2.89055	-0.44061
H	5.08428	-3.27428	-1.22561

H	4.99042	-2.22072	0.21284
H	4.05618	-3.72440	0.16317
H	1.89212	-4.07850	-1.76105
H	3.15888	-3.66650	-2.94992
H	1.57956	-2.84076	-2.99071
H	4.54162	-1.40124	-2.98035
H	4.43391	-0.09632	-1.76882
H	3.13275	-0.30777	-2.95375
H	4.24477	-0.01231	1.28234
H	5.83937	1.87319	1.17248
H	5.10856	4.03067	0.11132
H	-3.07986	2.64118	0.92054
H	-2.67825	3.21629	2.55470
H	-1.67796	3.70953	1.16760
H	-0.02765	-0.62439	3.63816
H	-1.85289	1.45259	3.93416
H	-1.50421	4.05042	-2.13606
H	-2.33496	1.66146	-1.40780
C	-2.65378	-1.44084	-0.85950
H	-2.45267	-0.84346	0.02657
H	-2.35033	-0.92170	-1.76375
H	-2.18441	-2.41781	-0.78012
C	1.33710	4.60276	-1.34930
C	2.62960	4.50359	-0.87758
H	0.99382	5.50781	-1.84072
H	3.32083	5.33495	-0.97780
N	0.11921	0.00429	-1.01850
H	1.16550	0.71501	-2.77764
H	0.56797	-0.95204	-2.81511
H	-0.56187	0.39571	-2.99506
S	-5.14714	-0.57094	-1.13334
O	-4.10214	-1.75814	-0.93070
O	-6.37196	-1.15549	-1.65532
O	-4.52001	0.59750	-1.74238
C	-5.50977	-0.10719	0.65443
F	-5.95953	-1.16969	1.32136
F	-6.43630	0.85393	0.67121
F	-4.39241	0.34580	1.23926

TSVI_2$_p$ (ν = 497i cm^{-1})

C	-1.06048	2.55507	-1.04943
C	-0.08392	1.58782	-0.37029
C	1.23010	2.36675	-0.49662
C	0.99555	3.59604	-1.10489
C	-0.43258	3.67933	-1.44130
C	-0.38816	1.38421	1.10276
N	0.11397	0.30873	1.70454
C	-0.23671	0.37111	3.03336
C	-0.96323	1.51398	3.23307
N	-1.05193	2.15374	2.00918
Re	1.20632	-1.05824	0.45235
N	2.77948	0.67729	0.32579
C	4.03552	0.37935	0.65662
C	5.12863	1.25445	0.50637
C	4.91430	2.50319	-0.03327
C	3.60193	2.88517	-0.39416
C	2.53294	1.94608	-0.16849
C	-1.74595	3.42688	1.80869
C	0.37809	0.28845	-2.36505
P	2.43252	-2.35950	-1.18888
C	1.43279	-3.38550	-2.35844
C	2.15513	-1.90444	1.85714
O	2.73050	-2.39507	2.76675
C	-0.07020	-2.43288	0.53651
O	-0.87393	-3.29835	0.58343
C	3.58260	-1.49022	-2.34886
C	3.54825	-3.65128	-0.48396
H	4.04593	-4.21425	-1.27985
H	4.30269	-3.18291	0.15420
H	2.96044	-4.33707	0.13199

H	0.78088	-4.04895	-1.78313
H	2.08898	-3.98796	-2.99533
H	0.80526	-2.75327	-2.99161
H	4.08349	-2.21697	-2.99656
H	4.33727	-0.93481	-1.78510
H	3.02643	-0.78260	-2.96851
H	4.19868	-0.61678	1.05072
H	6.11629	0.92305	0.80382
H	5.73464	3.19767	-0.18689
H	-2.60864	3.29401	1.15485
H	-2.08460	3.77521	2.78405
H	-1.07141	4.16434	1.37416
H	0.04880	-0.39770	3.73392
H	-1.42694	1.92722	4.11475
H	-0.88940	4.53031	-1.93400
H	-2.11805	2.34215	-1.16052
C	-2.19053	-0.56345	-1.12645
H	-2.22536	-0.25924	-0.09290
H	-2.20348	0.18992	-1.89513
H	-1.70257	-1.49838	-1.34868
C	2.04582	4.51165	-1.34346
C	3.32503	4.15704	-0.97137
H	1.84867	5.47220	-1.80861
H	4.15475	4.83949	-1.12834
N	0.01419	0.22666	-0.95183
H	1.40160	0.65494	-2.54673
H	0.30314	-0.71535	-2.7911
H	-0.30262	0.93777	-2.93803
S	-4.97130	-0.15571	-1.21315
O	-3.80125	-1.16314	-1.32408
O	-6.13097	-0.61468	-1.97870
O	-4.52136	1.23656	-1.33679
C	-5.46647	-0.34816	0.58576
F	-5.90498	-1.58928	0.82342
F	-6.44259	0.52101	0.87943
F	-4.41353	-0.10075	1.38177

VII'_2$_p$

C	-1.53506	2.12845	-1.23381
C	-0.43903	1.34416	-0.51709
C	0.70052	2.37010	-0.45726
C	0.22860	3.58468	-0.95172
C	-1.13796	3.39455	-1.44853
C	-0.80090	0.95004	0.90373
N	-0.04938	0.03507	1.51627
C	-0.52520	-0.09861	2.79839
C	-1.58176	0.75726	2.95577
N	-1.75328	1.41732	1.75460
Re	1.44262	-0.98988	0.35230
N	2.54882	1.02596	0.40835
C	3.80924	1.00670	0.84915
C	4.63541	2.13888	0.95369
C	4.14197	3.35896	0.55054
C	2.82108	3.44375	0.05620
C	2.02197	2.24457	0.02111
C	-2.82980	2.38526	1.51443
C	0.59181	0.35444	-2.53138
P	3.08168	-1.91023	-1.19491
C	2.45904	-3.09493	-2.46688
C	2.42875	-1.65170	1.81134
O	3.01865	-2.04619	2.75159
C	0.52151	-2.63951	0.31285
O	-0.03819	-3.67388	0.29720
C	4.07580	-0.74630	-2.23270
C	4.41530	-2.90012	-0.39400
H	5.11143	-3.28886	-1.14334
H	4.96107	-2.27138	0.31528
H	3.97313	-3.73304	0.15850
H	1.91597	-3.90632	-1.97472

H	3.29463	-3.51286	-3.03683
H	1.77712	-2.59239	-3.15835
H	4.81522	-1.30764	-2.81285
H	4.59460	-0.02139	-1.60016
H	3.42556	-0.20083	-2.92059
H	4.19730	0.03785	1.14012
H	5.64212	2.02546	1.33660
H	4.74907	4.25731	0.59909
H	-3.48694	2.03229	0.71834
H	-3.39945	2.47158	2.43911
H	-2.41535	3.35804	1.25235
H	-0.08795	-0.79481	3.49600
H	-2.23025	0.94939	3.79499
H	-1.72848	4.17945	-1.90636
H	-2.50620	1.70474	-1.45850
C	-1.20260	-0.81349	-1.43643
H	-1.72278	-0.99973	-0.49752
H	-1.89976	-0.35163	-2.14107
H	-0.85813	-1.76194	-1.84729
C	1.01579	4.75642	-0.92594
C	2.28957	4.67807	-0.41067
H	0.62066	5.69379	-1.30266
H	2.92353	5.55759	-0.36440
N	-0.00843	0.05740	-1.20430
H	1.50826	0.93044	-2.41440
H	0.82004	-0.59435	-3.01721
H	-0.11161	0.91217	-3.15973
S	-5.18877	-0.37994	-1.13464
O	-4.45934	-1.52127	-1.73306
O	-6.64847	-0.34989	-1.37897
O	-4.50354	0.93274	-1.26340
C	-5.06266	-0.71951	0.70546
F	-5.71075	-1.84876	1.04229
F	-5.59028	0.29355	1.42286
F	-3.77117	-0.85306	1.07844

VII_2$_p$

C	3.00788	-1.08523	1.79361
C	1.83778	-0.71455	0.88602
C	2.19972	0.71644	0.47644
C	3.45168	1.03019	1.00025
C	3.91075	-0.09068	1.82949
C	1.73758	-1.56475	-0.36692
N	0.58934	-1.57914	-1.04259
C	0.78026	-2.35333	-2.16271
C	2.07406	-2.80004	-2.16270
N	2.67682	-2.29650	-1.02552
Re	-1.07124	-0.47299	-0.23348
N	0.20330	1.38263	-0.76335
C	-0.37543	2.30278	-1.53912
C	0.22293	3.51621	-1.92067
C	1.48217	3.81277	-1.45116
C	2.14926	2.88086	-0.62387
C	1.48788	1.63799	-0.31923
C	4.08704	-2.51719	-0.69131
C	0.37849	0.23099	2.64134
P	-2.72387	0.93631	0.88013
C	-3.60922	0.19718	2.32185
C	-2.05199	-0.35956	-1.83543
O	-2.63521	-0.31116	-2.85665
C	-2.09969	-1.99595	0.19848
O	-2.74939	-2.94320	0.45285
C	-2.18358	2.56400	1.57318
C	-4.13318	1.46110	-0.18952
H	-4.82948	2.09006	0.37329
H	-3.75372	2.02440	-1.04688
H	-4.66003	0.58024	-0.56540
H	-4.10567	-0.72555	2.00968
H	-4.35572	0.89810	2.70837
H	-2.90430	-0.04371	3.12227
H	-3.05758	3.11261	1.93755
H	-1.68409	3.15908	0.80459
H	-1.49136	2.41648	2.40475
H	-1.37952	2.07835	-1.87797
H	-0.32513	4.19631	-2.56107
H	1.97153	4.74831	-1.70303
H	4.17798	-3.03938	0.26145
H	4.51921	-3.13035	-1.48084
H	4.61923	-1.56692	-0.63953
H	-0.01302	-2.53311	-2.87052
H	2.61725	-3.42766	-2.85088
H	4.84773	-0.09790	2.37360
H	3.08918	-2.04168	2.29237
C	0.25492	-2.12070	2.15543
H	0.37428	-2.89400	1.39690
H	0.95893	-2.29539	2.97506
H	-0.75952	-2.16447	2.54934
C	4.09009	2.25705	0.71712
C	3.44240	3.15748	-0.09885
H	5.06908	2.47741	1.12772
H	3.90429	4.10602	-0.35419
N	0.46765	-0.77353	1.54765
H	0.44411	1.23834	2.23514
H	-0.58378	0.10581	3.13783
H	1.17721	0.08038	3.37655

TSVII_1$_p$ (ν = 1010i cm^{-1})

N	-1.24879	-0.11142	1.72736
C	-0.32446	0.86023	1.87342
C	0.40733	0.99369	3.07441
C	-0.02305	0.25532	4.18812
C	-1.10390	-0.61244	4.06852
C	-1.65846	-0.80778	2.80895
C	0.00241	1.67985	0.63153
N	0.26548	0.64781	-0.46559
C	-1.06403	2.69547	0.11812
C	-0.46015	3.89764	-0.10260
C	0.89791	3.87238	0.38941
C	1.16133	2.65081	0.92915
C	-2.38606	2.32224	-0.29166
N	-3.43752	3.17433	-0.52675
C	-4.50505	2.41102	-0.94169
C	-4.08158	1.11033	-0.94951
N	-2.76870	1.06059	-0.53834
C	-3.49204	4.62066	-0.33900
Re	-1.60198	-0.77649	-0.37379
C	-1.73065	-1.14325	-2.22635
O	-1.81778	-1.34123	-3.38370
P	-0.54852	-2.95615	-0.14189
C	1.14463	-3.33384	-0.78245
C	-3.20724	-1.72245	-0.07527
O	-4.22867	-2.27806	0.12749
C	-0.48041	-3.72195	1.54107
C	-1.50655	-4.25321	-1.04701
H	-1.47047	-1.18025	4.91541
H	-2.42788	-1.54924	2.63468
H	-5.45944	2.85634	-1.17337
H	-4.62645	0.21967	-1.21593
H	-4.53783	4.90978	-0.23848
H	-2.95335	4.89867	0.56752
H	-3.05822	5.14326	-1.19546
H	0.51052	0.34956	5.12913
H	1.53632	4.74389	0.46917
H	-1.03601	-5.23240	-0.91314
H	-2.53068	-4.29199	-0.66819
H	-1.54047	-4.01415	-2.11346
H	0.14170	-3.13223	2.21746
H	-1.49190	-3.76944	1.95482
H	-0.07716	-4.73768	1.47725

H	1.30757	-4.41603	-0.80243
H	1.25727	-2.94322	-1.79777
H	1.89925	-2.88975	-0.13478
C	1.65478	1.75007	3.10385
C	2.07221	2.46275	2.03098
H	2.24515	1.70561	4.01357
H	2.98663	3.04813	2.06904
H	-0.90806	4.75675	-0.58270
C	0.32939	1.26045	-1.81010
H	1.04107	2.09589	-1.85690
H	0.66510	0.48650	-2.50431
H	-0.63535	1.62749	-2.17582
N	2.85739	-0.20081	-0.70598
H	2.59851	-1.03140	-1.25340
H	1.73480	0.20563	-0.45461
Si	3.76084	-0.90103	0.77783
Si	3.88469	0.82591	-1.89175
C	5.60314	0.07028	-2.06196
C	3.11593	0.74851	-3.61028
C	3.99320	2.57169	-1.21687
H	2.34171	1.50170	-3.77230
H	3.91635	0.92908	-4.33758
H	2.68905	-0.23547	-3.83399
H	3.02158	3.07240	-1.20545
H	4.39871	2.59870	-0.20020
H	4.66659	3.14995	-1.86082
H	6.13408	0.66309	-2.81728
H	6.20488	0.08751	-1.15047
H	5.55902	-0.96083	-2.42743
C	4.73469	-2.40104	0.18443
C	4.92732	0.36767	1.52409
C	2.46981	-1.44514	2.03853
H	5.43535	-0.12908	2.35975
H	5.70074	0.71953	0.83697
H	4.39951	1.23342	1.92905
H	4.12606	-3.09897	-0.40015
H	5.60904	-2.13183	-0.41351
H	5.09043	-2.94207	1.06986
H	2.65103	-0.95610	3.00057
H	1.44974	-1.20145	1.73273
H	2.52812	-2.52709	2.19737

VIII_1$_p$

C	-1.06473	1.59323	0.68525
N	-0.12795	0.82825	1.28289
C	0.44140	1.25894	2.42717
C	0.16392	2.51450	2.96122
C	-0.64411	3.38266	2.23568
C	-1.26382	2.93894	1.05494
Re	0.83185	-0.68325	-0.07349
C	1.48368	-1.84950	-1.41571
O	1.86110	-2.57712	-2.25772
C	-1.75903	0.99088	-0.52933
C	-2.66948	-0.26431	-0.38623
C	-3.82762	-0.02323	-1.06334
C	-3.87529	1.34920	-1.52511
C	-2.74044	1.99785	-1.15243
C	-2.22615	-1.52319	0.15052
N	-0.93839	-1.87572	0.31076
C	-0.93048	-3.17175	0.77142
C	-2.22057	-3.60633	0.90969
N	-3.03477	-2.56818	0.52073
N	-0.62754	0.52826	-1.44779
C	-0.06053	1.62715	-2.27120
C	-4.49411	-2.60162	0.58680
P	2.81687	0.71849	-0.41767
C	2.80956	2.50184	0.07742
C	1.80995	-1.63769	1.22591
O	2.38504	-2.24217	2.05458
C	4.30455	0.11744	0.49462
C	3.49927	0.82252	-2.13397
H	2.07355	3.06587	-0.50063
H	2.56829	2.60298	1.13864
H	3.79958	2.93309	-0.10252
H	4.11726	0.13120	1.57150
H	5.16674	0.75292	0.26997
H	4.52746	-0.91122	0.19941
H	4.47707	1.31438	-2.11003
H	2.83908	1.39675	-2.78732
H	3.61232	-0.18318	-2.54792
H	1.15985	0.59052	2.88540
H	0.63177	2.82126	3.88890
H	-0.78942	4.40825	2.55931
H	-4.86960	-1.64291	0.94718
H	-4.92454	-2.82007	-0.39344
H	-4.78536	-3.38419	1.28697
H	-0.00968	-3.69296	0.97579
H	-2.62822	-4.54184	1.25874
H	0.32478	2.40974	-1.61691
H	-0.81691	2.05569	-2.93379
H	0.75227	1.21852	-2.86867
H	-4.74574	1.81249	-1.97355
H	-4.61261	-0.74265	-1.24857
C	-1.98041	3.85754	0.17510
C	-2.62024	3.41968	-0.93511
H	-1.98001	4.90836	0.44520
H	-3.17722	4.10416	-1.56758
H	-1.03316	-0.14344	-2.10010

TSVII_2$_p$ (ν = 751i cm-1)

C	0.60913	2.98790	0.90202
C	-0.16884	1.81694	0.29949
C	-1.56701	2.07610	0.87419
C	-1.55847	3.24325	1.63167
C	-0.18866	3.77350	1.64778
C	-0.28054	1.88061	-1.21105
N	-0.56338	0.76244	-1.87356
C	-0.72901	1.09277	-3.19816
C	-0.55004	2.44304	-3.33065
N	-0.27396	2.93819	-2.06829
Re	-0.80186	-1.01566	-0.69174
N	-2.72180	0.09602	0.05618
C	-3.88419	-0.54269	-0.08173
C	-5.10728	-0.09692	0.45383
C	-5.13083	1.07025	1.18392
C	-3.93532	1.80736	1.34679
C	-2.73453	1.30165	0.73363
C	-0.08715	4.35943	-1.77291
C	0.22152	0.20498	2.07399
P	-1.25885	-2.81463	0.89016
C	0.16940	-3.67823	1.68382
C	-1.76525	-1.94721	-2.02722
O	-2.34108	-2.47669	-2.91130
C	0.73902	-1.94575	-1.22294
O	1.69317	-2.57149	-1.53321
C	-2.31607	-2.46055	2.36733
C	-2.15164	-4.26044	0.16509
H	-2.28681	-5.04400	0.91714
H	-3.13020	-3.94813	-0.21042
H	-1.57766	-4.66105	-0.67477
H	0.85693	-4.03309	0.91103
H	-0.19097	-4.53185	2.26692
H	0.71400	-3.00183	2.34668
H	-1.82771	-1.73113	3.01772
H	-2.48146	-3.38510	2.92968
H	-3.28243	-2.05454	2.05891
H	-3.85287	-1.47622	-0.63109
H	-6.00079	-0.68800	0.29338

H	-6.04940	1.43530	1.63350
H	0.89790	4.53466	-1.34015
H	-0.17275	4.90843	-2.71016
H	-0.85360	4.70611	-1.07864
H	-0.95686	0.34961	-3.94582
H	-0.58632	3.09390	-4.18931
H	0.10955	4.67160	2.17581
H	1.66006	3.16282	0.70624
C	-2.73208	3.73103	2.25063
C	-3.90178	3.01956	2.09265
H	-2.71136	4.64709	2.83139
H	-4.82513	3.36742	2.54509
N	0.35339	0.45349	0.62828
H	-0.82037	0.11220	2.40295
H	0.72688	-0.73551	2.30875
H	0.68511	1.00004	2.67416
N	3.08530	0.60620	0.56386
H	3.13220	1.58691	0.86398
H	1.89614	0.45477	0.49058
Si	3.96401	0.59391	-1.08847
Si	3.89762	-0.34585	1.94856
C	5.75255	-0.01666	1.95605
C	3.24748	0.30628	3.59091
C	3.50910	-2.15750	1.64020
H	3.12094	1.39469	3.57915
H	2.30160	-0.14470	3.89582
H	3.99472	0.07022	4.35805
H	3.31406	-2.66872	2.58886
H	2.63191	-2.28476	1.00020
H	4.34904	-2.66202	1.15200
H	6.16958	-0.57382	2.80450
H	6.27787	-0.34754	1.05690
H	5.97919	1.04239	2.11940
C	5.27548	1.93807	-0.96979
C	4.69617	-1.10444	-1.40702
C	2.74467	1.01347	-2.44214
H	3.92076	-1.87667	-1.41240
H	5.15304	-1.08270	-2.40456
H	5.47602	-1.39286	-0.69749
H	5.76159	2.05189	-1.94628
H	4.82995	2.90533	-0.71021
H	6.05221	1.71215	-0.23362
H	3.29143	1.02012	-3.39323
H	1.94524	0.27316	-2.52000
H	2.29017	2.00086	-2.31472
O	-2.65544	-0.68417	-2.75093
C	-2.08077	-1.98362	0.51708
O	-2.71939	-2.88173	0.92995
C	-2.15366	2.70987	1.25002
C	-4.18090	1.30908	-0.19940
H	-4.85302	2.01224	0.30183
H	-3.87975	1.72243	-1.16581
H	-4.70816	0.36801	-0.37779
H	-3.91924	-0.54036	2.28725
H	-4.25507	1.15790	2.71952
H	-2.73227	0.38901	3.22161
H	-3.00633	3.27786	1.63493
H	-1.76610	3.21001	0.35840
H	-1.36679	2.69525	2.00917
H	-1.32857	1.81392	-2.07841
H	-0.28697	3.89252	-2.89889
H	1.98479	4.53999	-2.04225
H	4.10429	-3.17606	0.65216
H	4.50202	-3.37016	-1.06952
H	4.59847	-1.76262	-0.31442
H	-0.02960	-2.89329	-2.53911
H	2.58447	-3.82465	-2.39947
H	4.80098	0.06430	2.49665
H	3.01375	-1.87483	2.54751
C	4.08949	2.26309	0.60003
C	3.44934	3.08716	-0.29998
H	5.05975	2.52660	1.00672
H	3.91250	4.01348	-0.62511
N	0.46245	-0.64794	1.60835
H	0.43516	1.32655	2.36177
H	-0.60589	0.16534	3.21692
H	1.15391	0.13852	3.47703
H	0.29948	-1.58117	1.98927

VIII_2$_p$

C	2.97329	-0.96971	1.95502
C	1.83581	-0.67893	0.98603
C	2.21151	0.70281	0.45690
C	3.45103	1.06225	0.97931
C	3.88192	0.02080	1.92482
C	1.73157	-1.66183	-0.16289
N	0.58356	-1.74048	-0.83425
C	0.76386	-2.64674	-1.85182
C	2.05060	-3.11108	-1.79269
N	2.65925	-2.48359	-0.72239
Re	-1.06436	-0.53534	-0.13897
N	0.23133	1.23969	-0.86900
C	-0.33737	2.08219	-1.73401
C	0.25536	3.27103	-2.19678
C	1.50117	3.62065	-1.72783
C	2.16443	2.76378	-0.81963
C	1.50514	1.54684	-0.42309
C	4.05399	-2.70615	-0.33131
C	0.36331	0.30776	2.74079
P	-2.69120	0.98924	0.83962
C	-3.47531	0.44970	2.42219
C	-2.06621	-0.60988	-1.73484

Experimental Section

General Details: All manipulations were carried out under an argon atmosphere using Schlenk techniques. Solvents were distilled from Na (hexane), Na/benzophenone (THF and Et_2O) and CaH_2 (CH_2Cl_2). Compounds *cis,trans*-[Re(CO)$_2$(N-N)(N-RIm)(PMe$_3$)]OTf (**1a-d**) were prepared as previously reported.[33] Deuterated dichloromethane, was stored under nitrogen in a Young tube and used without further purification. ^{1}H NMR and ^{13}C NMR spectra were recorded on a Bruker Avance 400, Bruker Advance 300 or DPX-300 spectrometer. NMR spectra are referred to the internal residual solvent peak for ^{1}H, ^{31}P{^{1}H} and ^{13}C{^{1}H} NMR. NMR samples were prepared under nitrogen using Kontes manifolds purchased from Aldrich. IR solution spectra were obtained in a Perkin-Elmer FT 1720-X spectrometer using 0.2 mm. CaF_2 cells.

NMR Labelling Schemes:

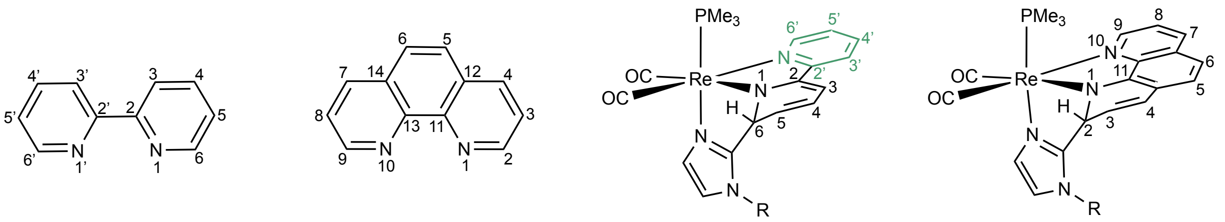

Reaction of *cis,trans*-[Re(bipy)(CO)$_2$(N-MesIm)(PMe$_3$)]OTf (1b) with KN(SiMe$_3$)$_2$. Synthesis of compound 2b. KN(SiMe$_3$)$_2$ (0.09 mL of a 0.5 M solution in toluene, 0.045 mmol) was added to a solution of *cis,trans*-[Re(CO)$_2$(bipy)(N-MesIm)(PMe$_3$)]OTf (**1b**) (30 mg, 0.037 mmol) in THF (25 mL) previously cooled to -78 ºC. The mixture was allowed to reach room temperature, and after 15 min the solvent was evaporated to dryness under reduced pressure. CH_2Cl_2 (15 mL) was added to the residue and the resulting slurry was then filtered via canula, concentrated under vacuum to a volume of 5 mL, and the addition of hexane (20 mL) caused the precipitation of a dark red solid, which was washed with hexane (20 mL), diethyl ether (2 x 20 mL), and dried under vacuum. Complex **2b** was characterized on solution at low temperature. Yield: 20 mg (47 %). IR (CH_2Cl_2, cm^{-1}): 1889, 1804 (ν_{CO}). ^{1}H NMR (CD_2Cl_2, 233 K): δ 8.87 [d (J= 5.2 Hz), 1H, $H_{6'}$], 7.60 [m, 1H, $H_{4'}$], 7.51 [d (J= 8.0 Hz), 1H, $H_{3'}$], 7.07 [m, 1H, $H_{5'}$], 6.95 [s, 1H, C*H* N-MesIm], 6.89 [s, 2H , Mes], 6.71 [m, 1H, C*H* N-MesIm], 5.78 [dd (J= 8.8, 5.4 Hz), 1H, H_4], 5.35 [m, 1H, H_3], 5.20 [d (J= 6.2 Hz), 1H, H_6], 4.18 [dd (J= 8.8, 6.2 Hz), 1H, H_5], 1.95, 1.88, 1.62 [s, 3H each, C*H$_3$* Mes], 1.23 [d (J_{HP}= 8.9 Hz), 9H, P(C*H$_3$*)$_3$]. ^{13}C{^{1}H} NMR (CD_2Cl_2, 233 K): δ 209.4 [d (J_{CP}= 4.1 Hz), *C*O], 207.1 [d (J_{CP}= 9.0 Hz), *C*O], 162.5, 157.5, 156.7, 139.0, 135.5, 135.0, 132.2 [quaternary], 151.8[$C_{6'}$], 136.3 [$C_{4'}$], 128.5 [*C*H N-MesIm and 2x*C*H Mes], 125.7 [C_4], 122.6 [$C_{5'}$], 120.8, 120.6 [$C_{3'}$ and *C*H N-MesIm], 109.0 [C_5], 99.7 [C_3], 62.4 [C_6], 17.9 [*C*H$_3$ N-MesIm], 17.8 [d (J_{CP}= 33.2 Hz), P(*C*H$_3$)$_3$], 17.2, 17.0 [*C*H$_3$ N-MesIm]. ^{31}P{^{1}H} NMR (CD_2Cl_2): -16.6.

Reaction of *cis,trans*-[Re(CO)$_2$(N-MeIm)(phen)(PMe$_3$)]OTf (1c) with KN(SiMe$_3$)$_2$. Synthesis of compound 2c. KN(SiMe$_3$)$_2$ (0.01 mL of a 0.5 M solution in toluene, 0.050 mmol) was added to a solution of *cis,trans*-[Re(CO)$_2$(N-MeIm)(phen)(PMe$_3$)]OTf (**1c**) (30 mg, 0.041 mmol) in THF (25 mL) previously cooled to -78 ºC. The mixture was allowed to reach room temperature, and after 20 min the solvent was evaporated to dryness under reduced pressure. CH_2Cl_2 (15 mL) was added to the residue and the resulting slurry was then filtered via canula, concentrated under vacuum to a volume of 5 mL, and the addition of hexane (20 mL) caused the precipitation of a brownish solid, which was washed with hexane (20 mL), diethyl ether (2 x 20 mL), and dried under vacuum. Slow diffusion of hexane (10 mL) into a concentrated solution in THF (5 mL) at – 20 ºC, afforded orange crystals of **2c**, one of which was used for a solid-state structure determination by X-ray diffraction. Yield: 18 mg (76 %). IR (CH_2Cl_2, cm^{-1}): 1890, 1806 (ν_{CO}). ^{1}H NMR (CD_2Cl_2): δ 8.91 [d (J= 4.9 Hz), 1H, H_9], 7.91 [d (J= 8.2 Hz), 1H, H_7], 7.12 [dd (J= 8.2, 4.9 Hz), 1H, H_8], 6.95 [d (J= 8.0 Hz), 1H, H_5/H_6], 6.89 [s, 1H, C*H* N-MeIm], 6.69 [d (J= 9.1 Hz), 1H, H_4], 6.62 [d (J= 8.0 Hz), 1H, H_5/H_6],], 6.60 [s, 1H, C*H* N-MeIm], 5.97 [dd (J= 9.1, 5.4 Hz), 1H, H_3], 5.65 [d (J= 5.4 Hz), 1H, H_2], 3.63 [s, 3H, C*H$_3$* N-MeIm], 1.30 [d ($^2J_{HP}$= 9.1 Hz), 9H, P(C*H$_3$*)$_3$]. ^{13}C{^{1}H} NMR (CD_2Cl_2, 213 K): δ 209.6 [d (J_{CP} = 6.1 Hz), *C*O], 206.7 [d (J_{CP} = 9.9 Hz), *C*O], 157.6, 145.2, 130.3, 120.4 [quaternary phen], 153.7 [N*C*N N-MeIm], 148.1 [C_9], 135.6 [C_7], 128.5 [*C*H N-MeIm], 127.3 [C_4], 125.8 [C_5/C_6], 120.9 [C_8], 121.6 [*C*H N-MeIm],

120.1 [C_3], 109.6 [C_5/C_6], 61.5 [C_2], 33.8 [CH_3 N-MeIm], 17.5 [d (J_{CP}= 33.5 Hz), P(CH_3)$_3$]. ^{31}P{^{1}H} NMR (CD_2Cl_2): -17.6. Anal. Calcd. for $C_{21}H_{22}N_4O_2PRe$: C 40.52, H 3.83, N 9.67. Found: C 40.60, H 3.78, N 9.60.

Reaction of *cis,trans*-[Re(CO)$_2$(N-MesIm)(phen)(PMe$_3$)]OTf (1d) with KN(SiMe$_3$)$_2$. Synthesis of compound 2d. Following the procedure described for the synthesis of compound **2c**, starting from *cis,trans*-[Re(CO)$_2$(N-MesIm)(phen)(PMe$_3$)]OTf (**1d**) (30 mg, 0.034 mmol) and KN(SiMe$_3$)$_2$ (0.08 mL of a 0.5 M solution in toluene, 0.040 mmol) allowed the isolation of compound **2d** as a brown solid. Yield: 22 mg (89 %). IR (CH_2Cl_2, cm^{-1}): 1891, 1808 (ν_{CO}). ^{1}H NMR (CD_2Cl_2): δ 8.94 [d (J= 4.9 Hz), 1H, H_9], 7.92 [d (J= 8.3 Hz), 1H, H_7], 7.14 [dd (J= 8.3, 4.9 Hz), 1H, H_8], 7.10, [s, 1H, C*H* N-MesIm], 7.00 [s, 1H, C*H* Mes], 6.91 [s, 1H, C*H* N-MesIm], 6.87 [d (J= 7.9 Hz), 2H, H_5/H_6], 6.63 [s, 1H, C*H* Mes], 6.60 [d (J= 7.9 Hz), 1H, H_5/H_6], 6.25 [d (J= 9.5 Hz), 1H, H_4], 5.50 [d (J= 5.9 Hz), 1H, H_2], 4.76 [dd (J= 9.5, 5.9 Hz), 1H, H_3], 2.35, 1.97, 1.66 [s, 3H each, CH_3 Mes], 1.31 [d (J_{HP}= 8.9 Hz), 9H, P(CH_3)$_3$]. ^{13}C{^{1}H} NMR (CD_2Cl_2): δ 209.9 [d (J_{CP} = 6.3 Hz), *C*O], 206.1 [d (J_{CP} = 7.7 Hz), *C*O],158.5, 155.7, 146.2, 139.9, 136.2, 136.0, 131.3, 129.6, 121.1 [quaternary], 148.3 [C_9], 136.2 [C_7], 130.6 [*C*H N-MesIm], 129.2, 129.1 [*C*H Mes], 127.0 [C_4], 126.4 [C_5/C_6], 121.3 [C_8], 120.9 [*C*H N-MesIm], 120.4 [C_3], 109.9 [C_5/C_6], 63.1 [C_2], 21.3 [*C*H_3 Mes], 18.5 [d (J_{CP}= 33.2 Hz), P(*C*H_3)$_3$], 17.7, 17.3 [*C*H_3 Mes]. ^{31}P{^{1}H} NMR (CD_2Cl_2): -16.8. Anal. Calcd. for $C_{29}H_{30}N_4O_2PRe$: C 50.94, H 4.42, N 8.19. Found: C 50.85, H 4.39, N 8.24.

Reaction of *cis,trans*-[Re(bipy)(CO)$_2$(N-MesIm)(PMe$_3$)]OTf (1b) with KN(SiMe$_3$)$_2$ and MeOTf. Synthesis of compound 3b. KN(SiMe$_3$)$_2$ (0.13 mL of a 0.5 M solution in toluene, 0.065 mmol) was added to a solution of *cis,trans cis,trans*-[Re(bipy)(CO)$_2$(N-MesIm)(PMe$_3$)]OTf (**1b**) (45 mg, 0.056 mmol) in THF (20 mL) previously cooled to -78 ºC. The mixture was allowed to reach room temperature, and after 20 min the solvent was evaporated to dryness under reduced pressure. CH_2Cl_2 (15 mL) was added to the residue and the resulting slurry was then filtered via canula, MeOTf (7 µL, 0.065 mmol) was added and the mixture was allowed to stir at room temperature for 30 min. The resulting solution was concentrated under vacuum to a volume of 5 mL, and the addition of hexane (20 mL) caused the precipitation of compound **3b** as a brown solid, which was washed with hexane (2 x 20 mL) and dried under vacuum. Yield: 28 mg (61 %). IR (CH_2Cl_2, cm^{-1}): 1927, 1849 (ν_{CO}). ^{1}H NMR (CD_2Cl_2): δ 8.92 [d (J= 5.5 Hz), 1H, $H_{6'}$], 8.00 [m, 1H, $H_{4'}$], 7.91 [d (J= 8.1 Hz), 1H, $H_{3'}$], 7.42 [m, 1H, $H_{5'}$], 7.35 [s, 1H, C*H* N-MesIm], 7.06, 6.99 [s, 1H each, Mes], 6.90 [s, 1H, C*H* N-MesIm], 6.78 [d (J= 5.6 Hz), 1H, H_3], 6.27 [dd (J= 9.3, 5.6 Hz), 1H, H_4], 5.16 [dd (J= 9.3, 5.6 Hz), 1H, H_5], 4.97 [d (J= 5.6 Hz), 1H, H_6], 3.19 [d ($^4J_{HP}$= 1.7 Hz), 3H, NCH_3], 2.37, 1.99, 1.73 [s, 3H each, CH_3 Mes], 1.45 [d (J_{HP}= 8.4 Hz), 9H, P(CH_3)$_3$]. ^{13}C{^{1}H} NMR (CD_2Cl_2): δ 203.0 [d (J_{CP}= 7.9 Hz), *C*O], 202.3 [d (J_{CP}= 4.0 Hz), *C*O], 156.1, 146.5, 145.5, 141.4, 135.3, 135.2, 131.5 [quaternary], 152.6 [$C_{6'}$], 139.7 [$C_{4'}$], 131.4 [*C*H N-MesIm], 130.0, 129.8 [*C*H Mes], 125.9 [$C_{5'}$], 124.6 [*C*H N-MesIm], 124.3, 123.8, 122.9 [C_3, $C_{3'}$, C_4 and C_5], 65.5 [C_6], 47.1 [d ($^3J_{CP}$= 3.6 Hz), N*C*H_3], 21.3 [*C*H_3 N-MesIm], 19.1 [d (J_{CP}= 33.6 Hz), P(*C*H_3)$_3$], 18.1, 17.2 [*C*H_3 N-MesIm]. ^{31}P{^{1}H} NMR (CD_2Cl_2): -24.7. Anal. Calcd. for $C_{29}H_{33}F_3N_4O_5PReS$: C 37.14, H 3.39, N 7.53. Found: C 36.89 H 3.43, N 7.50.

Reaction of *cis,trans*-[Re(CO)$_2$(N-MeIm)(phen)(PMe$_3$)]OTf (1c) with KN(SiMe$_3$)$_2$ and MeOTf. Synthesis of 3c and 3c'. KN(SiMe$_3$)$_2$ (0.13 mL of a 0.5 M solution in toluene, 0.065 mmol) was added to a solution of *cis,trans*-[Re(CO)$_2$(N-MeIm)(phen)(PMe$_3$)]OTf (**1c**) (40 mg, 0.055 mmol) in THF (20 mL) previously cooled to -78 ºC. The mixture was allowed to reach room temperature, and after 20 min the solvent was evaporated to dryness under reduced pressure. CH_2Cl_2 (15 mL) was added to the residue and the resulting slurry was then filtered via canula, MeOTf (7 µL, 0.065 mmol) was added and the mixture was allowed to stir at room temperature for 30 min. The resulting solution was concentrated under vacuum to a volume of 5 mL, and the addition of hexane (20 mL) caused the precipitation of a brown solid, which was washed with hexane (2 x 20 mL) and dried under vacuum. Slow diffusion of hexane (15 mL) into a concentrated solution in CH_2Cl_2 (5 mL) at room temperature, afforded orange crystals of **3c'**, one of which was used for a solid-state structure determination by X-ray diffraction. Crystallization of the mother liquor by slow diffusion of hexane (15 mL) into a concentrated solution in CH_2Cl_2 (5 mL) at -20 ºC, afforded orange crystals of **3c**.

Compound 3c: Yield: 14 mg (34 %). IR (CH_2Cl_2, cm^{-1}): 1927, 1850 (ν_{CO}). ^{1}H NMR (CD_2Cl_2): δ 9.28 [d (J= 5.0 Hz), 1H, H_9], 8.40 [d (J= 8.9 Hz), 1H, H_7], 7.93, 7.57 [d (J= 8.3 Hz), 1H each, H_5/H_6], 7.55 [m, 1H, H_8], 7.32 [d (J= 9.6 Hz), 1H, H_4], 6.94 [s, 1H, C*H* N-MeIm], 6.81 [dd (J= 9.6, 5.5 Hz), 1H, H_3], 6.72 [d (J= 1.6 Hz), 1H, C*H* N-MeIm], 5.38 [d (J= 5.5 Hz), 1H, H_2], 3.65 [s, 3H, CH_3 N-MeIm], 3.44 [d ($^4J_{HP}$= 1.9 Hz), 3H, NCH_3], 1.58 [d (J_{HP}=

8.5 Hz), 9H, P(CH_3)$_3$]. $^{13}C\{^1H\}$ NMR (CD_2Cl_2): δ 202.8, 202.3 [*C*O], 154.1 [d (J_{CP} = 1.0 Hz), C_9], 144.5, 144.0, 1403, 139.2, 130.7 [quaternary], 138.7 [C_7], 130.5, 130.4 [C_5/C_6 and *C*H N-MeIm], 128.6 [C_4], 127.8 [C_5/C_6], 125.7 [C_3], 125.2 [*C*H N-MeIm], 123.5 [C_8], 65.6 [C_2], 49.3 [d (J_{CP}= 3.1 Hz), N*C*H_3], 35.7 [*C*H_3 N-MeIm], 19.5 [d (J_{CP}= 34.0 Hz), P(*C*H_3)$_3$]. $^{31}P\{^1H\}$ NMR (CD_2Cl_2): -25.3. Anal. Calcd. for $C_{23}H_{25}F_3N_4O_5PReS$: C 37.14, H 3.39, N 7.53. Found: C 37.26, H 3.47, N 7.42. **Compound 3c'**: Yield: 16 mg (39 %). IR (CH_2Cl_2, cm^{-1}): 1923, 1844 (ν_{CO}). ^1H NMR (CD_2Cl_2): δ 9.52 [m, 1H, H_9], 8.27 [dd (J= 8.4, 1.2 Hz), 1H, H_7], 7.83 [d (J= 8.4 Hz), 1H H_5/H_6], 7.47 [m, 2H, H_8 and H_5/H_6], 7.20 [d (J= 9.6 Hz), 1H, H_4], 7.03 [dd (J= 9.6, 5.6 Hz), 1H, H_3], 6.85, 6.70 [d (J= 1.3 Hz), 1H each, C*H* N-MeIm], 5.53 [d (J= 5.6 Hz), 1H, H_2], 3.74 [s, 3H, CH_3 N-MeIm], 3.41 [s, 3H, NCH_3], 1.52 [d ($^2J_{HP}$= 8.6 Hz), 9H, P(CH_3)$_3$]. $^{13}C\{^1H\}$ NMR (CD_2Cl_2): δ 206.1 [d (J_{CP} = 8.0 Hz), *C*O], 202.6 [d (J_{CP} = 4.5 Hz), *C*O],155.8 [C_9], 146.4 [N*C*N N-MeIm], 144.4, 142.1, 130.5, 130.2 [quaternary phen], 137.2, 129.7, 127.5, 123.8 [C_5, C_6, C_7, C_8], 129.1 [*C*H N-MeIm], 127.6 [C_4], 127.0 [C_3], 125.3 126.0 [*C*H N-MeIm], 64.3 [C_2], 48.7 [*C*H_3 N-MeIm], 35.9 [N*C*H_3], 19.3 [d (J_{CP}= 34.2 Hz), P(*C*H_3)$_3$]. $^{31}P\{^1H\}$ NMR (CD_2Cl_2): -21.2. Anal. Calcd. for $C_{23}H_{25}F_3N_4O_5PReS$: C 37.14, H 3.39, N 7.53. Found: C 37.40, H 3.68, N 7.89.

Reaction of *cis,trans*-[Re(CO)$_2$(N-MesIm)(phen)(PMe$_3$)]OTf (1d) with KN(SiMe$_3$)$_2$ and MeOTf. Synthesis of 3d. Compound **3d** was prepared as described above for **3c** starting from *cis,trans*-[Re(CO)$_2$(N-MesIm)(phen)(PMe$_3$)]OTf (**1d**) (42 mg, 0.050 mmol), KN(SiMe$_3$)$_2$ (0.012 mL of a 0.5 M solution in toluene, 0.060 mmol) and MeOTf (7 μL, 0.060 mmol). Compound **3d** was obtained as a brown solid. Yield: 20 mg (47 %). IR (CH_2Cl_2, cm^{-1}): 1928, 1851 (ν_{CO}). ^1H NMR (CD_2Cl_2): δ 9.34 [d (J= 4.9 Hz), 1H, H_9], 8.48 [d (J= 8.3 Hz), 1H, H_7], 7.98 [d (J= 8.4 Hz), 1H, H_5/H_6], 7.62 [dd (J= 8.3, 4.9 Hz), 1H, H_8], 7.52 [d (J= 8.4 Hz), 1H, H_5/H_6], 7.17 [s, 1H, C*H* N-MesIm], 7.07, 6.95 [s, 1H each, C*H* Mes], 6.81 [d (J= 9.6 Hz), 1H, H_4], 6.75 [s, 1H, C*H* N-MesIm], 5.36 [m, 1H, H_3], 5.14 [d (J= 5.6 Hz), 1H, H_2], 3.38 [d ($^4J_{HP}$= 2.0 Hz), 3H, NCH_3], 2.38, 2.02 [s, 3H each, CH_3 Mes], 1.61 [d (J_{HP}= 8.5 Hz), 9H, P(CH_3)$_3$], 1.74 [s, 3H, CH_3 Mes]. $^{13}C\{^1H\}$ NMR (CD_2Cl_2): δ 202.7, 202.1 [*C*O], 154.4 [C_9], 145.1, 144.5, 141.5, 140.0, 135.6, 135.3 131.5, 130.7, 130.1 [quaternary], 139.0 [C_7], 131.5 [*C*H N-MesIm], 130.8 [C_5/C_6], 129.9 [2x*C*H Mes], 127.5, 127.0 [C_4 and C_5/C_6] 124.7 [C_3], 124.2 [*C*H N-MesIm], 123.7 [C_8], 65.7 [C_2], 49.3 [d ($^3J_{CP}$= 3.4 Hz), N*C*H_3], 21.3 [*C*H_3 Mes], 19.5 [d (J_{CP}= 34.7 Hz), P(*C*H_3)$_3$], 17.4, 17.3 [*C*H_3 Mes]. $^{31}P\{^1H\}$ NMR (CD_2Cl_2): -24.8.

Reaction of *cis,trans*-[Re(CO)$_2$(N-MeIm)(phen)(PMe$_3$)]OTf (1c) with KN(SiMe$_3$)$_2$ and HOTf. Synthesis of compound 9c. KN(SiMe$_3$)$_2$ (0.012 mL of a 0.5 M solution in toluene, 0.060 mmol) was added to a solution of *cis,trans*-[Re(CO)$_2$(N-MeIm)(phen)(PMe$_3$)]OTf (**1c**) (40 mg, 0.055 mmol) in THF (25 mL) previously cooled to -78 ºC. The mixture was allowed to reach room temperature, and after 20 min the solvent was evaporated to dryness under reduced pressure. CH_2Cl_2 (15 mL) was added to the residue and the resulting slurry was then filtered via canula, HOTf (6 μL, 0.066 mmol) was added and the mixture was allowed to stir at room temperature for 15 min. The colour of the solution changed from brown to orange, the solvent was evaporated under vacuum to a volume of 5 mL, and the addition of hexane (20 mL) caused the precipitation of a brownish solid, which was washed with hexane (15 mL), diethyl ether (15 mL), and dried under vacuum. Yield: 34 mg (75 %). IR (CH_2Cl_2, cm^{-1}): 1923, 1843 (ν_{CO}). ^1H NMR (CD_2Cl_2): δ 9.28 [d (J= 4.9 Hz), 1H, H_9], 8.31 [d (J= 8.3 Hz), 1H, H_7], 7.83 [d (J= 8.3 Hz), 1H, H_5/H_6], 7.73 [s_{br}, 1H, N*H*], 7.50 [d (J= 8.3 Hz), 1H, H_5/H_6], 7.44 [dd (J= 8.3, 4.9 Hz), 1H, H_8], 7.13 [d (J= 9.7 Hz), 1H, H_4], 6.93 [s, 1H, C*H* N-MeIm], 6.88 [dd (J= 9.7, 5.7 Hz), 1H, H_3], 6.65 [s, 1H, C*H* N-MeIm], 5.47 [m, 1H, H_2], 3.56 [s, 3H, CH_3 N-MeIm], 1.48 [d ($^2J_{HP}$= 8.9 Hz), 9H, P(CH_3)$_3$]. $^{13}C\{^1H\}$ NMR (CD_2Cl_2): δ 203.5 [d (J_{CP} = 4.5 Hz), *C*O], 202.9 [d (J_{CP} = 7.5 Hz), *C*O], 153.7 [C_9], 146.0, 142.2, 134.4, 133.7, 129.9 [quaternary phen and N*C*N N-MeIm], 138.0 [C_7], 130.4, 130.3 [C_3 and *C*H N-MeIm], 129.3 [C_5/C_6], 128.1 [C_4], 126.7 [C_5/C_6], 124.5 [*C*H N-MeIm], 122.7 [C_8], 57.3 [C_2], 35.3 [*C*H_3 N-MeIm], 18.7 [d (J_{CP}= 34.3 Hz), P(*C*H_3)$_3$]. $^{31}P\{^1H\}$ NMR (CD_2Cl_2): -22.4. Anal. Calcd. for $C_{22}H_{23}F_3N_4O_5PReS$: C 36.21, H 3.18, N 7.68. Found: C 36.01, H 3.25, N 7.61.

Reaction of *cis,trans*-[Re(CO)$_2$(N-MesIm)(phen)(PMe$_3$)]OTf (1d) with KN(SiMe$_3$)$_2$ and HOTf. Synthesis of compound 9d. Compound **9d** was prepared as described above for **9c** starting from *cis,trans*-[Re(CO)$_2$(N-MesIm)(phen)(PMe$_3$)]OTf (**1d**) (40 mg, 0.048 mmol), KN(SiMe$_3$)$_2$ (0.012 mL of a 0.5 M solution in toluene, 0.060 mmol) and HOTf (8 μL, 0.058 mmol). Slow diffusion of hexane (15 mL) into a concentrated solution of **9d** in CH_2Cl_2 (5 mL) at -20 ºC, afforded crystals of **9d**, one of which was used for a solid-state structure determination by X-ray diffraction. Yield: 30 mg (75 %). IR (CH_2Cl_2, cm^{-1}): 1924, 1845 (ν_{CO}). ^1H NMR (CD_2Cl_2): δ 9.34 [d (J= 4.8 Hz), 1H, H_9], 8.34 [d (J= 8.3 Hz), 1H, H_7], 7.84 [d (J= 8.4 Hz), 1H, H_5/H_6], 7.65 [s_{br}, 1H, N*H*], 7.47 [dd (J= 8.3, 4.8

Hz), 1H, H_8], 7.44 [d (J= 8.4 Hz), 1H, H_5/H_6], 7.13, 7.03, 6.88, 6.69 [s, 1H each, C*H* N-MesIm and C*H* Mes], 6.64 [d (J= 9.5 Hz), 1H, H_4], 5.55 [dd (J= 9.5, 5.6 Hz), 1H, H_3], 5.26 [m, 1H, H_2], 2.35, 1.98 [s, 3H each, CH_3 Mes], 1.51 [d (J_{HP}= 9.0 Hz), 9H, P(CH_3)$_3$], 1.24 [s, 3H, CH_3 Mes]. $^{13}C\{^1H\}$ NMR (CD_2Cl_2): δ 203.4 [d (J_{CP} = 5.8 Hz), *C*O], 202.6 [d (J_{CP} = 8.3 Hz), *C*O], 153.8 [C_9], 145.9, 145.8, 142.1, 141.2, 135.5, 135.4, 134.7, 134.0, 133.9 [quaternary], 138.1 [C_7], 131.4, 130.2, 129.9, 129.7, 129.5, 129.4, 121.4 [C_3, C_4, C_5, C_6, *C*H N-MesIm and 2x*C*H Mes], 126.5 [*C*H N-MesIm], 122.6 [C_8], 57.5 [C_2], 21.3 [CH_3 Mes], 18.8 [d (J_{CP}= 34.5 Hz), P(CH_3)$_3$], 17.5, 17.2 [CH_3 Mes]. $^{31}P\{^1H\}$ NMR (CD_2Cl_2): -22.3. Anal. Calcd. for $C_{30}H_{31}F_3N_4O_5PReS$: C 43.21, H 3.75, N 6.72. Found: C 43.14, H 3.57, N 6.91

Crystal Structure Determination Details (X-ray structures of 2b, 3c'and 9d)

General Description: Crystal data were collected on an Oxford Diffraction Xcalibur Nova single crystal diffractometer, using Cu-Kα radiation (λ= 1.5418 Å). Images were collected at a 65 mm fixed crystal-detector distance, using the oscillation method, with 1° oscillation and variable exposure time per image (4-16 s). Data collection strategy was calculated with the program CrysAlis^{Pro} CCD.[39] Data reduction and cell refinement was performed with the program CrysAlis^{Pro} RED.[39] An empirical absorption correction was applied using the SCALE3 ABSPACK.[39] Using the program suite WINGX,[40] the structures were solved by Patterson interpretation and phase expansion using SHELXL and refined with full-matrix least squares on F2 using SHELXL.[41] In general, all non-hydrogen atoms were refined anisotropically, and all hydrogen atoms were geometrically placed and refined using a riding mode. Molecular graphics were made with ORTEP 3.[42]

References

1. Barone, V.; Cossi, M., Quantum Calculation of Molecular Energies and Energy Gradients in Solution by a Conductor Solvent Model. *J. Phys. Chem. A*. **1998**, *102*, 1995-2001.

2. Cossi, M.; Rega, N.; Scalmani, G.; Barone, V., Energies, Structures, and Electronic Properties of Molecules in Solution with the C-Pcm Solvation Model. *J. Comput. Chem.* **2003**, *24*, 669-681.

3. Rappé, A. K.; Casewit, C. J.; Colwell, K. S.; III, W. A. G.; Skiff, W. M., Uff, a Full Periodic Table Force Field for Molecular Mechanics and Molecular Dynamics Simulations. *J. Am. Chem. Soc.* **1992**, *114*, 10024-10039.

4. Becke, A. D., Density-Functional Exchange-Energy Approximation with Correct Asymptotic Behavior. *Phys. Rev. A: At., Mol., Opt. Phys.* **1988**, *38*, 3098-3100.

5. Becke, A. D., Density-Functional Thermochemistry. Iii. The Role of Exact Exchange. *J. Chem. Phys.* **1993**, *98*, 5648-5652.

6. Lee, C.; Yang, W.; Parr, R. G., Development of the Colle-Salvetti Correlation-Energy Formula into a Functional of the Electron Density. *Phys. Rev. B: Condens. Matter Mater. Phys.* **1988**, *37*, 785-789.

7. Hehre, W. J.; Radom, L.; Pople, J. A.; Schleyer, P. v. R., *Ab Initio Molecular Orbital Theory*; Wiley: New York, 1986.

8. Hay, P. J.; Wadt, R. W., Ab Initio Effective Core Potentials for Molecular Calculations. Potentials for the Transition Metal Atoms Sc to Hg. *J. Chem. Phys.* **1985**, *82*, 270-283.

9. Li, X.; Frisch, M. J., Energy Represented Direct Inversion in the Iterative Subspace within a Hybrid Geometry Optimization Method. *J. Chem. Theory Comput.* **2006**, *2*, 835-839.

10. Schlegel, H. B., Optimization of Equilibrium Geometries and Transition Structures. *J. Comput. Chem.* **1982**, *3*, 214-218.

11. Schlegel, H. B., Estimating the Hessian for Gradient-Type Geometry Optimizations. *Theor. Chem. Acc.* **1984**, *66*, 333-340.

12. Tomasi, J.; Persico, M., Molecular Interactions in Solution: An Overview of Methods Based on Continuous Distributions of the Solvent. *Chem. Rev.* **1994**, *94*, 2027-2094.

13. Gonzalez, C.; Schlegel, H. B., An Improved Algorithm for Reaction Path Following. *J. Chem. Phys.* **1989**, *90*, 2154-2161.

14. Gonzalez, C.; Schlegel, H. B., Reaction Path Following in Massweighted Internal Coordinates. *J. Phys. Chem.* **1990**, *94*, 5523-5527.

15. McQuarrie, D. A., *Statistical Mechanics*; Harper and Row: New York, 1976.

16. Ribeiro, R. F.; Marenich, A. V.; Cramer, C. J.; Truhlar, D. G., Use of Solution-Phase Vibrational Frequencies in Continuum Models for the Free Energy of Solvation. *J. Phys. Chem. B* **2011**, *115*, 14556-14562.

17. Bader, R. F. W., *Atoms in Molecules. A Quantum Theory*; Oxford University Press: Oxford, 1990.

18. Bader, R. F. W.; Popelier, P. L. A.; Keith, T. A., Theoretical Definition of a Functional Group and the Molecular Orbital Paradigm. *Angew. Chem. Int. Ed. Engl.* **1994**, *33*, 620-631.

19. Biegler-Konig, F. W.; Bader, R. F. W.; Tang, T. H., Calculation of the Average Properties of Atoms in Molecules. Ii. *J. Comput. Chem.* **1982**, *3*, 317-328.

20. Glendening, E. D.; Reed, A. E.; Carpenter, J. E.; Weinhold, F. *Nbo*, 3.1; University of Wisconsin: Madison, WI, 2012.

21. Weinhold, F.; Landis, C. R., *Valency and Bonding: A Natural Bond Orbital Donor-Acceptor Perspective*; Cambridge University Press: Cambridge, 2005.

22. Cheeseman, J. R.; Trucks, G. W.; Keith, T. A.; Frisch, M. J., A Comparison of Models for Calculating Nuclear Magnetic Resonance Shielding Tensors. *J. Chem. Phys.* **1996**, *104*, 5497-5509.

23. Ditchfield, R., Self-Consistent Perturbation Theory of Diamagnetism. 1. Gauge-Invariant Lcao Method for Nmr Chemical Shifts. *Mol. Phys.* **1974**, *27*, 789-807.

24. London, F., The Quantic Theory of Inter-Atomic Currents in Aromatic Combinations. *J. Phys. Radium* **1937**, *8*, 397-409.

25. McWeeny, R., Mcweeny, R. Perturbation Theory for Fock-Dirac Density Matrix. *Phys. Rev.* **1962**, *126*, 1028-1034.

26. Wolinski, K.; Hilton, J. F.; Pulay, P., Efficient Implementation of the Gauge-Independent Atomic Orbital Method for Nmr Chemical Shift Calculations. *J. Am. Chem. Soc.* **1990**, *112*, 8251-8260.

27. Schleyer, P. v. R.; Maerker, C.; Dransfeld, A.; Jiao, H. J.; Hommes, N. J. R. v. E., Nucleus-Independent Chemical Shifts: A Simple and Efficient Aromaticity Probe. *J. Am. Chem. Soc.* **1996**, *118*, 6317-6318.

28. Chen, Z.; Wannere, C. S.; Corminboeuf, C.; Puchta, R.; Schleyer, P. v. R., Nucleus-Independent Chemical Shifts (Nics) as an Aromaticity Criterion. *Chem. Rev.* **2005**, *105*, 3842-3888.

29. Dutta, B. J.; Bhattacharyya, P. K., Reactivity and Aromaticity of Nucleobases Are Sensitive toward External Electric Field. *J. Phys. Chem. B* **2014**, *118*, 9573-9582.

30. Frash, M. V.; Hopkinson, A. C.; D.K. Bohme, Corannulene as a Lewis Base: Computational Modeling of Protonation and Lithium Cation Binding. *J. Am. Chem. Soc.* **2001**, *123*, 6687-6695.

31. Schleyer, P. v. R.; Jiao, H.; Hommes, N. J. R. v. E.; Malkin, V. G.; Malkina, O., An Evaluation of the Aromaticity of Inorganic Rings: Refined Evidence from Magnetic Properties. *J. Am. Chem. Soc.* **1997**, *119*, 12669-12670.

32. Schleyer, P. v. R.; Manoharan, M.; Wang, Z. X.; Kiran, B.; Jiao, H.; Puchta, R.; Hommes, N. J. R. v. E., Dissected Nucleus Independent Chemical Shift Analysis of Π-Aromaticity and Antiaromaticity. *Org. Lett.* **2001**, *3*, 2465-2468.

33. Cañadas, P.; Pérez, J.; López, R.; Riera, L., 1,10-Phenanthroline Ring-Opening Mediated by *cis*-{$Re(CO)_2$} Complexes. *Inorg. Chem. Front.* **2023**, *10*, 900–907.

34. Espinal-Viguri, M.; Fombona, S.; Álvarez, D.; Díaz, J.; Menéndez, M. I.; López, R.; Pérez, J.; Riera, L., Regiochemistry Control by Bipyridine Substituents in the Deprotonation of Re(I) and Mo(II) N-Alkylimidazole Complexes. *Chem. Eur. J.* **2019**, *25*, 9253–9265.

35. Fombona, S.; Espinal-Viguri, M.; Huertos, M. A.; Díaz, J.; López, R.; Menéndez, M. I.; Pérez, J.; Riera, L., Activation of Aromatic C-C Bonds of 2,2'-Bipyridine Ligands. *Chem. Eur. J.* **2016**, *22*, 17160 – 17164.

36. Arévalo, R.; Menéndez, M. I.; López, R.; Merino, I.; Riera, L.; Pérez, J., Nucleophilic Additions to Coordinated 1,10-Phenanthroline: Intramolecular, Intermolecular, Reversible, and Irreversible. *Chem. Eur. J.* **2016**, *22*, 17972–17975.

37. Arévalo, R.; López, R.; Falvello, L. R.; Riera, L.; Pérez, J., Building C(sp^3) Molecular Complexity on 2,2'-Bipyridine and 1,10-Phenanthroline in Rhenium Tricarbonyl Complexes. *Chem. Eur. J.* **2021**, *27*, 379-389.

38. Frisch, M. J. T., G. W.; Schlegel, H. B.; Scuseria, G. E.; Robb, M. A.; Cheeseman, J. R.; Scalmani, G.; Barone, V.; Mennucci, B.; Petersson, G. A.; Nakatsuji, H.; Caricato, M.; Li, X.; Hratchian, H. P.; Izmaylov, A. F.; Bloino, J.; Zheng, G.; Sonnenberg, J. L.; Hada, M.; Ehara, M.; Toyota, K.; Fukuda, R.; Hasegawa, J.; Ishida, M.; Nakajima, T.; Honda, Y.; Kitao, O.; Nakai, H.; Vreven, T.; Montgomery, J. A.; Peralta, J. E.; Ogliaro, F.; Bearpark, M.; Heyd, J. J.; Brothers, E.; Kudin, K. N.; Staroverov, V. N.; Kobayashi, R.; Normand, J.; Raghavachari, K.; Rendell, A.; Burant, J. C.; Iyengar, S. S.; Tomasi, J.; Cossi, M.; Rega, N.; Millam, J. M.; Klene, M.; Knox, J. E.; Cross, J. B.; Bakken, V.; Adamo, C.; Jaramillo, J.; Gomperts, R.; Stratmann, R. E.; Yazyev, O.; Austin, A. J.; Cammi, R.; Pomelli, C.; Ochterski, J. W.; Martin, R. L.; Morokuma, K.; Zakrzewski, V. G.; Voth, G. A.; Salvador, P.; Dannenberg, J. J.; Dapprich, S.; Daniels, A. D.; Farkas; Foresman, J. B.; Ortiz, J. V.; Cioslowski, J.; Fox, D. J. *Gaussian 09*, D.01; Gaussian Inc.: Wallingford CT, 2009.

39. *CrysAlis^{Pro} CCD, CrysAlis^{Pro} RED.* Oxford Diffraction Ltd., Abingdon, Oxfordshire, UK.

40. Farrugia, L. J., WinGX suite for small-molecule single-crystal crystallography. *J. Appl. Crystallogr.* **1999**, *32*, 837-838.

41. a) Sheldrick, G. M., SHELXT-Integrated Space-Group and Crystal-Structure Determination. *Acta Cryst.* **2015**, *A71*, 3-8; b) Sheldrick, G. M., Crystal structure refinement with *SHELXL*. *Acta Cryst.,* **2015**, *C71*, 3-8.

42 Farrugia, L. J., ORTEP-3 for Windows- a version of ORTEP-III with a Graphical User Interface (GUI). *J. Appl. Cryst.* **1997**, *30*, 565.

Figure S12. ^{1}H NMR spectrum of compound **2b** in CD_2Cl_2 at 233 K.

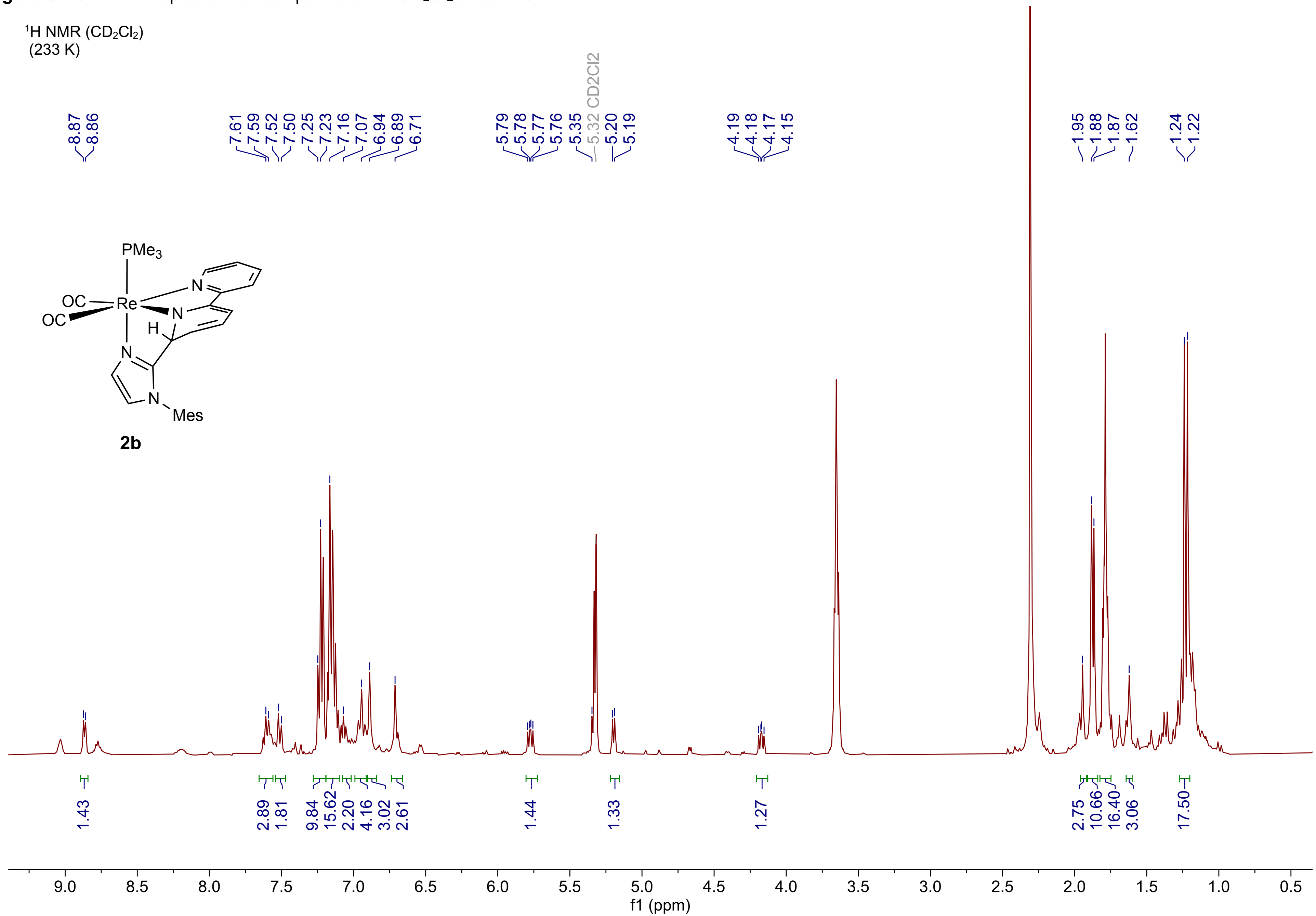

Figure S13. ^{13}C {^{1}H} NMR spectrum of compound **2b** in CD_2Cl_2 at 233 K.

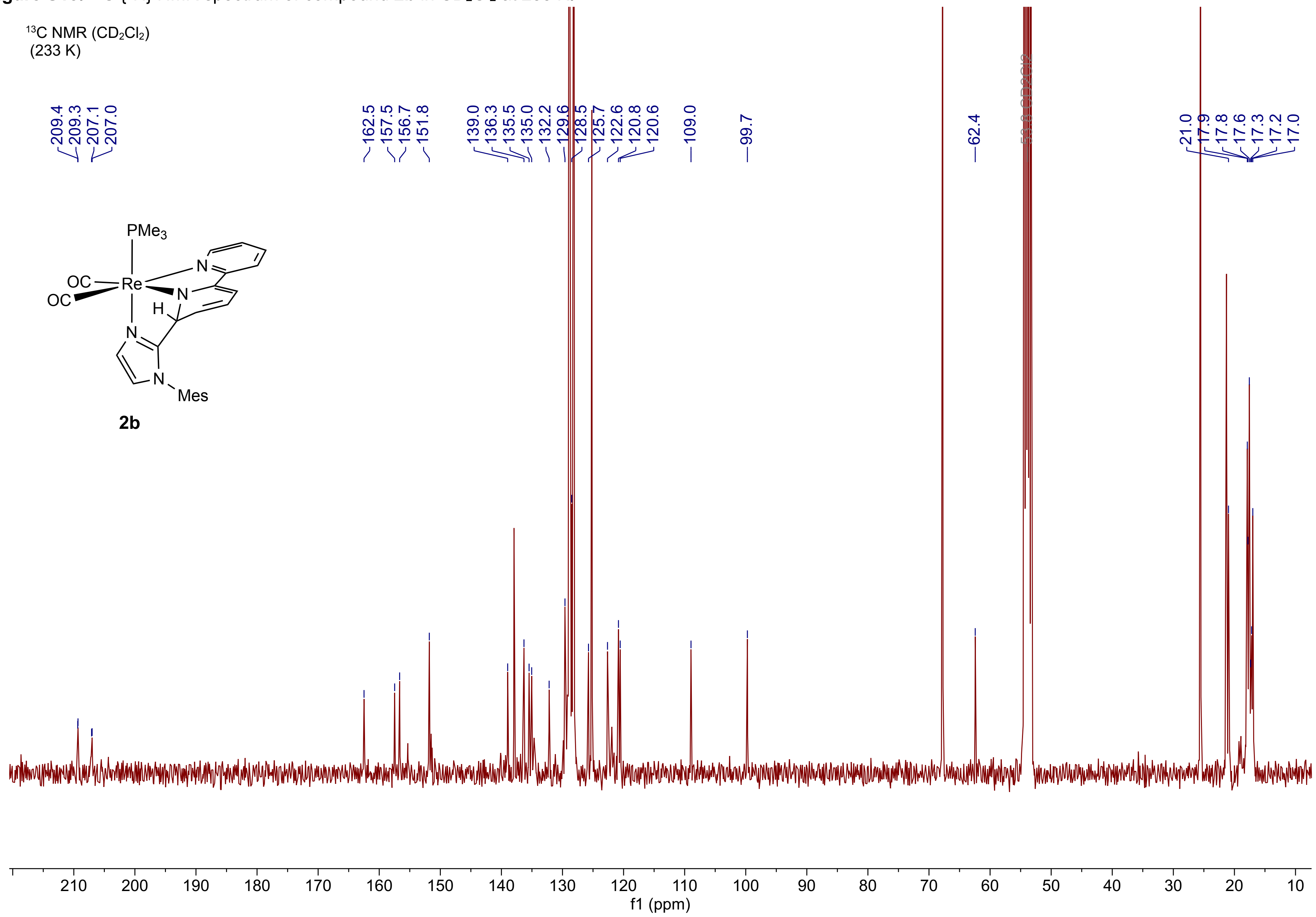

Figure S14. $^{31}P\{^{1}H\}$ NMR spectrum of compound **2b** in CD_2Cl_2

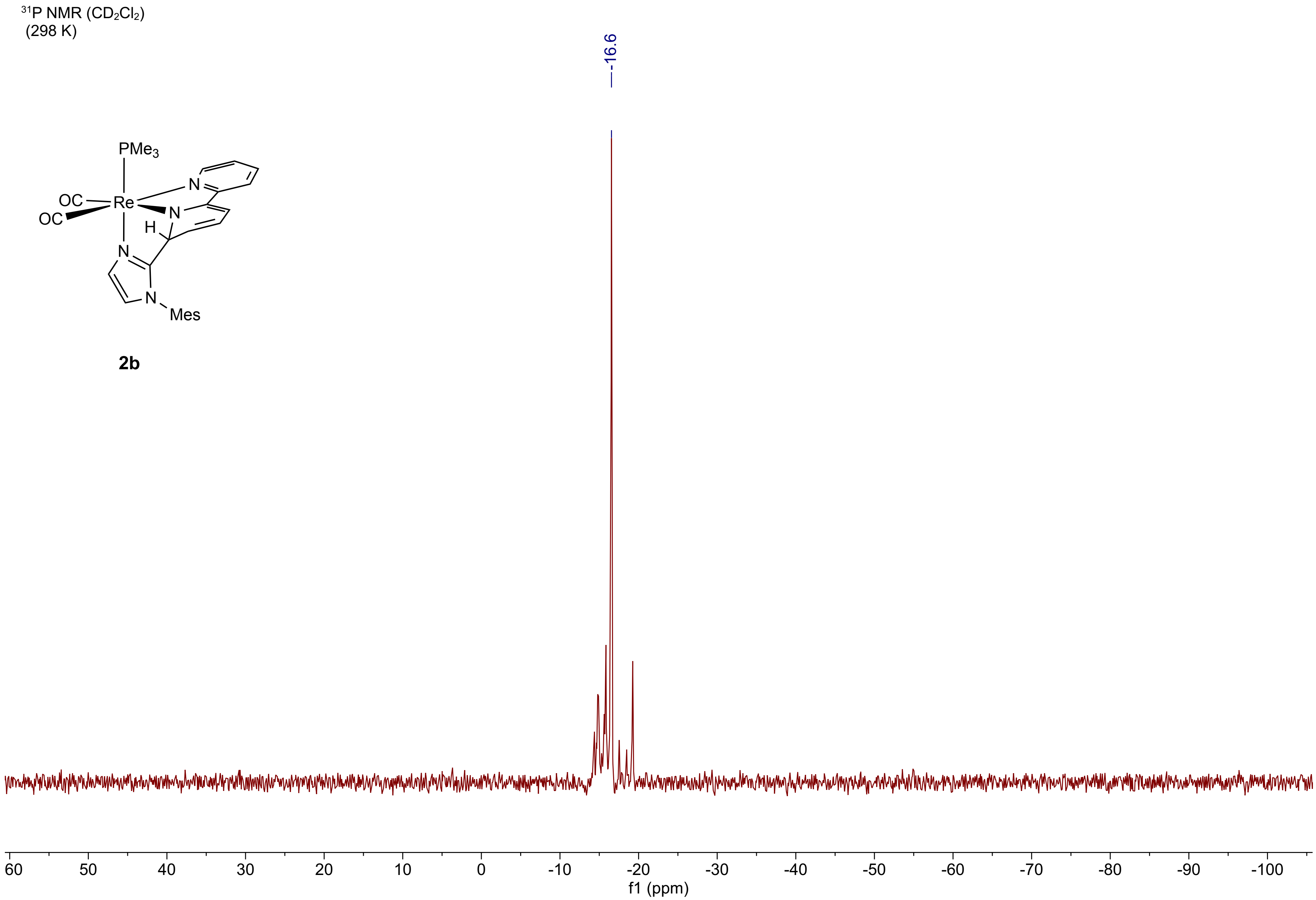

2b

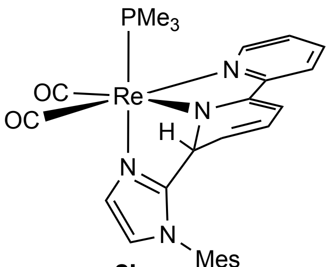

2b

Figure S15. ^{1}H-^{1}H COSY NMR spectrum of compound **2b** in CD_2Cl_2 at 233

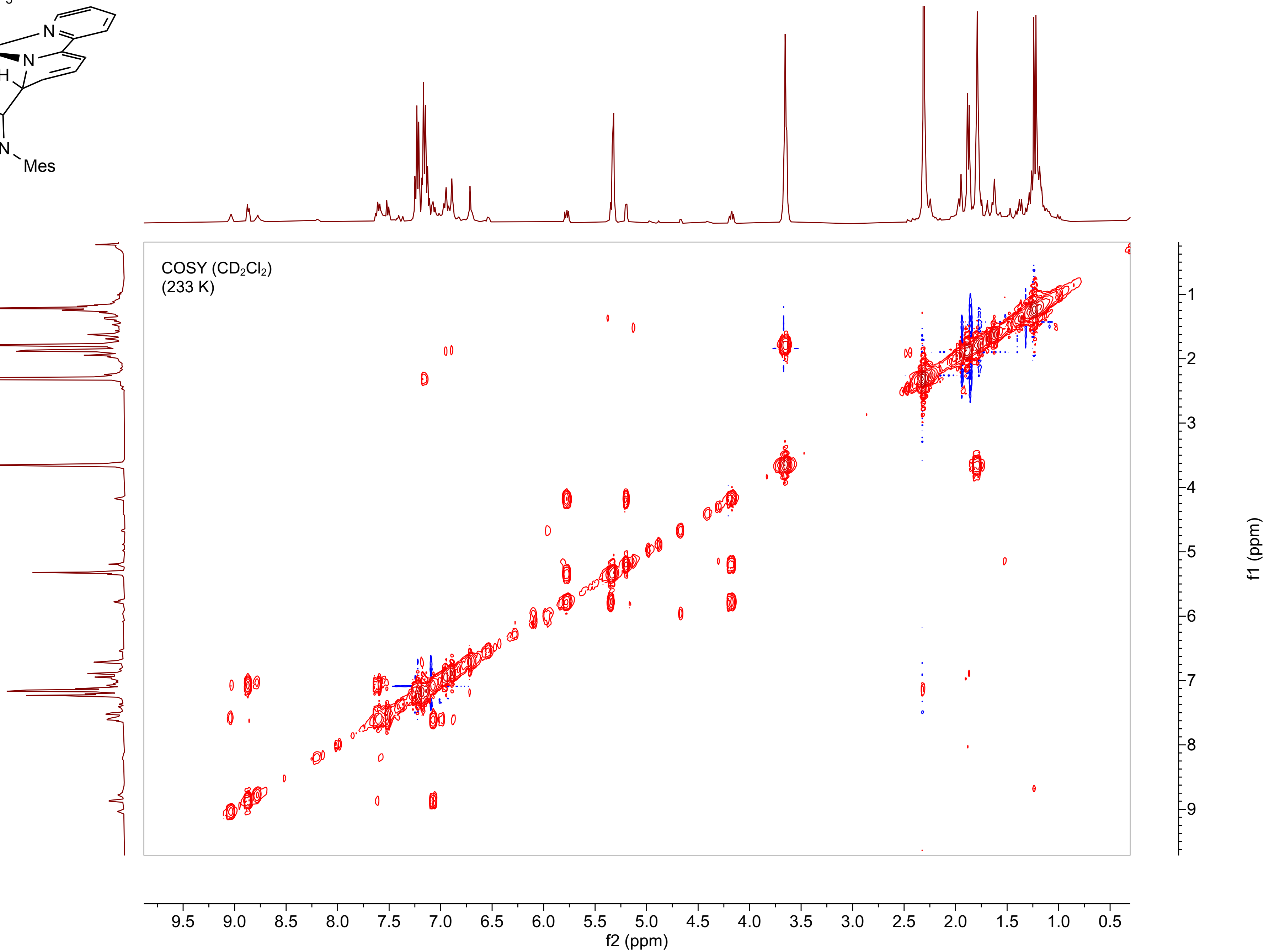

Figure S16. ^{1}H-^{13}C HSQC NMR spectrum of compound **2b** in CD_2Cl_2 at 233

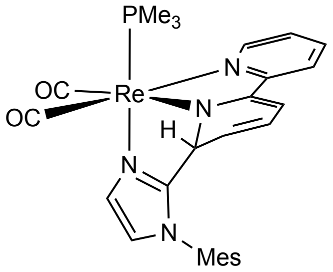

2b

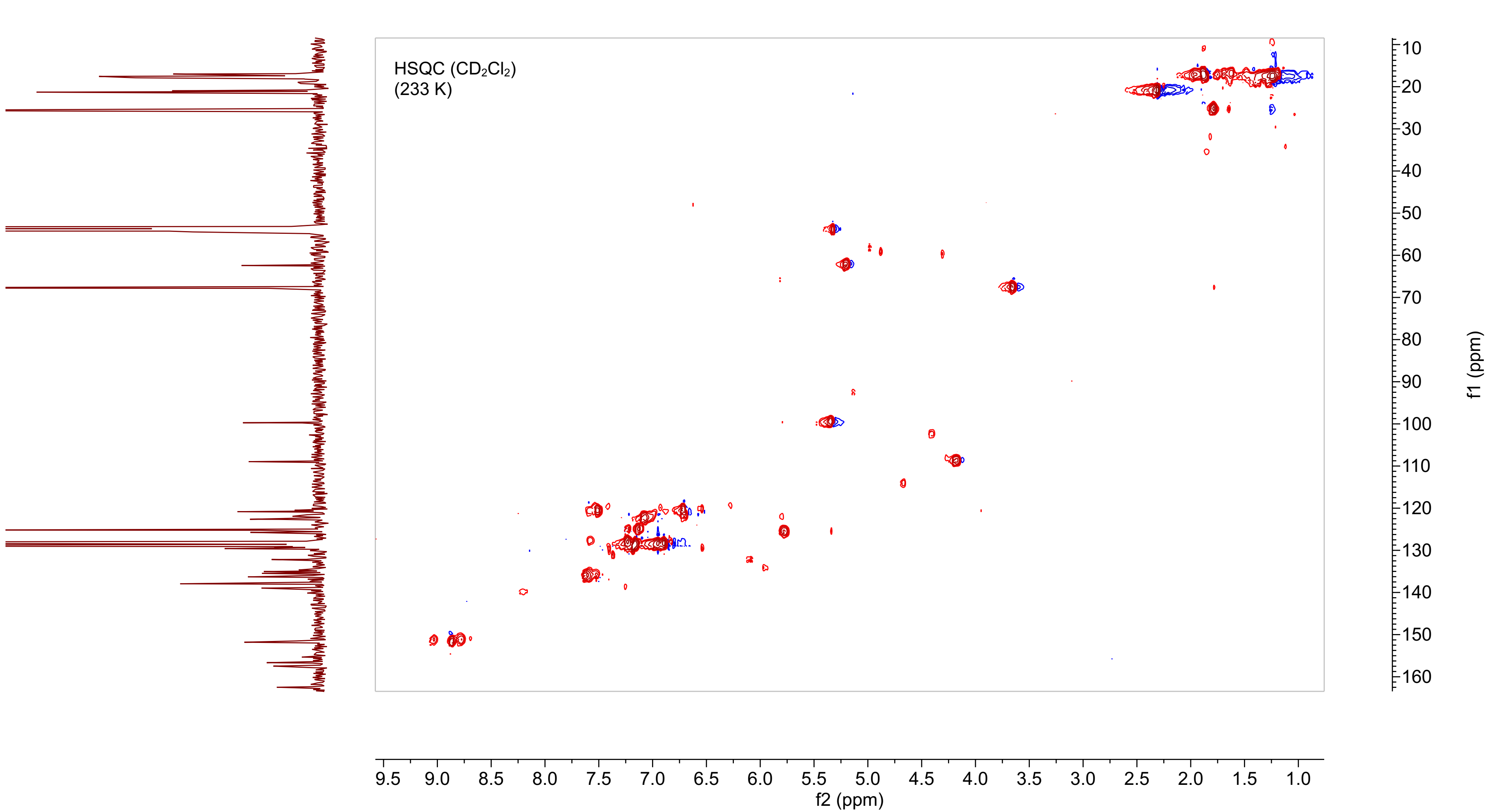

Figure S17. ^{1}H NMR spectrum of compound **2c** in CD_2Cl_2.

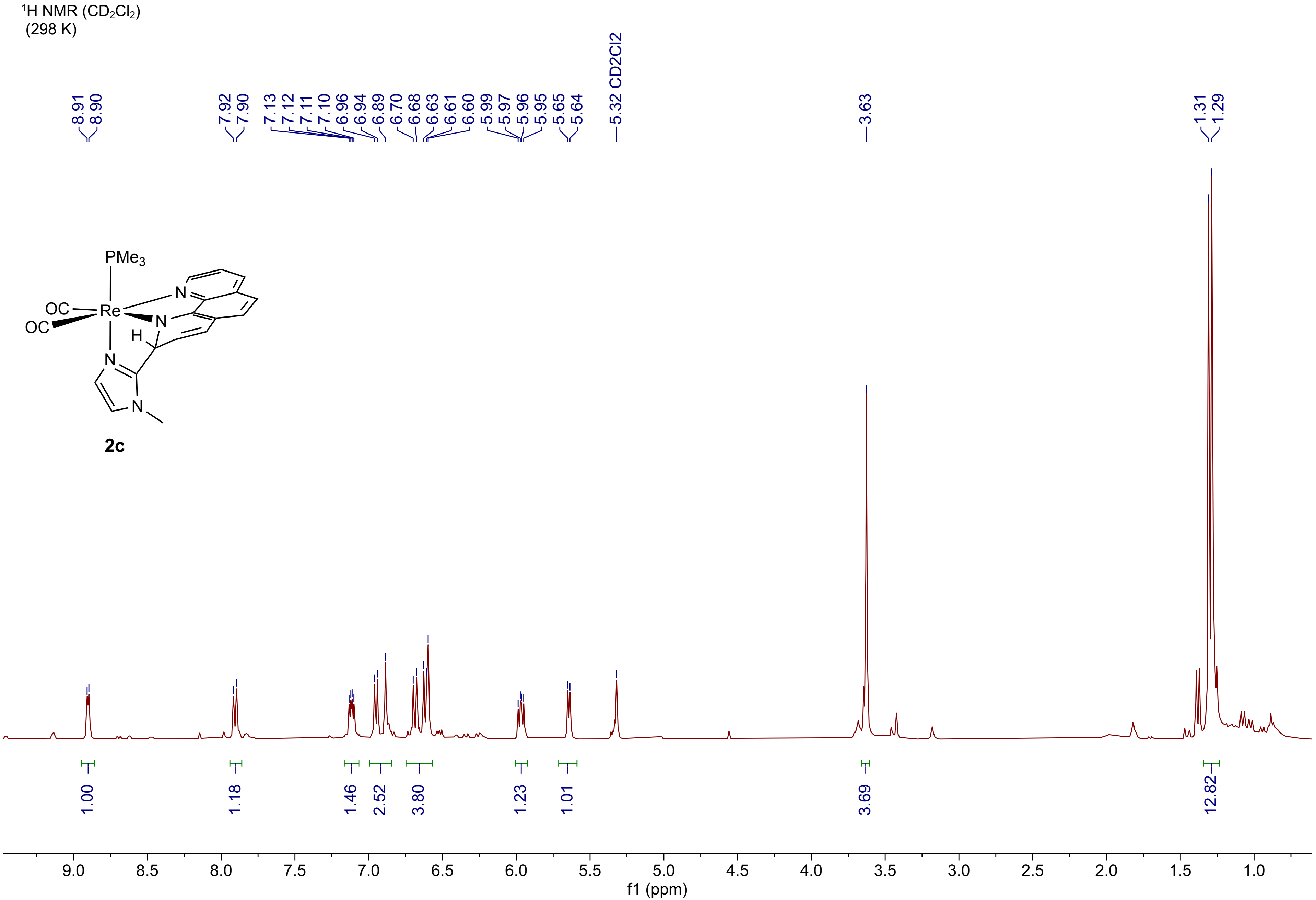

Figure S18. $^{13}C\ \{^1H\}$ NMR spectrum of compound **2c** in CD_2Cl_2 at 213 K

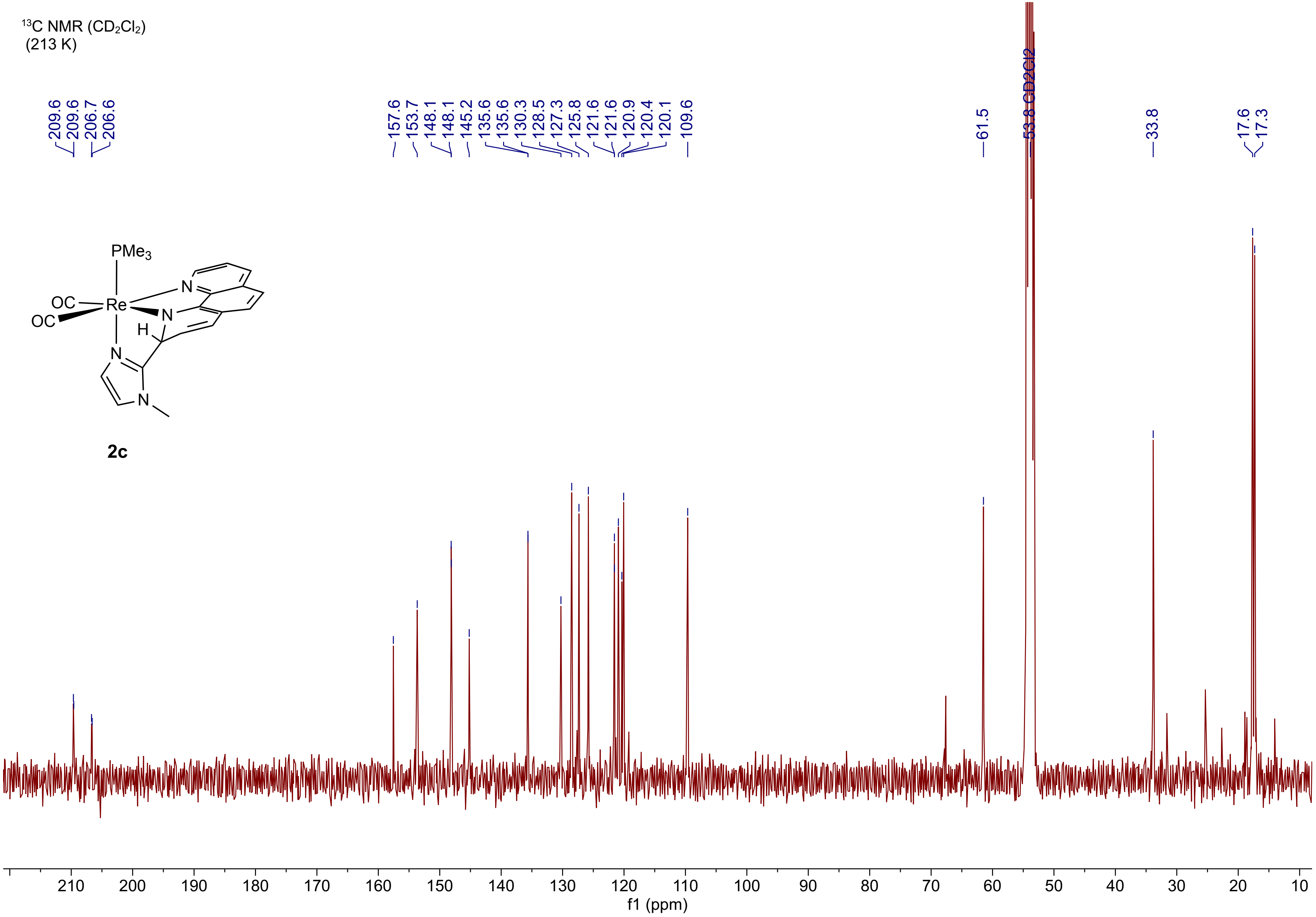

Figure S19. $^{31}P\{^1H\}$ NMR spectrum of compound **2c** in CD_2Cl_2.

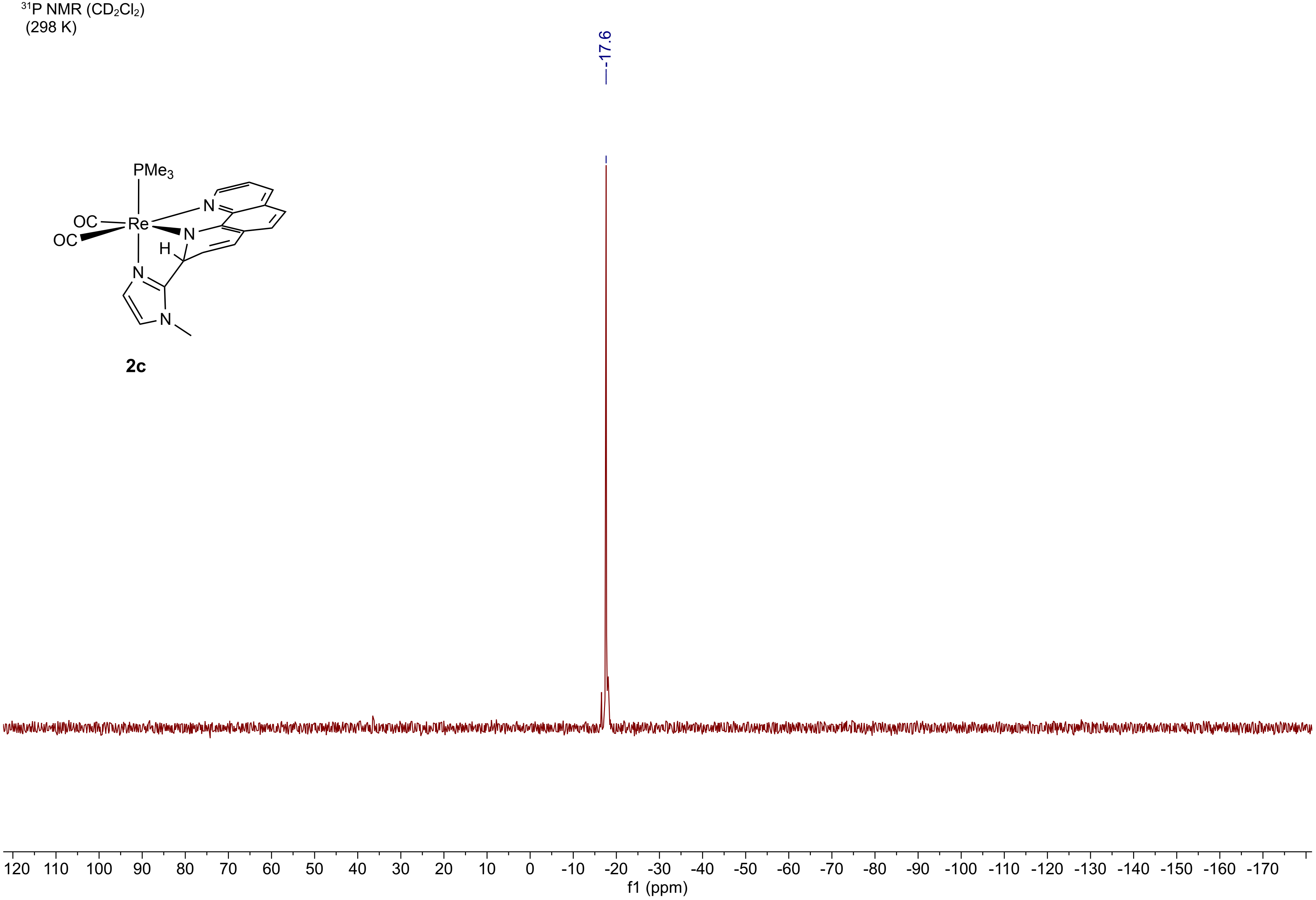

Figure S20. ^1H-^1H COSY NMR spectrum of compound **2c** in CD_2Cl_2 at 213

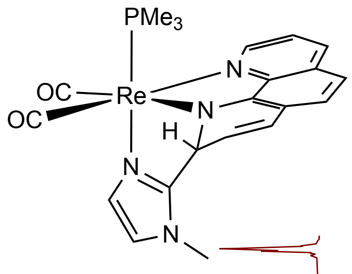

2c

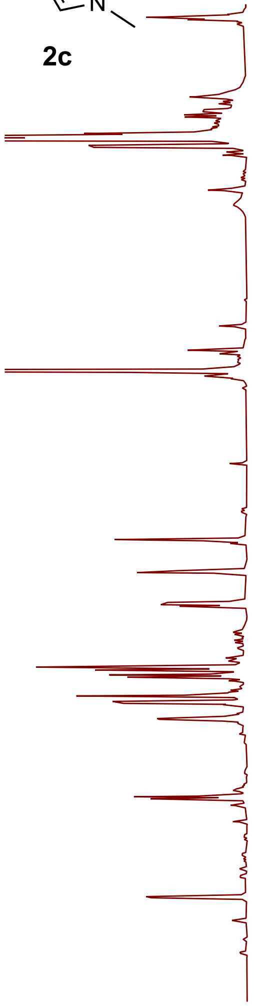

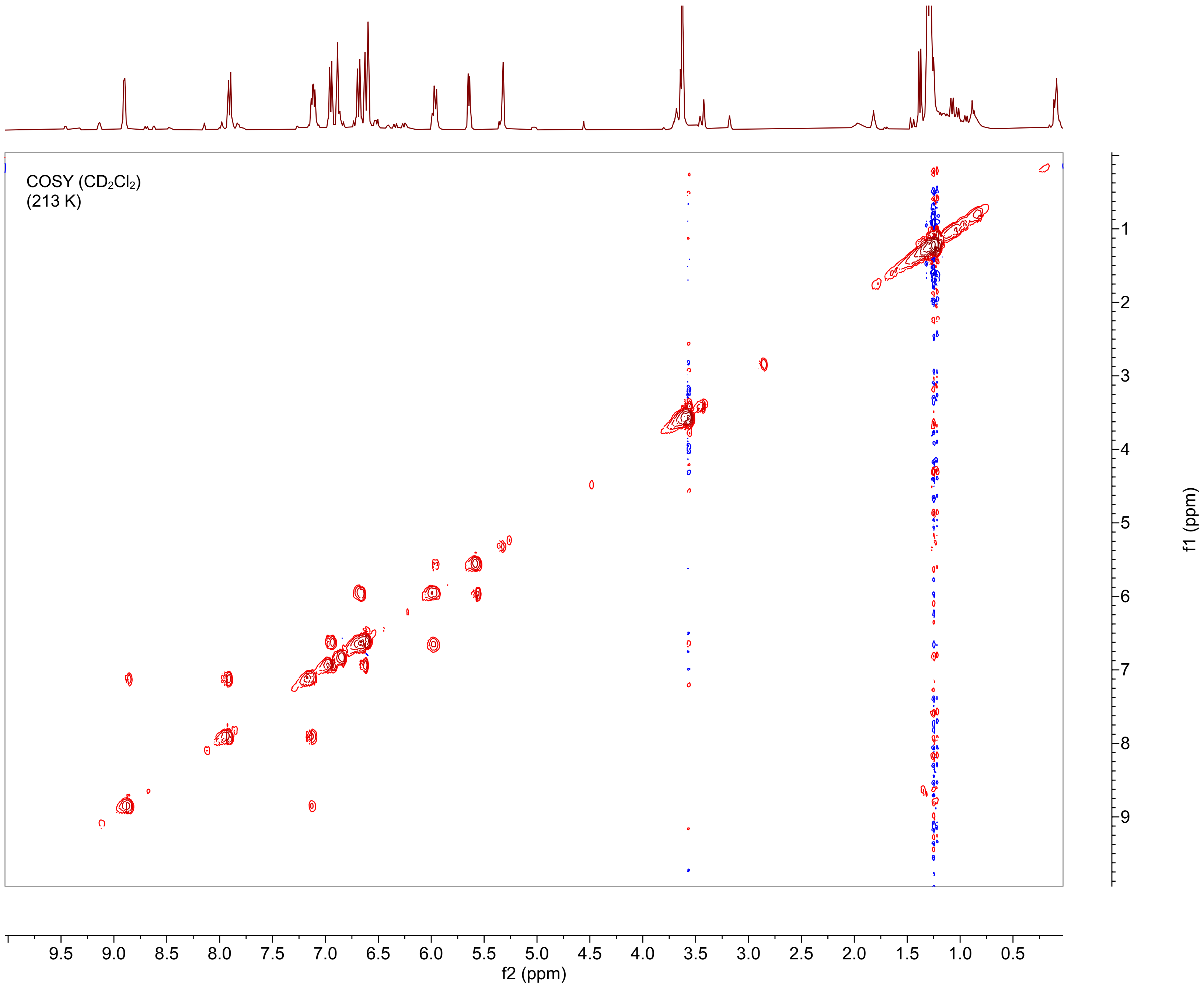

Figure S21. ^{1}H-^{13}C HSQC NMR spectrum of compound **2c** in CD_2Cl_2 at 213 K.

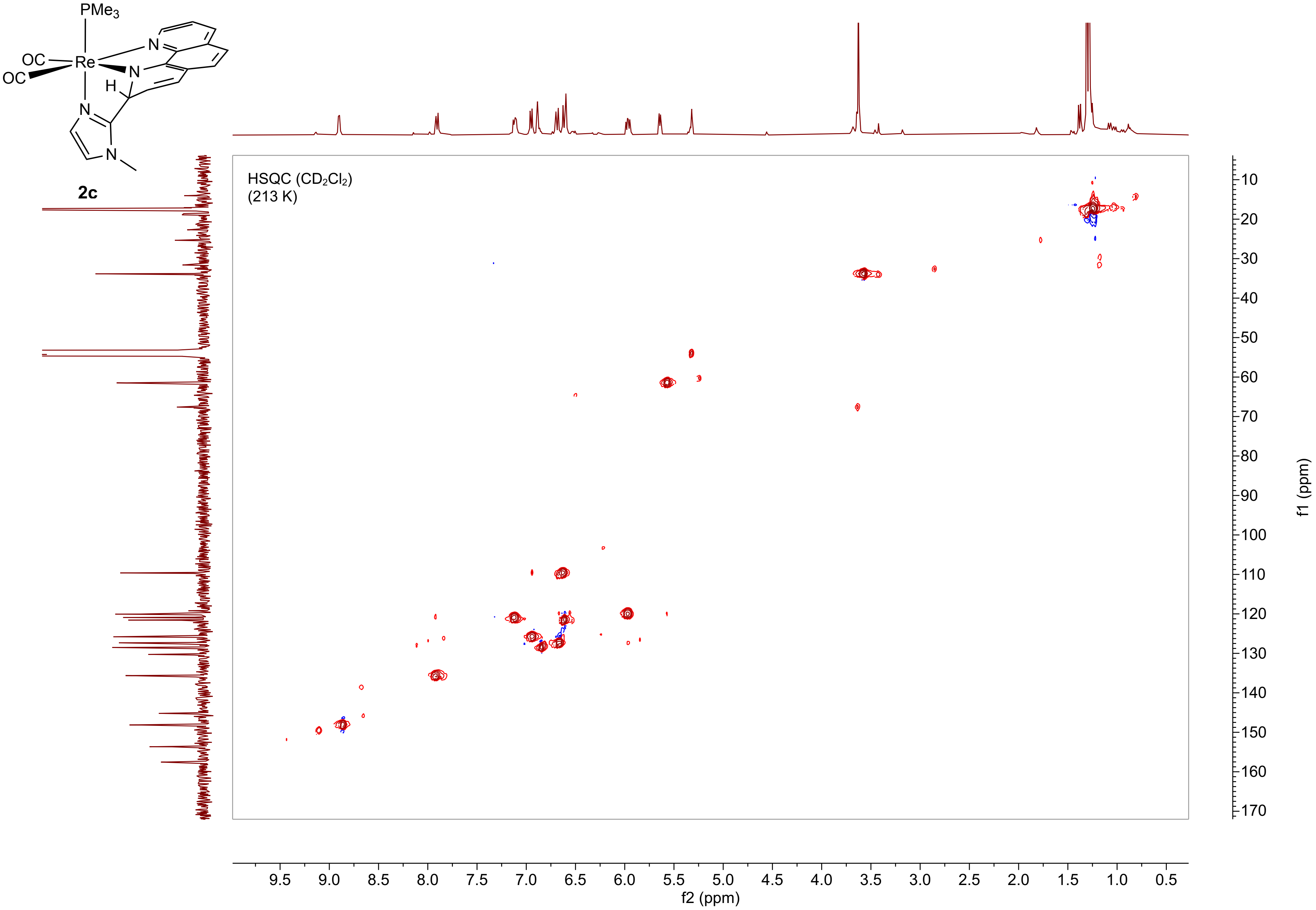

Figure S22. ${}^{1}H$-${}^{13}C$ HMBC NMR spectrum of compound **2c** in CD_2Cl_2 at 213 K.

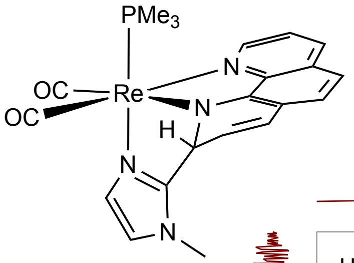

2c

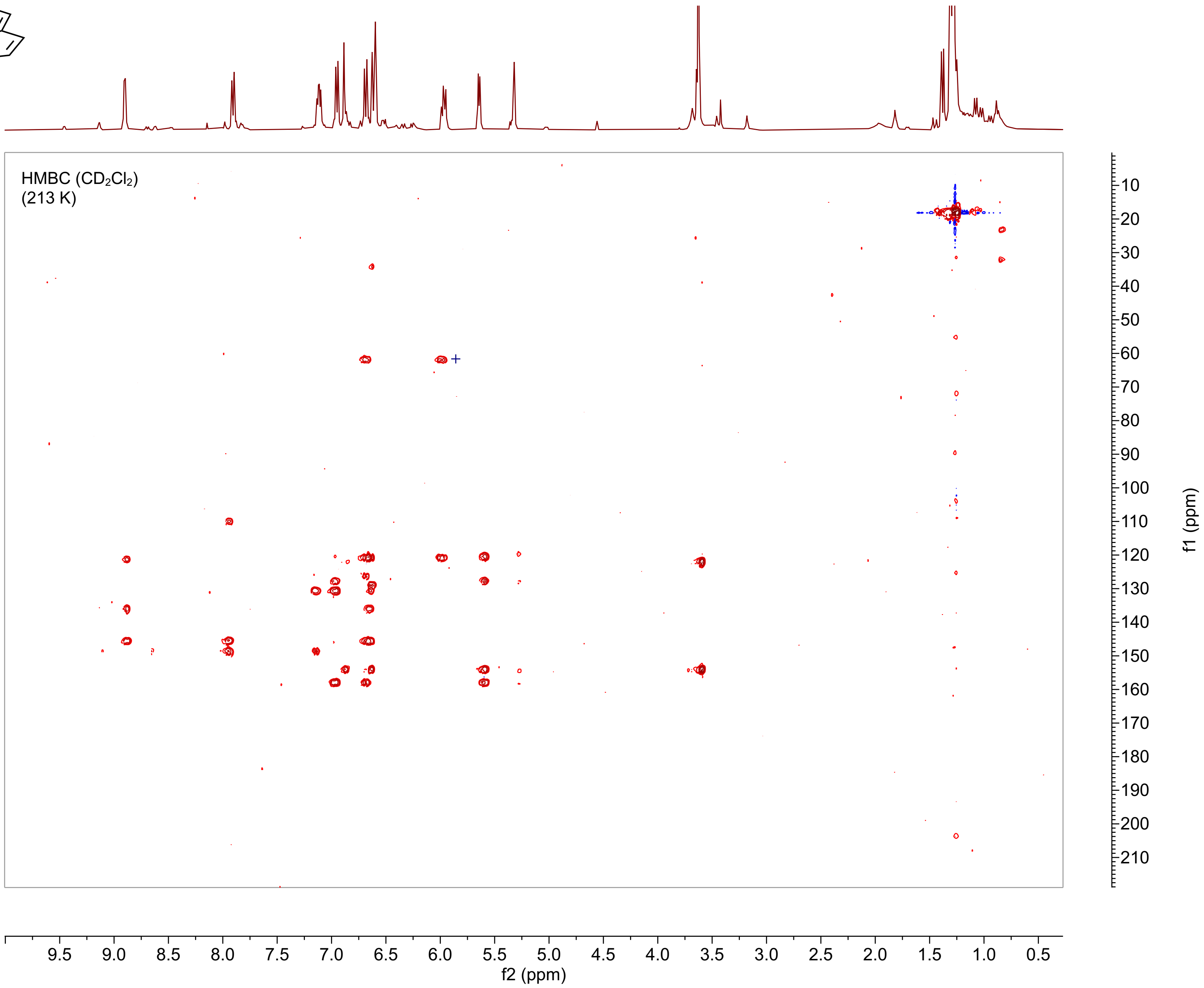

Figure S23. ^{1}H NMR spectrum of compound **2d** in CD_2Cl_2.

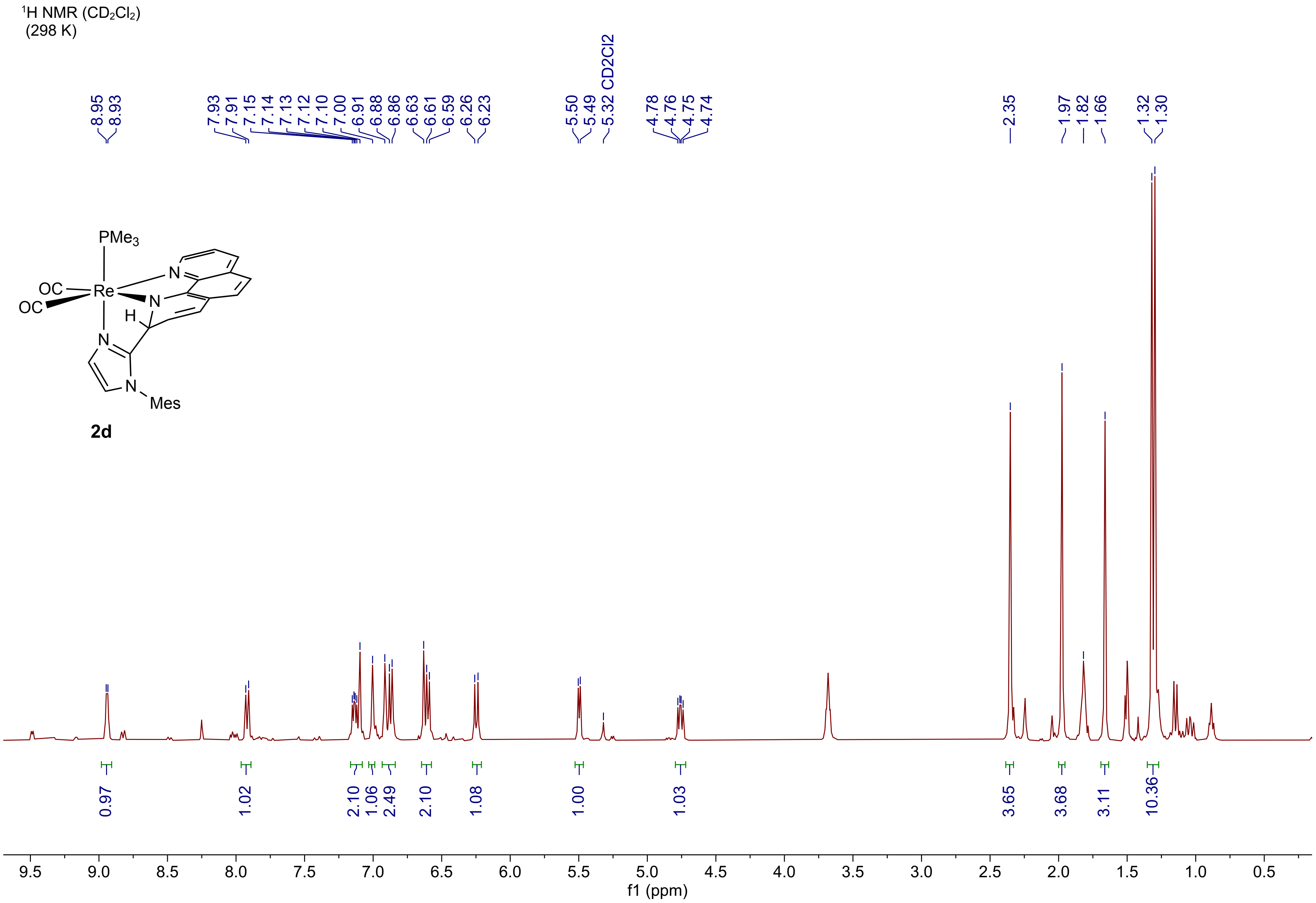

Figure S24. $^{13}C\{^1H\}$ NMR spectrum of compound **2d** in CD_2Cl_2.

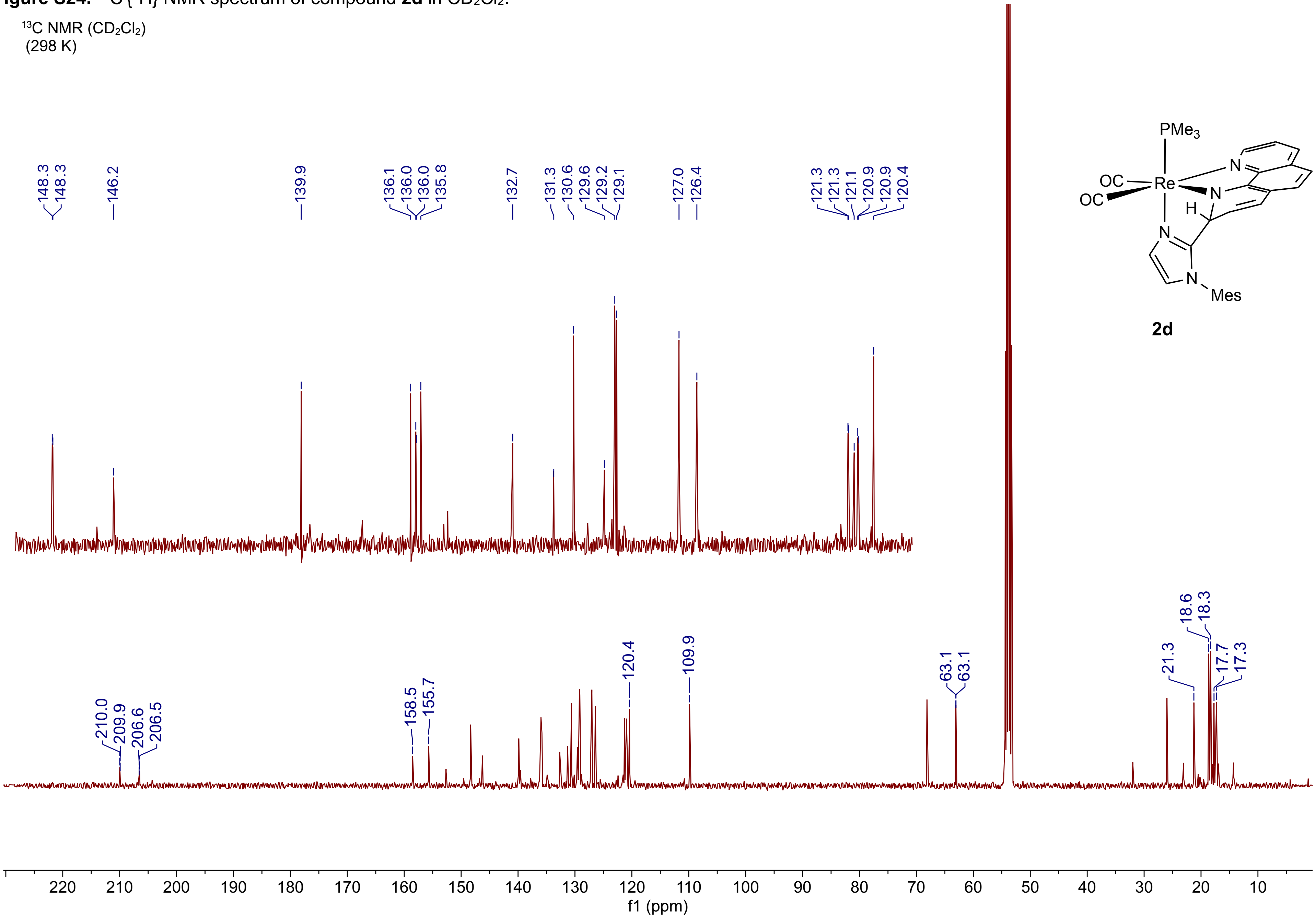

Figure S25. $^{31}P\{^1H\}$ NMR spectrum of compound **2d** in CD_2Cl_2.

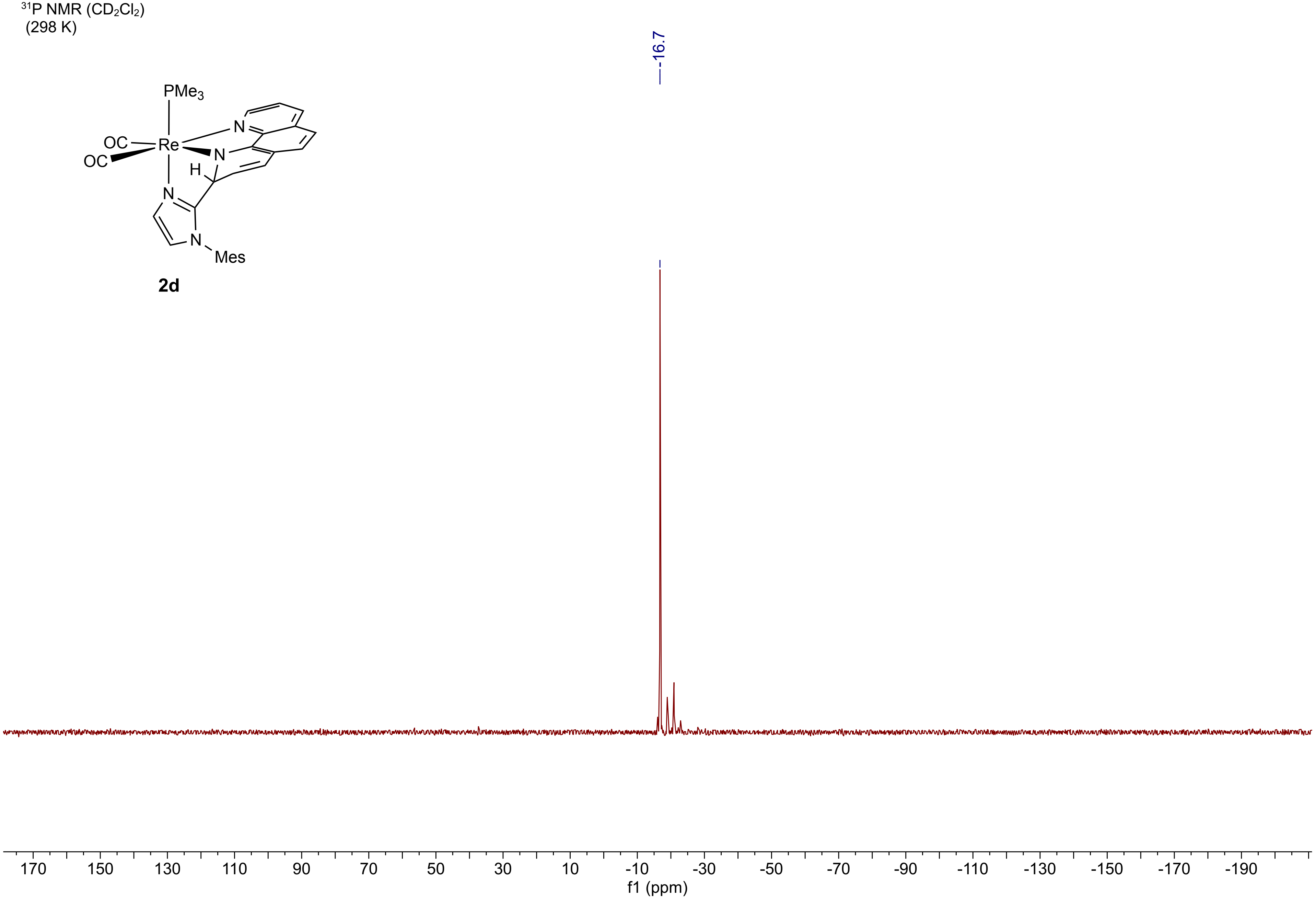

Figure S26. ^{1}H-^{1}H COSY NMR spectrum of compound **2d** in CD_2Cl_2.

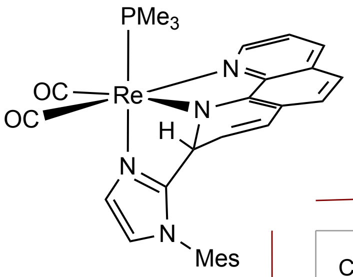

2d

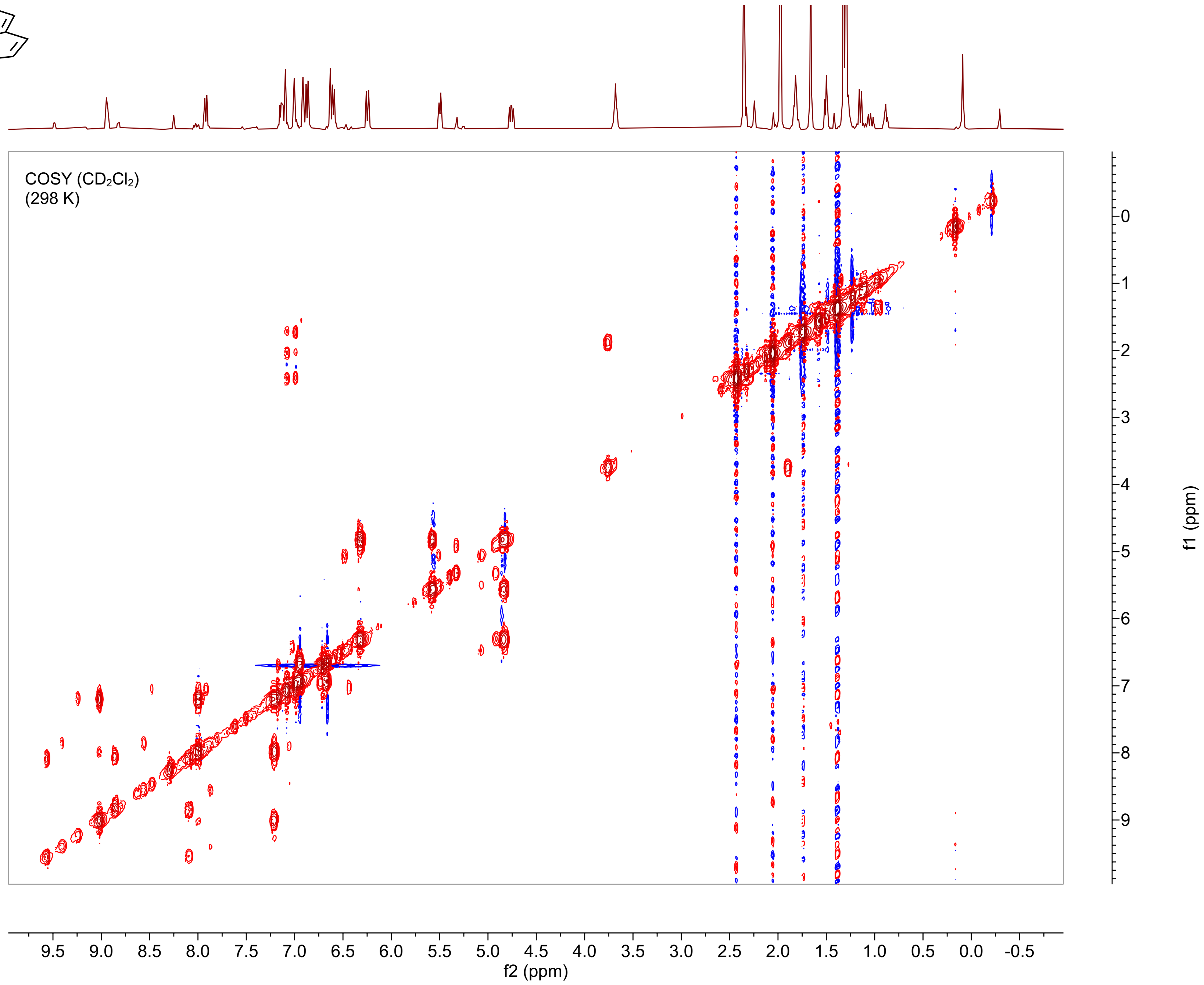

Figure S27. ^{1}H-^{13}C HSQC NMR spectrum of compound **2d** in CD_2Cl_2.

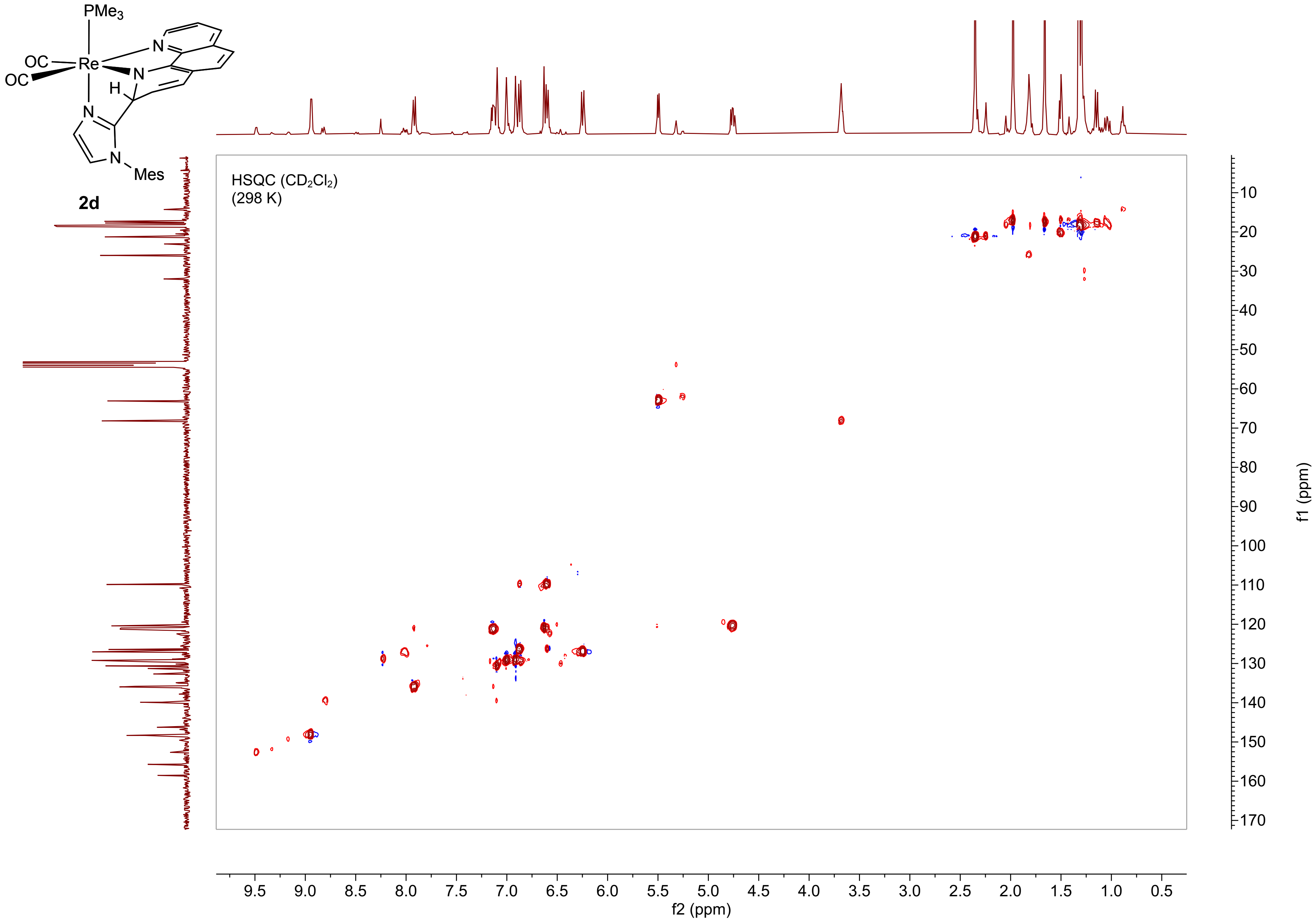

Figure S28. ^{1}H-^{13}C HMBC NMR spectrum of compound **2d** in CD_2Cl_2.

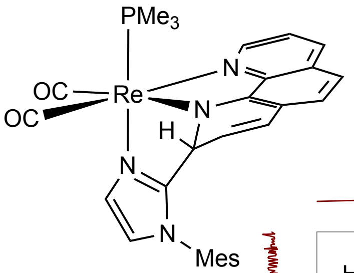

2d

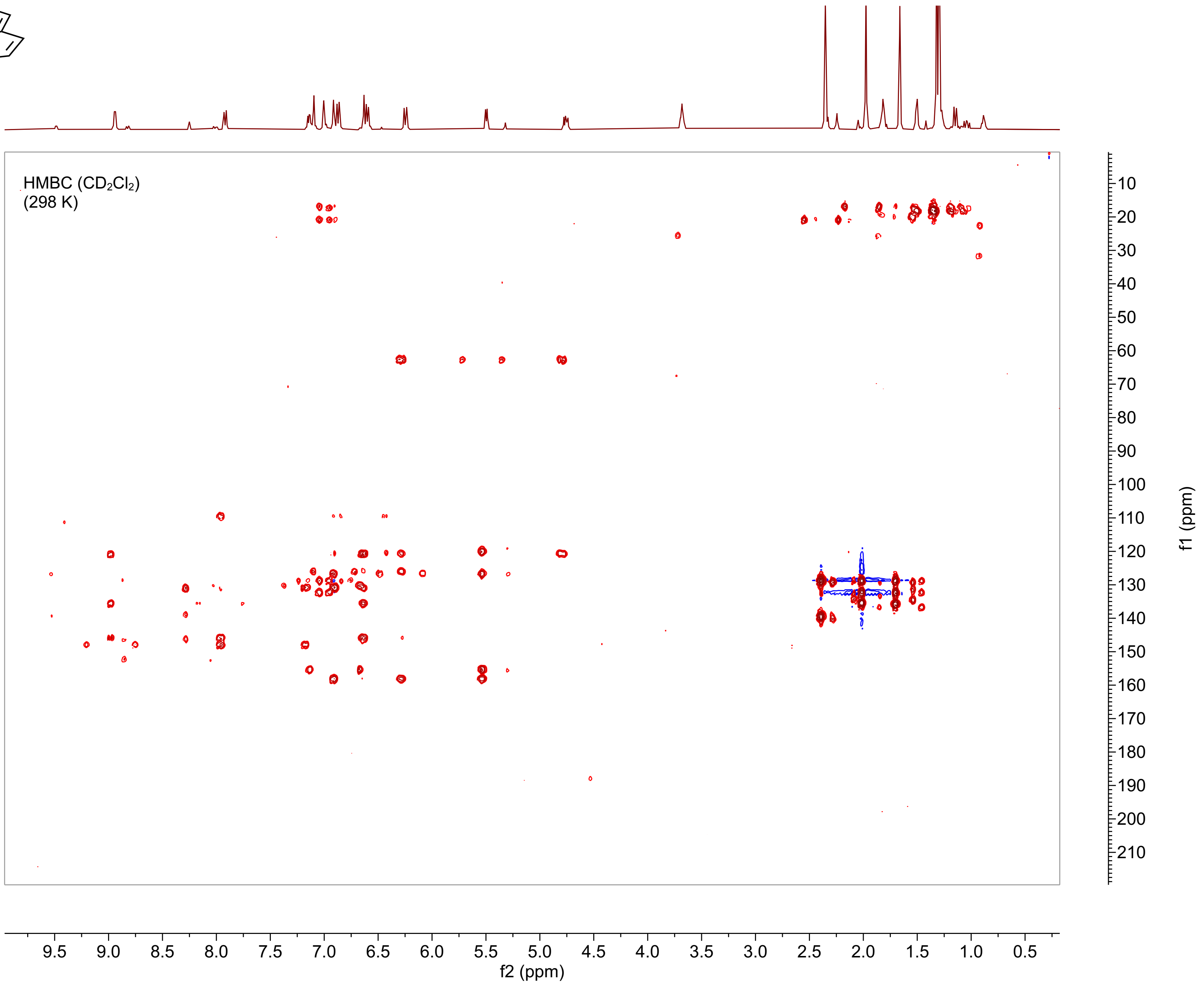

Figure S29. ^{1}H NMR spectrum of compound **3b** in CD_2Cl_2.

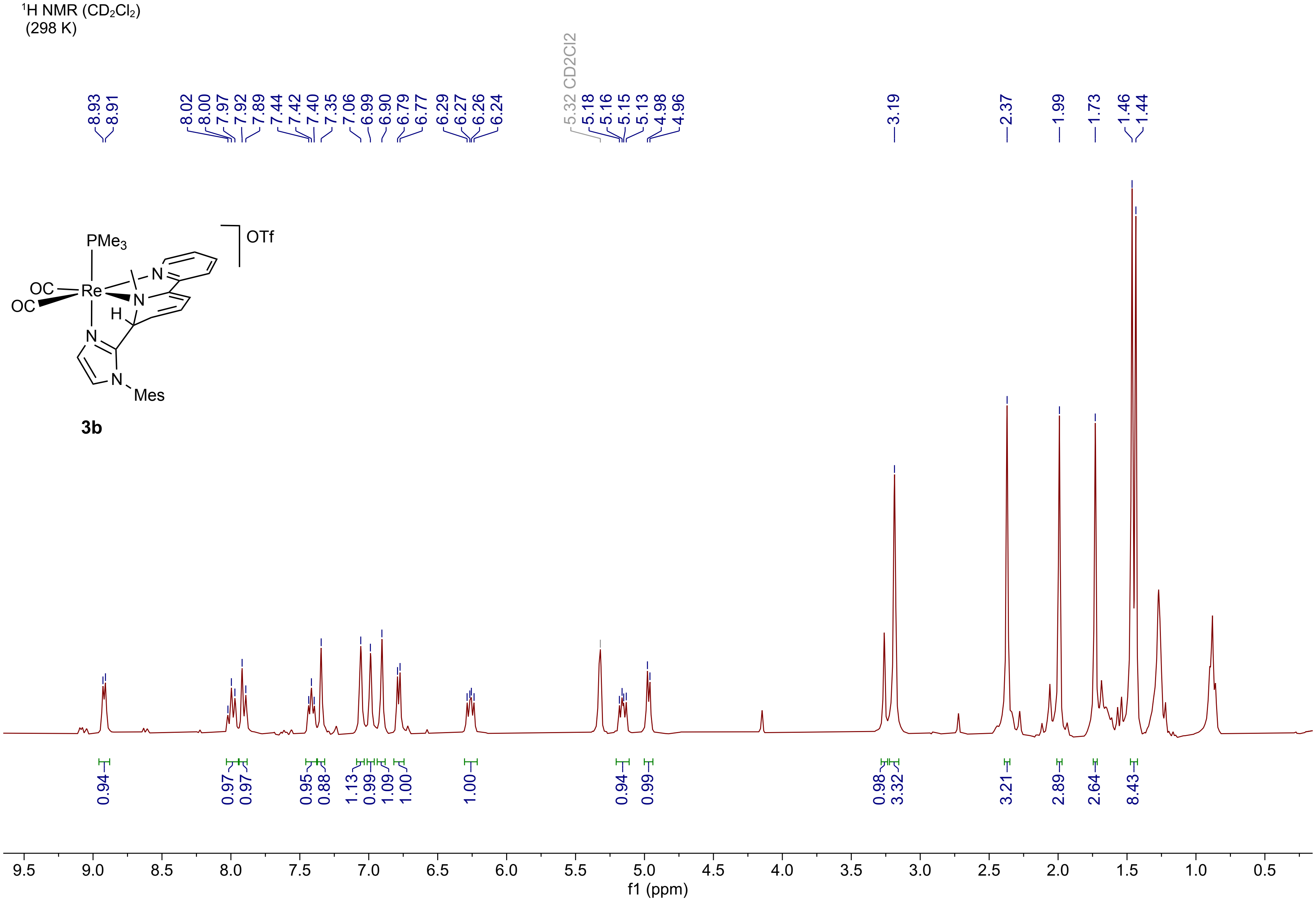

Figure S30. $^{13}C\{^{1}H\}$ NMR spectrum of compound **3b** in CD_2Cl_2.

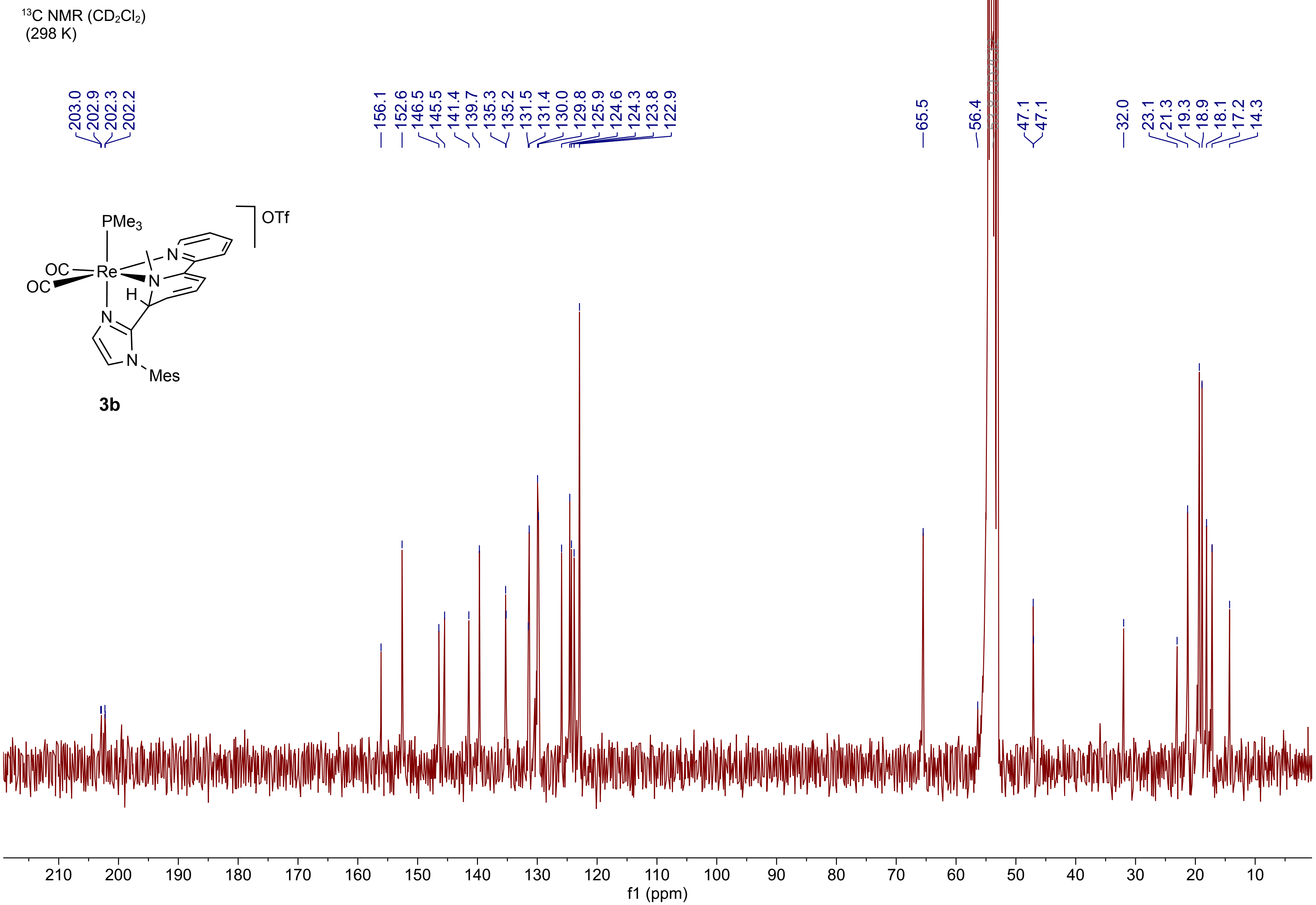

Figure S31. $^{31}P\{^{1}H\}$ NMR spectrum of compound **3b** in CD_2Cl_2.

^{31}P NMR (CD_2Cl_2)
(298 K)

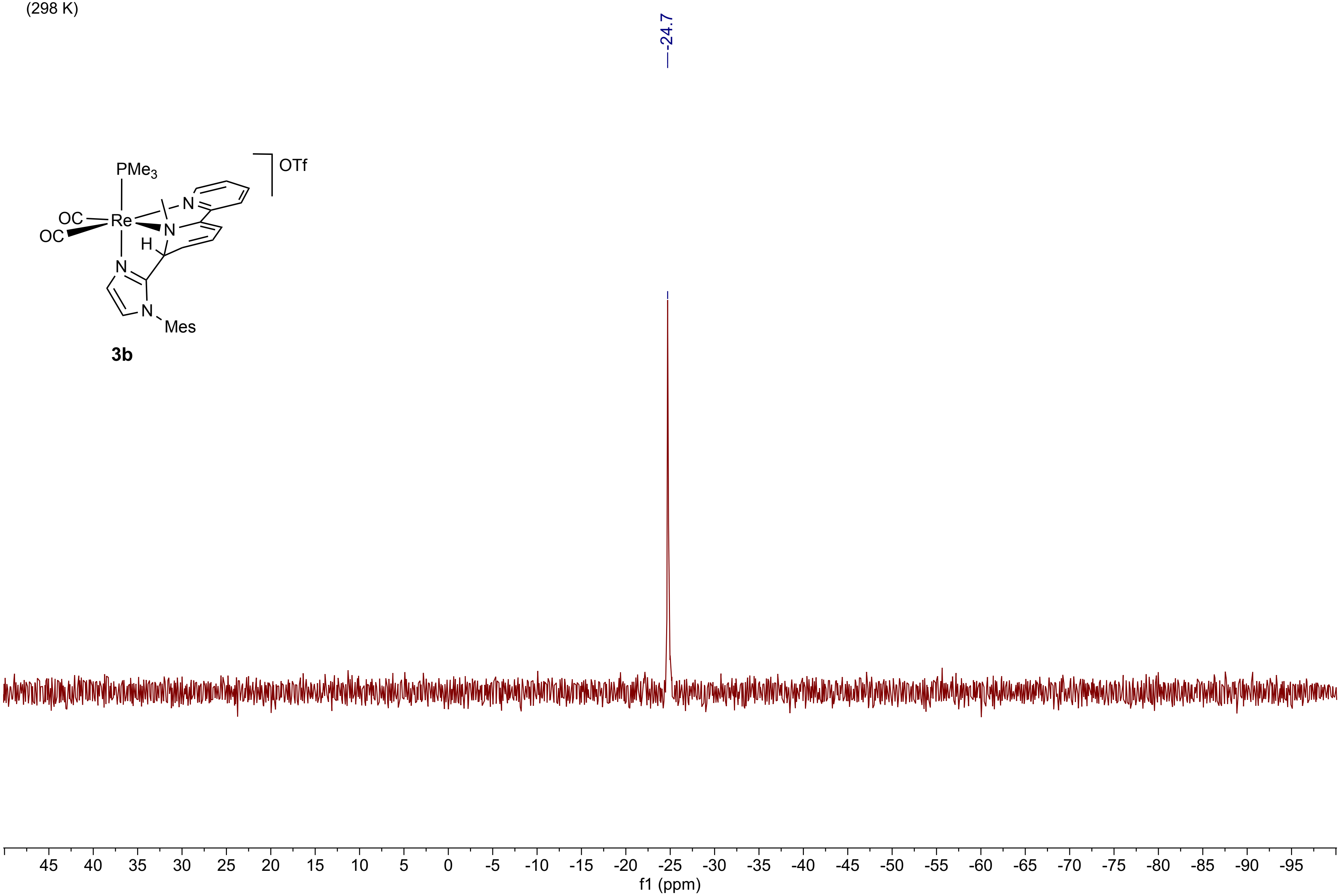

Figure S32. ^{1}H-^{1}H COSY NMR spectrum of compound **3b** in CD_2Cl_2.

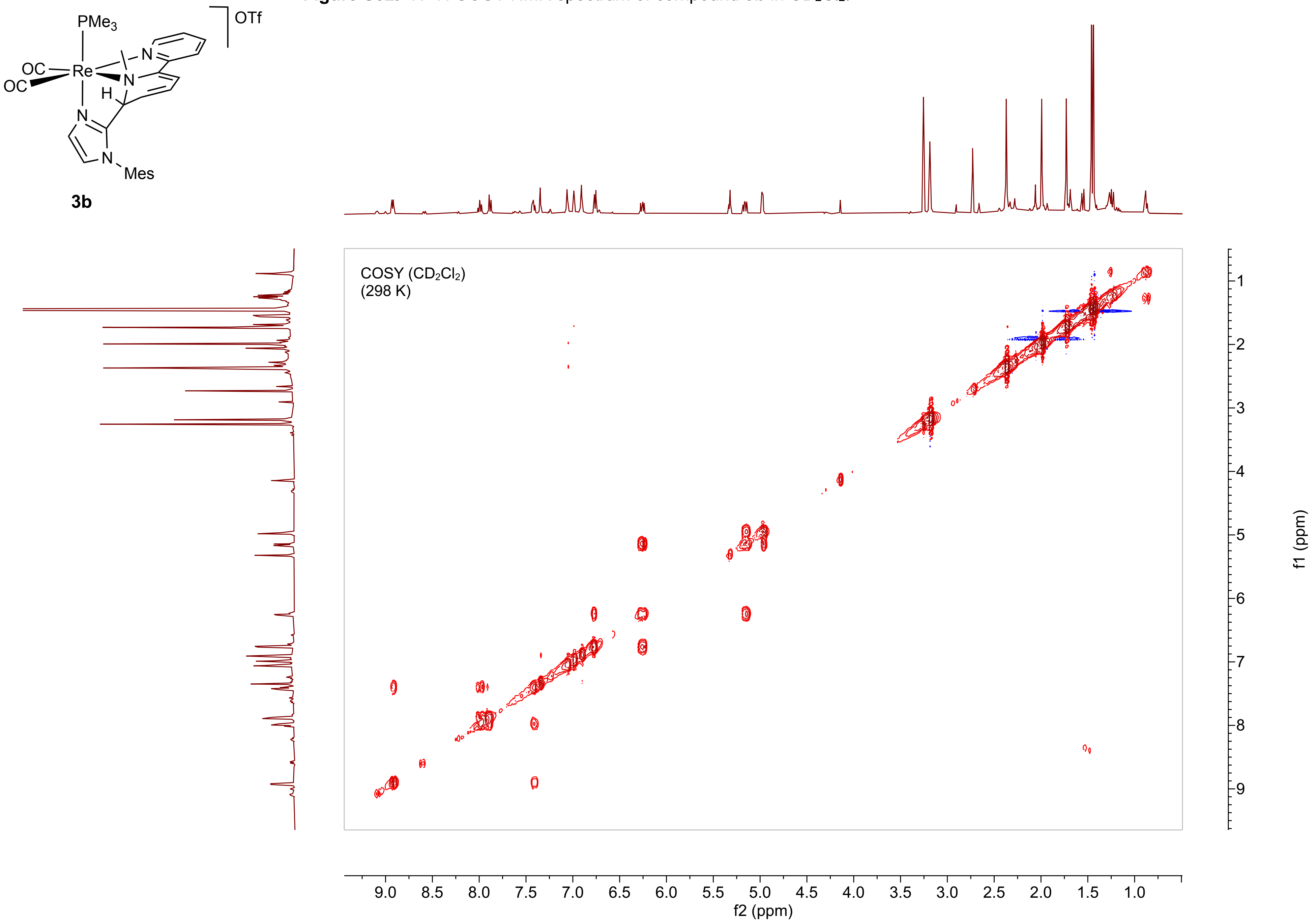

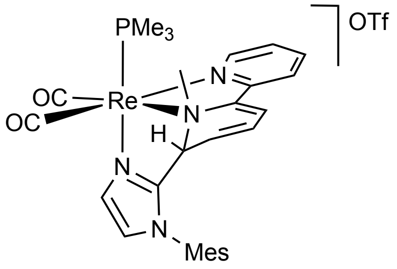

3b

Figure S33. ^{1}H-^{13}C HSQC NMR spectrum of compound **3b** in CD_2Cl_2.

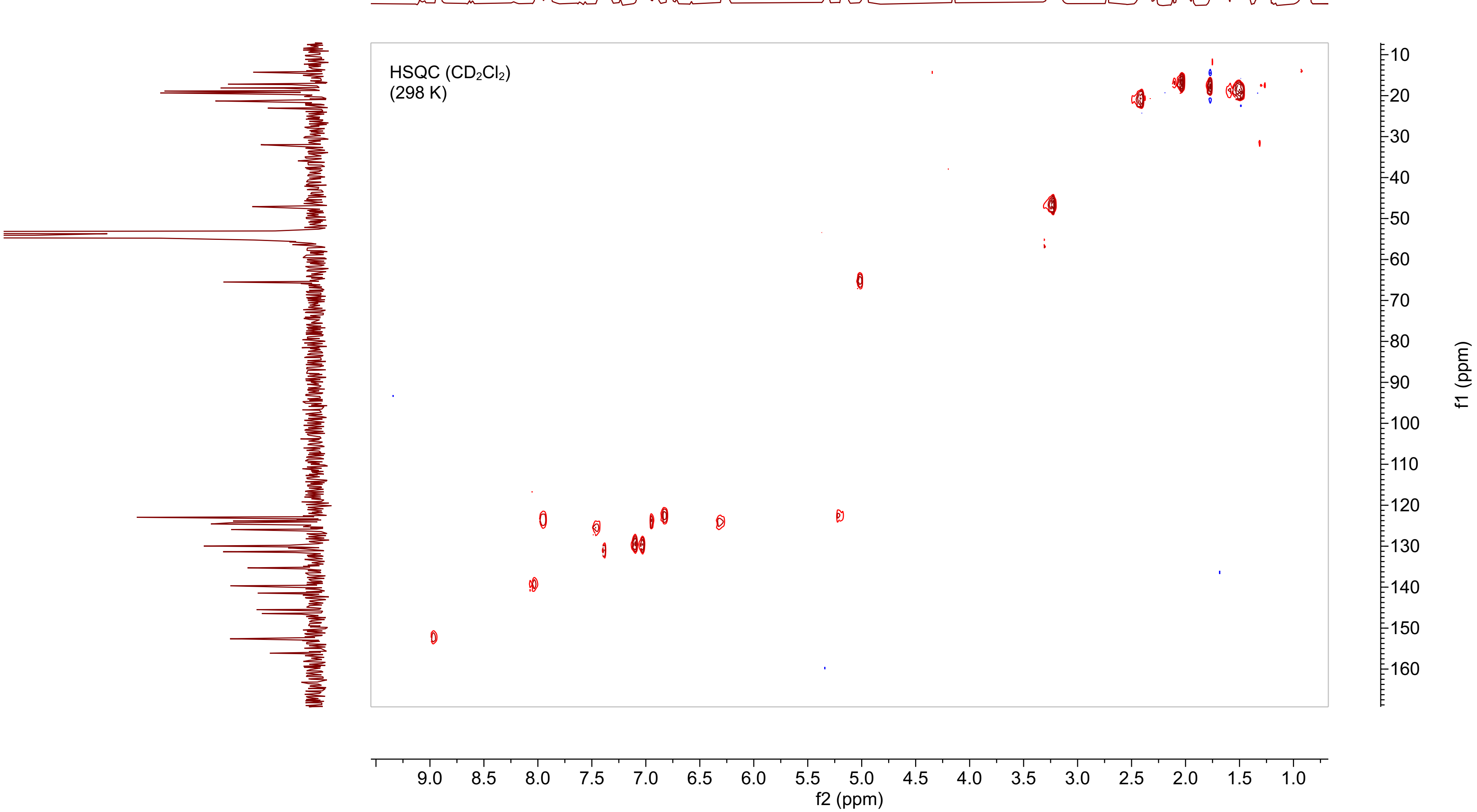

Figure S34. ^{1}H NMR spectrum of compound **3c** in CD_2Cl_2.

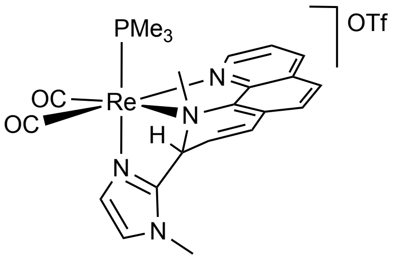

3c

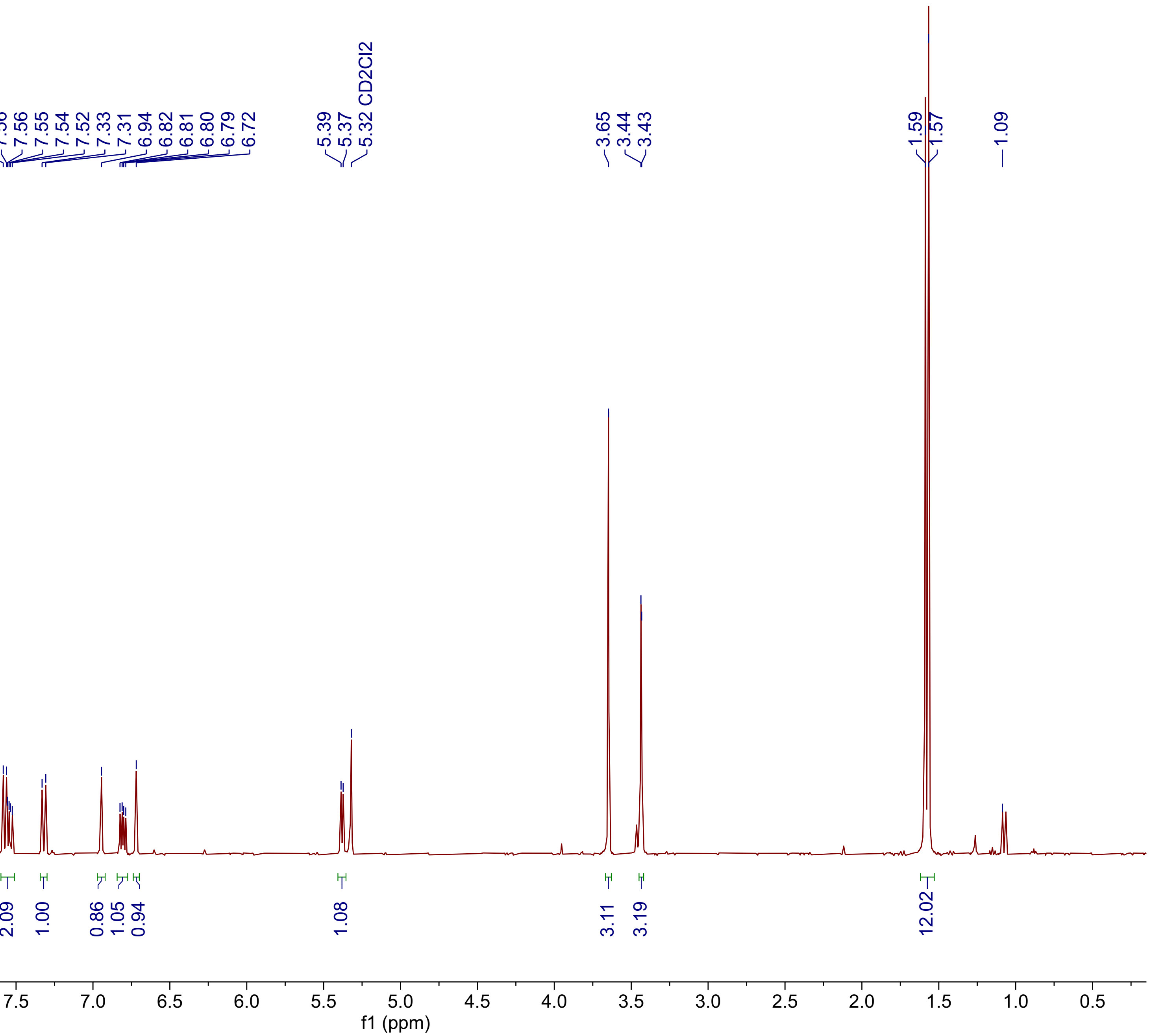

Figure S35. $^{13}C\{^{1}H\}$ NMR spectrum of compound **3c** in CD_2Cl_2.

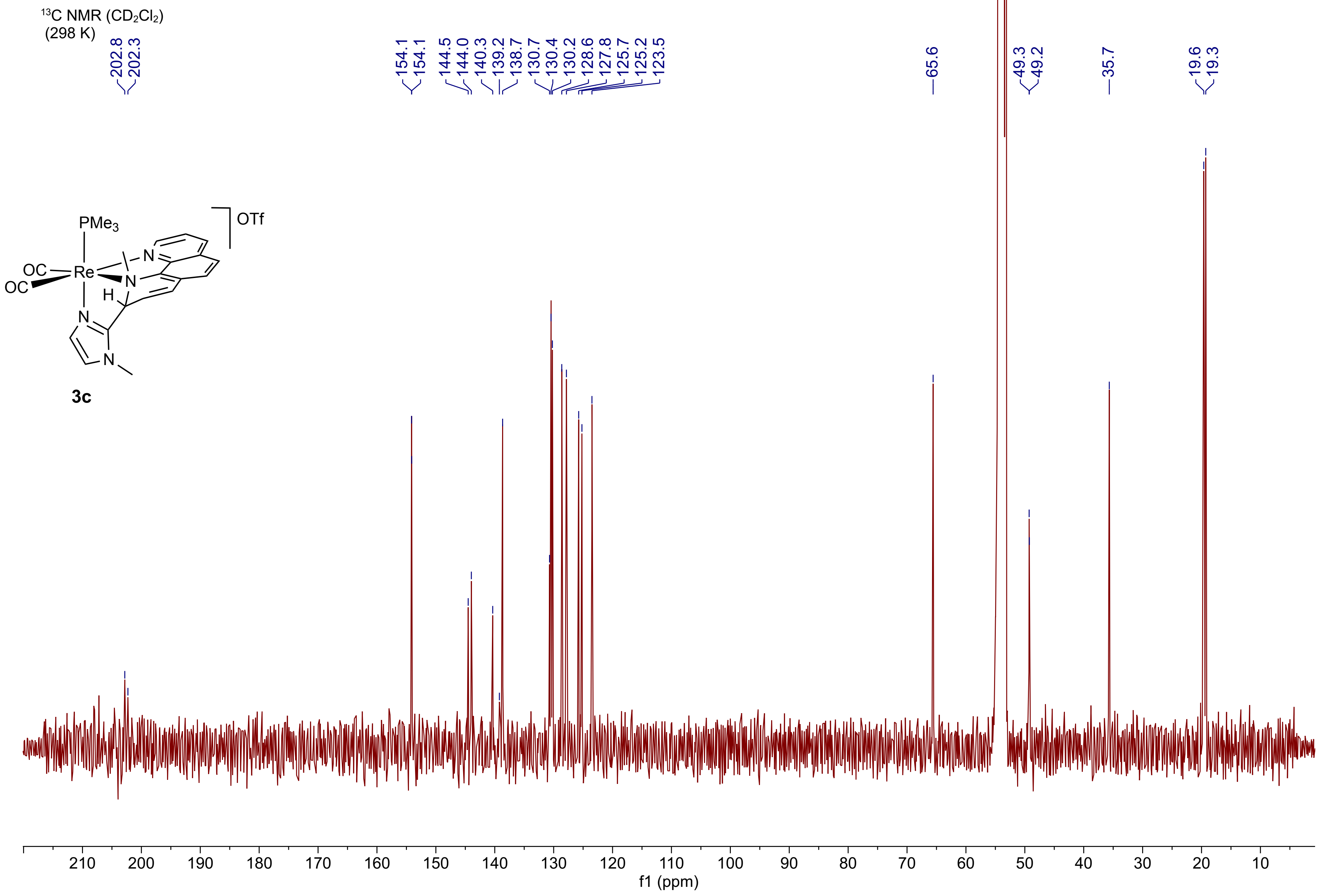

Figure S36. $^{31}P\{^1H\}$ NMR spectrum of compound **3c** in CD_2Cl_2.

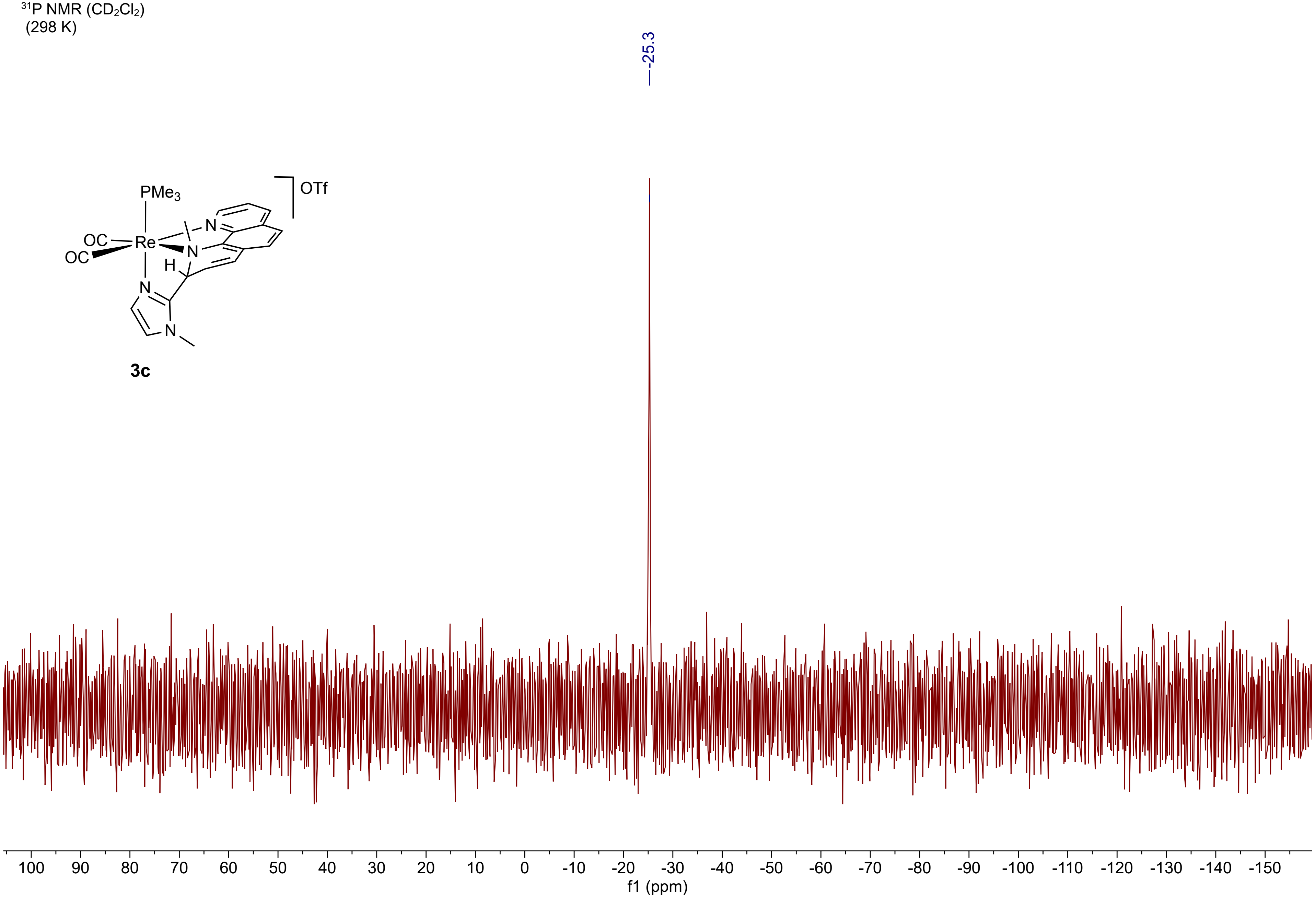

Figure S37. ^{1}H-^{1}H COSY NMR spectrum of compound **3c** in CD_2Cl_2.

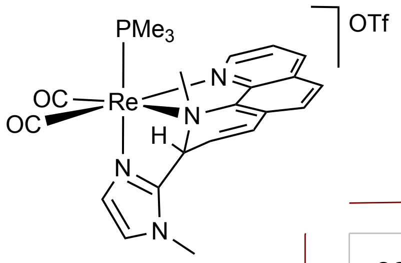

3c

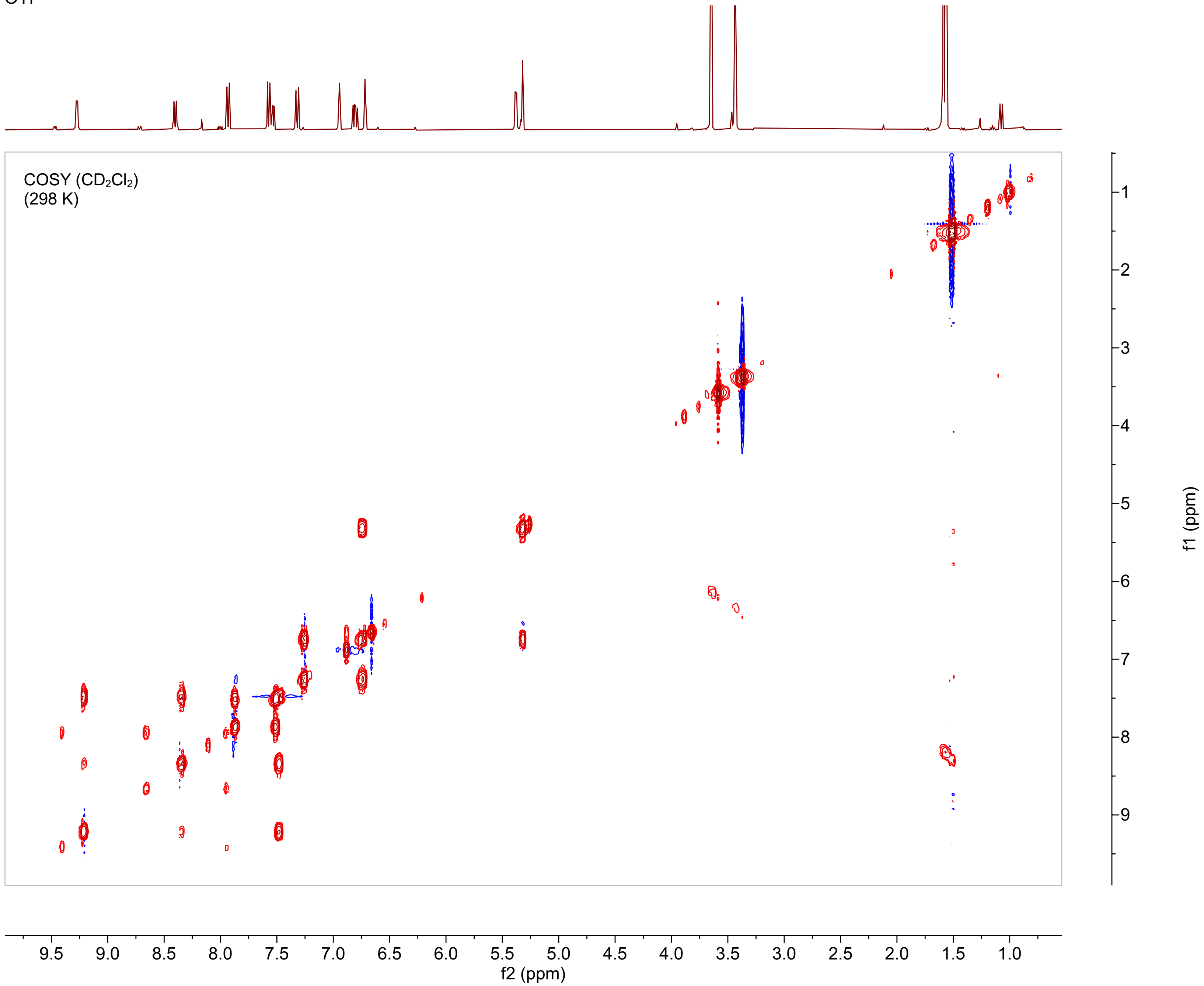

Figure S38. ^{1}H-^{13}C HSQC NMR spectrum of compound **3c** in CD_2Cl_2.

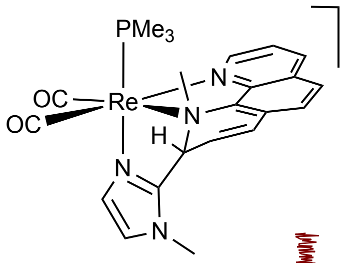

3c

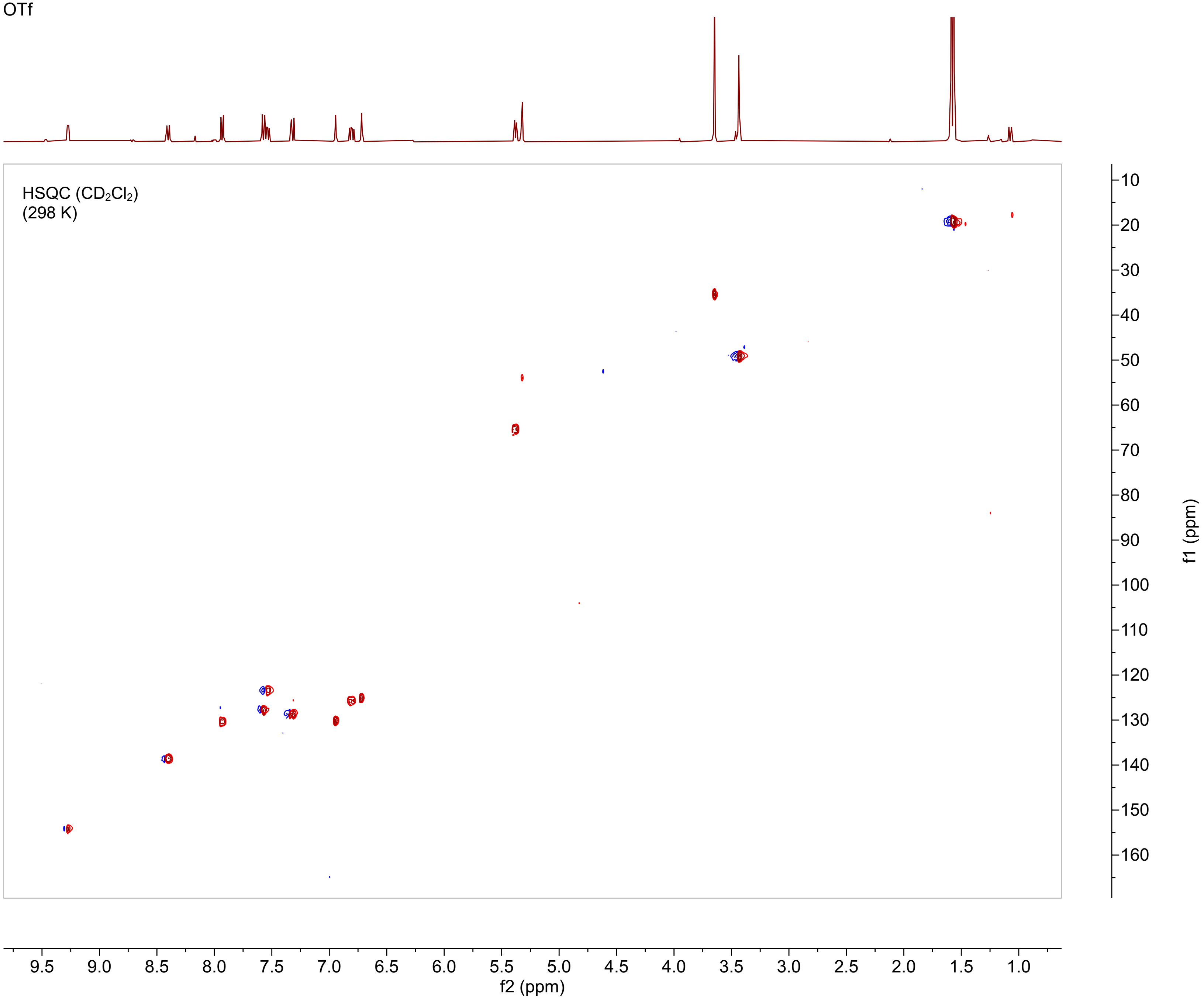

Figure S39. ^{1}H NMR spectrum of compound **3c'** in CD_2Cl_2.

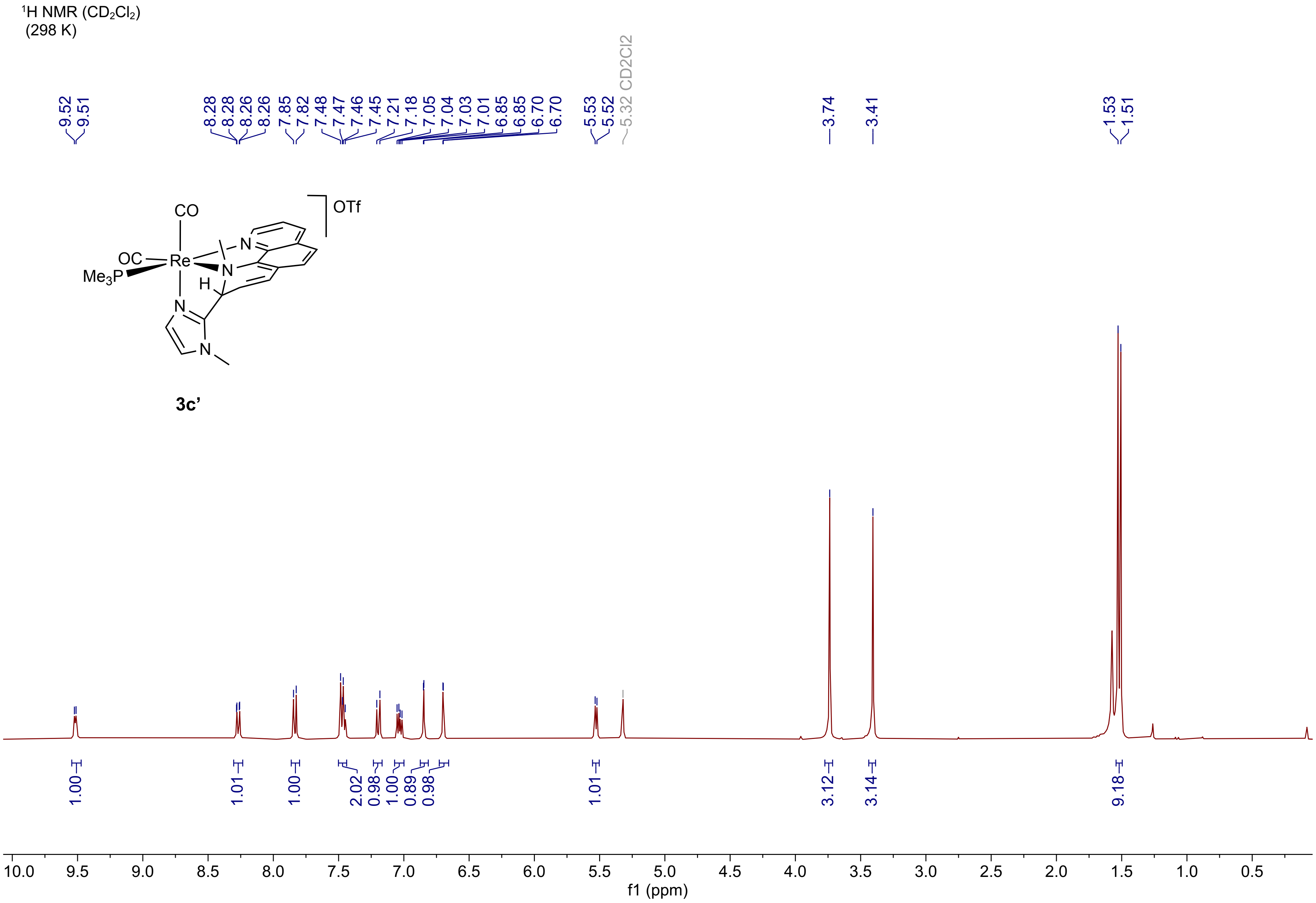

Figure S40. $^{13}C\{^1H\}$ NMR spectrum of compound **3c'** in CD_2Cl_2.

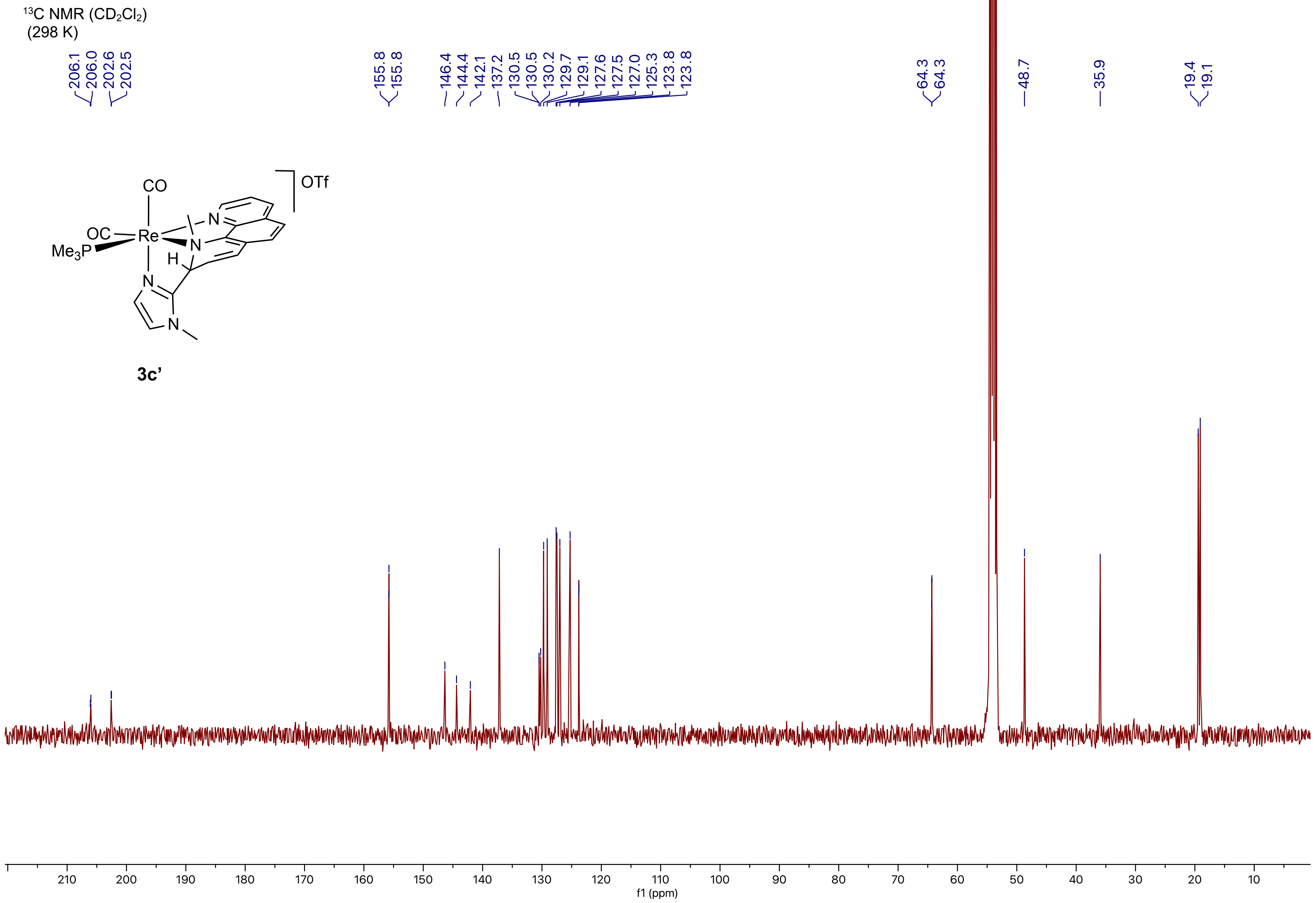

Figure S41. $^{31}P\{^{1}H\}$ NMR spectrum of compound **3c'** in CD_2Cl_2.

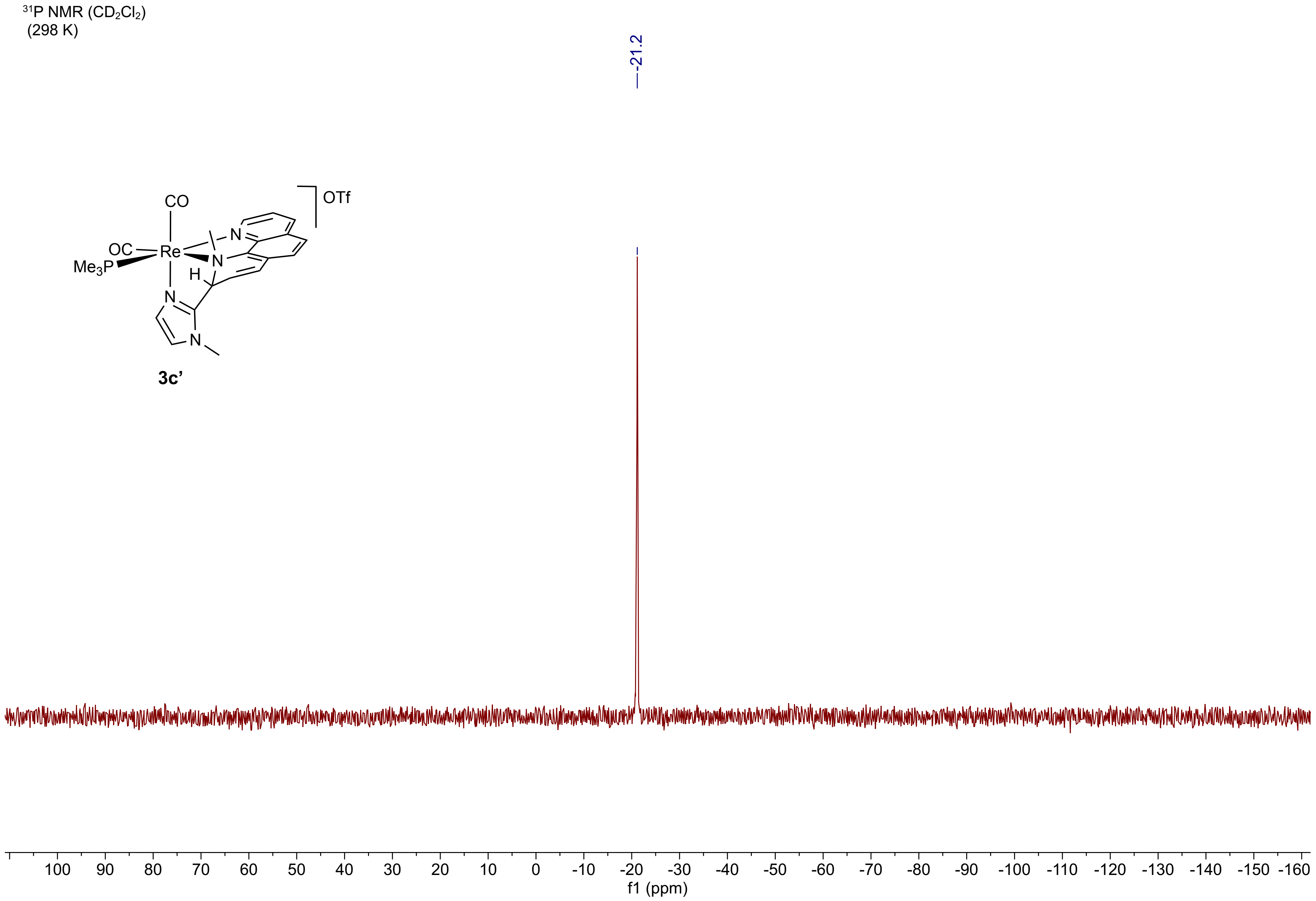

3c'

Figure S42. ^{1}H-^{1}H COSY NMR spectrum of compound **3c'** in CD_2Cl_2.

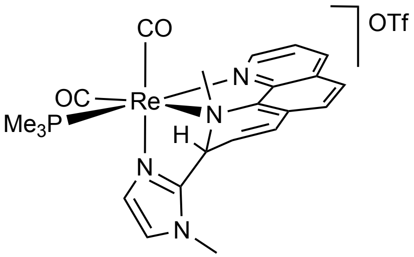

3c'

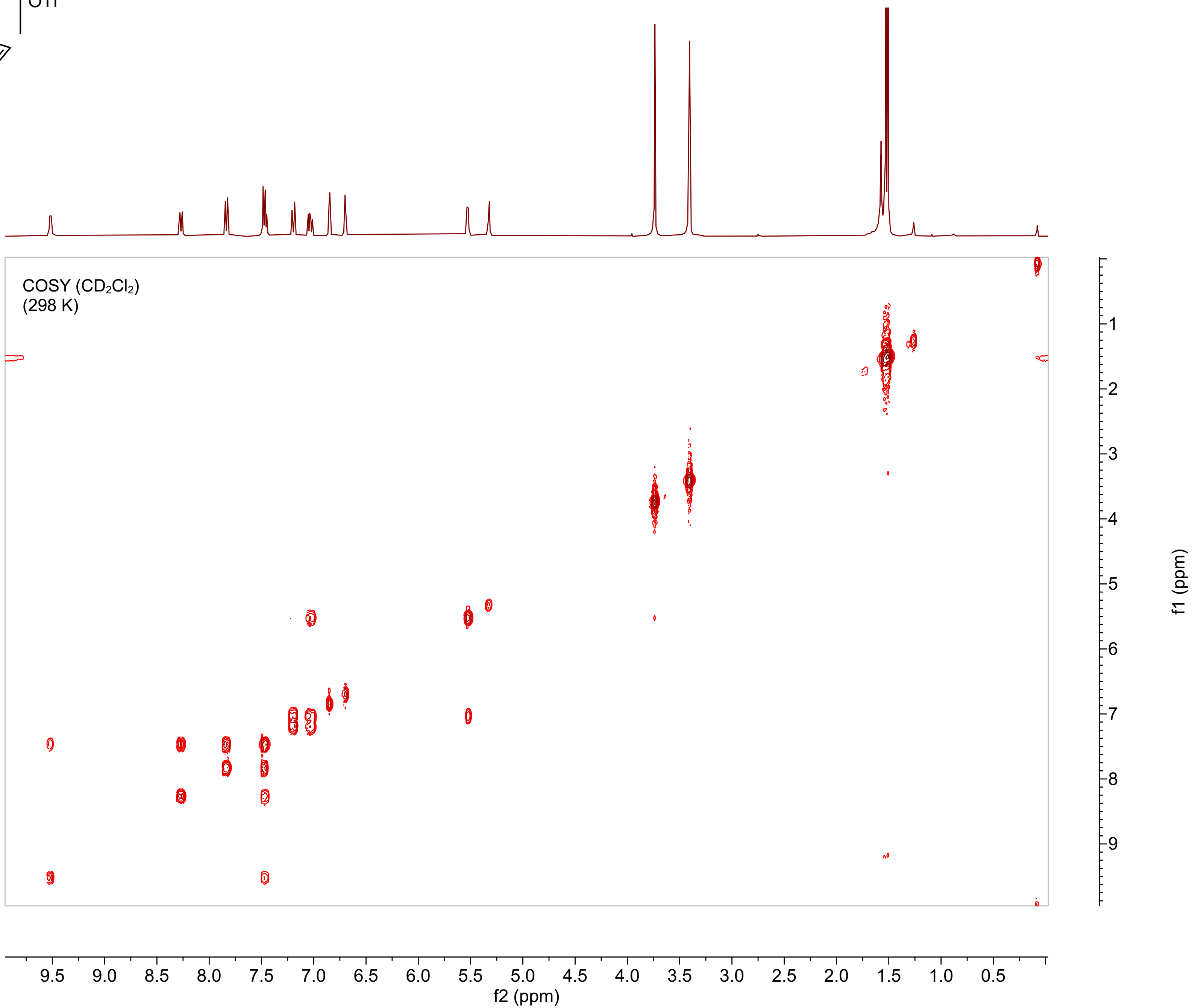

Figure S43. ^1H-^{13}C HSQC NMR spectrum of compound **3c'** in CD_2Cl_2.

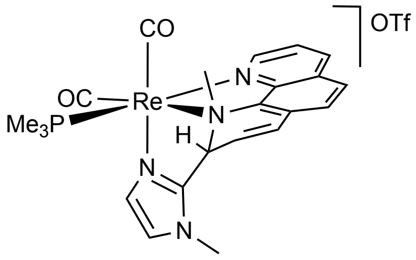

3c'

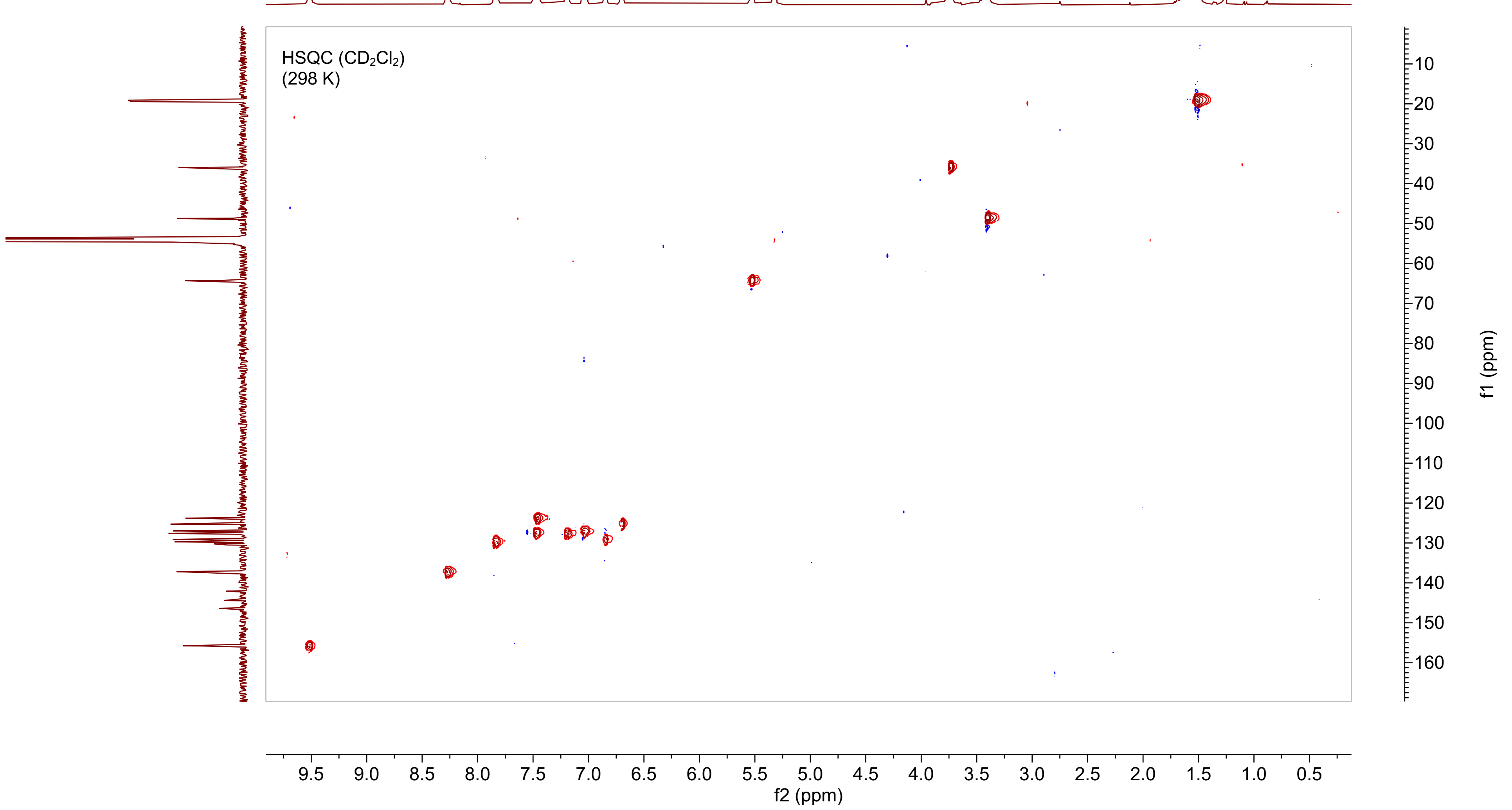

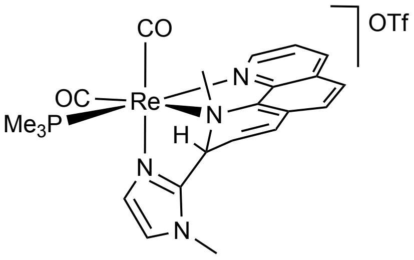

3c'

Figure S44. ^{1}H-^{13}C HMBC NMR spectrum of compound **3c'** in CD_2Cl_2.

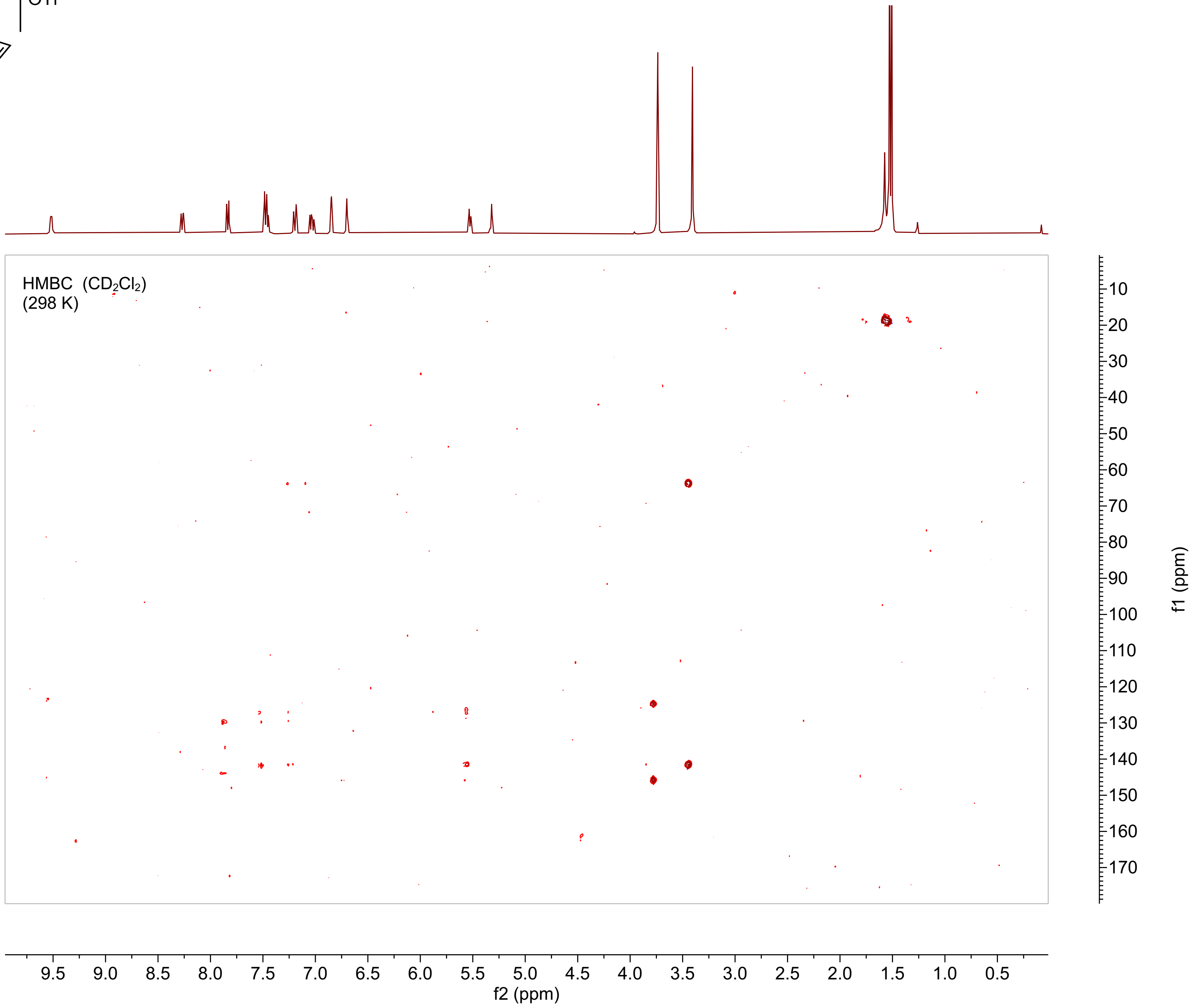

Figure S45. ^1H NMR spectrum of compound **3d** in CD_2Cl_2.

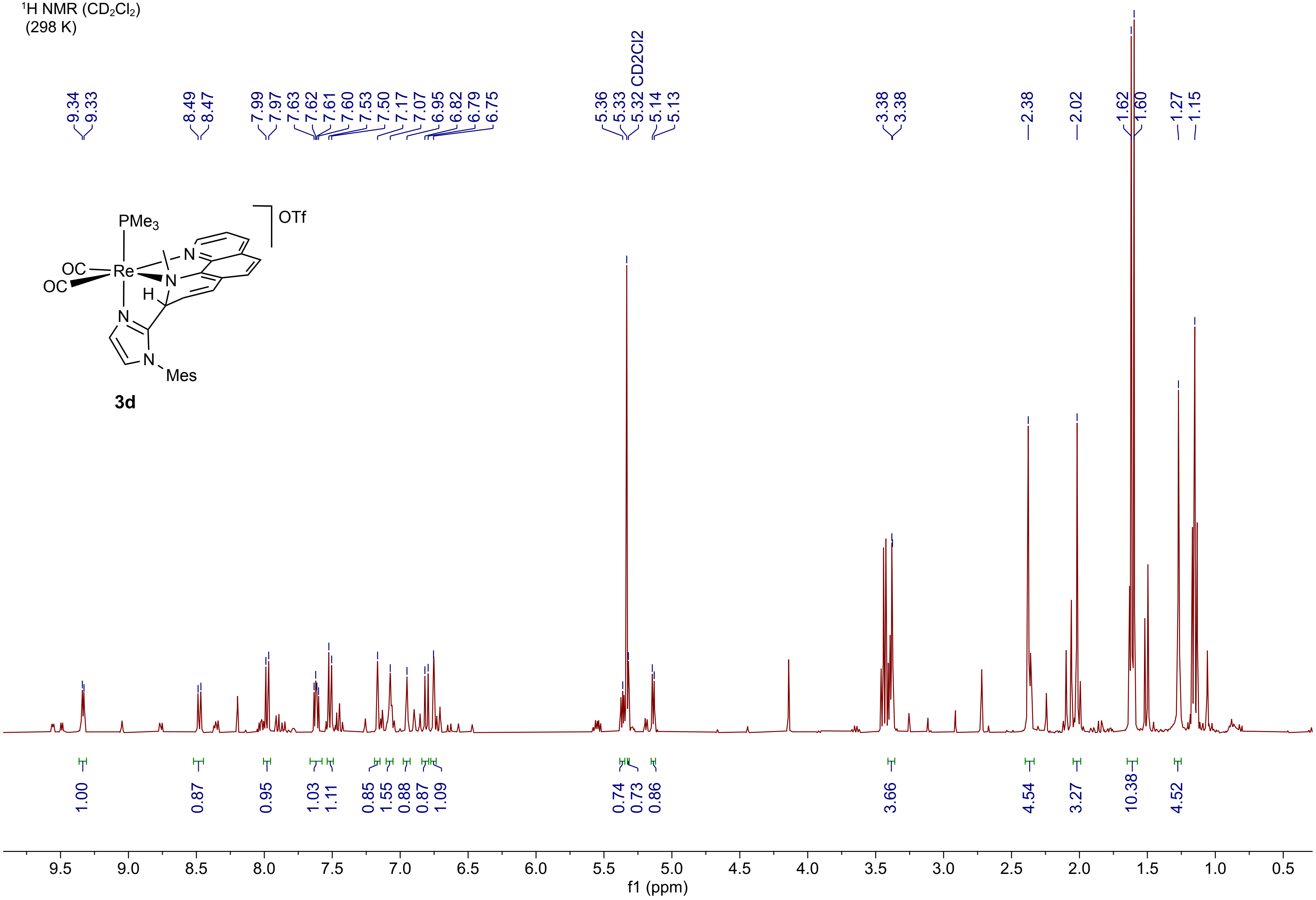

Figure S46. $^{13}C\{^1H\}$ NMR spectrum of compound **3d** in CD_2Cl_2.

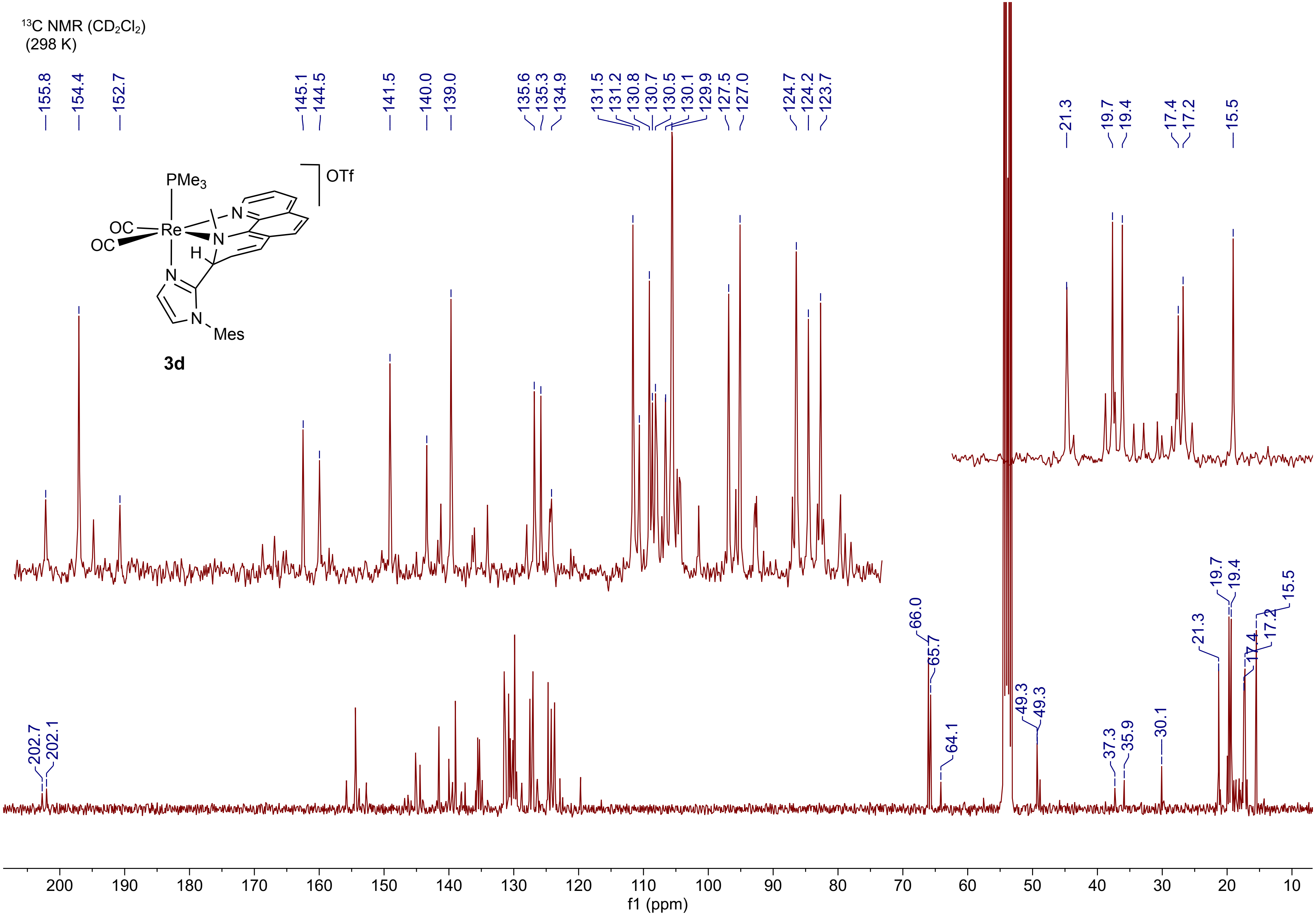

Figure S47. $^{31}P\{^1H\}$ NMR spectrum of compound **3d** in CD_2Cl_2.

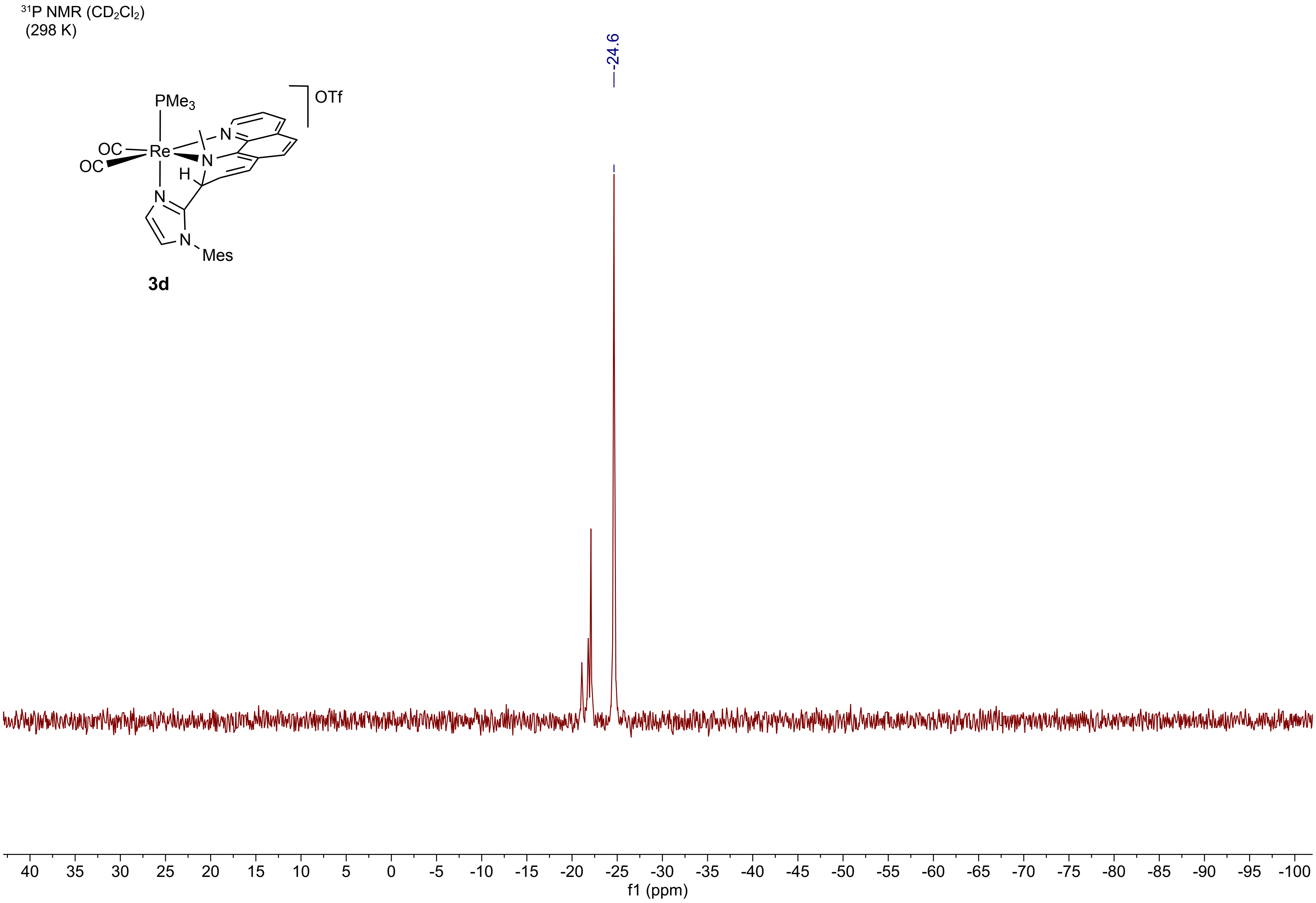

Figure S48. ^{1}H-^{1}H COSY NMR spectrum of compound **3d** in CD_2Cl_2.

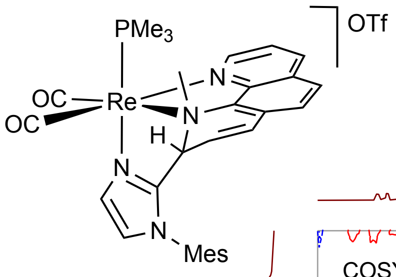

3d

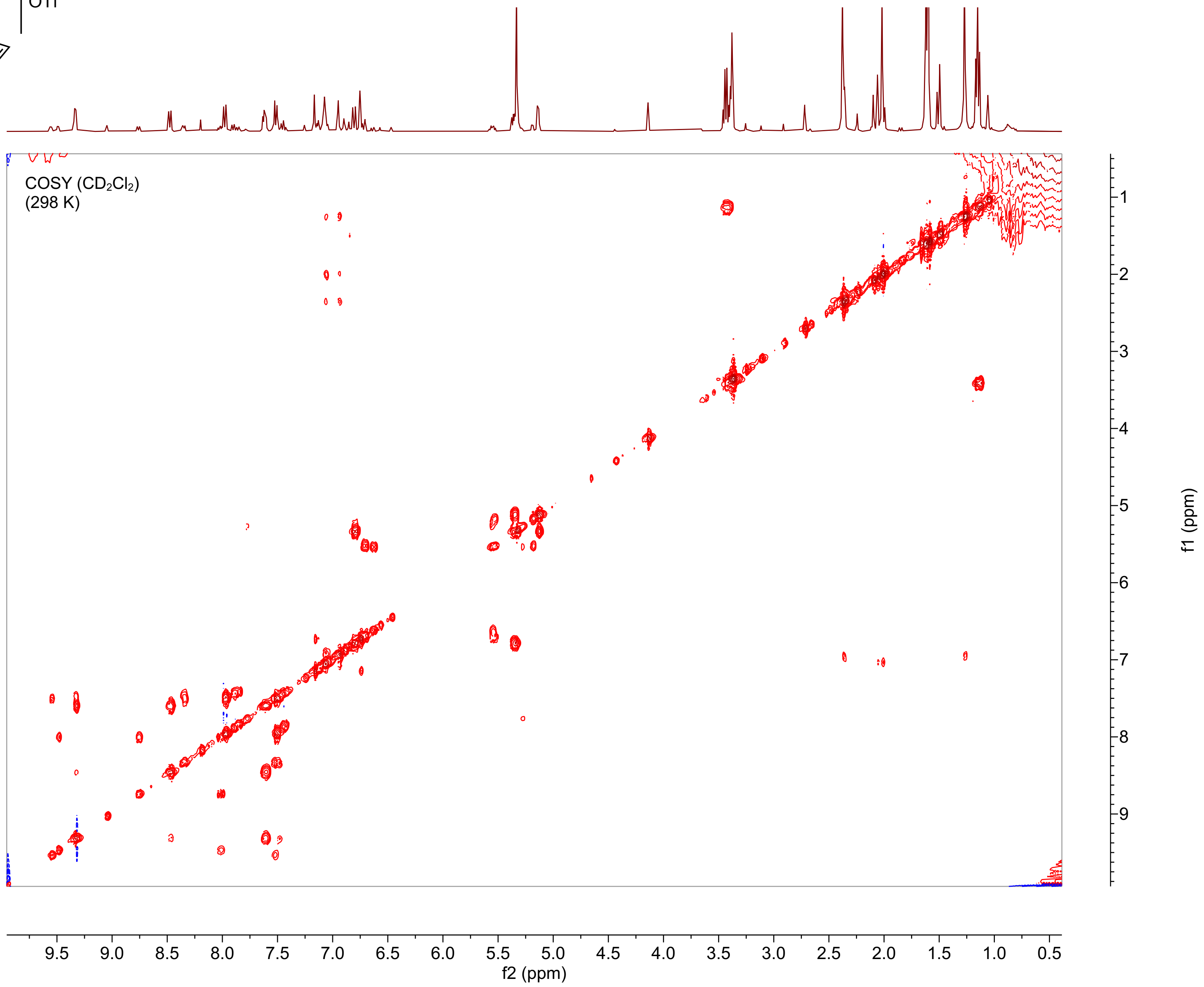

Figure S49. ^{1}H-^{13}C HSQC NMR spectrum of compound **3d** in CD_2Cl_2.

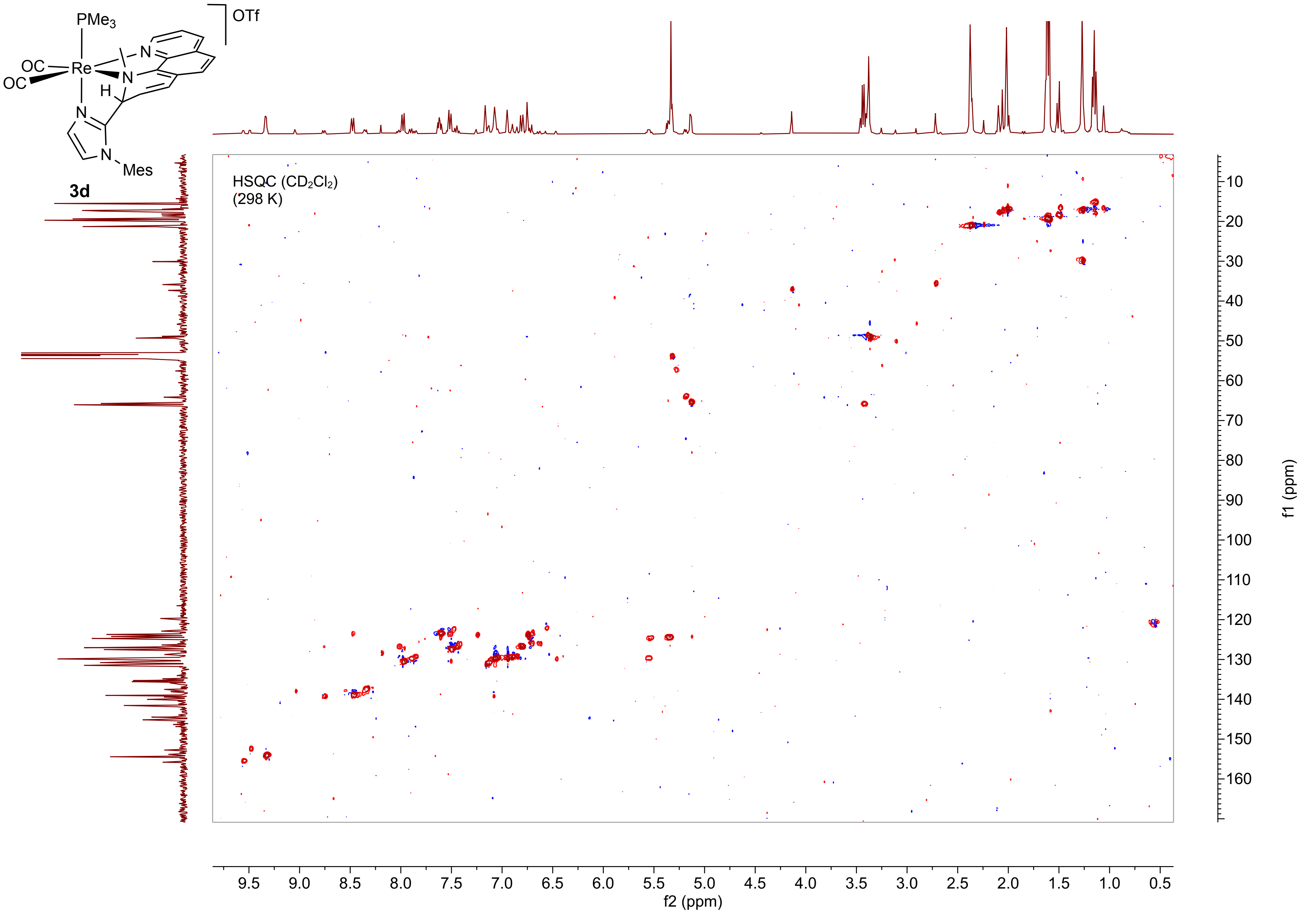

Figure S50. ^{1}H-^{13}C HMBC NMR spectrum of compound **3d** in CD_2Cl_2.

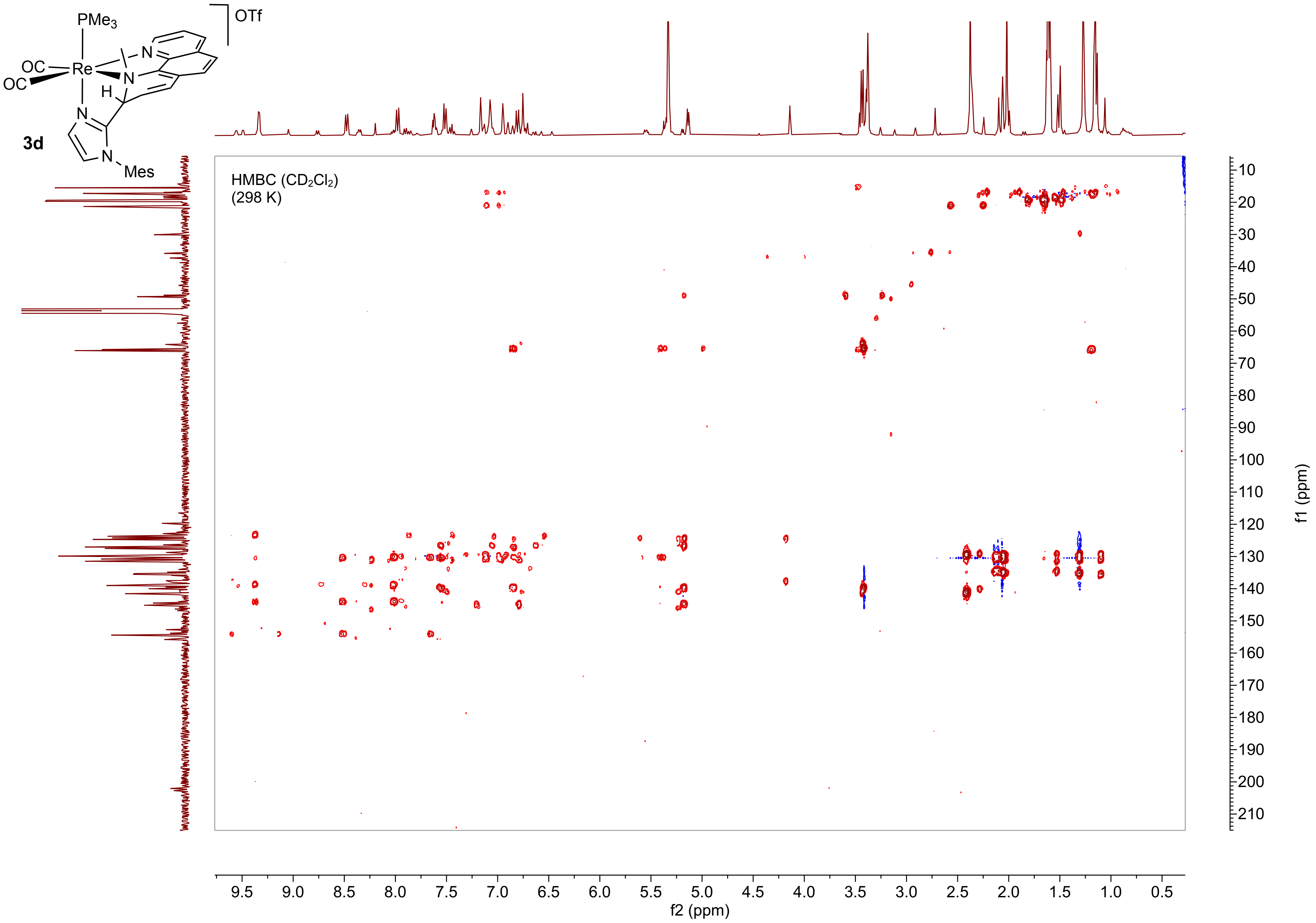

Figure S51. ^{1}H NMR spectrum of compound **9c** in CD_2Cl_2.

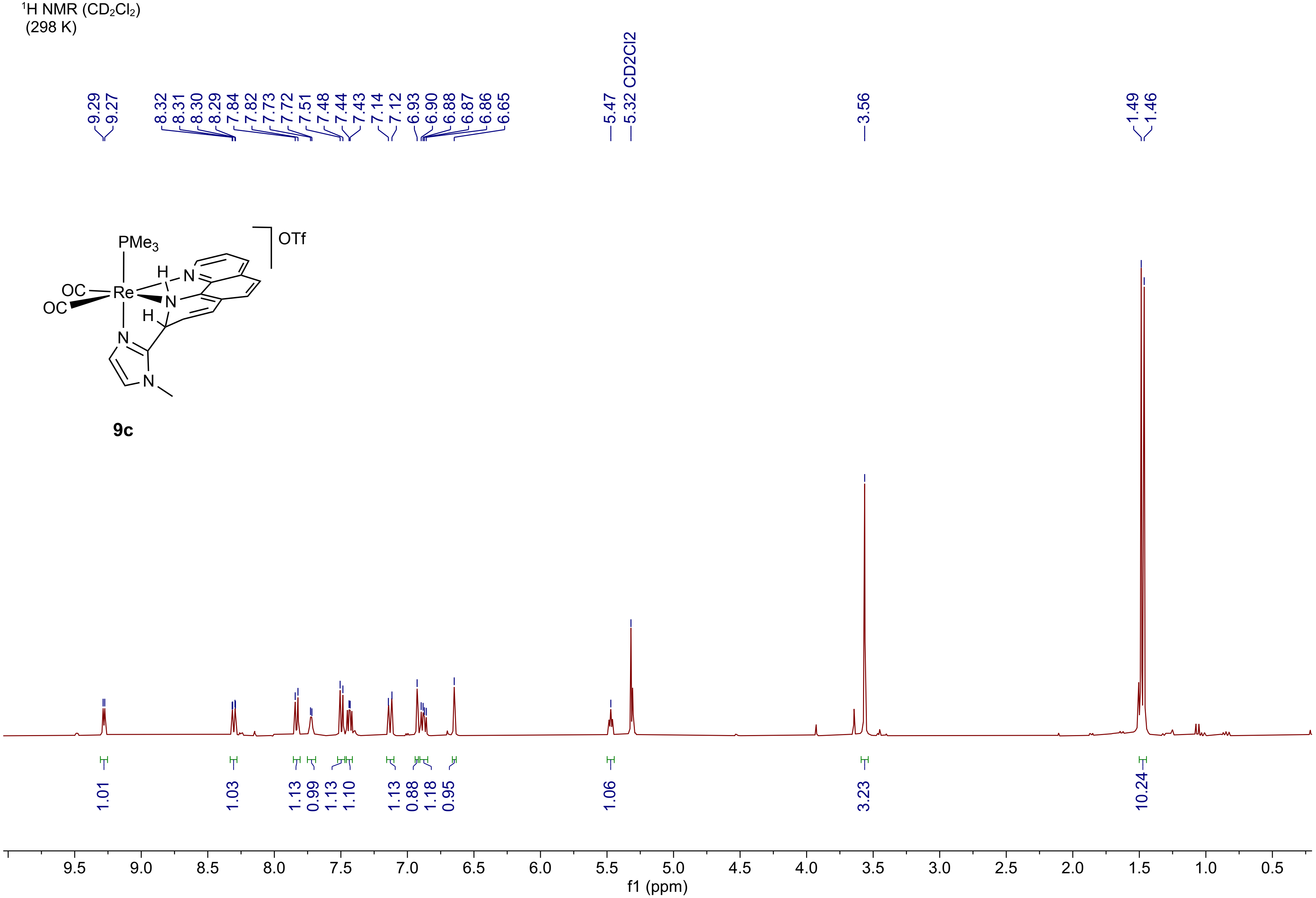

9c

Figure S52. $^{13}C\{^{1}H\}$ NMR spectrum of compound **9c** in CD_2Cl_2.

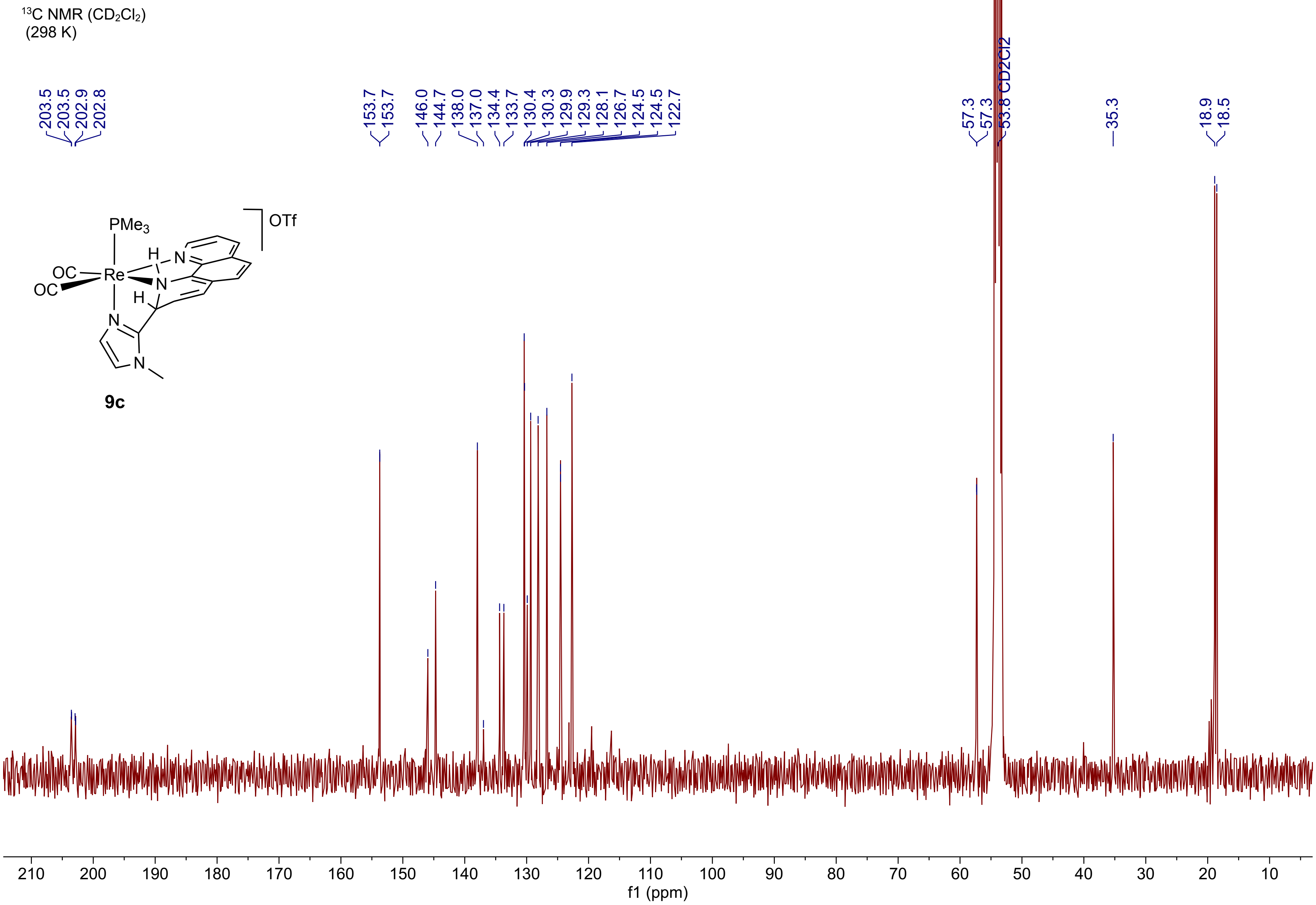

9c

Figure S53. $^{31}P\{^1H\}$ NMR spectrum of compound **9c** in CD_2Cl_2.

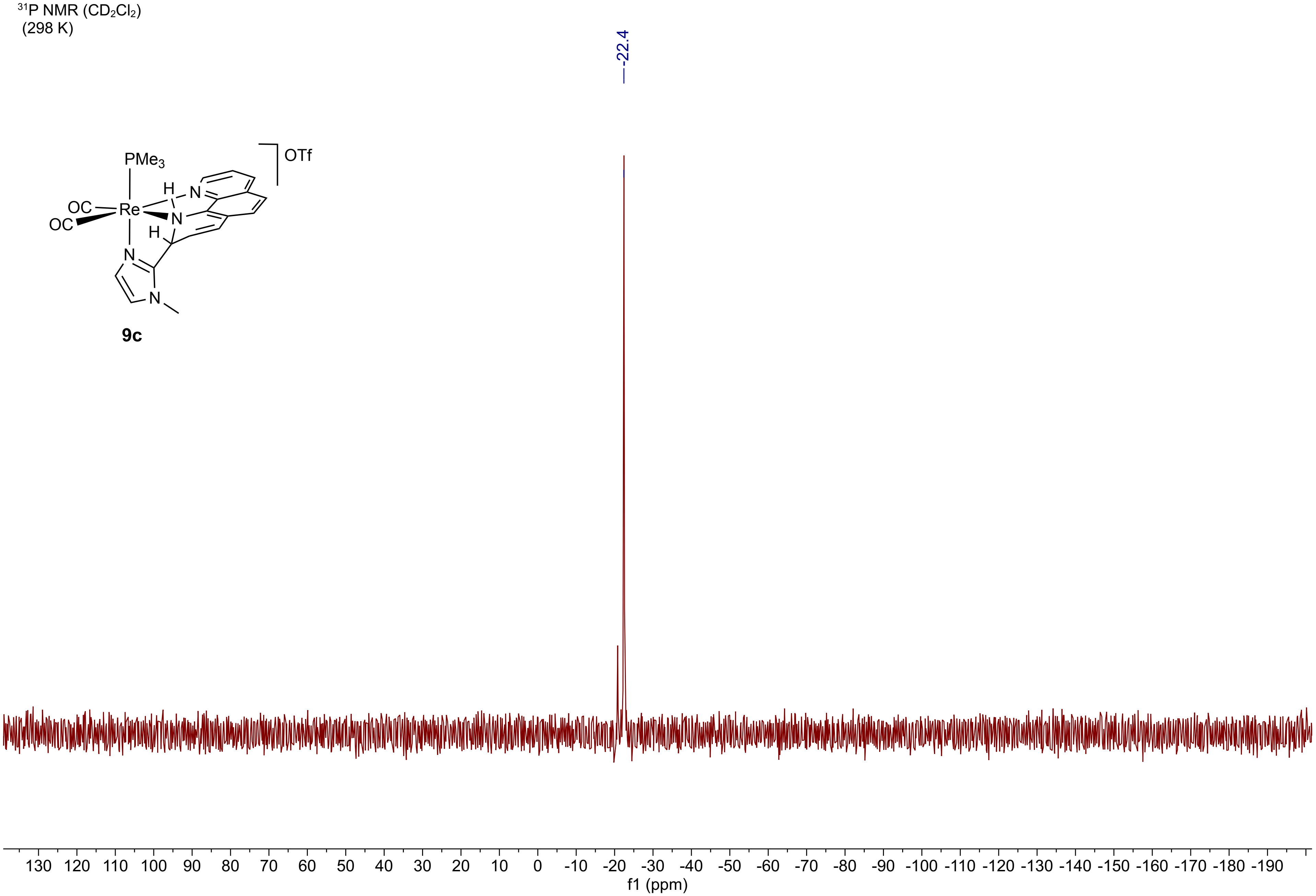

Figure S54. ^{1}H-^{1}H COSY NMR spectrum of compound **9c** in CD_2Cl_2.

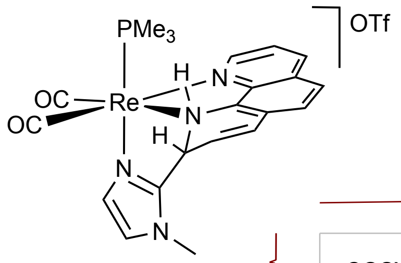

9c

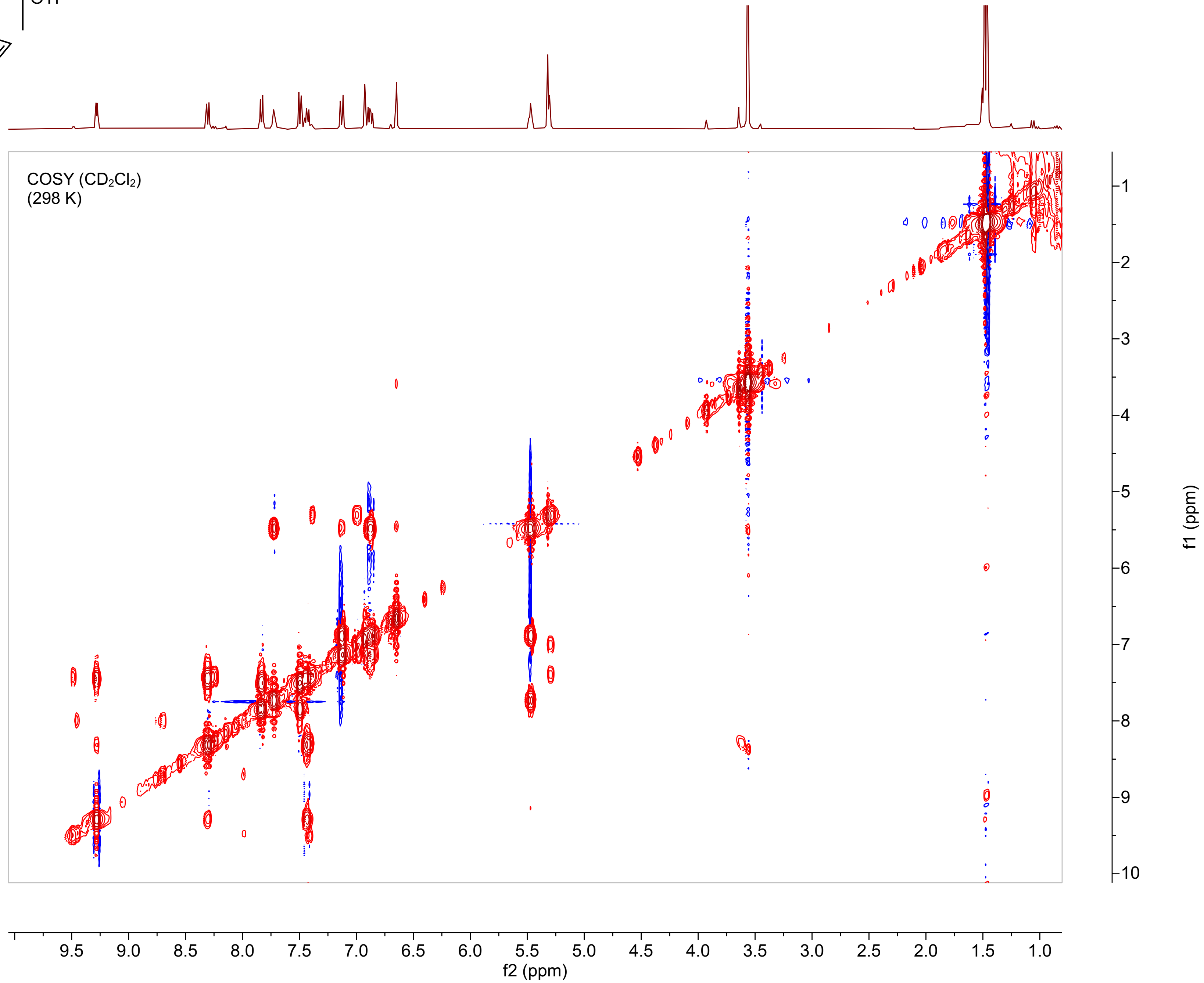

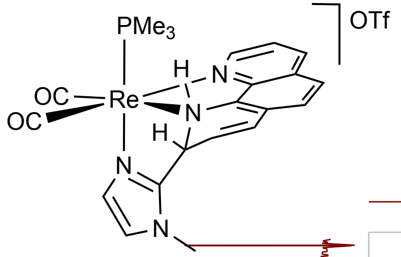

9c

Figure S55. ^{1}H-^{13}C HSQC NMR spectrum of compound **9c** in CD_2Cl_2.

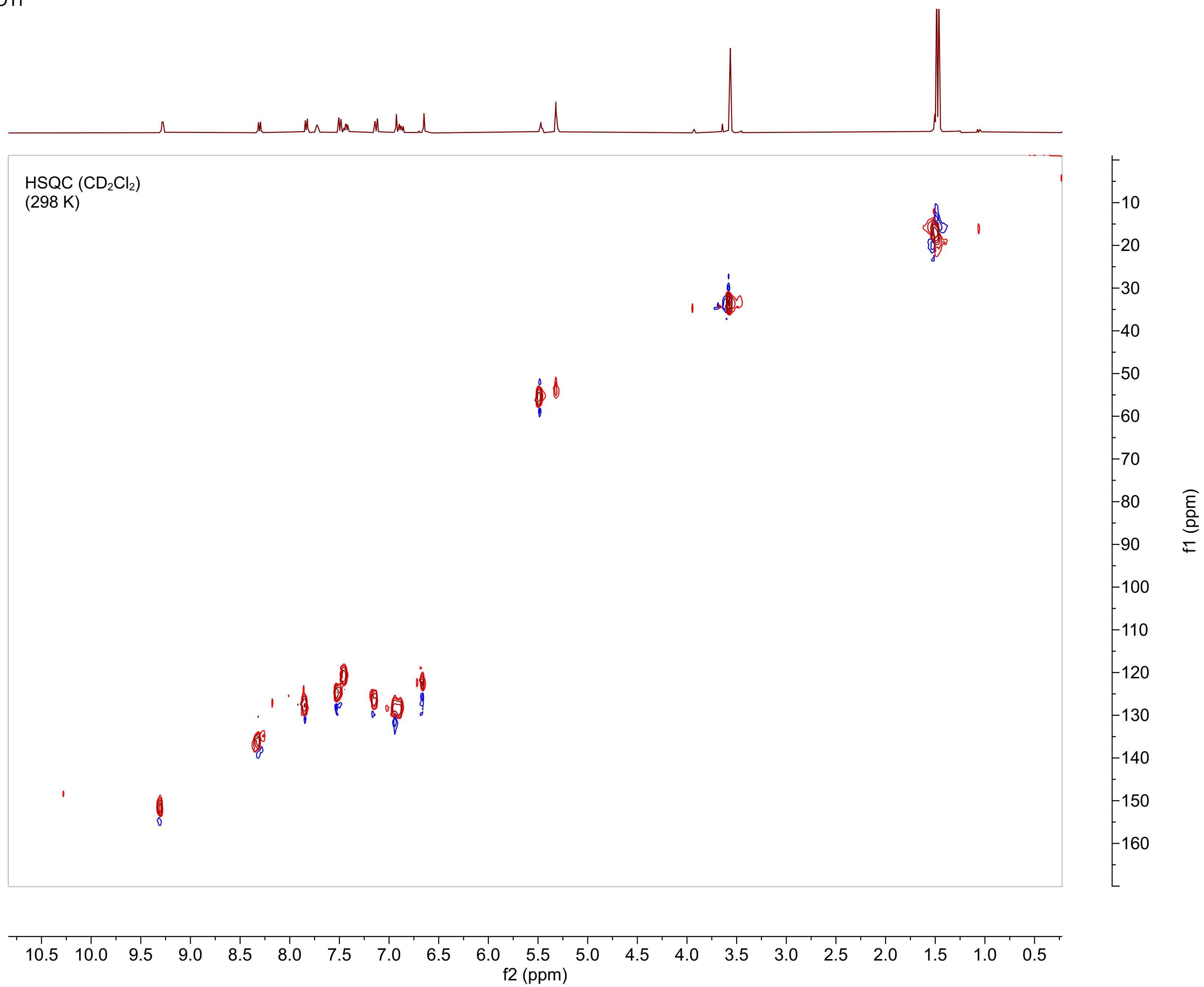

Figure S56. ^{1}H NMR spectrum of compound **9d** in CD_2Cl_2.

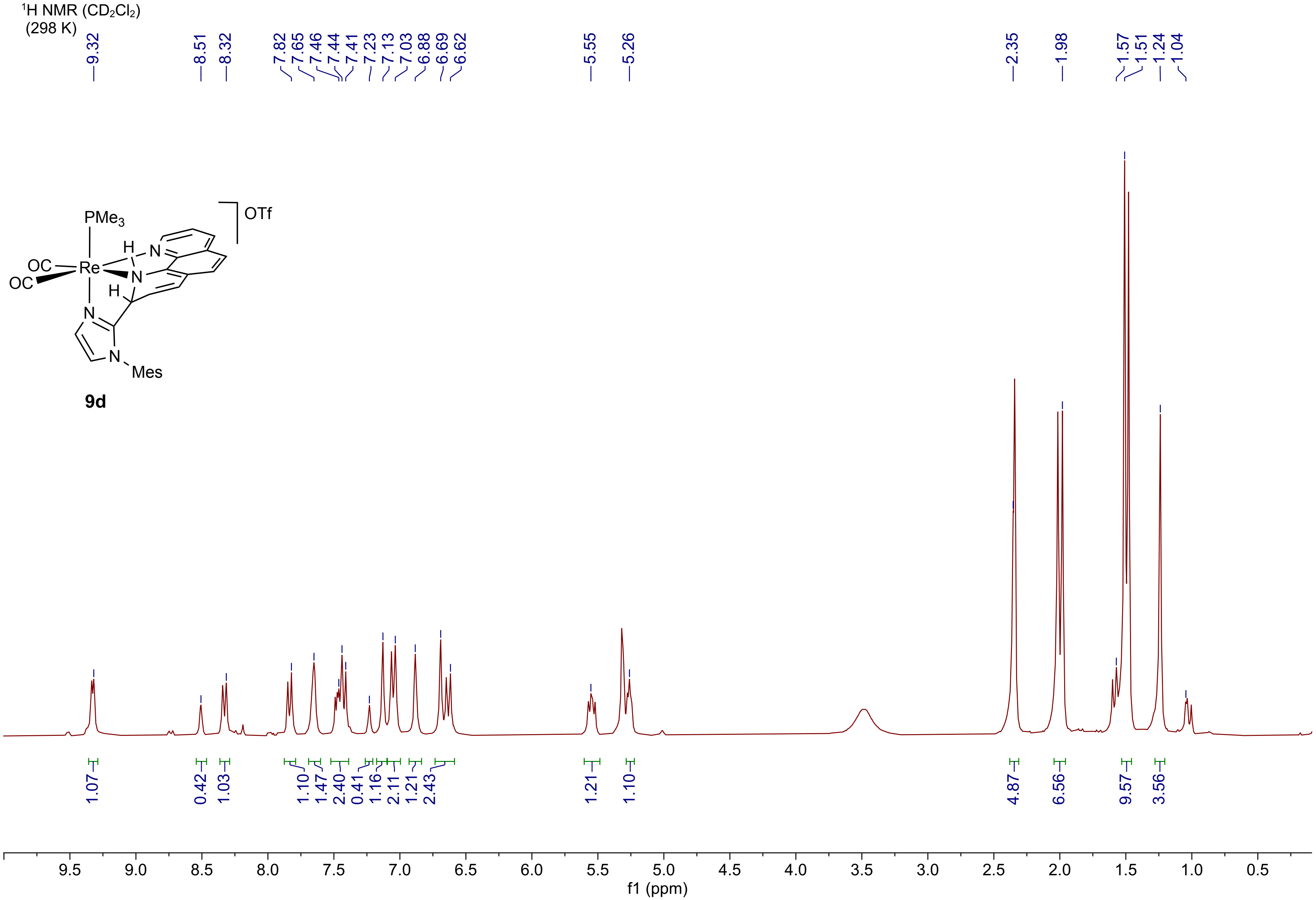

Figure S57. ^{13}C {^{1}H} NMR spectrum of compound **9d** in CD_2Cl_2.

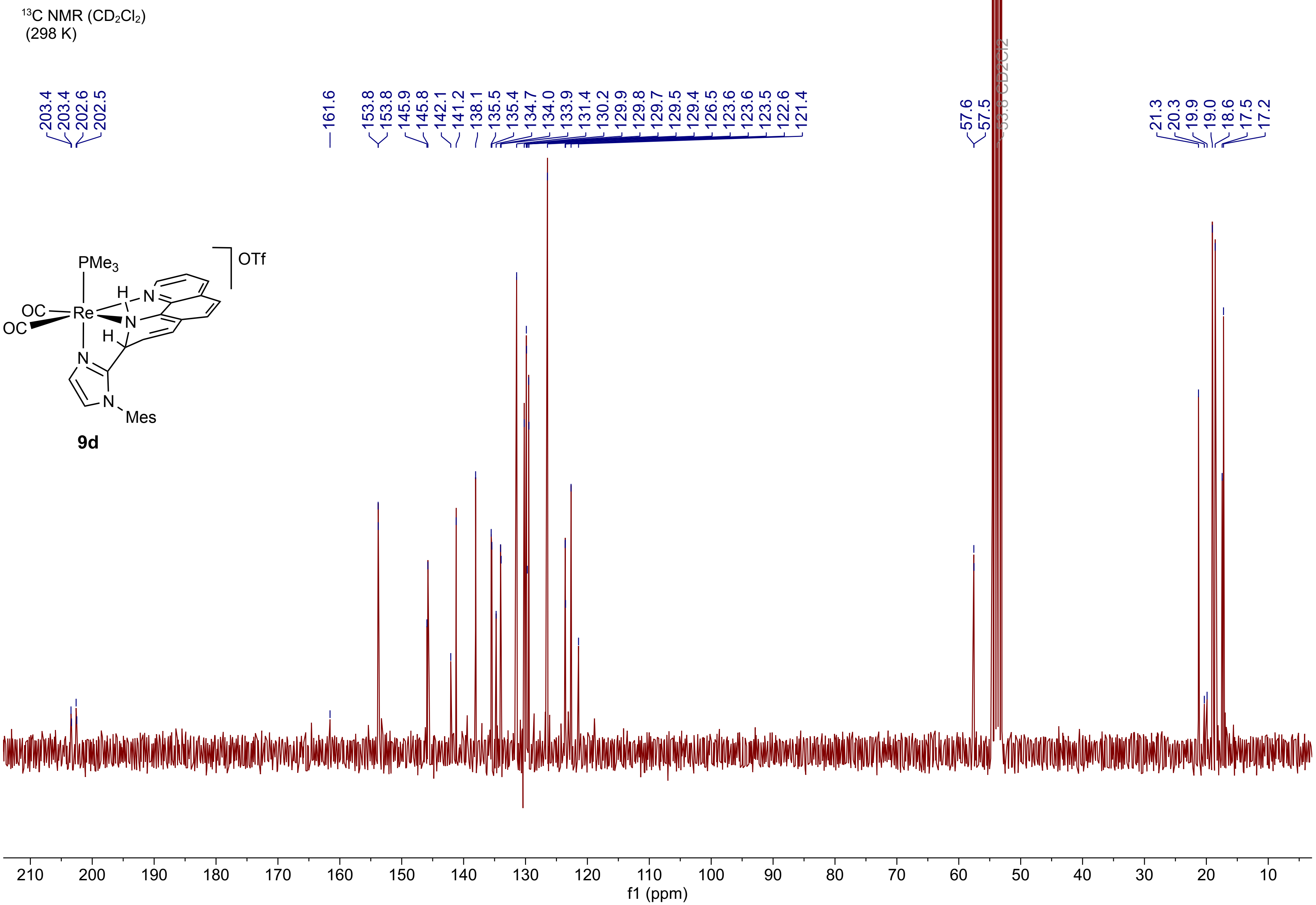

Figure S58. $^{31}P\ \{^1H\}$ NMR spectrum of compound **9d** in CD_2Cl_2.

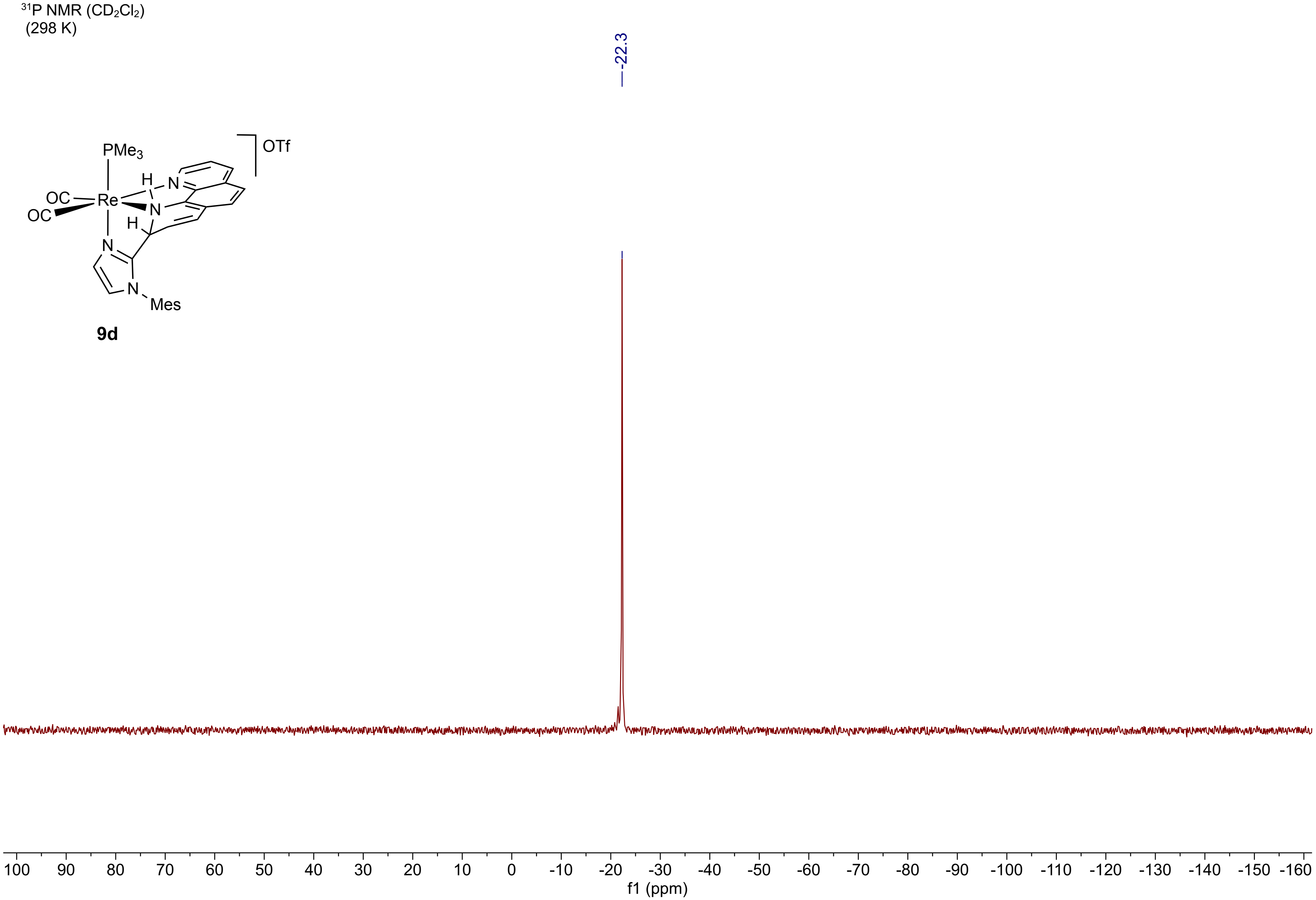

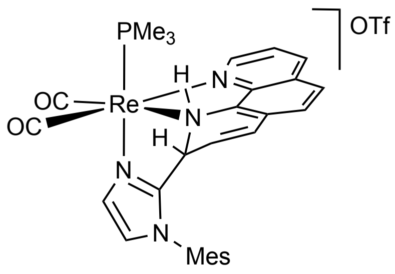

9d

Figure S59. ^{1}H-^{1}H COSY NMR spectrum of compound **9d** in CD_2Cl_2.

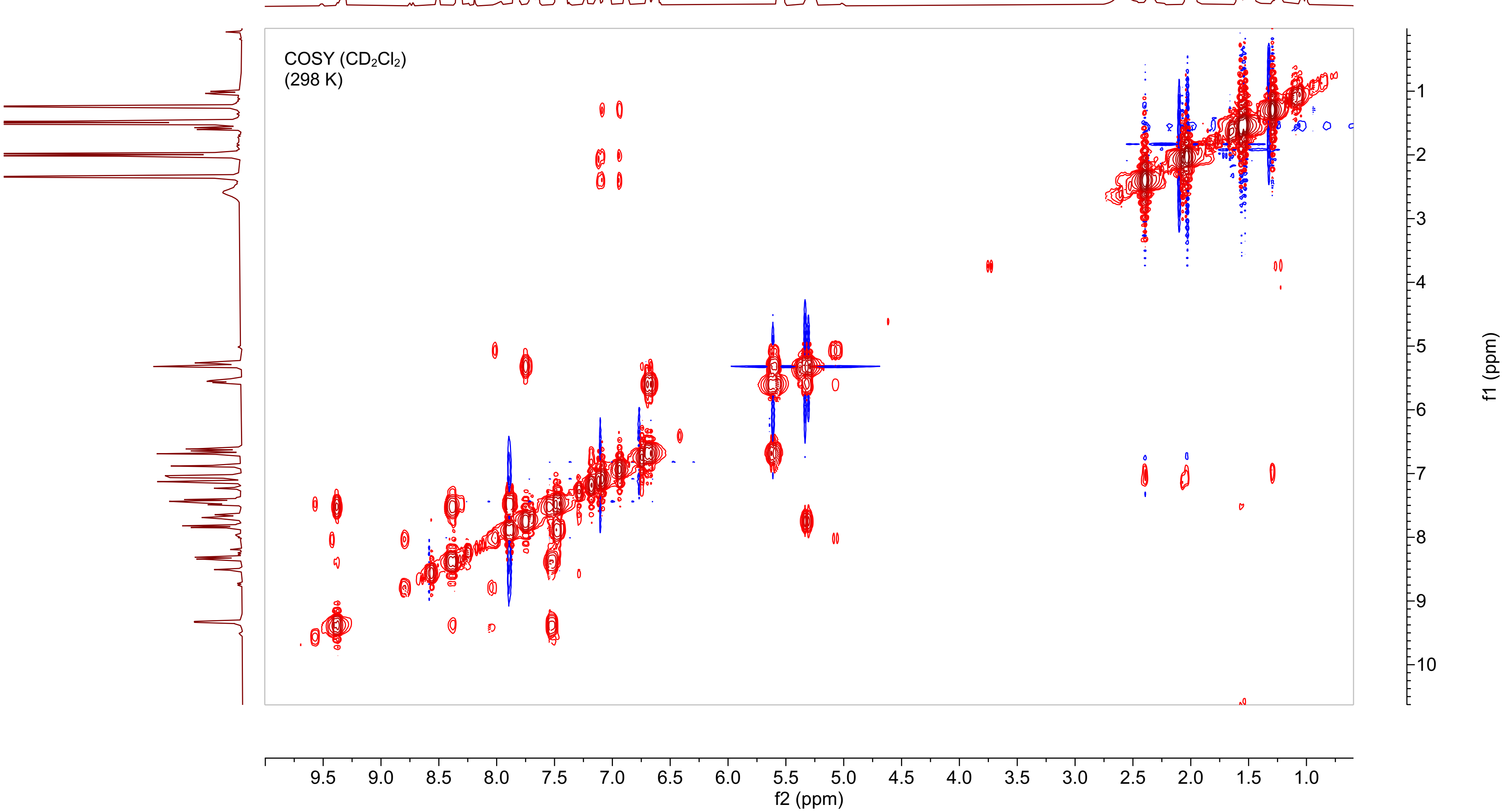

Figure S60. ^{1}H-^{13}C HSQC NMR spectrum of compound **9d** in CD_2Cl_2.

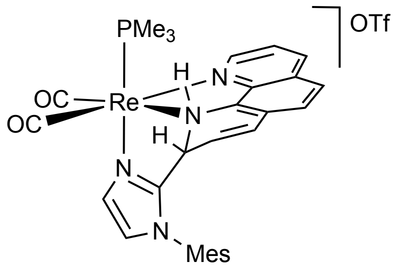

9d

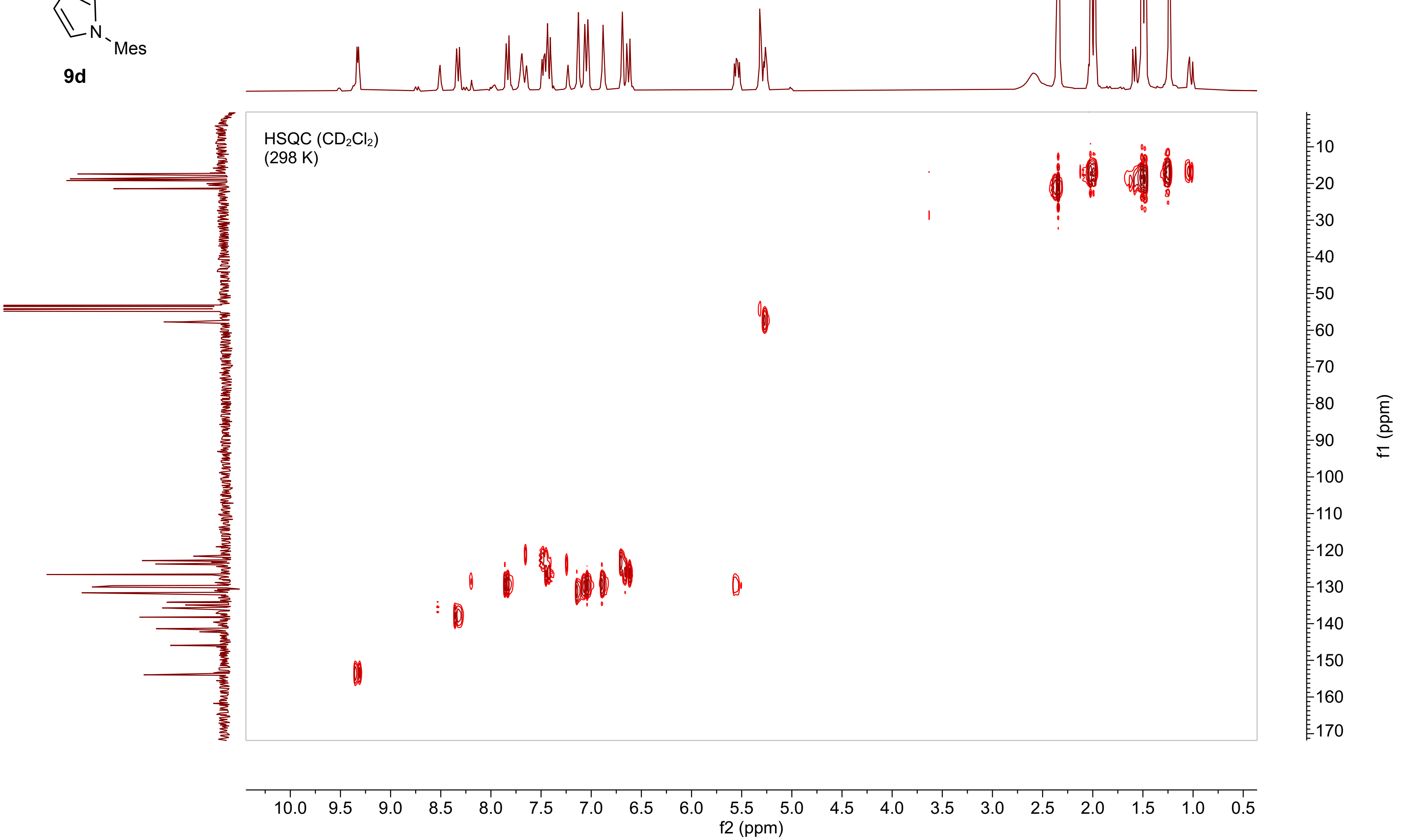

Supplement: Supplementary file 1 — ic3c04434_si_001.pdf [file ic3c04434_si_001.pdf]
